# Ligand-enabled Ir-catalyzed intermolecular diastereoselective and enantioselective allylic alkylation of 3-substituted indoles†
†Electronic supplementary information (ESI) available. CCDC 1060973. For ESI and crystallographic data in CIF or other electronic format see DOI: 10.1039/c5sc01772f


**DOI:** 10.1039/c5sc01772f

**Published:** 2015-06-08

**Authors:** Xiao Zhang, Wen-Bo Liu, Hang-Fei Tu, Shu-Li You

**Affiliations:** a State Key Laboratory of Organometallic Chemistry , Shanghai Institute of Organic Chemistry , Chinese Academy of Sciences , 345 Lingling Lu , Shanghai 200032 , China . Email: slyou@sioc.ac.cn; b Collaborative Innovation Center of Chemical Science and Engineering , Tianjin , China

## Abstract

An Ir-catalyzed asymmetric allylic alkylation of 3-substituted indoles is reported. The reaction provides indoline products containing multiple contiguous stereocenters with high site-, regio-, diastereo- and enantioselectivities in one step from a wide range of readily available starting materials. The key to this method is the high level of diastereocontrol enabled by an iridium catalyst derived from a *N*-aryl phosphoramidite ligand (Me-THQphos, **1c**).

## Abstract 2

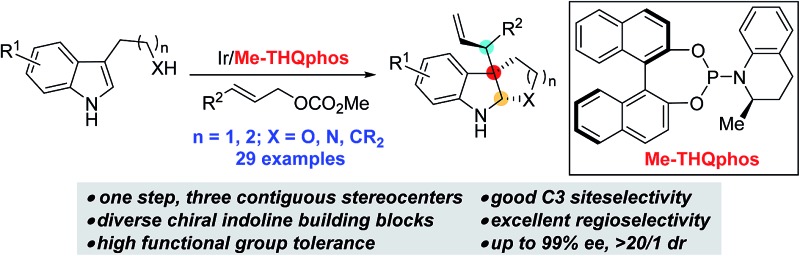
A ligand-enabled Ir-catalyzed diastereoselective and enantioselective allylic alkylation of 3-substituted indoles is reported, providing indoline products containing three contiguous stereocenters in one step with high site-, regio-, diastereo- and enantioselectivities from a wide range of readily available starting materials.

## 

Polycyclic indoline skeletons are present in a variety of alkaloid natural products with diverse biological activities, and many of them bear multiple contiguous stereogenic centers.^1^ A large number of notable methods have been devised toward the synthesis of this privileged structure in the past several decades,^2^ including a chiral pool strategy from tryptophan derivatives,^3^ stereoselective functionalization of oxindoles followed by cyclizations,^4^ and recently, catalytic enantioselective tandem C3-functionalization/cyclization reactions of 3-substituted indoles,^5^ and others.^6^

However, the existing methods are mainly limited to the construction of endocyclic vicinal stereocenters (C2 and C3) of indolines. Given the complexity and importance of indoline alkaloids with an additional tertiary stereocenter (C3′) adjacent to the C3 quaternary center (Fig. 1), the development of efficient methods to install these three contiguous stereocenters in one operation is highly appealing but remains a formidable challenge in organic synthesis.^7^ We envisioned that iridium-catalyzed reactions of 3-substituted indoles with allylic carbonates have the potential to create these stereogenic arrangements in a single step.

**Fig. 1:**
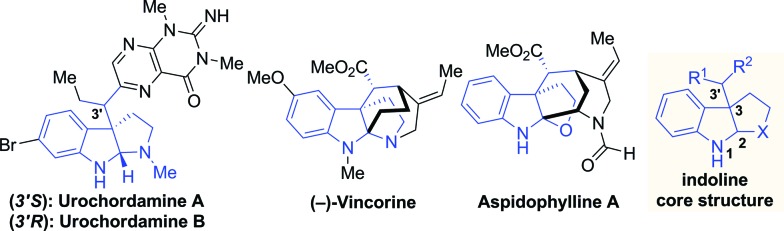
Representative indoline natural products.

To achieve this, the reaction must show high levels of site selectivity for indoles with regard to N1, C3 and the side chain nucleophiles (X),^8^ regioselectivity toward branched or linear (C1′ *vs.* C3′), and enantioselectivity and diastereoselectivity with respect to the new C3–C3′ bond (Scheme 1). Despite the rapid growth of Ir-catalyzed allylic alkylations,^9^ control of the regio- and enantioselectivity of the reaction using sterically hindered nucleophiles has proven to be problematic. Two recently reported examples have provided direct access to 3-substituted pyrroloindolines in one step by iridium-catalysis,^10^ however, these reactions either afford only linear allylated products or lack enantioselectivity. In particular, the diastereocontrol of prochiral nucleophiles represents a significant challenge. To date, only a handful of examples of the intermolecular diastereoselective allylic alkylation of prochiral nucleophiles have been established,^11–14^ through the use of iridium catalysis combined with a judicious choice of additives,^12,13^ or synergistic iridium/amine dual catalysis.^14^ Moreover, the nucleophiles investigated in these accounts were limited to glycine derivatives, azlactones, and enolates. Our group has a strong focus on and interest in addressing the facial selectivity of prochiral nucleophiles by engineering new ligand structures. We have developed an iridacycle complex derived from 2-methyl-1,2,3,4-tetrahydroquinoline phosphoramidite (Me-THQPhos, **1c**) and [Ir(COD)Cl]_2_,^15^ which was found to be an efficient catalyst enabling diastereocontrol in both intra- and intermolecular Ir-catalyzed allylic allylations, by our group^16^ and others.^13^ We recently found that the use of ligand **1c** is crucial to the outcome with regard to regio-, diastereo-, and enantioselectivities in the synthesis of indolines (Scheme 1). Herein, we wish to report our preliminary results on this unprecedented Ir-catalyzed tandem allylic alkylation/cyclization reaction.

**Scheme 1:**
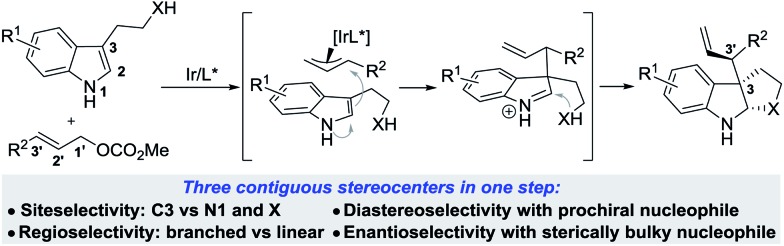
Ir-catalyzed allylic alkylation of 3-substituted indoles.

Since we hypothesized that the catalysts may play a key role in the stereoselectivity, our studies were initiated by investigating phosphoramidite ligands with tryptophol **2a** and cinnamyl carbonate **3a** as model substrates (Table 1). Exposure of the substrates to the catalyst derived from the Feringa ligand **1a** (ref. 17*a*) or the Alexakis ligand **1b** (ref. 17*b*) and 1 equiv. of Cs_2_CO_3_ in THF provided furoindoline **4aa** in good yield as a mixture of diastereomers (2/1–3/1 dr, entries 1 and 2). We envisioned that the low diastereoselectivity may originate from the weak interaction between the sterically encumbered cyclometalated Ir–allyl complex^18^ and the bulky nucleophile. Efforts to improve the diastereoselectivity were made by conducting a survey of several *N*-aryl substituted ligands (entries 3–9). The superior diastereocontrol observed in the cases of the less bulky ligands (*i.e.*, **1c–e**, **1g** and **1i**) compared to that of the more bulky ligands (*i.e.*, **1a**, **1b**, **1f** and **1h**) again suggested the importance of the ligand structure in the facial discrimination of indoles. However, if the ligand's steric bulk was too small (*i.e.*, **1e** and **1g**), the reactivity tended to be lower (28–32% yields, entries 5 and 7). To our delight, ligand **1c** finally emerged as the optimal one for both yield and diastereoselectivity (entry 3).

**Table 1:** Optimization of the reaction conditions^*a*^

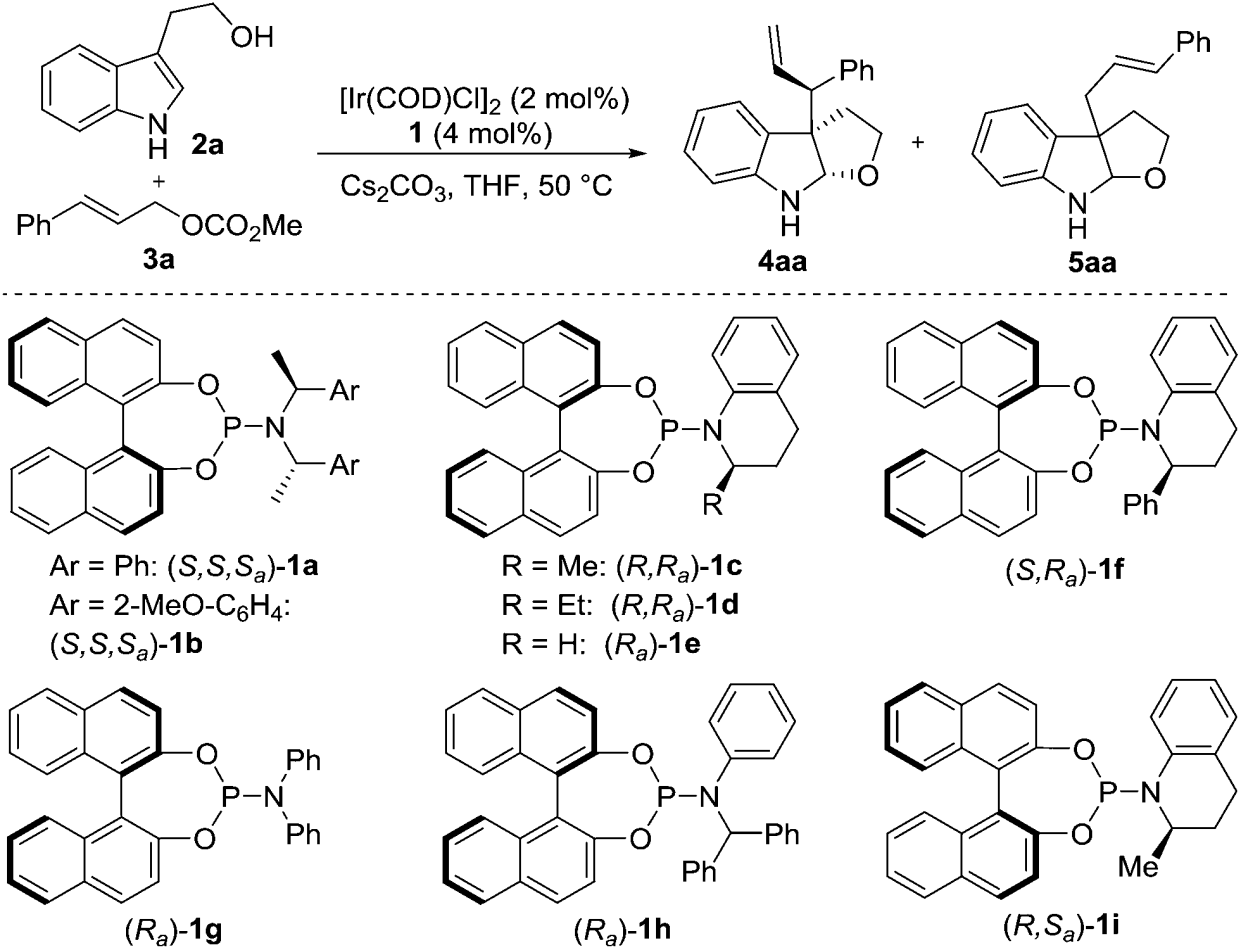					
Entry	Ligand (**1**)	*t* (h)	dr (**4aa**)^*b*^	Yield (**4aa**)^*c*^ (%)	ee^*d*^ (%)
1	**1a**	12	2/1	50	86
2	**1b**	3	3/1	76	90
3	**1c**	5	5/1	84	87
4	**1d**	9	5/1	84	86
5	**1e**	24	10/1	28	90
6	**1f**	3	1/1	72	90
7	**1g**	24	9/1	32	92
8^*e*^	**1h**	3	2/1	86	90
9	**1i**	24	9/1	36	–90
10^*f*^	**1c**	6	8/1	82	90
11^*f*^ ^,^ ^*g*^	**1c**	12	15/1	70	95
12^*f*^ ^,^ ^*g*^ ^,^ ^*h*^	**1c**	12	>20/1	82	95

^*a*^The reaction was performed with 0.2 mmol of **2a**, 1.5 equiv. of **3a** and 1 equiv. of Cs_2_CO_3_. Ir-catalyst was prepared *in situ* with *n*-PrNH_2_.

^*b*^Determined by ^1^H NMR of the crude reaction mixture. Unless otherwise noted, **4aa**/**5aa** = >95/5.

^*c*^Isolated yield.

^*d*^ee of **4aa** was determined using HPLC analysis (Chiralpak IC).

^*e*^ **4aa**/**5aa** = 90/10.

^*f*^1 equiv. of Et_3_N was used with **2a**/**3a** = 2/1.

^*g*^At rt.

^*h*^Ir-catalyst was prepared *in situ* with TBD.

Further optimization was conducted by screening various bases, temperatures, substrate ratios, and the activators used in the catalyst *in situ* preparation (entries 10–12).^19–21^ These results revealed that the use of excess nucleophile and the combination of Et_3_N and catalyst generated *in situ* by TBD could provide **4aa** in 82% yield with >20/1 dr and 95% ee at room temperature (entry 12).

With the optimized reaction conditions in hand, we next studied the scope of different allylic carbonates amenable to the chemistry (Scheme 2). Carbonates containing either electron-donating (4-MeO, 4-Me, 3-MeO and 3-Me) or electron- withdrawing (4-F, 4-Cl and 4-Br) substituents on the phenyl ring were well tolerated and delivered the corresponding products efficiently (**4aa–4ah**, 66–82% yields, 10/1–>20/1 dr and 94–97% ee).

**Scheme 2:**
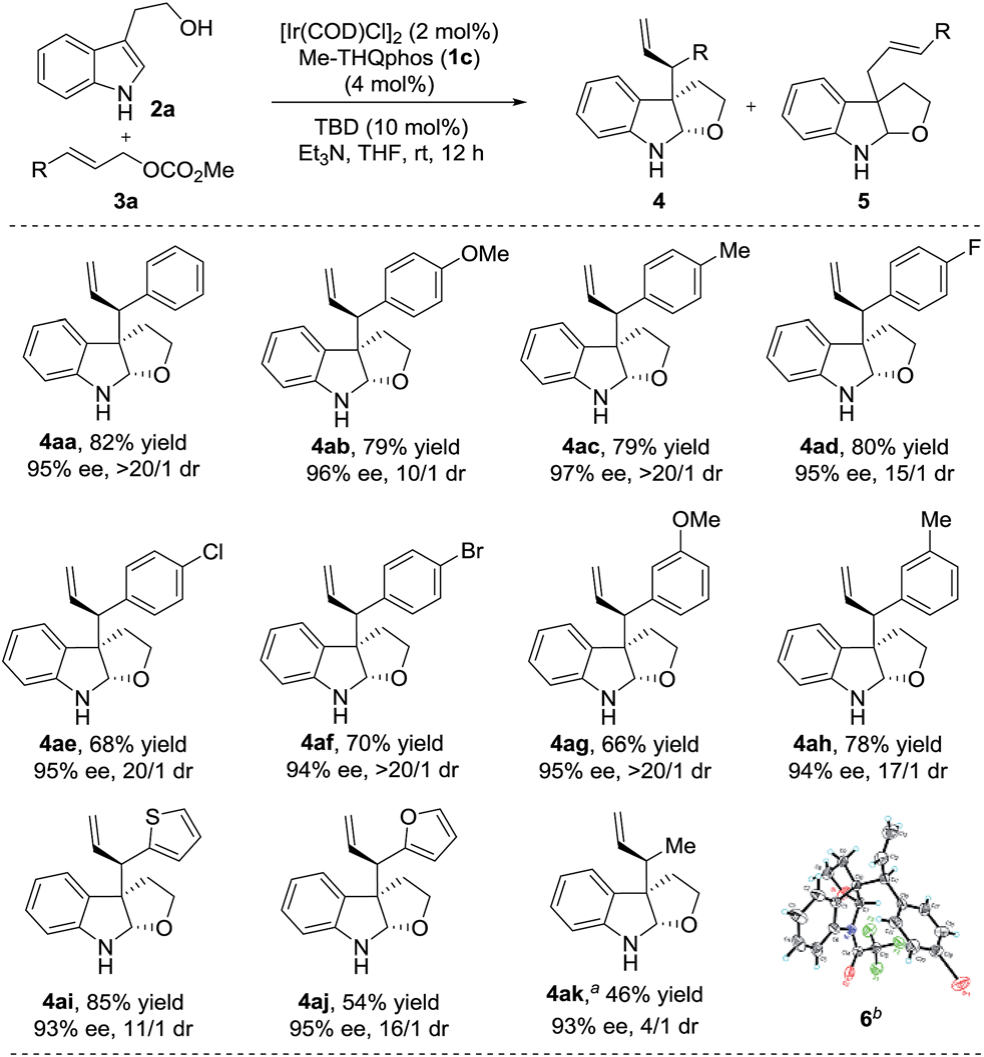
Substrate scope of electrophiles. The reactions were performed under the conditions of Table 1, entry 12. The yields of isolated product **4** are shown. The dr was determined using ^1^H NMR of the crude reaction mixture and the ee was determined using chiral HPLC. Unless otherwise noted, **4**/**5** = >95/5. ^*a*^Reaction time was 18 h and **4**/**5** = 85/15. ^*b*^The absolute configuration of **4af** was determined using X-ray analysis of its trifluoroacetyl-protected derivative **6**.^19^ The absolute configuration of all other products was determined by analogy.

In addition, heteroaryl allylic carbonates, such as 2-thienyl and 2-furanyl, were also compatible with this transformation (**4ai** and **4aj**, 11/1–16/1 dr and 93–95% ee). Gratifyingly, the reaction with methyl crotyl carbonate led to the desired product **4ak** in excellent enantioselectivity (93% ee), albeit with a slightly lower dr than for aromatic substituted carbonates. To the best of our knowledge, this is the first example of the application of an aliphatic allylic carbonate in the Ir-catalyzed allylic alkylation of prochiral nucleophiles which can achieve good diastereo- and enantioselectivities.

Subsequently, we set out to investigate the scope of nucleophiles. A variety of indoles with diverse substituents were examined and the results are summarized in Scheme 3.^22^ Different substituents (4-Me, 5-MeO, 5-Cl, 5-Br and 6-Cl) on the tryptophols were well tolerated in all cases delivering the desired furoindoline products smoothly (**4ba–4fa**, 50–83% yields, 10/1–>20/1 dr and 91–97% ee). Pyranoindoline **4ga** was also obtained in good yield and selectivities. In addition, employing tryptamines with varied protecting groups (CO_2_Me, CO_2_Et, Boc, CO_2_^*i*^Bu and Ts) on the nitrogen of the linkage and halogen substituents on the aromatic ring all resulted in the corresponding products (58–75% yields, **4ha–4la**). Upon treatment with LiAlH_4_, the N–Me substituted compound **4ma** was obtained in 40% yield in two steps.^19^ The excellent diastereo- and enantioselectivities (14/1–>20/1 dr and 96–99% ee) of these particularly valuable pyrroloindolines demonstrate the potential of this method in the synthesis of complex alkaloid natural products. We also found that indoles with carbon nucleophile side chains were well compatible and proceeded to give cyclopentaneindoline products **4na–4qa** under the same conditions in moderate yields (42–67%) with excellent stereoselectivities (19/1–>20/1 dr and 97–99% ee).^23^

**Scheme 3:**
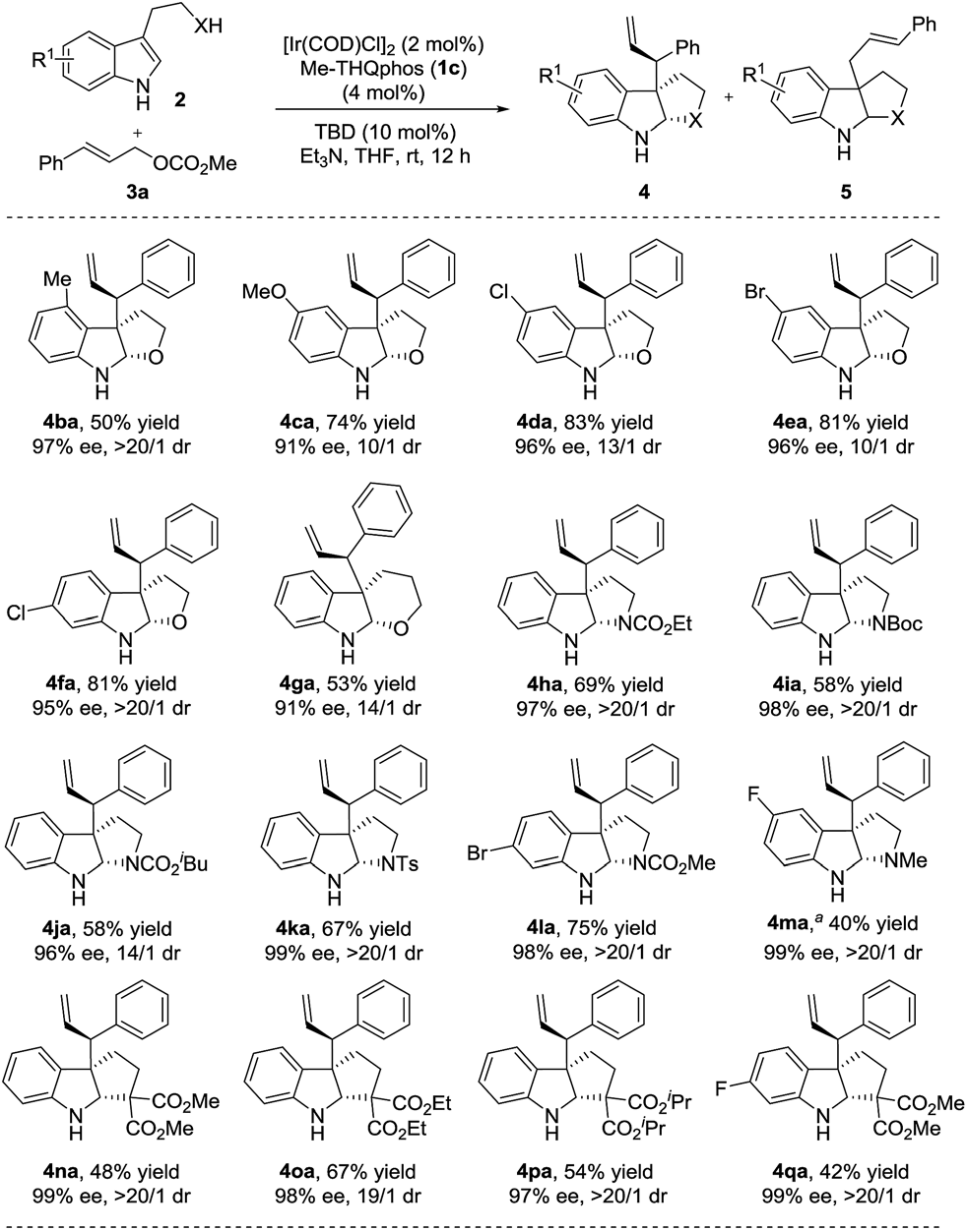
Substrate scope of nucleophiles. The reactions were performed under the conditions of Table 1, entry 12. The yields of isolated product **4** are shown. The dr was determined using ^1^H NMR of the crude reaction mixture and the ee was determined using chiral HPLC. Unless otherwise noted, **4**/**5** = >95/5. ^*a*^ **4ma** was obtained through an allylic alkylation of methyl (2-(5-fluoro-1*H*-indol-3-yl)ethyl)carbamate and a subsequent reduction using LiAlH_4_.^19^

Remarkably, the reaction also tolerates chiral nucleophiles such as tryptophan derivatives. Subjecting the l-tryptophan derivative (–)-**2r** to the reaction conditions afforded the corresponding product *cis*-**4ra** with a good dr and in a moderate yield (Scheme 4a). Similarly, employment of the d-tryptophan derivative (+)-**2r** led to indoline *trans*-**4ra** in a slightly better yield, albeit as a 3.5/1 mixture of diastereoisomers (Scheme 4b). Both of the major isomers of the above reactions shared the identical stereochemical configuration at the three newly formed centers (C2, C3 and C3′), suggesting that ligand-enabled catalyst control is responsible for establishing the absolute stereochemistry at these three centers,^15*b*^ while a pre-existing chiral center in the substrate could also bias the stereochemical outcomes albeit to a lesser extent.

**Scheme 4:**
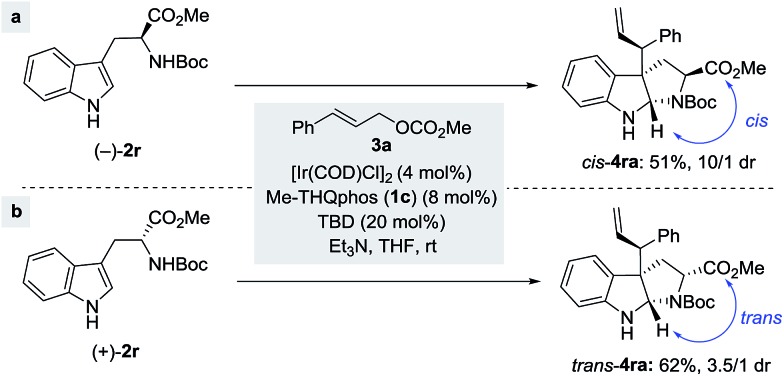
Allylic alkylation of *N*-Boc protected tryptophan methyl ester.

In order to demonstrate the utility of this method, a gram-scale reaction was carried out, achieving good yield and selectivities (Scheme 5a). Furthermore, hydroboration/oxidation of the terminal olefin in **4aa** was achieved without a loss of enantiomeric purity (eqn (1), Scheme 5b). Finally, Pt/C-catalyzed hydrogenation afforded the corresponding furoindoline **8** and pyrroloindoline **9** (eqn (2), Scheme 5b), with excellent diastereoselectivities and enantioselectivities, showcasing the synthetic potential of this protocol.

**Scheme 5:**
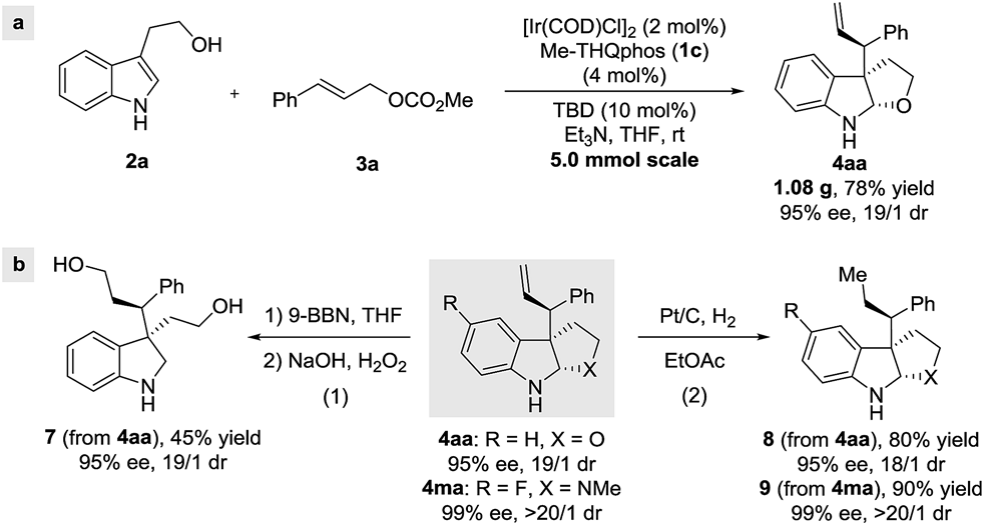
Gram-scale synthesis and product transformations.

## Conclusions

In conclusion, we have developed an iridium-catalyzed intermolecular allylic alkylation reaction of 3-substituted indoles with high site-, regio-, diastereo-, and enantioselectivities. The transformation, in which three stereocenters are formed in a single step, provides efficient access to structurally complex polycyclic indolines from simple starting materials. We also demonstrate that stereoselectivity is mainly controlled by the catalyst when tryptophan derivatives are used. Critical to this strategy is the employment of the Me-THQPhos (**1c**) ligand. Further mechanistic studies and the application of this method in natural product synthesis is ongoing in our laboratory.
