# Supplementary material for: Ligand-enabled Ir-catalyzed intermolecular diastereoselective and enantioselective allylic alkylation of 3-substituted indoles
Source: Chem Sci. 2015 Jun 8;6(8):4525–9. doi: 10.1039/c5sc01772f (PMC5666512; doi:10.1039/c5sc01772f)
Supplement: Supplementary file 1 [file SC-006-C5SC01772F-s001.pdf]

Electronic Supplementary Material (ESI) for Chemical Science.
This journal is © The Royal Society of Chemistry 2015

Supporting Information for

Ligand-Enabled Ir-Catalyzed Intermolecular Diastereoselective and Enantioselective Allylic Alkylation of 3-Substituted Indoles

Xiao Zhang, Wen-Bo Liu, Hang-Fei Tu, and Shu-Li You*

State Key Laboratory of Organometallic Chemistry, Shanghai Institute of Organic Chemistry, Chinese Academy of Sciences, 345 Lingling Lu, Shanghai 200032, China; Collaborative Innovation Center of Chemical Science and Engineering (Tianjin)

E-mail: slyou@sioc.ac.cn

Table of Contents

General methods	S2
Optimization of the reaction conditions	S2-3
Experimental details and characterization data	S3-18
Procedure for the synthesis of **4ma**	S18-19
Ir-catalyzed asymmetric allylic alkylation of tryptophan derivatives	S19-21
Gram-scale synthesis of **4aa**	S21-22
Procedure for the synthesis of **7**	S22-23
Procedure for Pt/C catalyzed hydrogenation of compound **4aa**	S23-24
Procedure for the synthesis of **9**	S24
Determination of the absolute configuration	S25-28
Copies of NMR spectra and HPLC chromatographs	S29-134

General methods. Unless stated otherwise, all reactions were carried out in flame-dried glassware under a dry argon atmosphere. All solvents were freshly distilled according to standard methods prior to use.

^{1}H and ^{13}C NMR spectra were recorded on a Varian instrument (300 MHz and 75 MHz, 400 MHz and 100 MHz, respectively) and internally referenced to tetramethylsilane signal or residual protio solvent signals. ^{19}F NMR spectra were recorded on Varian instrument (282 MHz and 376 MHz, respectively) and referenced relative to $CFCl_3$. Data for ^{1}H NMR are recorded as follows: chemical shift (δ, ppm), multiplicity (s = singlet, d = doublet, t = triplet, m = multiplet or unresolved, br = broad singlet, coupling constant(s) in Hz, integration). Data for ^{13}C NMR are reported in terms of chemical shift (δ, ppm).

Table S1. Optimization of the reaction conditions[a]

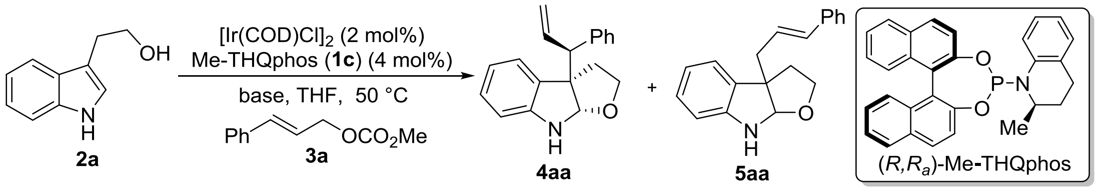

entry	base	t (h)	conv. (%)[b]	dr (**4aa**)[b]	yield (**4aa**) (%)[c]	ee (%)[d]
1	Cs_2CO_3	5	>95	5/1	84	87
2	-	36	45	9/1	42	86
3	K_2CO_3	36	72	4/1	58	81
4	K_3PO_4	36	54	>20/1	15	80
5	DBN	24	-	9/1	-	91
6	DBU	15	62	10/1	-	90
7	DABCO	24	32	6/1	-	83
8	P.S.	24	23	12/1	-	59
9	Et_3N	24	90	8/1	64	87
10[e]	Et_3N	6	>95	8/1	82	90
11[e,f]	Et_3N	12	>95	15/1	70	95

12[e,f,g]	Et_3N	12	>95	>20/1	82	95

[a] 2.0 mol% of $[Ir(COD)Cl]_2$, 4.0 mol% of **1c**, 100 mol% of base, 150 mol% of **3a** and 100 mol% of **2a** in THF at 50 °C. Unless noted, Ir-catalyst was prepared *via* *n*-$PrNH_2$ activation. [b] Determined by ^1H NMR of the crude reaction mixture. Unless noted, **4aa**/**5aa**: >97/3. [c] Isolated yield. [d] Ee of **4aa** was determined by HPLC analysis (Chiralpak IC). [e] 100 mol% of **3a** and 200 mol% of **2a** were used. [f] The reaction was performed at rt. [g] Ir-catalyst was *in situ* prepared by TBD (10 mol%) activation.

General procedure for Ir-catalyzed intermolecular diastereoselective and enantioselective allylic alkylation of 3-substituted indoles

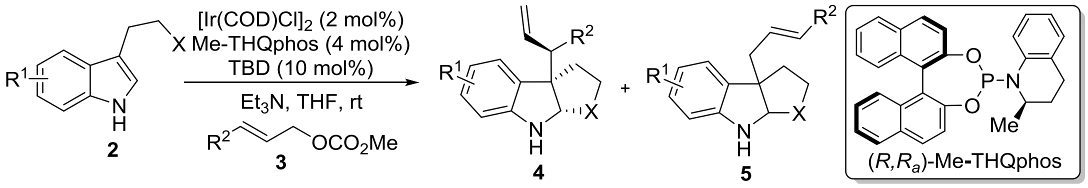

A flame-dried Schlenk tube was cooled to room temperature and filled with argon. To this flask were added $[Ir(COD)Cl]_2$ (5.4 mg, 0.008 mmol, 2.0 mol%), (*R*,*R*$_a$)-**1c** (7.4 mg, 0.016 mmol, 4.0 mol%), TBD (5.6 mg, 0.04 mmol, 10 mol%), and THF (1.0 mL). The reaction mixture was stirred at room temperature for 10 min, generating an orange solution. To another Schlenk tube were added indole derivative **2** (0.8 mmol, 200 mol%), allylic carbonate **3** (0.40 mmol, 100 mol%), Et_3N (40.4 mg, 0.40 mmol, 100 mol%), and THF (3 mL). Then, the above pre-formed catalyst solution was transferred to this tube. The reaction mixture was stirred at room temperature until allylic carbonate **3** was fully consumed (monitored by TLC). Upon completion of the reaction, the mixture was concentrated under reduced pressure. The ratio of **4**/**5** and diastereoselectivity of **4** were determined by ^1H NMR of the crude reaction mixture. Then the residue was purified by silica gel column chromatography (PE/EA = 20/1) to afford the desired product **4**. The characterization data of the products are summarized below.

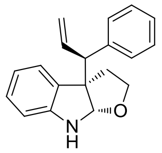

4aa. Yellow oil, 82% yield, 95% ee [Daicel Chiralpak IC (0.46 cm x 25 cm), *n*-hexane/2-propanol = 100/1, *v* = 1.0 mL·min^{-1}, λ = 254 nm, t (minor) = 19.96 min, t (major) = 26.60 min]; $[\alpha]_D^{21}$ = -22.4 (c = 1.0, $CHCl_3$). ^{1}H NMR (300 MHz, $CDCl_3$, minor diastereomer indicated by *) δ 2.17 (dd, *J* = 4.5, 11.7 Hz, 1H), 2.33 (dt, *J* = 7.2, 11.7 Hz, 1H), 3.46-3.55 (m, 2H), 3.96 (t, *J* = 7.5 Hz, 1H), 4.30 (br s, 1H), 5.09 (d, *J* = 17.1 Hz, 1H), 5.17 (d, *J* = 10.2 Hz, 1H), 5.39 (s, 1H), 5.44 (s, 1H*), 6.34 (ddd, *J* = 8.7, 10.2, 16.8 Hz, 1H), 6.47 (d, *J* = 7.8 Hz, 1H), 6.68-6.73 (m, 1H), 6.88-6.91 (m, 3H), 7.06 (dt, *J* = 1.2, 7.8 Hz, 1H), 7.15-7.19 (m, 3H). ^{13}C NMR (100 MHz, $CDCl_3$) δ 39.2, 57.4, 61.9, 67.6, 96.6, 108.4, 117.1, 118.5, 124.8, 126.7, 128.1, 128.4, 128.6, 130.2, 137.9, 140.6, 150.5. IR (thin film): ν_{max} (cm^{-1}) = 3403, 3348, 2970, 2867, 1606, 1470, 1412, 1360, 1314, 1241, 1154, 1109, 1040, 1011, 957, 915, 744, 722, 700, 663; HRMS (ESI) calcd for $C_{19}H_{20}NO$ $[M+H]^+$: 278.1539. Found: 278.1529.

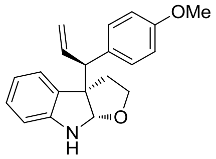

4ab. Yellow oil, 79% yield, 96% ee [Agilent 1260 Infinity Analytical SFC system Daicel Chiralpak AD-H (0.46 cm x 15 cm), CO_2/2-propanol = 90/10, *v* = 1.3 mL·min^{-1}, λ = 214 nm, t (minor) = 22.01 min, t (major) = 36.45 min]; $[\alpha]_D^{22}$ = -0.70 (c = 1.0, $CHCl_3$). ^{1}H NMR (400 MHz, $CDCl_3$, minor diastereomer indicated by *) δ 2.16 (dd, *J* = 4.8, 12.0 Hz, 1H), 2.27-2.35 (m, 1H), 3.47-3.54 (m, 2H), 3.74 (s, 3H), 3.78 (s, 3H*), 3.96 (t, *J* = 7.6 Hz, 1H), 4.36 (s, 1H), 4.54 (s, 1H*), 5.05 (d, *J* = 17.2 Hz, 1H), 5.15 (d, *J* = 10.4 Hz, 1H), 5.37 (s, 1H), 5.41 (s, 1H*), 5.93-6.01 (m, 1H*), 6.27-6.36 (m, 1H), 6.48 (d, *J* = 8.0 Hz, 1H), 6.54 (d, *J* = 7.6 Hz, 1H*), 6.69-6.72 (m, 3H), 6.80 (d, *J* = 8.4 Hz, 2H), 6.92 (d, *J* = 7.6 Hz, 1H), 7.06 (t, *J* = 6.8 Hz, 1H). ^{13}C NMR (100 MHz, $CDCl_3$) δ 39.2, 55.0, 56.6, 61.9, 67.6, 96.6, 108.4, 113.4, 116.8, 118.4, 124.8, 128.3, 129.5, 130.3, 132.7, 138.1, 150.5, 158.2. IR (thin film): ν_{max} (cm^{-1})

= 3400, 2966, 2869, 1608, 1510, 1467, 1412, 1360, 1306, 1243, 1179, 1108, 1034, 953, 916, 832, 808, 742, 656; HRMS (ESI) calcd for $C_{20}H_{22}NO_2$ $[M+H]^+$: 308.1645. Found: 308.1639.

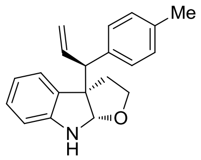

4ac. Yellow oil, 79% yield, 97% ee [Daicel Chiralpak IC (0.46 cm x 25 cm), *n*-hexane/2-propanol = 100/1, *v* = 1.0 mL·min^{-1}, λ = 254 nm, t (minor) = 19.57 min, t (major) = 28.16 min]; $[\alpha]_D^{23}$ = -7.5 (c = 1.0, $CHCl_3$). ^1H NMR (400 MHz, $CDCl_3$, minor diastereomer indicated by *) δ 2.17 (dd, *J* = 4.8, 12.0 Hz, 1H), 2.28 (s, 3H), 2.30-2.36 (m, 1H), 3.48-3.54 (m, 2H), 3.97 (t, *J* = 7.6 Hz, 1H), 4.33 (br s, 1H), 5.07 (d, *J* = 17.2 Hz, 1H), 5.14 (d, *J* = 10.0 Hz, 1H), 5.39 (s, 1H), 5.42 (s, 1H*), 6.32 (ddd, *J* = 8.4, 10.0, 16.8 Hz, 1H), 6.49 (d, *J* = 8.0 Hz, 1H), 6.72 (t, *J* = 6.8 Hz, 1H), 6.78 (d, *J* = 8.4 Hz, 2H), 6.94 (d, *J* = 7.2 Hz, 1H), 6.98 (d, *J* = 8.0 Hz, 2H), 7.06 (t, *J* = 7.2 Hz, 1H). ^{13}C NMR (100 MHz, $CDCl_3$) δ 21.0, 39.3, 57.1, 61.9, 67.6, 96.6, 108.4, 116.9, 118.5, 124.8, 128.3, 128.5, 128.8, 130.4, 136.2, 137.6, 138.1, 150.5. IR (thin film): ν_{max} (cm^{-1}) = 3407, 3352, 2921, 2853, 1607, 1512, 1467, 1411, 1359, 1314, 1241, 1108, 1066, 1041, 1014, 957, 916, 801, 739, 657; HRMS (ESI) calcd for $C_{20}H_{22}NO$ $[M+H]^+$: 292.1696. Found: 292.1685.

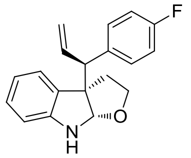

4ad. Yellow oil, 80% yield, 95% ee [Agilent 1260 Infinity Analytical SFC system Daicel Chiralpak AD-H (0.46 cm x 15 cm), CO_2/2-propanol = 95/5, *v* = 1.3 mL·min^{-1}, λ = 230 nm, t (minor) = 24.01 min, t (major) = 38.60 min]; $[\alpha]_D^{25}$ = -14.2 (c = 1.0, $CHCl_3$). ^1H NMR (400 MHz, $CDCl_3$, minor diastereomer indicated by *) δ 2.18 (dd, *J* = 4.4, 11.6 Hz, 1H), 2.31 (dt, *J* = 7.2, 11.6 Hz, 1H), 3.47-3.55 (m, 2H), 3.98 (t, *J* = 8.0 Hz, 1H), 4.35 (br s, 1H), 5.07 (d, *J* = 16.8 Hz, 1H), 5.18 (d, *J* = 10.0

Hz, 1H), 5.33 (s, 1H), 5.43 (s, 1H*), 6.31 (ddd, *J* = 8.0, 10.0, 16.8 Hz, 1H), 6.47 (d, *J* = 8.0 Hz, 1H), 6.71 (dt, *J* = 0.8, 7.6 Hz, 1H), 6.80-6.87 (m, 4H), 6.92 (d, *J* = 7.6 Hz, 1H), 7.06 (dt, *J* = 1.2, 8.0 Hz, 1H). ^{13}C NMR (100 MHz, $CDCl_3$) δ 39.2, 56.6, 61.8, 67.7, 96.6, 108.5, 114.8 (*J* = 21.3 Hz), 117.3, 118.5, 124.7, 128.6, 129.8, 130.0 (*J* = 7.6 Hz), 136.3 (*J* = 3.1 Hz), 137.7, 150.5, 161.6 (*J* = 244.4 Hz). ^{19}F NMR (376 MHz, $CDCl_3$) δ -115.91 (m). IR (thin film): ν_{max} (cm^{-1}) = 3346, 2946, 2869, 1605, 1507, 1469, 1412, 1314, 1222, 1159, 1109, 1042, 1012, 959, 918, 836, 808, 740, 656; HRMS (ESI) calcd for $C_{19}H_{19}FNO$ $[M+H]^+$: 296.1445. Found: 296.1444.

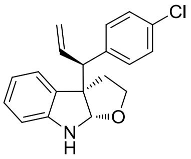

4ae. Yellow oil, 68% yield, 95% ee [Daicel Chiralpak IC (0.46 cm x 25 cm), *n*-hexane/2-propanol = 100/1, *v* = 1.0 mL·min^{-1}, λ = 254 nm, t (minor) = 20.17 min, t (major) = 23.74 min]; $[\alpha]_D^{21}$ = 0.03 (c = 1.0, $CHCl_3$). ^{1}H NMR (400 MHz, $CDCl_3$, minor diastereomer indicated by *) δ 2.18 (dd, *J* = 4.4, 11.6 Hz, 1H), 2.30 (dt, *J* = 7.2, 11.6 Hz, 1H), 3.47-3.53 (m, 2H), 3.97 (t, *J* = 7.2 Hz, 1H), 4.34 (br s, 1H), 5.05-5.09 (m, 1H), 5.19 (dd, *J* = 1.2, 10.4 Hz, 1H), 5.32 (s, 1H), 5.42 (s, 1H*), 6.30 (ddd, *J* = 8.4, 10.0, 16.8 Hz, 1H), 6.47 (d, *J* = 7.6 Hz, 1H), 6.53 (d, *J* = 8.0 Hz, 1H*), 6.72 (t, *J* = 7.6 Hz, 1H), 6.77-6.81 (m, 2H), 6.94 (d, *J* = 7.6 Hz, 1H), 7.06 (t, *J* = 7.6 Hz, 1H), 7.11-7.14 (m, 2H). ^{13}C NMR (100 MHz, $CDCl_3$) δ 39.3, 56.8, 61.7, 67.6, 96.6, 108.5, 117.5, 118.5, 124.7, 128.2, 128.6, 129.6, 129.9, 132.5, 137.5, 139.1, 150.5. IR (thin film): ν_{max} (cm^{-1}) = 3346, 2969, 2871, 1607, 1486, 1408, 1361, 1315, 1239, 1156, 1091, 1042, 1012, 954, 919, 825, 804, 743, 705, 656; HRMS (ESI) calcd for $C_{19}H_{19}ClNO$ $[M+H]^+$: 312.1150. Found: 312.1140.

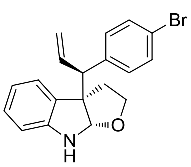

4af. Yellow oil, 70% yield, 94% ee [Daicel Chiralpak IC (0.46 cm x 25 cm),

n-hexane/2-propanol = 100/1, v = 1.0 mL·min^{-1}, λ = 254 nm, t (minor) = 21.82 min, t (major) = 26.31 min]; $[\alpha]_D^{25}$ = -0.13 (c = 1.0, $CHCl_3$). ^1H NMR (400 MHz, $CDCl_3$, minor diastereomer indicated by *) δ 2.19 (dd, *J* = 4.8, 12.0 Hz, 1H), 2.26-2.34 (m, 1H), 3.48-3.54 (m, 2H), 3.98 (t, *J* = 7.6 Hz, 1H), 4.32 (s, 1H), 5.07 (d, *J* = 17.2 Hz, 1H), 5.19 (d, *J* = 10.4 Hz, 1H), 5.32 (s, 1H), 5.43 (s, 1H*), 6.25-6.34 (m, 1H), 6.48 (d, *J* = 8.0 Hz, 1H), 6.70-6.75 (m, 3H), 6.95 (d, *J* = 7.6 Hz, 1H), 7.07 (t, *J* = 7.6 Hz, 1H), 7.26-7.29 (m, 2H). ^{13}C NMR (100 MHz, $CDCl_3$) δ 39.3, 56.9, 61.7, 67.6, 96.6, 108.5, 117.5, 118.5, 120.7, 124.7, 128.6, 129.6, 130.3, 131.1, 137.4, 139.6, 150.5. IR (thin film): ν_{max} (cm^{-1}) = 3347, 3051, 2924, 2858, 1607, 1483, 1405, 1314, 1239, 1109, 1072, 1042, 1008, 958, 918, 823, 802, 741, 656; HRMS (ESI) calcd for $C_{19}H_{19}BrNO$ $[M+H]^+$: 356.0645. Found: 356.0639.

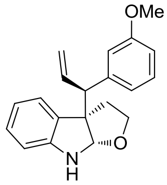

4ag. Yellow oil, 66% yield, 95% ee [Daicel Chiralpak IC (0.46 cm x 25 cm), *n*-hexane/2-propanol = 100/1, v = 1.0 mL·min^{-1}, λ = 254 nm, t (minor) = 39.92 min, t (major) = 57.94 min]; $[\alpha]_D^{23}$ = -20.4 (c = 1.0, $CHCl_3$). ^1H NMR (400 MHz, $CDCl_3$, minor diastereomer indicated by *) δ 2.21 (dd, *J* = 4.8, 12.0 Hz, 1H), 2.28-2.36 (m, 1H), 3.49-3.54 (m, 2H), 3.55 (s, 3H), 3.98 (t, *J* = 7.6 Hz, 1H), 4.32 (br s, 1H), 5.11 (d, *J* = 17.2 Hz, 1H), 5.18 (d, *J* = 10.0 Hz, 1H), 5.36 (s, 1H), 5.43 (s, 1H*), 6.29-6.38 (m, 2H), 6.48 (d, *J* = 8.0 Hz, 1H), 6.55 (d, *J* = 7.6 Hz, 1H), 6.71-6.75 (m, 2H), 7.00-7.11 (m, 3H). ^{13}C NMR (100 MHz, $CDCl_3$) δ 39.6, 54.8, 57.7, 61.8, 67.5, 96.6, 108.5, 112.9, 113.2, 117.1, 118.4, 121.1, 124.8, 128.4, 129.0, 130.1, 137.8, 142.1, 150.6, 159.0. IR (thin film): ν_{max} (cm^{-1}) = 3403, 3355, 2945, 2867, 1602, 1484, 1468, 1436, 1414, 1316, 1262, 1158, 1041, 1012, 917, 739, 699, 657; HRMS (ESI) calcd for $C_{20}H_{22}NO_2$ $[M+H]^+$: 308.1645. Found: 308.1639.

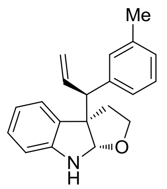

4ah. Yellow oil, 78% yield, 94% ee [Agilent 1260 Infinity Analytical SFC system Daicel Chiralpak IC (0.46 cm x 15 cm), CO_2/2-propanol = 90/10, v = 1.3 mL·min^{-1}, λ = 230 nm, t (minor) = 10.56 min, t (major) = 11.34 min]; $[\alpha]_D^{25}$ = -32.0 (c = 1.0, $CHCl_3$). ^{1}H NMR (400 MHz, $CDCl_3$, minor diastereomer indicated by *) δ 2.15-2.20 (m, 1H), 2.20 (s, 3H), 2.31 (dt, J = 6.8, 11.2 Hz, 1H), 3.47-3.53 (m, 2H), 3.96 (t, J = 7.6 Hz, 1H), 4.24 (br s, 1H), 5.08 (d, J = 16.8 Hz, 1H), 5.15 (d, J = 10.0 Hz, 1H), 5.37 (s, 1H), 5.41 (s, 1H*), 6.32 (ddd, J = 8.4, 10.0, 16.8 Hz, 1H), 6.47 (d, J = 7.6 Hz, 1H), 6.55 (d, J = 8.0 Hz, 1H*), 6.67-6.73 (m, 3H), 6.93 (d, J = 7.6 Hz, 1H), 6.98 (d, J = 7.6 Hz, 1H), 7.03-7.07 (m, 2H). ^{13}C NMR (100 MHz, $CDCl_3$) δ 21.4, 39.2, 57.5, 61.9, 67.6, 96.5, 108.4, 117.0, 118.5, 124.8, 125.5, 127.4, 127.9, 128.4, 129.5, 130.3, 137.5, 138.0, 140.5, 150.5. IR (thin film): ν_{max} (cm^{-1}) = 3406, 3353, 3030, 2970, 2867, 1606, 1469, 1412, 1360, 1314, 1242, 1154, 1041, 1013, 961, 915, 886, 782, 739, 702, 664; HRMS (ESI) calcd for $C_{20}H_{22}NO$ $[M+H]^+$: 292.1696. Found: 292.1697.

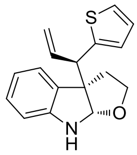

4ai. Yellow oil, 85% yield, 93% ee [Agilent 1260 Infinity Analytical SFC system Daicel Chiralpak AD-H (0.46 cm x 15 cm), CO_2/2-propanol = 85/15, v = 1.3 mL·min^{-1}, λ = 230 nm, t (minor) = 16.40 min, t (major) = 20.62 min]; $[\alpha]_D^{23}$ = -25.7 (c = 1.0, $CHCl_3$). ^{1}H NMR (400 MHz, $CDCl_3$, minor diastereomer indicated by *) δ 2.18 (dd, J = 4.8, 12.0 Hz, 1H), 2.30 (dt, J = 7.2, 11.6 Hz, 1H), 2.49 (dt, J = 7.2, 11.6 Hz, 1H*), 3.51-3.57 (m, 1H), 3.93-4.00 (m, 2H), 4.45 (s, 1H), 4.54 (s, 1H*), 5.07-5.18 (m, 2H), 5.47 (d, J = 1.6 Hz, 1H), 5.85-5.94 (m, 1H*), 6.18-6.27 (m, 1H), 6.51 (d, J = 8.0 Hz, 1H), 6.62 (d, J = 4.0 Hz, 1H), 6.67 (d, J = 6.8 Hz, 1H*), 6.71-6.75 (m, 1H), 6.84-6.87 (m, 1H), 6.90-6.92 (m, 1H*), 7.02 (d, J = 7.2 Hz, 1H), 7.07-7.11 (m, 2H). ^{13}C NMR (100 MHz, $CDCl_3$) δ 39.5, 52.9, 61.9, 67.4, 96.8, 108.4, 117.2, 118.6, 124.2,

124.9, 125.4, 126.2, 128.7, 129.7, 137.8, 142.9, 150.8. IR (thin film): ν_{max} (cm^{-1}) = 3350, 3077, 3051, 2944, 2867, 1607, 1469, 1437, 1358, 1313, 1237, 1153, 1108, 1040, 1012, 954, 918, 739, 696; HRMS (ESI) calcd for $C_{17}H_{18}NOS$ $[M+H]^+$: 284.1104. Found: 284.1094.

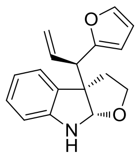

4aj. Yellow oil, 54% yield, 95% ee [Daicel Chiralpak IC (0.46 cm x 25 cm), *n*-hexane/2-propanol = 100/1, v = 1.0 mL·min^{-1}, λ = 254 nm, t (minor) = 22.80 min, t (major) = 55.77 min]; $[\alpha]_D^{25}$ = -8.1 (c = 1.0, $CHCl_3$). ^1H NMR (400 MHz, $CDCl_3$, minor diastereomer indicated by *) δ 2.11 (dd, *J* = 4.8, 12.4 Hz, 1H), 2.19 (dd, *J* = 4.8, 12.4 Hz, 1H*), 2.30 (dt, *J* = 7.2, 12.0 Hz, 1H), 2.52 (dt, *J* = 7.2, 12.0 Hz, 1H*), 3.48-3.55 (m, 1H), 3.74 (d, *J* = 9.6 Hz, 1H*), 3.79 (d, *J* = 8.0 Hz, 1H), 3.90-3.94 (m, 1H), 3.97-4.01 (m, 1H*), 4.55 (s, 1H), 4.99-5.04 (m, 1H), 5.12-5.15 (m, 1H), 5.48 (s, 1H*), 5.61 (s, 1H), 5.88 (dd, *J* = 0.4, 3.2 Hz, 1H), 6.05 (ddd, *J* = 8.0, 10.4, 17.2 Hz, 1H), 6.27-6.28 (m, 1H), 6.31-6.33 (m, 1H*), 6.51-6.53 (m, 1H*), 6.54-6.57 (m, 1H), 6.61-6.52 (m, 1H*), 6.67-6.71 (m, 1H), 6.80-6.82 (m, 1H), 7.04-7.08 (m, 1H), 7.34-7.40 (m, 1H). ^{13}C NMR (100 MHz, $CDCl_3$) δ 38.1, 49.6, 61.3, 67.3, 96.1, 107.3, 108.3, 110.2, 117.9, 118.6, 124.2, 128.4, 130.7, 135.2, 141.2, 150.0, 154.0. IR (thin film): ν_{max} (cm^{-1}) = 3406, 3351, 2968, 2869, 1607, 1483, 1412, 1360, 1315, 1259, 1150, 1069, 1012, 957, 919, 799, 737, 658; HRMS (ESI) calcd for $C_{17}H_{18}NO_2$ $[M+H]^+$: 268.1332. Found: 268.1332.

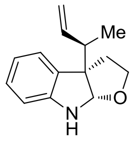

4ak. Yellow oil, 46% yield, 93% ee [Agilent 1260 Infinity Analytical SFC system Daicel Chiralcel OJ-H (0.46 cm x 15 cm), CO_2/2-propanol = 90/10, v = 1.3 mL·min^{-1}, λ = 214 nm, t (minor) = 8.61 min, t (major) = 10.69 min]; $[\alpha]_D^{25}$ = -45.0 (c

= 1.0, $CHCl_3$). ^{1}H NMR (400 MHz, $CDCl_3$, minor diastereomer indicated by *) δ 0.92 (d, *J* = 6.4 Hz, 3H), 1.27 (d, *J* = 6.4 Hz, 3H*), 2.07 (dd, *J* = 4.4, 11.6 Hz, 1H), 2.18 (dt, *J* = 7.2, 11.6 Hz, 1H), 2.49 (t, *J* = 6.8 Hz, 1H), 3.04 (t, *J* = 6.8 Hz, 1H*), 3.47-3.53 (m, 1H), 3.88 (t, *J* = 6.8 Hz, 1H*), 3.96 (t, *J* = 7.6 Hz, 1H), 4.56 (s, 1H), 5.05-5.10 (m, 2H), 5.33 (s, 1H), 5.72-5.81 (m, 1H*), 5.94-6.03 (m, 1H), 6.56-6.58 (m, 1H), 6.72 (t, *J* = 7.2 Hz, 1H), 7.03-7.09 (m, 2H). ^{13}C NMR (100 MHz, $CDCl_3$) δ 15.2, 39.2, 44.9, 61.3, 67.6, 97.6, 108.1, 115.3, 118.4, 124.6, 128.2, 140.0, 140.4, 150.4. IR (thin film): ν_{max} (cm^{-1}) = 3406, 3344, 2967, 2933, 2866, 1637, 1607, 1466, 1416, 1360, 1313, 1251, 1222, 1070, 1035, 1001, 952, 914, 741, 659; HRMS (ESI) calcd for $C_{14}H_{18}NO$ $[M+H]^+$: 216.1383. Found: 216.1385.

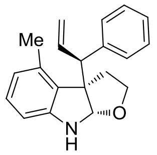

4ba. White solid, 50% yield, 97% ee [Daicel Chiralpak IC (0.46 cm x 25 cm), *n*-hexane/2-propanol = 90/10, *v* = 1.0 mL·min^{-1}, λ = 254 nm, t (minor) = 7.59 min, t (major) = 14.46 min]; $[\alpha]_D^{24}$ = -61.2 (c = 1.0, $CHCl_3$). ^{1}H NMR (400 MHz, $CDCl_3$, minor diastereomer indicated by *) δ 2.10-2.24 (m, 2H), 2.34 (s, 3H*), 2.37 (s, 3H), 3.46-3.52 (m, 1H), 3.60-3.65 (m, 1H), 4.04 (d, *J* = 8.0 Hz, 1H), 4.48 (br s, 1H), 4.85 (d, *J* = 16.8 Hz, 1H), 4.93 (d, *J* = 10.4 Hz, 1H), 5.68 (s, 1H), 5.76 (s, 1H*), 6.00 (ddd, *J* = 8.0, 10.0, 16.8 Hz, 1H), 6.43 (d, *J* = 7.6 Hz, 1H), 6.56 (d, *J* = 7.6 Hz, 1H), 6.99 (t, *J* = 7.6 Hz, 1H), 7.23-7.34 (m, 5H). ^{13}C NMR (100 MHz, $CDCl_3$) δ 18.7, 35.3, 53.9, 62.2, 67.0, 94.6, 106.2, 116.7, 121.7, 126.9, 128.2, 128.4, 128.9, 129.6, 133.9, 137.9, 140.5, 150.1. IR (thin film): ν_{max} (cm^{-1}) = 3354, 3057, 2977, 2922, 2868, 1590, 1491, 1464, 1365, 1313, 1260, 1159, 1061, 1022, 998, 968, 919, 742, 700, 677, 655; HRMS (ESI) calcd for $C_{20}H_{22}NO$ $[M+H]^+$: 292.1696. Found: 292.1694. mp = 81.0-84.6 ^oC.

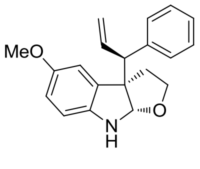

4ca. Yellow oil, 74% yield, 91% ee [Agilent 1260 Infinity Analytical SFC system Daicel Chiralpak AD-H (0.46 cm x 15 cm), CO_2/2-propanol = 85/15, *v* = 1.3 mL·min^{-1}, λ = 230 nm, t (minor) = 10.68 min, t (major) = 14.90 min]; $[\alpha]_D^{21}$ = +28.6 (c = 1.0, $CHCl_3$). ^{1}H NMR (400 MHz, $CDCl_3$, minor diastereomer indicated by *) δ 2.16 (dd, *J* = 4.4, 12.0 Hz, 1H), 2.34 (dt, *J* = 7.6, 12.0 Hz, 1H), 2.44 (dt, *J* = 7.6, 12.0 Hz, 1H*), 3.42-3.54 (m, 2H), 3.65 (s, 3H*), 3.68 (s, 3H), 3.97 (t, *J* = 7.2 Hz, 1H), 4.10 (br s, 1H), 5.10 (d, *J* = 17.2 Hz, 1H), 5.18 (d, *J* = 10.4 Hz, 1H), 5.38 (s, 1H), 5.42 (s, 1H*), 6.34 (ddd, *J* = 8.8, 10.4, 17.2 Hz, 1H), 6.43-6.46 (m, 2H), 6.49-6.51 (m, 2H*), 6.65 (dd, *J* = 2.4, 8.4 Hz, 1H), 6.91-6.93 (m, 2H), 7.17-7.19 (m, 3H). ^{13}C NMR (100 MHz, $CDCl_3$) δ 38.8, 55.8, 57.2, 62.3, 67.6, 97.5, 109.5, 111.4, 113.6, 117.3, 126.8, 128.1, 128.7, 132.0, 137.7, 140.6, 144.4, 153.2. IR (thin film): ν_{max} (cm^{-1}) = 3359, 3061, 3029, 2943, 2867, 1633, 1598, 1489, 1437, 1259, 1209, 1182, 1153, 1035, 1012, 959, 917, 805, 741, 702, 649; HRMS (ESI) calcd for $C_{20}H_{22}NO_2$ [M+H]$^+$: 308.1645. Found: 308.1632.

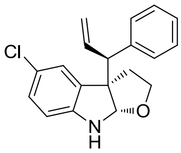

4da. Yellow oil, 83% yield, 96% ee [Daicel Chiralpak IC (0.46 cm x 25 cm), *n*-hexane/2-propanol = 100/1, *v* = 1.0 mL·min^{-1}, λ = 254 nm, t (minor) = 14.65 min, t (major) = 18.74 min]; $[\alpha]_D^{20}$ = +36.4 (c = 1.0, $CHCl_3$). ^{1}H NMR (400 MHz, $CDCl_3$, minor diastereomer indicated by *) δ 2.14 (dd, *J* = 4.4, 12.0 Hz, 1H), 2.28-2.36 (m, 1H), 2.39-2.47 (m, 1H*), 3.47-3.53 (m, 2H), 3.66 (d, *J* = 10.0 Hz, 1H*), 3.97 (t, *J* = 7.6 Hz, 1H), 4.35 (br s, 1H), 5.10 (d, *J* = 17.2 Hz, 1H), 5.20 (d, *J* = 10.0 Hz, 1H), 5.39 (s, 1H), 5.45 (s, 1H*), 6.31 (ddd, *J* = 8.8, 10.4, 17.2 Hz, 1H), 6.38 (d, *J* = 8.0 Hz, 1H), 6.44 (d, *J* = 8.0 Hz, 1H*), 6.84 (d, *J* = 2.0 Hz, 1H), 6.90-6.92 (m, 2H), 7.01 (dd, *J* = 2.0, 8.4 Hz, 1H), 7.19-7.20 (m, 3H). ^{13}C NMR (100 MHz, $CDCl_3$) δ 39.1, 57.2, 62.1, 67.6, 96.9, 109.1, 117.7, 123.0, 124.9, 127.0, 128.2, 128.3, 128.6, 132.2, 137.2, 140.2, 149.0. IR (thin film): ν_{max} (cm^{-1}) = 3418, 3344, 2972, 2869, 1633, 1604, 1479, 1448, 1360, 1334, 1259, 1223, 1165, 1110, 1066, 1011, 959, 919, 888, 809, 736, 701, 666,

620; HRMS (ESI) calcd for $C_{19}H_{19}ClNO$ $[M+H]^+$: 312.1150. Found: 312.1150.

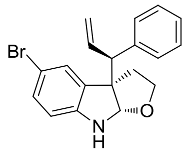

4ea. Yellow oil, 81% yield, 96% ee [Daicel Chiralpak IC (0.46 cm x 25 cm), *n*-hexane/2-propanol = 100/1, v = 1.0 mL·min^{-1}, λ = 254 nm, t (minor) = 15.11 min, t (major) = 18.77 min]; $[\alpha]_D^{20}$ = +48.7 (c = 1.0, $CHCl_3$). ^{1}H NMR (400 MHz, $CDCl_3$, minor diastereomer indicated by *) δ 2.13 (dd, *J* = 4.4, 12.0 Hz, 1H), 2.31 (dd, *J* = 5.6, 10.4 Hz, 1H), 3.46-3.53 (m, 2H), 3.65 (d, *J* = 10.0 Hz, 1H*), 3.97 (t, *J* = 7.6 Hz, 1H), 4.37 (s, 1H), 5.10 (d, *J* = 16.8 Hz, 1H), 5.20 (d, *J* = 10.0 Hz, 1H), 5.37 (s, 1H), 5.43 (s, 1H*), 6.26-6.35 (m, 2H), 6.40 (d, *J* = 8.4 Hz, 1H*), 6.90-6.92 (m, 2H), 6.96 (d, *J* = 2.0 Hz, 1H), 7.13-7.20 (m, 4H). ^{13}C NMR (100 MHz, $CDCl_3$) δ 39.0, 57.1, 62.0, 67.6, 96.7, 109.6, 109.9, 117.7, 127.0, 127.7, 128.2, 128.6, 131.1, 132.7, 137.2, 140.1, 149.5. IR (thin film): ν_{max} (cm^{-1}) = 3417, 3342, 3061, 2972, 2869, 1602, 1476, 1448, 1434, 1360, 1334, 1259, 1223, 1163, 1124, 1065, 1035, 1011, 959, 919, 885, 808, 750, 735, 701, 668; HRMS (ESI) calcd for $C_{19}H_{19}BrNO$ $[M+H]^+$: 356.0645. Found: 356.0640.

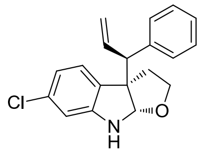

4fa. Yellow oil, 81% yield, 95% ee [Agilent 1260 Infinity Analytical SFC system Daicel Chiralpak IC (0.46 cm x 15 cm), CO_2/2-propanol = 90/10, v = 1.3 mL·min^{-1}, λ = 214 nm, t (minor) = 11.54 min, t (major) = 14.19 min]; $[\alpha]_D^{23}$ = -5.0 (c = 1.0, $CHCl_3$). ^{1}H NMR (400 MHz, $CDCl_3$, minor diastereomer indicated by *) δ 2.12 (dd, *J* = 4.4, 11.6 Hz, 1H), 2.33 (dt, *J* = 7.2, 12.0 Hz, 1H), 3.46-3.52 (m, 2H), 3.97 (t, *J* = 7.6 Hz, 1H), 4.42 (br s, 1H), 5.09 (d, *J* = 16.8 Hz, 1H), 5.19 (d, *J* = 10.4 Hz, 1H), 5.39 (s, 1H), 5.44 (s, 1H*), 6.01 (ddd, *J* = 8.4, 10.0, 16.8 Hz, 1H*), 6.30 (ddd, *J* = 8.4, 10.0, 16.8 Hz, 1H), 6.44 (d, *J* = 1.6 Hz, 1H), 6.64 (dd, *J* = 1.6, 7.6 Hz, 1H), 6.74 (d, *J* = 7.6

Hz, 1H), 6.90-6.92 (m, 2H), 7.18-7.20 (m, 3H). ^{13}C NMR (100 MHz, $CDCl_3$) δ 39.0, 57.1, 61.4, 67.7, 96.8, 108.3, 117.6, 118.2, 125.5, 126.9, 128.2, 128.6, 128.8, 134.0, 137.4, 140.3, 151.4. IR (thin film): ν_{max} (cm^{-1}) = 3418, 3342, 2971, 2868, 1603, 1483, 1452, 1416, 1359, 1313, 1242, 1111, 1069, 1036, 958, 909, 839, 797, 734, 701, 669; HRMS (ESI) calcd for $C_{19}H_{19}ClNO$ $[M+H]^+$: 312.1150. Found: 312.1150.

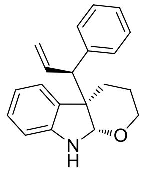

4ga. Yellow oil, 53% yield, 91% ee [Agilent 1260 Infinity Analytical SFC system Daicel Chiralcel OJ-H (0.46 cm x 15 cm), CO_2/2-propanol = 85/15, v = 1.3 mL·min^{-1}, λ = 230 nm, t (major) = 7.66 min, t (minor) = 13.15 min]; $[\alpha]_D^{25}$ = -20.7 (c = 1.0, $CHCl_3$). ^{1}H NMR (400 MHz, $CDCl_3$, minor diastereomer indicated by *) δ 1.32-1.41 (m, 1H), 1.52-1.58 (m, 1H), 1.94-2.02 (m, 1H), 2.09-2.14 (m, 1H), 3.34 (d, J = 10.0 Hz, 1H), 3.37-3.43 (m, 1H), 3.60-3.65 (m, 1H), 4.21 (s, 1H), 4.34 (s, 1H*), 5.06 (s, 1H), 5.10-5.21 (m, 2H), 6.05 (ddd, J = 10.0, 10.0, 16.8 Hz, 1H*), 6.32 (ddd, J = 10.0, 10.0, 16.8 Hz, 1H), 6.50-6.52 (m, 1H), 6.61-6.67 (m, 2H), 6.72-6.75 (m, 2H), 7.08 (dt, J = 1.2, 7.6 Hz, 1H), 7.11-7.17 (m, 3H). ^{13}C NMR (100 MHz, $CDCl_3$) δ 21.0, 25.0, 50.9, 57.4, 61.0, 91.8, 109.3, 117.7, 118.3, 124.6, 126.4, 127.5, 128.0, 129.2, 130.2, 137.5, 140.2, 149.7. IR (thin film): ν_{max} (cm^{-1}) = 3334, 3078, 2918, 1605, 1484, 1464, 1411, 1321, 1235, 1205, 1068, 1012, 945, 914, 745, 713, 638; HRMS (ESI) calcd for $C_{20}H_{22}NO$ $[M+H]^+$: 292.1696. Found: 292.1693.

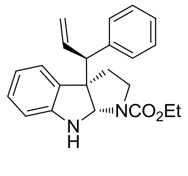

4ha. Yellow oil, 69% yield, 97% ee [Daicel Chiralpak IC (0.46 cm x 25 cm), n-hexane/2-propanol = 80/20, v = 0.4 mL·min^{-1}, λ = 254 nm, t (minor) = 14.37 min, t (major) = 40.95 min]; $[\alpha]_D^{22}$ = -563.5 (c = 1.0, $CHCl_3$). ^{1}H NMR (400 MHz, d_6-DMSO) δ 1.25 (t, J = 6.4 Hz, 3H), 2.13 (dd, J = 6.0, 12.4 Hz, 1H), 2.27-2.35 (m,

1H), 2.84-2.91 (m, 1H), 3.57 (t, J = 9.6 Hz, 1H), 3.63 (d, J = 8.4 Hz, 1H), 4.11 (q, J = 6.8 Hz, 2H), 5.06 (d, J = 16.8 Hz, 1H), 5.14 (d, J = 10.4 Hz, 1H), 5.32 (s, 1H), 5.87 (br s, 1H), 6.30-6.39 (m, 1H), 6.51 (d, J = 7.6 Hz, 1H), 6.60 (t, J = 7.2 Hz, 1H), 6.82 (d, J = 7.2 Hz, 1H), 7.00-7.03 (m, 3H), 7.22-7.28 (m, 3H). ^{13}C NMR (100 MHz, d_6-DMSO) δ 14.1, 34.3, 44.6, 56.0, 59.9, 73.1, 78.0, 108.0, 116.4, 116.6, 123.5, 126.0, 127.3, 127.7, 128.3, 129.4, 137.8, 140.3, 150.1, 153.3. IR (thin film): ν_{max} (cm^{-1}) = 3365, 2976, 2879, 1684, 1606, 1467, 1418, 1380, 1347, 1319, 1234, 1198, 1170, 1110, 1049, 920, 892, 744, 701, 673; HRMS (ESI) calcd for $C_{22}H_{25}N_2O_2$ [M+H]$^+$: 349.1911. Found: 349.1907.

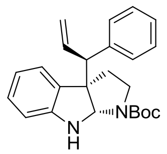

4ia. Yellow oil, 58% yield, 98% ee [Daicel Chiralpak AD-H (0.46 cm x 25 cm), *n*-hexane/2-propanol = 100/1, v = 1.0 mL·min^{-1}, λ = 254 nm, t (major) = 22.66 min, t (minor) = 48.68 min]; $[\alpha]_D^{25}$ = -242.0 (c = 1.0, $CHCl_3$). ^{1}H NMR (400 MHz, $CDCl_3$, rotamer) δ 1.42 (s, 6H), 1.50 (s, 3H), 2.13-2.19 (m, 1H), 2.24-2.30 (m, 1H), 2.91-2.98 (m, 1H), 3.46 (d, J = 9.2 Hz, 1H), 3.50-3.69 (m, 1H), 4.45 and 4.89 (s, 1H), 5.09-5.26 (m, 3H), 6.26-6.35 (m, 1H), 6.51 (d, J = 8.0 Hz, 1H), 6.61-6.65 (m, 2H), 6.90-6.92 (m, 2H), 7.03-7.07 (m, 1H), 7.16-7.20 (m, 3H). ^{13}C NMR (150 MHz, $CDCl_3$, rotamer) δ 28.4, 28.6, 29.7, 33.6, 33.7, 45.4, 45.8, 56.6, 56.8, 60.4, 61.5, 78.9, 79.7, 80.1, 109.1, 109.3, 117.5, 117.6, 118.0, 118.5, 124.4, 124.5, 126.7, 126.8, 127.86, 127.95, 127.98, 128.5, 128.6, 128.7, 128.8, 129.2, 129.9, 130.0, 137.3, 137.4, 140.4, 149.7, 150.1, 153.4, 154.3. IR (thin film): ν_{max} (cm^{-1}) = 3402, 3030, 2975, 2880, 1684, 1607, 1480, 1398, 1367, 1315, 1252, 1205, 1163, 1112, 1051, 994, 920, 891, 746, 704, 673; HRMS (ESI) calcd for $C_{24}H_{29}N_2O_2$ [M+H]$^+$: 377.2224. Found: 377.2215.

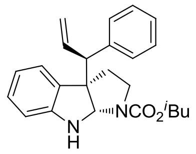

4ja. Yellow oil, 58% yield, 96% ee [Phenomenex Lu x 5u Cellulose-4 (0.46 cm x 25 cm), *n*-hexane/2-propanol = 100/1, *v* = 1.0 mL·min^{-1}, λ = 254 nm, t (minor) = 22.27 min, t (major) = 43.56 min]; $[\alpha]_D^{25}$ = -109.6 (c = 1.0, $CHCl_3$). ^1H NMR (600 MHz, d_6-DMSO) δ 0.94-0.95 (m, 6H), 1.94 (br s, 1H), 2.13 (dd, *J* = 6.0, 12.6 Hz, 1H), 2.29-2.34 (m, 1H), 2.87-2.88 (m, 1H), 3.58 (t, *J* = 9.6 Hz, 1H), 3.63 (d, *J* = 8.4 Hz, 1H), 3.83 (br s, 2H), 5.06 (d, *J* = 16.8 Hz, 1H), 5.14 (dd, *J* = 1.2, 10.2 Hz, 1H), 5.34 (s, 1H), 5.83 (br s, 1H), 6.34 (ddd, *J* = 9.0, 10.2, 17.4 Hz, 1H), 6.51 (d, *J* = 8.4 Hz, 1H), 6.60 (t, *J* = 7.2 Hz, 1H), 6.82 (d, *J* = 7.2 Hz, 1H), 7.00-7.02 (m, 3H), 7.20-7.23 (m, 3H). ^{13}C NMR (150 MHz, d_6-DMSO) δ 18.40, 18.43, 27.1, 34.4, 44.6, 56.0, 60.0, 70.1, 78.1, 108.1, 116.5, 116.7, 123.5, 126.1, 127.4, 127.7, 128.3, 129.5, 137.8, 140.3, 150.1, 153.4. IR (thin film): ν_{max} (cm^{-1}) = 3360, 2961, 2875, 1687, 1606, 1468, 1418, 1384, 1359, 1319, 1260, 1198, 1109, 1050, 989, 918, 886, 816, 744, 701, 674; HRMS (ESI) calcd for $C_{24}H_{29}N_2O_2$ $[M+H]^+$: 377.2224. Found: 377.2223.

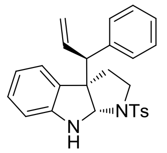

4ka. Yellow oil, 67% yield, 99% ee [Daicel Chiralpak AD-H (0.46 cm x 15 cm) and Chiralpak IC (0.46 cm x 25 cm), *n*-hexane/2-propanol/Et_2NH = 80/20/0.1, *v* = 0.7 mL·min^{-1}, λ = 230 nm, t (minor) = 43.60 min, t (major) = 46.26 min]; $[\alpha]_D^{25}$ = -157.5 (c = 1.0, $CHCl_3$). ^1H NMR (400 MHz, $CDCl_3$, minor diastereomer indicated by *) δ 1.96-2.10 (m, 2H), 2.36 (s, 3H*), 2.45 (s, 3H), 3.03 (dt, *J* = 6.0, 10.8 Hz, 1H), 3.30 (d, *J* = 9.2 Hz, 1H), 3.41 (t, *J* = 9.2 Hz, 1H), 4.58 (s, 1H), 5.01 (d, *J* = 17.2 Hz, 1H), 5.06 (d, *J* = 10.4 Hz, 1H), 5.26 (s, 1H), 6.03-6.12 (m, 1H), 6.52 (d, *J* = 8.0 Hz, 1H), 6.58 (d, *J* = 7.2 Hz, 1H), 6.64 (t, *J* = 7.6 Hz, 1H), 6.80 (d, *J* = 7.2 Hz, 2H), 7.06 (t, *J* = 7.6 Hz, 1H), 7.13-7.19 (m, 3H), 7.30 (d, *J* = 8.0 Hz, 2H), 7.69 (d, *J* = 8.0 Hz, 2H). ^{13}C NMR (100 MHz, $CDCl_3$) δ 21.5, 34.8, 47.5, 56.6, 62.0, 81.7, 109.6, 117.8, 118.7, 124.3, 126.9, 127.1, 128.1, 128.6, 128.8, 129.4, 129.7, 136.3, 136.9, 140.0, 143.4, 149.5. IR (thin film): ν_{max} (cm^{-1}) = 3387, 2967, 2917, 1714, 1600, 1486, 1462, 1400, 1337, 1251, 1155, 1112, 1059, 920, 884, 779, 754, 700, 660; HRMS (ESI) calcd for $C_{26}H_{27}N_2O_2S$

$[M+H]^+$: 431.1788. Found: 431.1782.

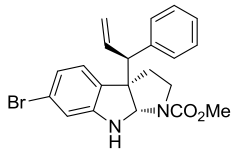

4la. Yellow oil, 75% yield, 98% ee [Daicel Chiralpak AD-H (0.46 cm x 25 cm), *n*-hexane/2-propanol = 100/1, *v* = 1.0 mL·min^{-1}, λ = 254 nm, t (minor) = 39.25 min, t (major) = 44.19 min]; $[\alpha]_D^{23}$ = -530.0 (c = 1.0, $CHCl_3$). ^1H NMR (400 MHz, d_6-DMSO) δ 2.07 (dd, *J* = 5.6, 12.4 Hz, 1H), 2.23-2.31 (m, 1H), 2.81-2.88 (m, 1H), 3.51-3.62 (m, 2H), 3.62 (s, 3H), 5.03 (d, *J* = 16.8 Hz, 1H), 5.11 (d, *J* = 10.0 Hz, 1H), 5.28 (s, 1H), 6.24 (br s, 1H), 6.24-6.33 (m, 1H), 6.60 (s, 1H), 6.67 (s, 2H), 6.98-7.00 (m, 2H), 7.20-7.21 (m, 3H). ^{13}C NMR (100 MHz, d_6-DMSO) δ 34.2, 44.7, 51.5, 55.7, 59.8, 78.2, 110.4, 116.8, 118.9, 120.8, 125.1, 126.2, 127.5, 128.2, 129.0, 137.3, 140.0, 151.9, 153.6. IR (thin film): ν_{max} (cm^{-1}) = 3352, 2951, 2875, 1681, 1601, 1452, 1394, 1314, 1230, 1199, 1110, 1051, 989, 954, 920, 850, 777, 752, 724, 700, 625; HRMS (ESI) calcd for $C_{21}H_{22}BrN_2O_2$ $[M+H]^+$: 413.0859. Found: 413.0857.

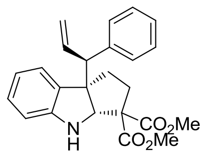

4na. Yellow oil, 48% yield, 99% ee [Agilent 1260 Infinity Analytical SFC system Daicel Chiralpak IC (0.46 cm x 15 cm), CO_2/2-propanol = 95/5, *v* = 1.3 mL·min^{-1}, λ = 230 nm, t (major) = 20.66 min, t (minor) = 23.09 min]; $[\alpha]_D^{25}$ = -74.2 (c = 1.0, $CHCl_3$). ^1H NMR (400 MHz, $CDCl_3$) δ 1.87-1.96 (m, 1H), 2.12-2.28 (m, 3H), 3.37 (br s, 1H), 3.65 (s, 3H), 3.67 (d, *J* = 9.6 Hz, 1H), 3.79 (s, 3H), 4.58 (s, 1H), 5.08 (d, *J* = 17.2 Hz, 1H), 5.14 (d, *J* = 10.4 Hz, 1H), 6.31-6.40 (m, 2H), 6.74-6.81 (m, 3H), 7.01-7.05 (m, 1H), 7.10-7.11 (m, 3H), 7.18 (d, *J* = 7.6 Hz, 1H). ^{13}C NMR (100 MHz, $CDCl_3$) δ 32.0, 38.0, 52.2, 52.7, 59.7, 62.3, 67.8, 69.6, 109.8, 116.5, 118.8, 124.6, 126.4, 127.9, 128.3, 128.9, 132.0, 138.2, 141.3, 151.7, 169.4, 171.9. IR (thin film): ν_{max} (cm^{-1}) = 3387, 2953, 2870, 1726, 1602, 1485, 1453, 1434, 1330, 1261, 1234,

1190, 1152, 1101, 1063, 1025, 993, 918, 742, 702; HRMS (ESI) calcd for $C_{24}H_{26}NO_4$ $[M+H]^+$: 392.1856. Found: 392.1854.

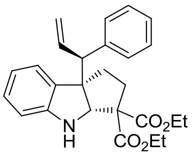

4oa. Yellow oil, 67% yield, 98% ee [Agilent 1260 Infinity Analytical SFC system Daicel Chiralpak IC (0.46 cm x 15 cm), CO_2/2-propanol = 95/5, v = 1.3 mL·min^{-1}, λ = 230 nm, t (major) = 21.84 min, t (minor) = 26.37 min]; $[\alpha]_D^{25}$ = -72.4 (c = 1.0, $CHCl_3$). ^{1}H NMR (400 MHz, $CDCl_3$, minor diastereomer indicated by *) δ 1.19 (t, J = 7.2 Hz, 3H), 1.29 (t, J = 6.8 Hz, 3H), 1.87-1.95 (m, 1H), 2.11-2.27 (m, 3H), 3.39 (br s, 1H), 3.67 (d, J = 8.8 Hz, 1H), 4.03-4.11 (m, 1H), 4.16-4.32 (m, 3H), 4.58 (s, 1H), 4.80 (s, 1H*), 5.08 (d, J = 16.8 Hz, 1H), 5.14 (d, J = 10.0 Hz, 1H), 6.32-6.41 (m, 2H), 6.73-6.77 (m, 1H), 6.79-6.81 (m, 2H), 7.00-7.04 (m, 1H), 7.09-7.13 (m, 3H), 7.18 (d, J = 8.4 Hz, 1H). ^{13}C NMR (100 MHz, $CDCl_3$) δ 14.09, 14.12, 32.0, 37.9, 59.7, 61.0, 61.4, 62.3, 67.8, 69.4, 109.6, 116.4, 118.6, 124.5, 126.3, 127.9, 128.3, 128.9, 131.9, 138.3, 141.4, 151.8, 169.0, 171.6. IR (thin film): ν_{max} (cm^{-1}) = 3390, 2979, 1724, 1603, 1484, 1466, 1367, 1257, 1231, 1183, 1152, 1098, 1058, 1024, 917, 857, 803, 746, 702; HRMS (ESI) calcd for $C_{26}H_{30}NO_4$ $[M+H]^+$: 420.2169. Found: 420.2167.

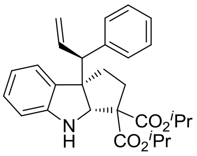

4pa. Yellow oil, 54% yield, 97% ee [Agilent 1260 Infinity Analytical SFC system Daicel Chiralpak IC (0.46 cm x 15 cm), CO_2/2-propanol = 95/5, v = 1.3 mL·min^{-1}, λ = 230 nm, t (major) = 14.24 min, t (minor) = 17.33 min]; $[\alpha]_D^{25}$ = -81.8 (c = 1.0, $CHCl_3$). ^{1}H NMR (400 MHz, $CDCl_3$) δ 1.17 (d, J = 6.4 Hz, 3H), 1.19 (d, J = 6.4 Hz, 3H), 1.26 (d, J = 4.8 Hz, 3H), 1.28 (d, J = 5.2 Hz, 3H), 1.85-1.94 (m, 1H), 2.08-2.26 (m, 3H), 3.37 (br s, 1H), 3.67 (d, J = 8.8 Hz, 1H), 4.57 (s, 1H), 4.99-5.14

(m, 4H), 6.32-6.41 (m, 2H), 6.74 (t, *J* = 7.2 Hz, 1H), 6.80-6.81 (m, 2H), 7.01 (t, *J* = 8.0 Hz, 1H), 7.09-7.12 (m, 3H), 7.17 (d, *J* = 7.2 Hz, 1H). ^{13}C NMR (100 MHz, $CDCl_3$) δ 21.5, 21.6, 21.7, 32.1, 37.8, 59.7, 62.3, 67.8, 68.4, 68.8, 69.2, 109.5, 116.3, 118.5, 124.5, 126.3, 127.9, 128.3, 128.9, 131.9, 138.4, 141.4, 151.8, 168.4, 171.1. IR (thin film): ν_{max} (cm^{-1}) = 3400, 2980, 1719, 1603, 1485, 1464, 1374, 1260, 1234, 1183, 1156, 1095, 1063, 997, 980, 913, 847, 808, 739, 702; HRMS (ESI) calcd for $C_{28}H_{34}NO_4$ $[M+H]^+$: 448.2482. Found: 448.2484.

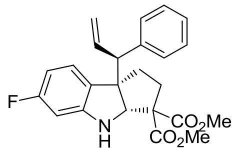

4qa. Yellow oil, 42% yield, 99% ee [Agilent 1260 Infinity Analytical SFC system Daicel Chiralpak IC (0.46 cm x 15 cm), CO_2/2-propanol = 95/5, *v* = 1.3 mL·min^{-1}, λ = 230 nm, t (major) = 10.15 min, t (minor) = 12.56 min]; $[\alpha]_D^{24}$ = -75.8 (c = 1.0, $CHCl_3$). ^{1}H NMR (400 MHz, $CDCl_3$) δ 1.86-1.94 (m, 1H), 2.13-2.27 (m, 3H), 3.53 (br s, 1H), 3.64 (s, 3H), 3.64-3.66 (m, 1H), 3.78 (s, 3H), 4.61 (s, 1H), 5.08 (d, *J* = 16.8 Hz, 1H), 5.15 (d, *J* = 10.0 Hz, 1H), 6.04 (dd, *J* = 2.0, 9.6 Hz, 1H), 6.31 (ddd, *J* = 8.8, 10.4, 17.2 Hz, 1H), 6.40-6.45 (m, 1H), 6.81-6.83 (m, 2H), 7.04-7.08 (m, 1H), 7.12-7.14 (m, 3H). ^{13}C NMR (100 MHz, $CDCl_3$) δ 31.9, 37.9, 52.3, 52.8, 59.5, 61.6, 67.6, 70.3, 96.9 (*J* = 25.8 Hz), 104.9 (*J* = 22.8 Hz), 116.8, 125.0 (*J* = 10.6 Hz), 126.5, 127.3, 128.0, 128.9, 137.8, 141.1, 153.0 (*J* = 11.4 Hz), 162.5, 164.9, 170.5 (*J* = 243.6 Hz). ^{19}F NMR (376 MHz, $CDCl_3$) δ -114.96 (m). IR (thin film): ν_{max} (cm^{-1}) = 3391, 2958, 2321, 1727, 1610, 1493, 1453, 1435, 1329, 1236, 1194, 1146, 1101, 1072, 995, 919, 741, 704; HRMS (ESI) calcd for $C_{24}H_{25}FNO_4$ $[M+H]^+$: 410.1762. Found: 410.1761.

Procedure for the synthesis of 4ma

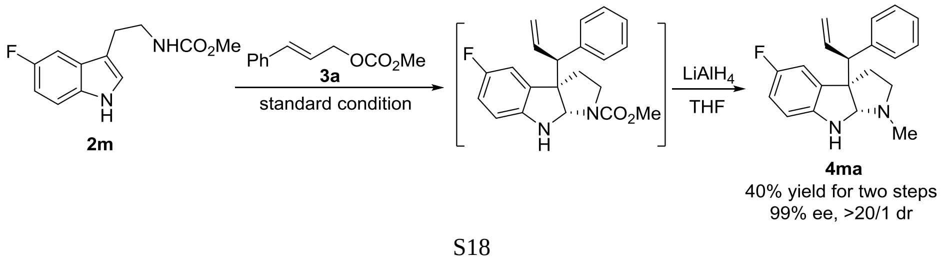

Following the general procedure for Ir-catalyzed intermolecular allylic alkylation of indole derivatives, the reaction of **2m** (239.1 mg, 1.2 mmol) and **3a** (582.0 mg, 2.4 mmol) was conducted under the standard conditions. Upon completion of the reaction, the mixture was concentrated under reduced pressure. The residue was subjected to a short column chromatography (PE/EA = 10/1) to afford the crude product (177.8 mg, 41% yield).

A flame-dried 25mL round bottom flask was cooled to room temperature and filled with argon. To this flask was added $LiAlH_4$ (47.9 mg, 1.26 mmol), the atmosphere was exchanged with argon three times before the addition of THF (10 mL) and the slow addition of the solution of the crude product obtained above (147.8 mg, 0.42 mmol) in THF (5 mL). After the reaction was complete (monitored by TLC), the reaction mixture was quenched with water, extracted with ether. The combined organic layers were washed with brine, dried over Na_2SO_4, and filtrated. The solvent was removed under reduced pressure, the residue was purified by silica gel column chromatography (EA/Et_3N = 100/0.1) to afford the desired product **4ma**. Colorless liquid, 126.7 mg, 98% yield, 99% ee [Daicel Chiralcel OD-H (0.46 cm x 25 cm), *n*-hexane/2-propanol = 98/2, v = 0.5 mL·min^{-1}, λ = 214 nm, t (minor) = 17.91 min, t (major) = 22.19 min]; $[\alpha]_D^{29}$ = -19.0 (c = 1.0, $CHCl_3$). ^1H NMR (400 MHz, $CDCl_3$) δ 2.00-2.06 (m, 1H), 2.32-2.39 (m, 1H), 2.35 (s, 3H), 2.61-2.64 (m, 2H), 3.26 (br s, 1H), 3.47 (d, *J* = 8.8 Hz, 1H), 4.32 (s, 1H), 5.07 (dt, *J* = 1.6, 16.8 Hz, 1H), 5.19 (dt, *J* = 1.2, 10.4 Hz, 1H), 6.31 (ddd, *J* = 8.8, 10.4, 17.2 Hz, 1H), 6.42 (dd, *J* = 4.4, 8.4 Hz, 1H), 6.69-6.78 (m, 2H), 6.85-6.88 (m, 2H), 7.15-7.19 (m, 3H); ^{13}C NMR (150 MHz, $CDCl_3$) δ 36.9, 37.9, 52.4, 58.5, 61.9, 86.6, 110.3 (*J* = 8.1 Hz), 112.0 (*J* = 24.2 Hz), 114.3 (*J* = 23.6 Hz), 117.5, 126.7, 128.1, 128.9, 135.2 (*J* = 7.5 Hz), 137.7, 140.6, 147.0, 157.1 (*J* = 234 Hz). ^{19}F NMR (376 MHz, $CDCl_3$) δ -125.7 (m). IR (thin film): ν_{max} (cm^{-1}) = 3390, 2930, 2857, 2790, 1603, 1486, 1448, 1347, 1247, 1184, 1158, 1113, 996, 917, 871, 804, 750, 702, 670; HRMS (ESI) calcd for $C_{20}H_{22}FN_2$ $[M+H]^+$: 309.1762. Found: 309.1764.

Ir-catalyzed asymmetric allylic alkylation of tryptophan derivatives

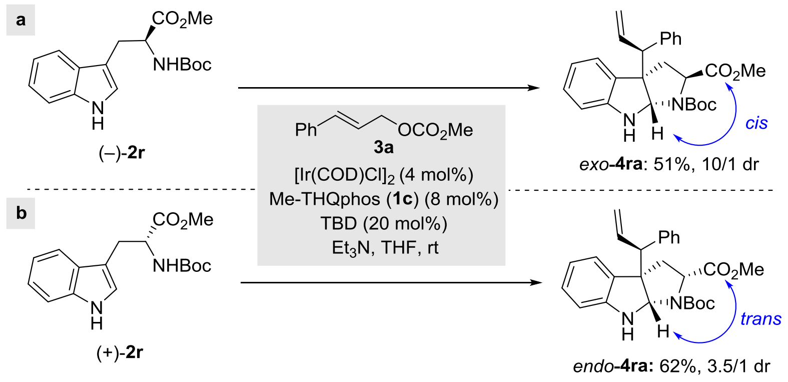

A flame-dried Schlenk tube was cooled to room temperature and filled with argon. To this flask were added $[Ir(COD)Cl]_2$ (10.8 mg, 0.016 mmol, 4.0 mol%), (R,R_a)-**1c** (14.8 mg, 0.032 mmol, 8.0 mol%), TBD (11.2 mg, 0.08 mmol, 20 mol%), and THF (1.0 mL). The reaction mixture was stirred at room temperature for 10 min, generating an orange solution. To another Schlenk tube were added indole derivative **2r** (0.8 mmol, 200 mol%), allylic carbonate **3a** (0.40 mmol, 100 mol%), Et_3N (40.4 mg, 0.40 mmol, 100 mol%), and THF (3 mL). Then, the above pre-formed catalyst solution was transferred to this tube. The reaction mixture was stirred at room temperature until allylic carbonate **3** was fully consumed (monitored by TLC). Upon completion of the reaction, the mixture was concentrated under reduced pressure. The ratio of branched/linear ratio and diastereoselectivity of **4ra** were determined by ^{1}H NMR of the crude reaction mixture. Then the residue was purified by silica gel column chromatography (PE/EA = 20/1) to afford the desired product **4ra**. The characterization data of the products are summarized below.

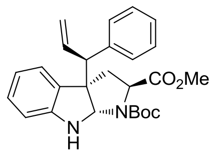

cis-**4ra**. Yellow oil, 51% yield. $[\alpha]_D^{24}$ = -226.0 (c = 1.0, $CHCl_3$). ^{1}H NMR (600 MHz, d_6-DMSO) δ 1.37-1.47 (m, 9H), 2.41-2.55 (m, 2H), 3.56 (d, J = 7.8 Hz, 1H), 3.71 (s, 3H), 3.86 (br, 1H), 5.08 (d, J = 16.8 Hz, 1H), 5.17 (d, J = 10.2 Hz, 1H), 5.32 (s, 1H), 5.86 (br s, 1H), 6.35 (ddd, J = 10.2, 10.2, 17.4 Hz, 1H), 6.58 (d, J = 7.8 Hz, 1H), 6.62 (t, J = 7.8 Hz, 1H), 6.80 (br s, 1H), 6.94 (t, J = 4.2 Hz, 2H), 7.04 (t, J = 7.8

Hz, 1H), 7.20-7.21 (m, 3H). ^{13}C NMR (100 MHz, d_6-DMSO) δ 27.6, 38.2, 51.4, 55.7, 58.7, 66.1, 108.7, 116.9, 117.0, 123.8, 125.8, 126.2, 127.5, 127.6, 128.1, 128.4, 129.3, 137.4, 139.9, 149.4, 152.5, 172.3. IR (thin film): ν_{max} (cm^{-1}) = 3399, 2977, 2322, 1747, 1690, 1607, 1482, 1392, 1362, 1333, 1316, 1258, 1198, 1155, 1048, 1032, 994, 922, 899, 863, 780, 749, 701; HRMS (ESI) calcd for $C_{26}H_{31}N_2O_4$ [M+H]$^+$: 435.2278. Found: 435.2278.

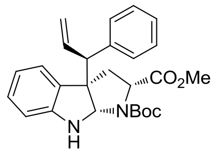

trans-**4ra**. Yellow oil, 62% yield. $[\alpha]_D^{24}$ = -110.6 (c = 1.0, $CHCl_3$). ^{1}H NMR (600 MHz, d_6-DMSO, minor diastereomer indicated by *) δ 1.39-1.44 (m, 9H), 2.41 (d, *J* = 13.2 Hz, 1H), 2.71 (t, *J* = 12.0 Hz, 1H), 3.16 (s, 3H), 3.63 (d, *J* = 4.8 Hz, 1H), 4.29 (d, *J* = 5.4 Hz, 1H), 5.08 (d, *J* = 16.8 Hz, 1H), 5.12 (d, *J* = 8.4 Hz, 1H*), 5.14 (d, *J* = 10.2 Hz, 1H), 5.28 (d, *J* = 17.4 Hz, 1H*), 5.37 (s, 1H), 6.00-6.06 (m, 1H*), 6.31 (ddd, *J* = 10.2, 10.2, 18.6 Hz, 1H), 6.52-6.54 (m, 2H), 6.70 (d, *J* = 7.2 Hz, 1H), 6.98 (t, *J* = 7.8 Hz, 1H), 7.01 (d, *J* = 3.0 Hz, 2H), 7.23-7.26 (m, 3H). ^{13}C NMR (100 MHz, d_6-DMSO) δ 27.7, 38.4, 50.8, 55.6, 58.7, 73.2, 79.2, 108.2, 112.9, 116.3, 116.7, 117.0, 123.7, 125.8, 126.2, 126.4, 127.5, 127.6, 128.0, 128.3, 128.6, 128.9, 137.7, 140.1, 141.8, 144.0, 150.4, 152.7, 170.9. IR (thin film): ν_{max} (cm^{-1}) = 3404, 2976, 2322, 1754, 1734, 1690, 1608, 1483, 1453, 1389, 1366, 1322, 1230, 1168, 1112, 1033, 994, 903, 863, 744, 720, 700; HRMS (ESI) calcd for $C_{26}H_{31}N_2O_4$ [M+H]$^+$: 435.2278. Found: 435.2279.

Gram-scale synthesis of 4aa

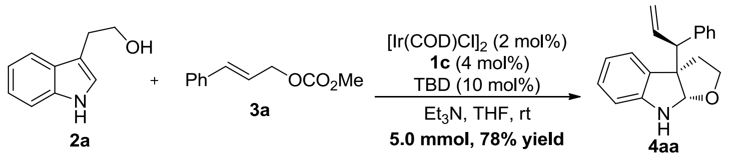

A flame-dried Schlenk tube was cooled to room temperature and filled with argon. To this flask were added $[Ir(COD)Cl]_2$ (67.1 mg, 0.10 mmol, 2.0 mol%), (*R*,*R*$_a$)-**1c** (92.2 mg, 0.20 mmol, 4.0 mol%), TBD (69.5 mg, 0.50 mmol, 10 mol%),

and THF (5.0 mL). The reaction mixture was stirred at room temperature for 10 min, yielding an orange solution. To another Schlenk tube were added tryptophol **2a** (1.61 g, 10.0 mmol, 200 mol%), cinnamyl methyl carbonate **3a** (960.0 mg, 5.0 mmol, 100 mol%), Et_3N (505.0 mg, 5.0 mmol, 100 mol%), and THF (15 mL). Then, the above pre-formed catalyst solution was transferred to this tube. The reaction mixture was stirred at room temperature until allylic carbonate **3a** was fully consumed (monitored by TLC). Upon completion of the reaction, the mixture was concentrated under reduced pressure. The diastereoselectivity of **4aa** was determined by ^{1}H NMR of the crude reaction mixture. Then the residue was purified by silica gel column chromatography (PE/EA = 20/1) to afford the desired product **4aa** (1.08 g, 19/1 dr, 78% yield, 95% ee).

Procedure for the synthesis of 7

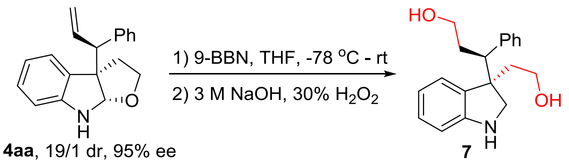

4aa, 19/1 dr, 95% ee **7**

Compound **4aa** (170.4 mg, 0.62 mmol, 95% ee) was dissolved in degassed THF (4 mL) and then 9-BBN (6.0 mL, 0.5 M in THF, 3.0 mmol) was added at -78 ℃. The reaction mixture was allowed to warm to room temperature slowly and stirred overnight. Then the reaction mixture was cooled to 0 ℃, 3M aqueous NaOH (1.5 mL) solution was added. After 5 min, 30% H_2O_2 (2.0 mL) was added by syringe. After 8 h, saturated aqueous Na_2SO_3 solution was added, then the reaction mixture was extracted three times with Et_2O (10.0 mL). The combined organic layers were washed with brine, dried over Na_2SO_4, filtered, and concentrated. The diastereoselectivity of **7** was determined by ^{1}H NMR of the crude reaction mixture. Then the residue was purified by silica gel column chromatography (PE:EA = 1:1) to give **7** as yellow oil in 45% yield (83.0 mg). The enantiomeric excess was determined by HPLC analysis [Daicel Chiralpak IC (0.46 cm x 25 cm), *n*-hexane/2-propanol = 80/20, v = 0.7 mL·min^{-1}, λ = 230 nm, t (minor) = 25.62 min, t (major) = 27.46 min] to be 95%. $[\alpha]_D^{24}$ = -9.9 (c = 1.0, $CHCl_3$). ^{1}H NMR (400 MHz, $CDCl_3$) δ 1.53 (dt, J = 4.0, 14.0

Hz, 1H), 1.67-1.74 (m, 1H), 1.85-1.91 (m, 2H), 3.04-3.11 (m, 1H), 3.19 (dd, *J* = 4.4, 9.6 Hz, 1H), 3.20-3.26 (m, 3H), 3.29-3.40 (m, 3H), 3.43-3.49 (m, 1H), 3.92 (d, *J* = 8.8 Hz, 1H), 6.71 (d, *J* = 7.6 Hz, 1H), 6.84 (dt, *J* = 0.8, 7.6 Hz, 1H), 7.00 (d, *J* = 7.6 Hz, 1H), 7.10 (dt, *J* = 1.6, 8.0 Hz, 1H), 7.17-7.19 (m, 2H), 7.23-7.32 (m, 3H). ^{13}C NMR (100 MHz, $CDCl_3$) δ 35.0, 44.0, 49.4, 52.5, 54.2, 60.0, 61.0, 110.7, 120.1, 123.2, 126.8, 127.9, 128.3, 128.9, 133.4, 141.5, 150.8. IR (thin film): ν_{max} (cm^{-1}) = 2963, 2361, 2337, 1261, 1091, 1020, 866, 798, 699; HRMS (ESI) calcd for $C_{19}H_{24}NO_2$ $[M+H]^+$: 298.1802. Found: 298.1802.

Procedure for Pt/C catalyzed hydrogenation of compound 4aa

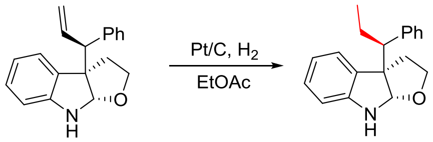

4aa, 19/1 dr, 95% ee **8**

To a solution of **4aa** (164.2 mg, 0.59 mmol, 95% ee) in EtOAc (10.0 mL), Pt/C (63.7 mg, platinum on activated charcoal, 10% Pt basis, 5 mol%) was added. The reaction mixture was stirred under H_2 atmosphere (1 atm) at room temperature for 24 h. After the reaction was complete (monitored by LC-MS), the crude reaction mixture was filtered with celite and washed with EtOAc. The solvents were removed under reduced pressure. Then the residue was purified by silica gel column chromatography (PE/EA = 9/1) to afford the desired product **8**. White solid, 132.4 mg, 80% yield, 95% ee [Daicel Chiralpak IC (0.46 cm x 25 cm), *n*-hexane/2-propanol = 98/2, *v* = 0.5 mL·min^{-1}, λ = 254 nm, t (minor) = 28.95 min, t (major) = 36.78 min]; $[\alpha]_D^{24}$ = -32.4 (c = 1.0, $CHCl_3$, minor diastereomer indicated by *). ^{1}H NMR (400 MHz, $CDCl_3$) δ 0.70 (t, *J* = 7.2 Hz, 3H), 1.87-1.97 (m, 2H), 2.19-2.22 (m, 2H), 2.72 (dd, *J* = 4.8, 10.4 Hz, 1H), 3.43-3.50 (m, 1H), 3.88-3.93 (m, 1H), 4.30 (br s, 1H), 5.35 (s, 1H), 6.44 (d, *J* = 7.6 Hz, 1H), 6.56 (d, *J* = 8.4 Hz, 1H*), 6.66 (t, *J* = 7.6 Hz, 1H*), 6.72 (t, *J* = 7.6 Hz, 1H), 6.84-6.86 (m, 2H), 7.01-7.06 (m, 2H), 7.10-7.14 (m, 3H). ^{13}C NMR (100 MHz, $CDCl_3$) δ 12.7, 24.7, 39.5, 55.8, 62.3, 67.6, 96.6, 108.3, 118.3, 124.4, 126.5, 127.8, 128.2, 128.7, 130.6, 141.4, 150.6. IR (thin film): ν_{max} (cm^{-1}) = 3414, 3077, 3020, 2965, 2935, 2871, 1604, 1465, 1410, 1336, 1313, 1260, 1093, 1064, 1004, 966,

946, 877, 744, 708, 657; HRMS (ESI) calcd for $C_{19}H_{22}NO$ $[M+H]^+$: 280.1696. Found: 280.1701. mp = 93-97 °C.

Procedure for the synthesis of 9

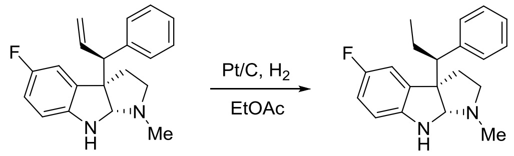

4ma, 99% ee, >20/1 dr   **9**

To a solution of **4ma** (71.8 mg, 0.20 mmol, 99% ee) in EtOAc (10.0 mL), Pt/C (12.0 mg, platinum on activated charcoal, 10% Pt basis, 5 mol%) was added. The reaction mixture was stirred under H_2 atmosphere (1 atm) at room temperature for 18 h. After the fully conversion of **4ma** (monitored by LC-MS), the crude reaction mixture was filtrated with celite and washed with EtOAc. The solvents were removed under reduced pressure. Then the residue was purified by silica gel column chromatography (EA/Et_3N = 100/0.1) to afford the desired product **9**. White solid, 65.0 mg, 90% yield, 98% ee [Daicel Chiralpak AD-H (0.46 cm x 25 cm), *n*-hexane/2-propanol/Et_2NH = 90/10/0.1, v = 0.5 mL·min^{-1}, λ = 280 nm, t (major) = 9.00 min, t (minor) = 27.67 min]; $[\alpha]_D^{26}$ = -40.1 (c = 1.0, $CHCl_3$). ^1H NMR (400 MHz, $CDCl_3$, minor diastereomer indicated by *) δ 0.70 (t, *J* = 7.2 Hz, 3H), 1.82-1.96 (m, 2H), 2.05-2.11 (m, 1H), 2.21-2.27 (m, 1H), 2.32 (s, 3H), 2.39 (s, 3H*), 2.49-2.64 (m, 3H), 3.54 (br s, 1H), 4.25 (s, 1H), 4.38 (s, 1H*), 6.40 (dd, *J* = 4.4, 8.4 Hz, 1H), 6.72-6.78 (m, 2H), 6.83-6.85 (m, 2H), 7.15-7.16 (m, 3H). ^{13}C NMR (100 MHz, $CDCl_3$) δ 12.8, 24.4, 37.0, 38.2, 52.5, 56.9, 62.4, 86.7, 110.3 (*J* = 8.1 Hz), 111.7 (*J* = 23.4 Hz), 114.0 (*J* = 23.1 Hz), 126.5, 127.8, 129.0, 135.8 (*J* = 7.1 Hz), 141.5, 147.0, 157.1 (*J* = 233.6 Hz). ^{19}F NMR (376 MHz, $CDCl_3$) δ -125.9. IR (thin film): ν_{max} (cm^{-1}) = 3144, 3073, 3027, 2957, 2928, 2863, 1602, 1487, 1450, 1350, 1254, 1186, 1161, 1111, 1023, 931, 899, 875, 797, 744, 699; HRMS (ESI) calcd for $C_{20}H_{24}FN_2$ $[M+H]^+$: 311.1918. Found: 311.1916. mp = 88.6-90.9 °C.

Determination of the absolute configuration

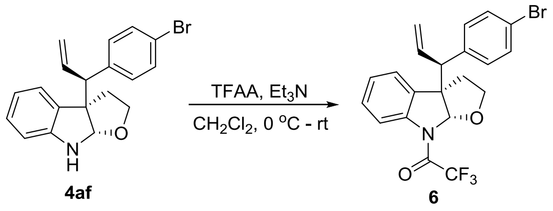

A flame-dried flask was charged with **4af** (340.8 mg, 0.96 mmol, 94% ee), Et_3N (505.0 mg, 5.0 mmol) and CH_2Cl_2 (20 mL), and then cooled to 0 ℃. A solution of TFAA (425.0 mg, 2.02 mmol) in CH_2Cl_2 (5 mL) was added dropwise to this flask. Then, the reaction mixture was stirred at room temperature. After the reaction was complete (monitored by TLC), The reaction mixture was quenched by saturated $NaHCO_3$ aqueous (15 mL), extracted with Et_2O (25 mL x 3). The combined organic layers were washed with brine, separated, and dried over anhydrous Na_2SO_4. The solvent was evaporated, and the residue was purified by silica gel column chromatography (PE/EA = 40/1) to afford the desired product **6**. White solid, 84% yield, >99% ee (after recrystallization) [Daicel Chiralpak IC, *n*-hexane/2-propanol = 100/1, v = 1.0 mL·min^{-1}, λ = 254 nm, t (minor) = 10.55 min, t (major) = 11.26 min]; $[\alpha]_D^{24}$ = +49.7 (c = 1.0, $CHCl_3$). ^{1}H NMR (400 MHz, $CDCl_3$) δ 2.32 (dd, *J* = 4.4, 12.0 Hz, 1H), 2.41 (dt, *J* = 7.6, 12.0 Hz, 1H), 2.47 (ddd, *J* = 4.8, 8.8, 11.6 Hz, 1H), 3.57 (d, *J* = 8.4 Hz, 1H), 4.05 (t, *J* = 8.0 Hz, 1H), 5.14 (d, *J* = 16.8 Hz, 1H), 5.25 (d, *J* = 10.0 Hz, 1H), 5.91 (d, *J* = 1.2 Hz, 1H), 6.24 (ddd, *J* = 8.8, 10.0, 16.8 Hz, 1H), 6.63 (d, *J* = 13.6 Hz, 2H), 7.11 (d, *J* = 7.2 Hz, 1H), 7.19-7.23 (m, 1H), 7.27 (d, *J* = 13.6 Hz, 2H), 7.32-7.37 (m, 1H), 8.06 (d, *J* = 8.4 Hz, 1H); ^{13}C NMR (100 MHz, $CDCl_3$) δ 38.0, 56.5, 61.4, 69.0, 96.2, 115.7 (q, *J* = 287 Hz), 117.7, 118.6, 121.3, 124.6, 125.9, 129.4, 129.9, 131.4, 132.5, 135.9, 137.8, 142.4, 154.8 (q, *J* = 38.0 Hz). ^{19}F NMR (376 MHz, $CDCl_3$) δ -70.2. IR (thin film): ν_{max} (cm^{-1}) = 2922, 2853, 1699, 1595, 1484, 1462, 1432, 1367, 1281, 1241, 1206, 1146, 1119, 1074, 1020, 928, 871, 823, 770, 725, 692; HRMS (ESI) calcd for $C_{21}H_{18}BrF_3NO_2$ $[M+H]^+$: 452.0468. Found: 452.0471. mp = 84.4-85.7 ^oC.

X-ray analysis of 6: In a round-bottom flask, **6** was dissolved in CH_3OH. Then this solution was placed in refrigerator at -20 °C for one week. Colorless crystal containing CH_3OH molecule was obtained and suitable for X-ray diffraction. A representative crystal was surveyed on a Bruker APEX diffractometer. All crystallographic calculations were facilitated by the SHELXL-97 system. (CCDC 1060973 contains the supplementary crystallographic data for **6**. These data can be obtained free of charge from the Cambridge Crystallographic Data Centre via www.ccdc.cam.ac.uk /data request/cif.)

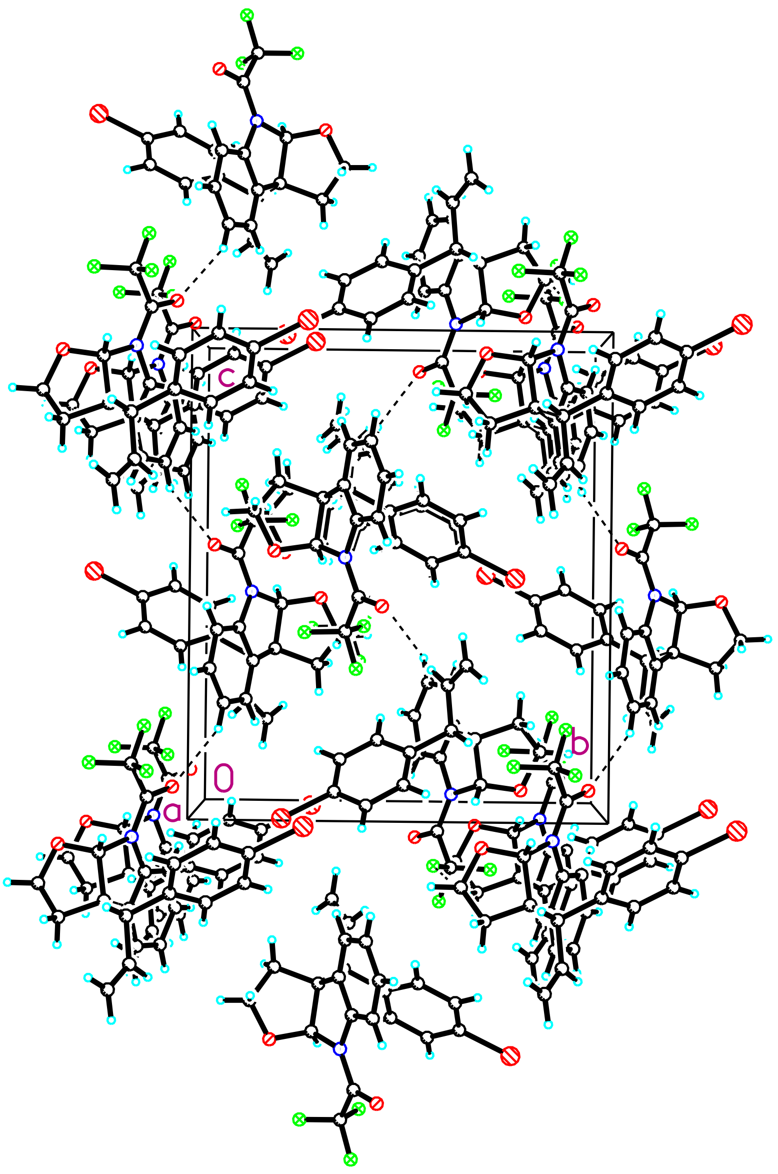

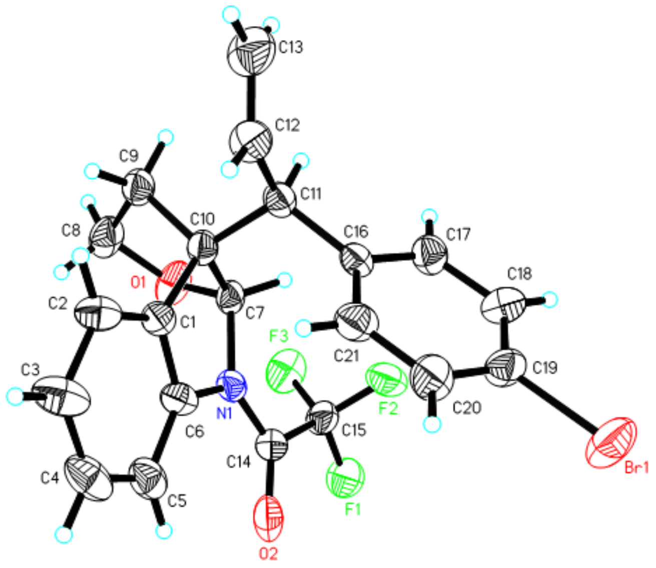

Table 1. Crystal data and structure refinement for cd212454.

Identification code	cd212454
Empirical formula	C21 H17 Br F3 N O2
Formula weight	452.27
Temperature	293(2) K
Wavelength	0.71073 A
Crystal system, space group	Orthorhombic, P2(1)2(1)2(1)
Unit cell dimensions	a = 9.1964(10) A alpha = 90 deg.<br>b = 13.4028(16) A beta = 90 deg.<br>c = 15.6110(17) A gamma = 90 deg.
Volume	1924.2(4) A^3
Z, Calculated density	4, 1.561 Mg/m^3
Absorption coefficient	2.181 mm^-1
F(000)	912
Crystal size	0.212 x 0.143 x 0.101 mm

Theta range for data collection	2.00 to 25.99 deg.
Limiting indices	-11<=h<=11, -14<=k<=16, -18<=l<=19
Reflections collected / unique	11763 / 3776 [R(int) = 0.0602]
Completeness to theta = 25.99	100.0 %
Absorption correction	Empirical
Max. and min. transmission	1.00000 and 0.25258
Refinement method	Full-matrix least-squares on F^2
Data / restraints / parameters	3776 / 0 / 253
Goodness-of-fit on F^2	0.946
Final R indices [I>2sigma(I)]	R1 = 0.0419, wR2 = 0.0797
R indices (all data)	R1 = 0.0963, wR2 = 0.0953
Absolute structure parameter	0.000(11)
Largest diff. peak and hole	0.293 and -0.174 e.A^-3

Compound **4aa**'s ^{1}H NMR Spectra

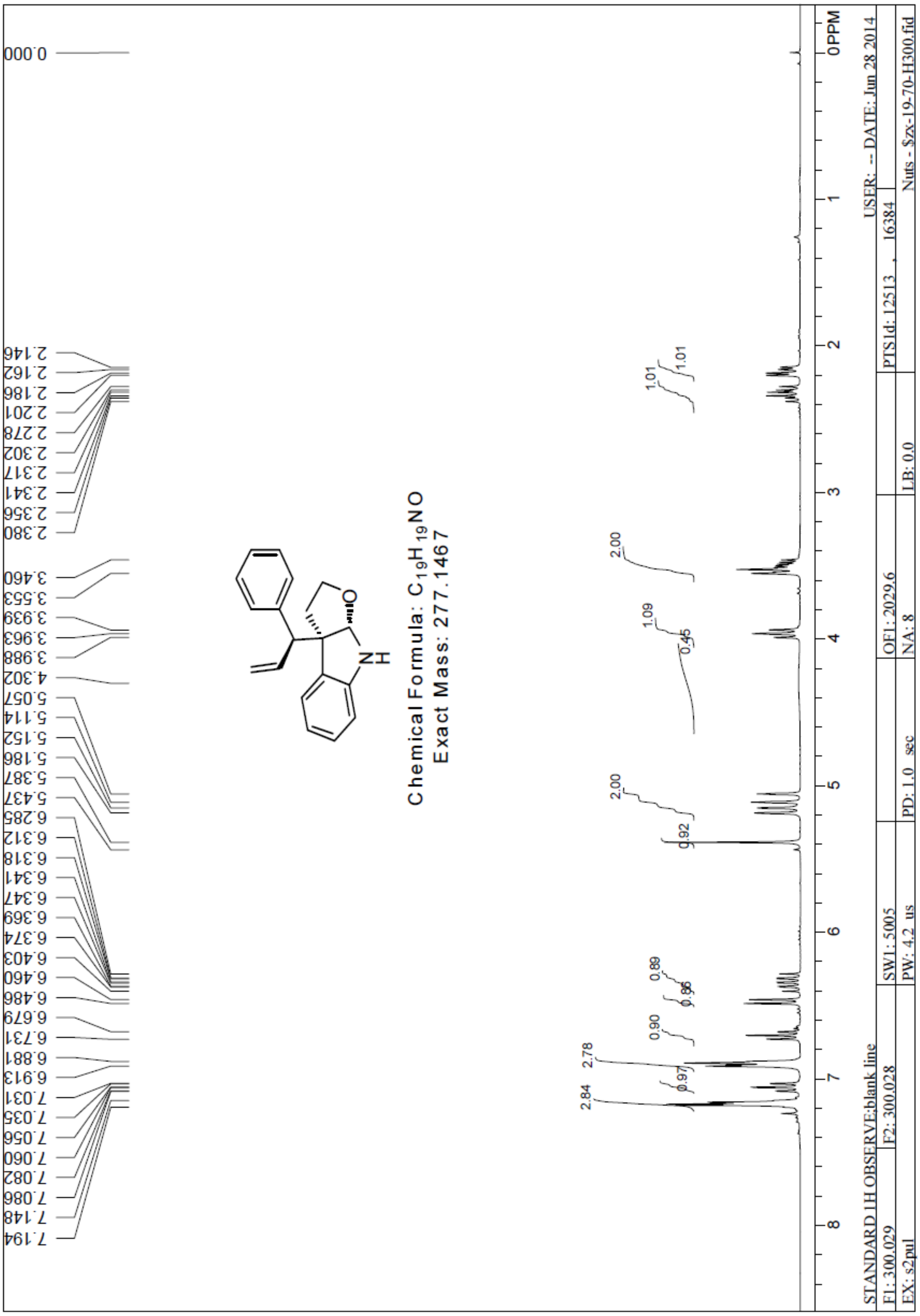

Compound **4aa**’s ^{13}C NMR Spectra

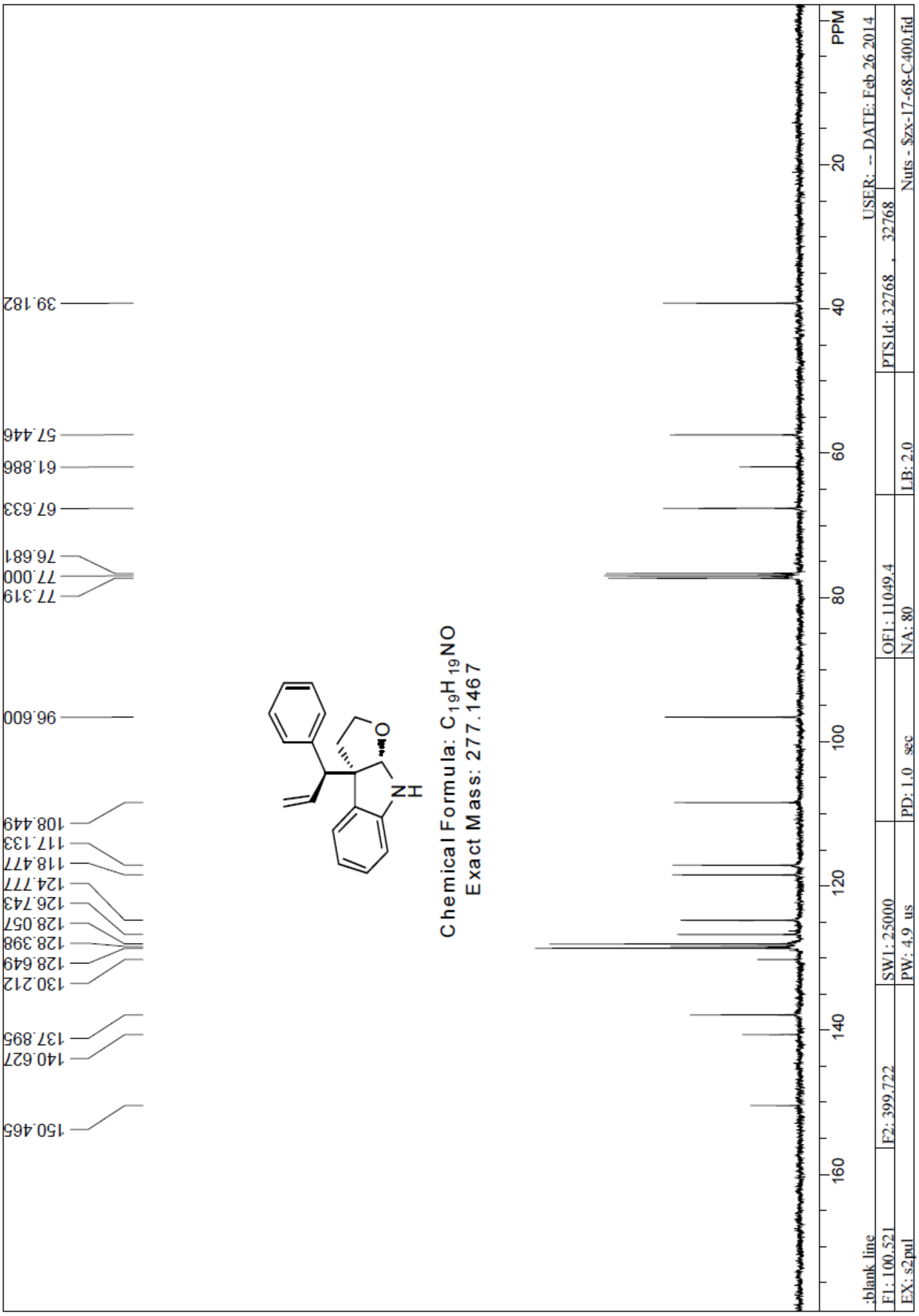

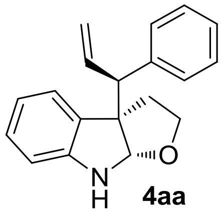

4aa

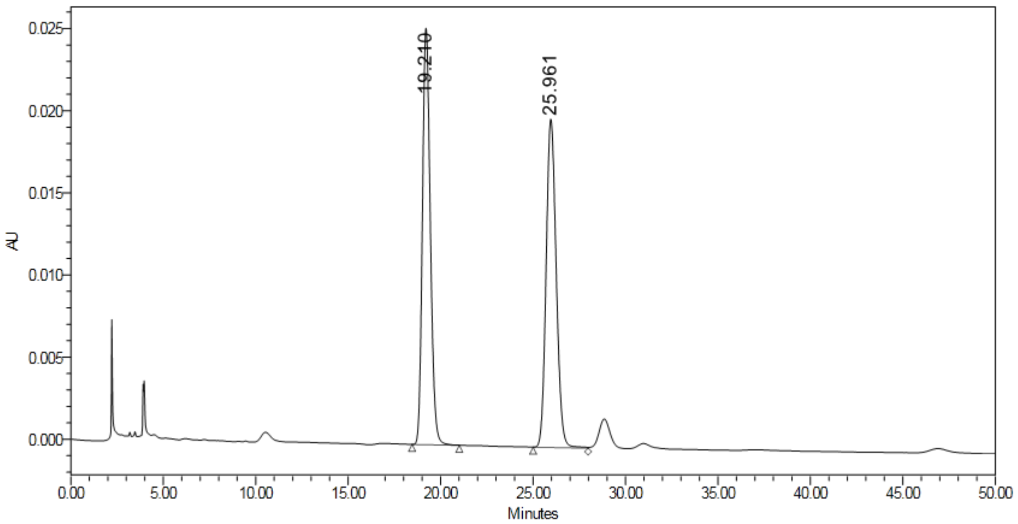

	RT (min)	Area (µV*sec)	% Area	Height (µV)	% Height
1	19.210	751290	49.80	25350	55.91
2	25.961	757291	50.20	19991	44.09

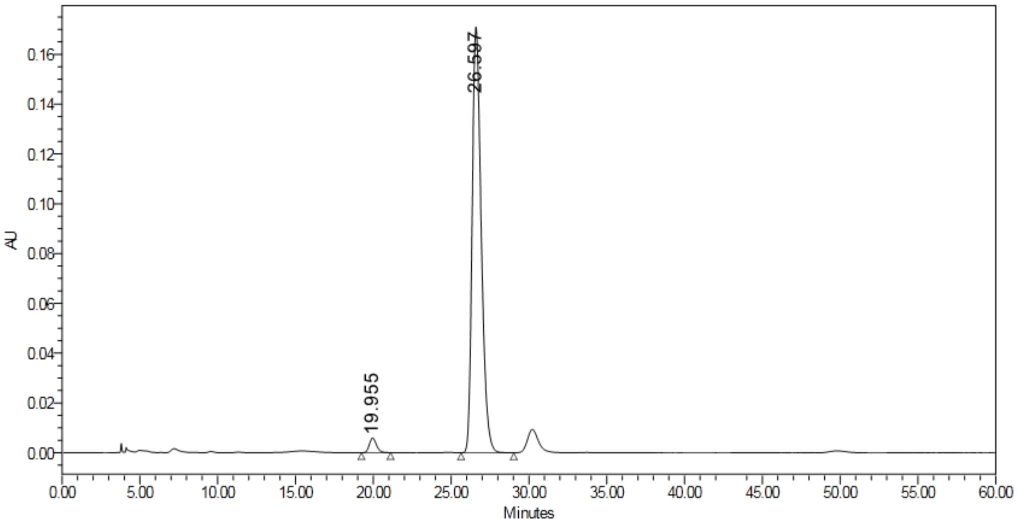

	RT (min)	Area (µV*sec)	% Area	Height (µV)	% Height
1	19.955	190041	2.67	5916	3.34
2	26.597	6917928	97.33	171004	96.66

Compound **4ab**'s ^{1}H NMR Spectra

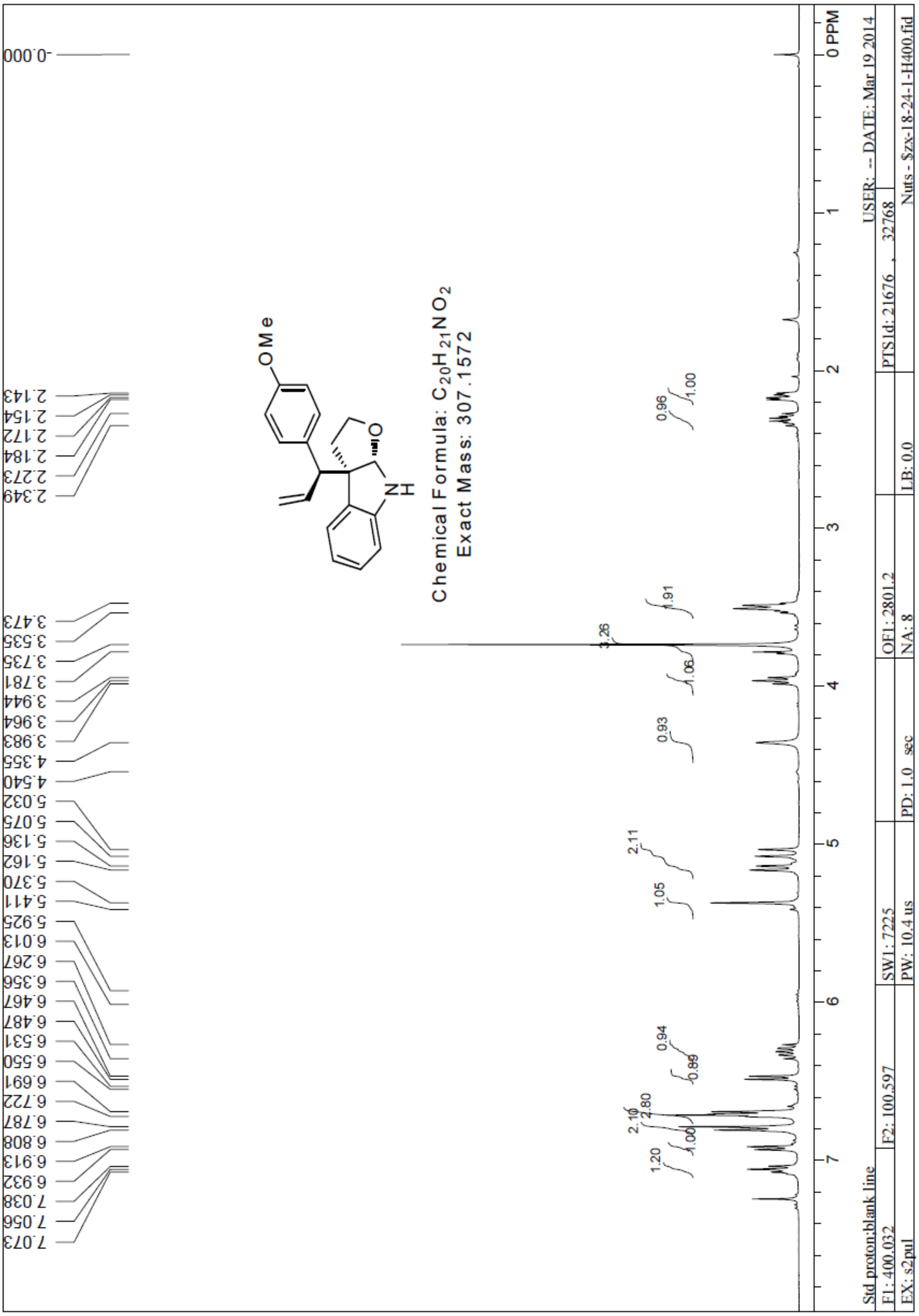

Compound **4ab**'s ^{13}C NMR Spectra

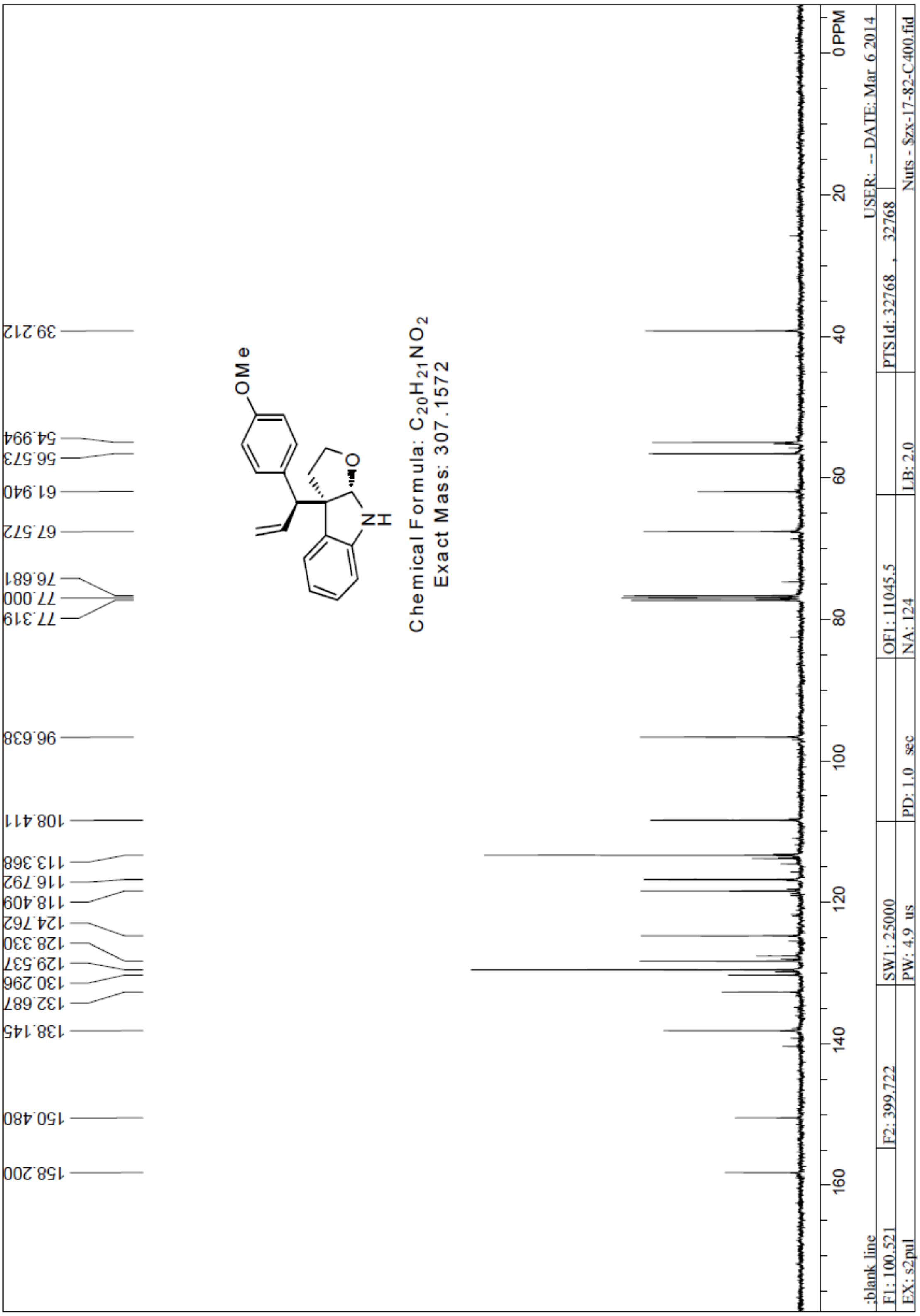

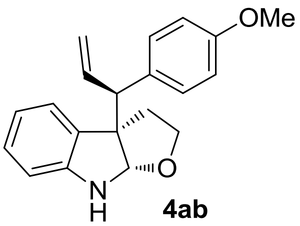

4ab

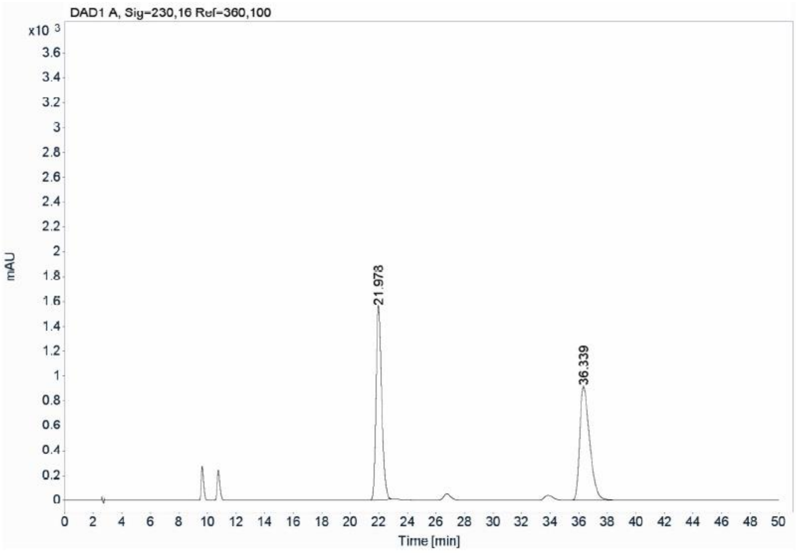

Signal: DAD1 A, Sig=230, 16 Ref=360, 100

RT [min]	Type	Width [min]	Area	Height	Area%
21.978	BV	0.4225	42917.0703	1557.1534	49.3744
36.339	BB	0.6951	44004.6602	913.3144	50.6256
		Sum	86921.7305		

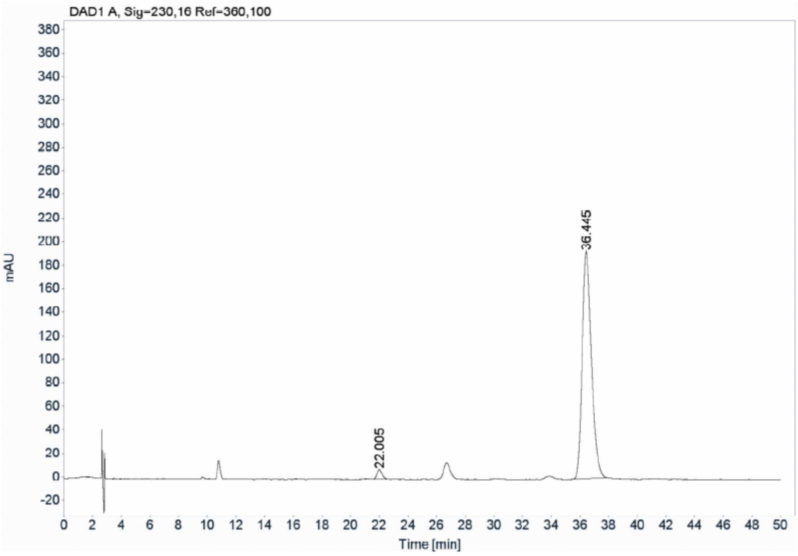

Signal: DAD1 A, Sig=230, 16 Ref=360, 100

RT [min]	Type	Width [min]	Area	Height	Area%
22.005	BB	0.3022	193.0531	7.5778	2.1985
36.445	BB	0.6119	8588.0537	192.3616	97.8015
		Sum	8781.1068		

Compound **4ac**'s ^{1}H NMR Spectra

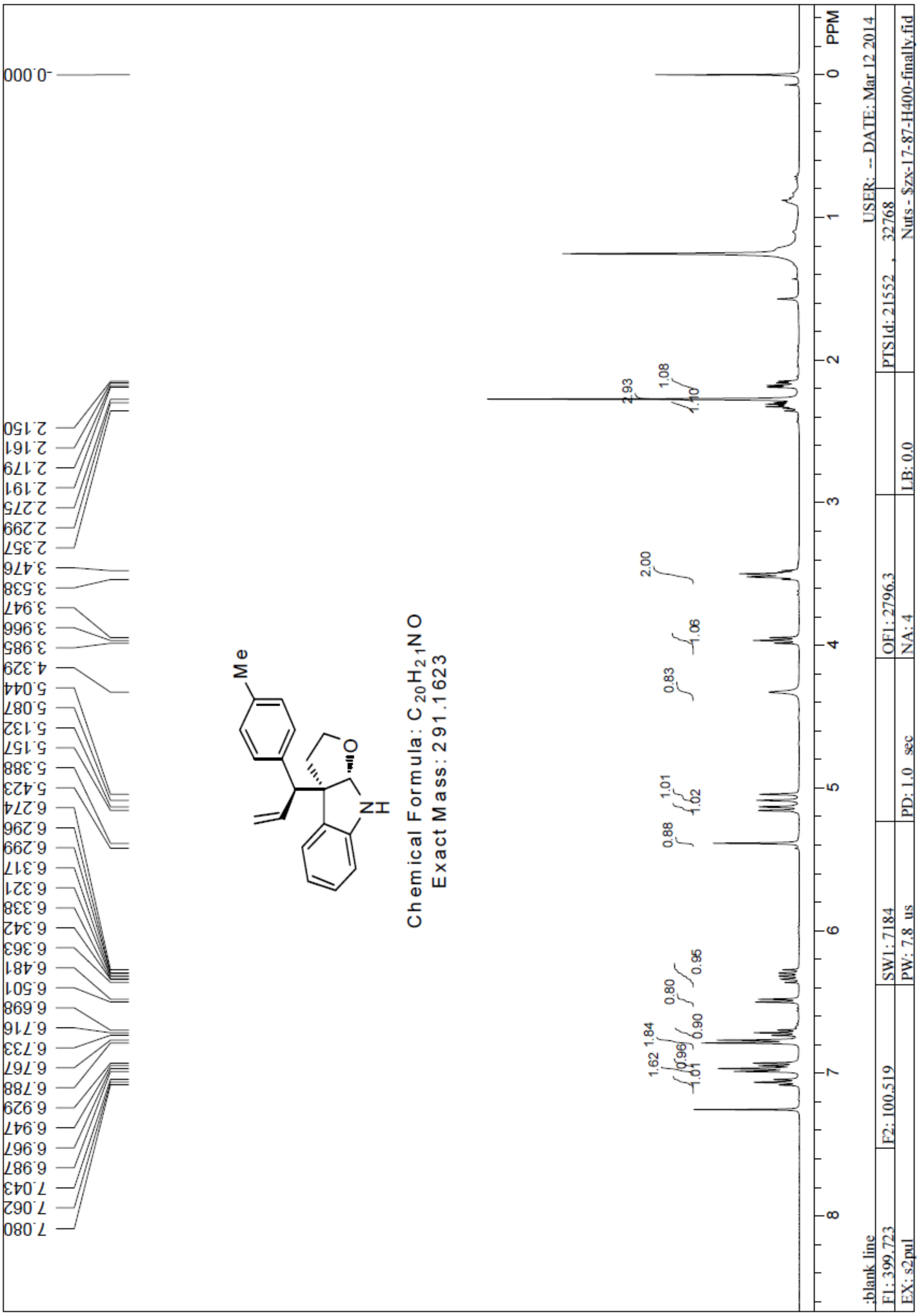

Compound **4ac**'s ^{13}C NMR Spectra

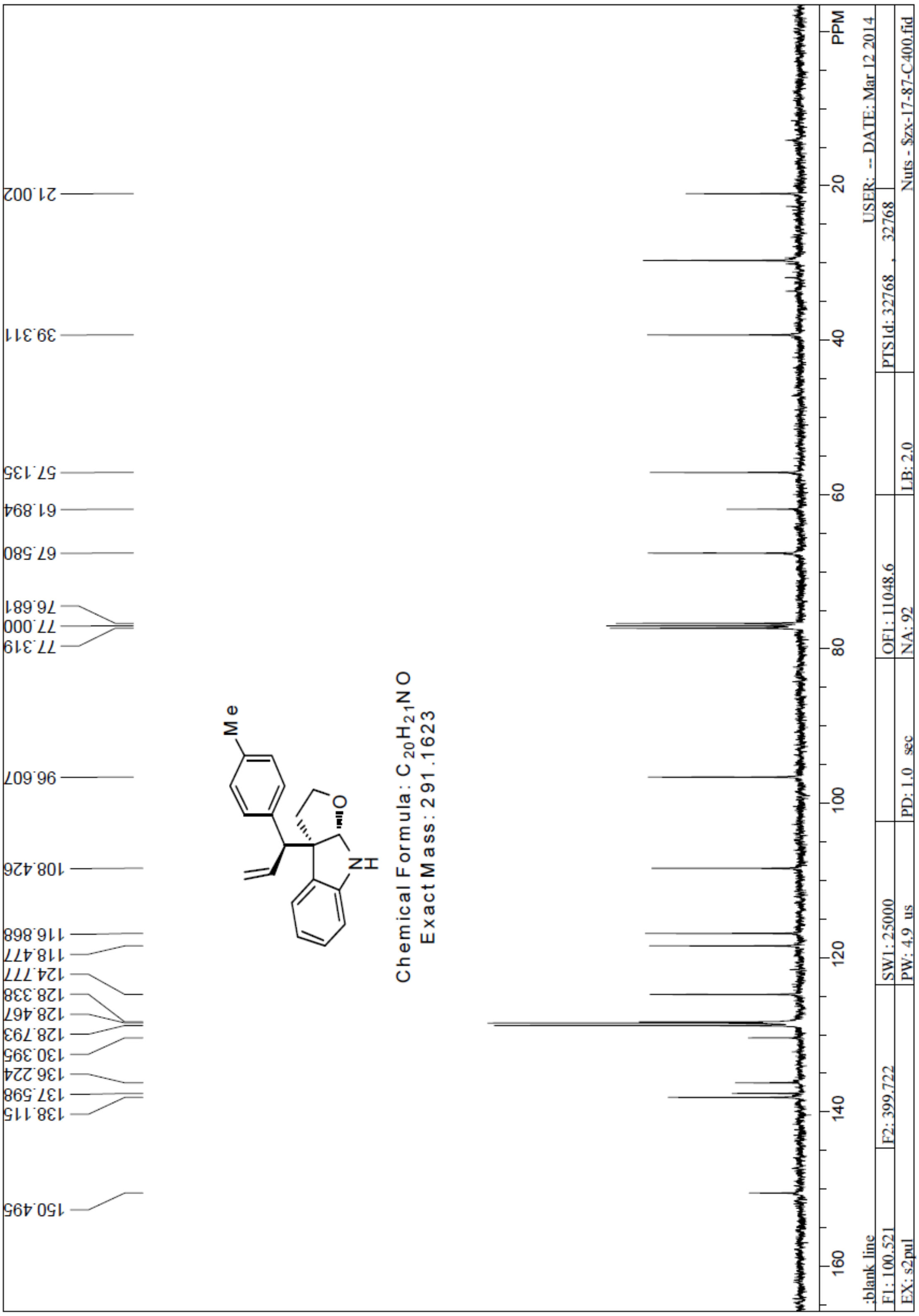

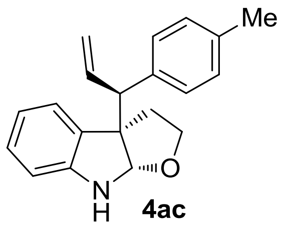

4ac

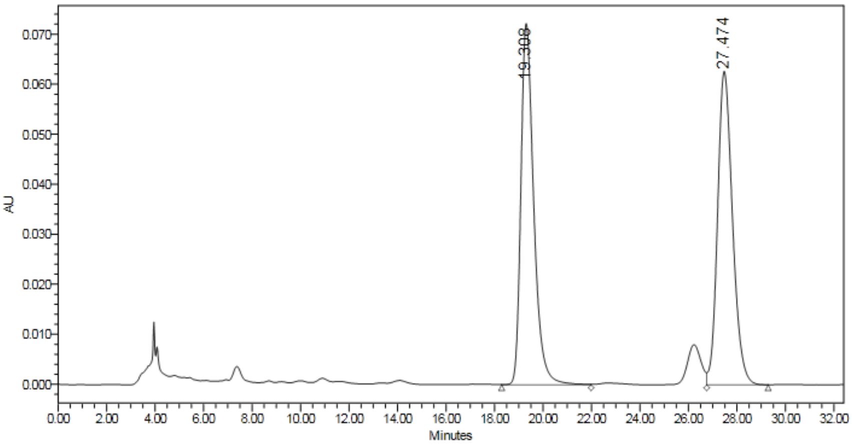

	RT (min)	Area (µV*sec)	% Area	Height (µV)	% Height
1	19.308	2657851	49.74	72193	53.54
2	27.474	2685995	50.26	62655	46.46

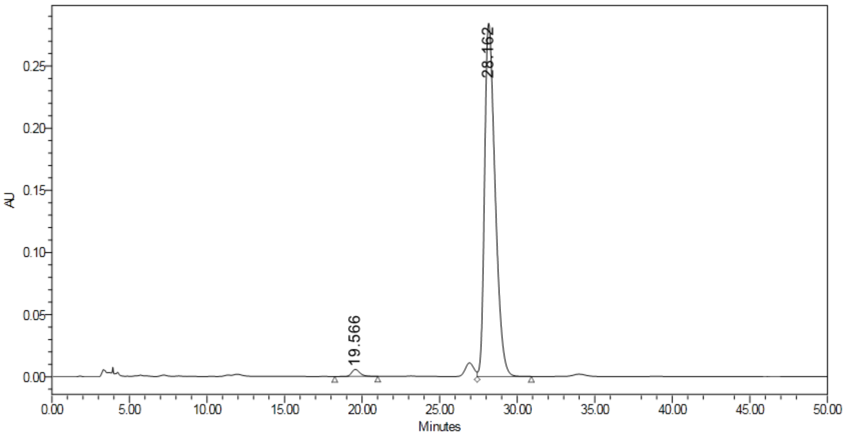

	RT (min)	Area (µV*sec)	% Area	Height (µV)	% Height
1	19.566	214327	1.57	5674	1.96
2	28.162	13440920	98.43	284323	98.04

Compound **4ad**'s ^{1}H NMR Spectra

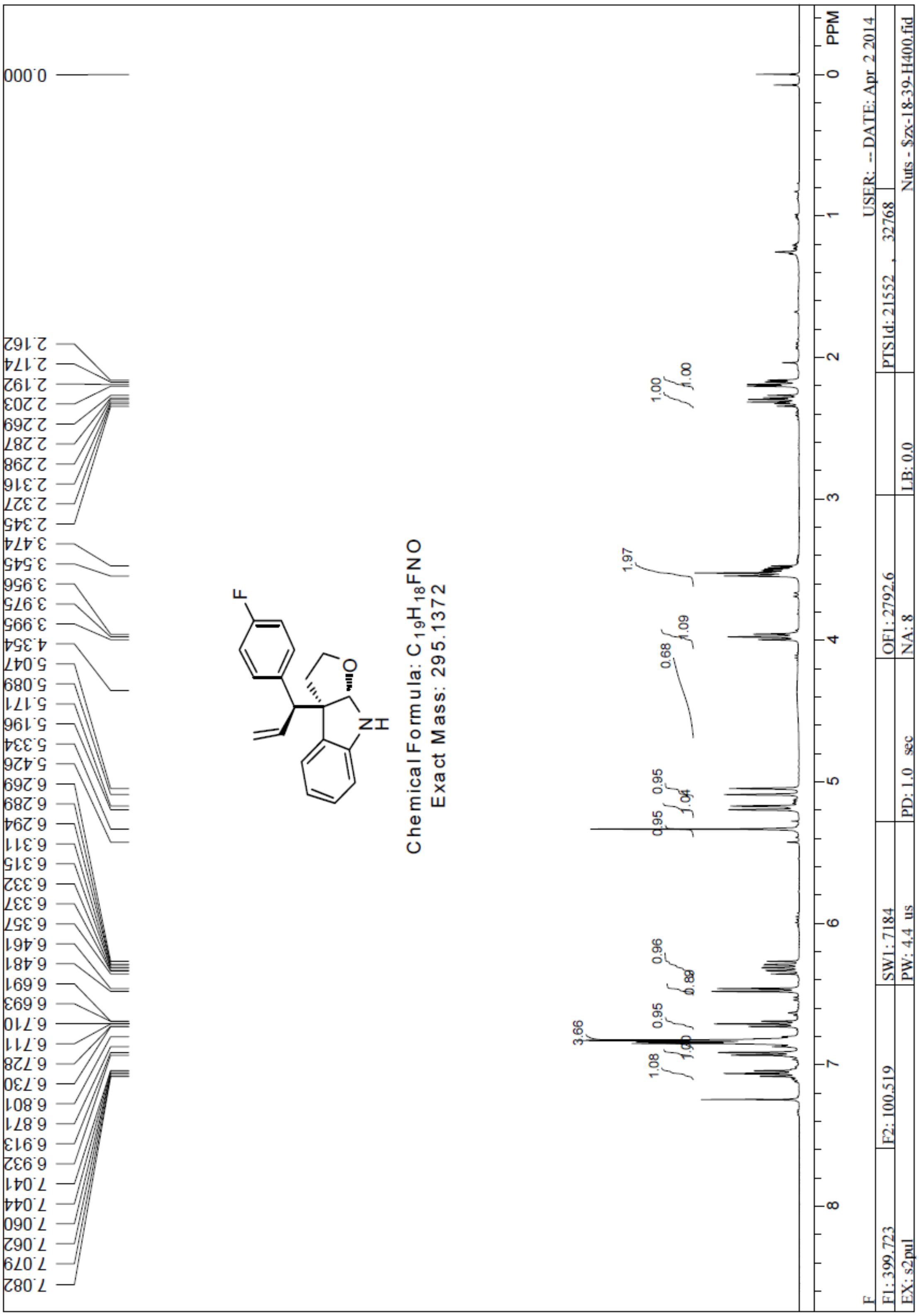

Compound **4ad**'s ^{13}C NMR Spectra

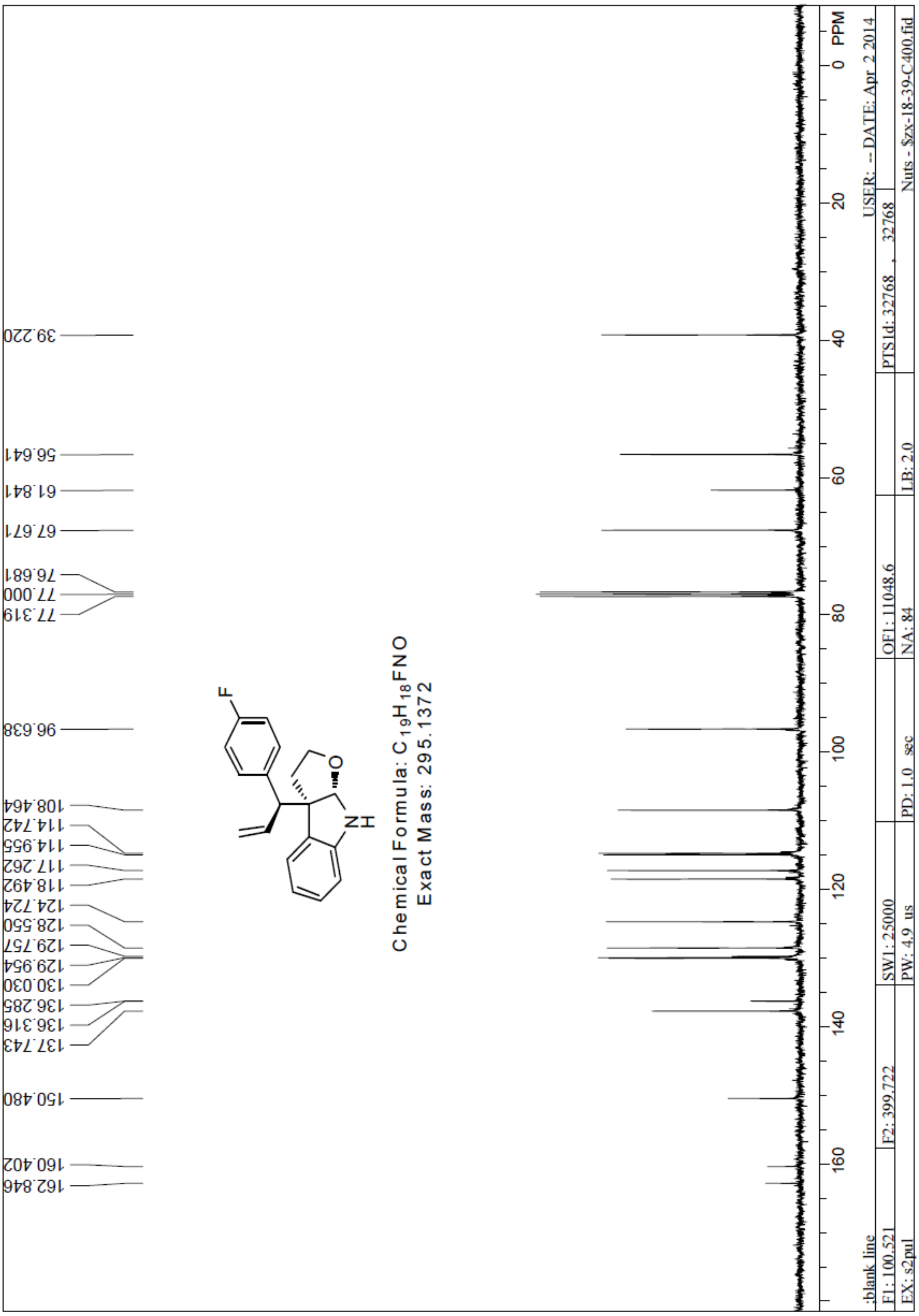

Compound **4ad**'s ^{19}F NMR Spectra

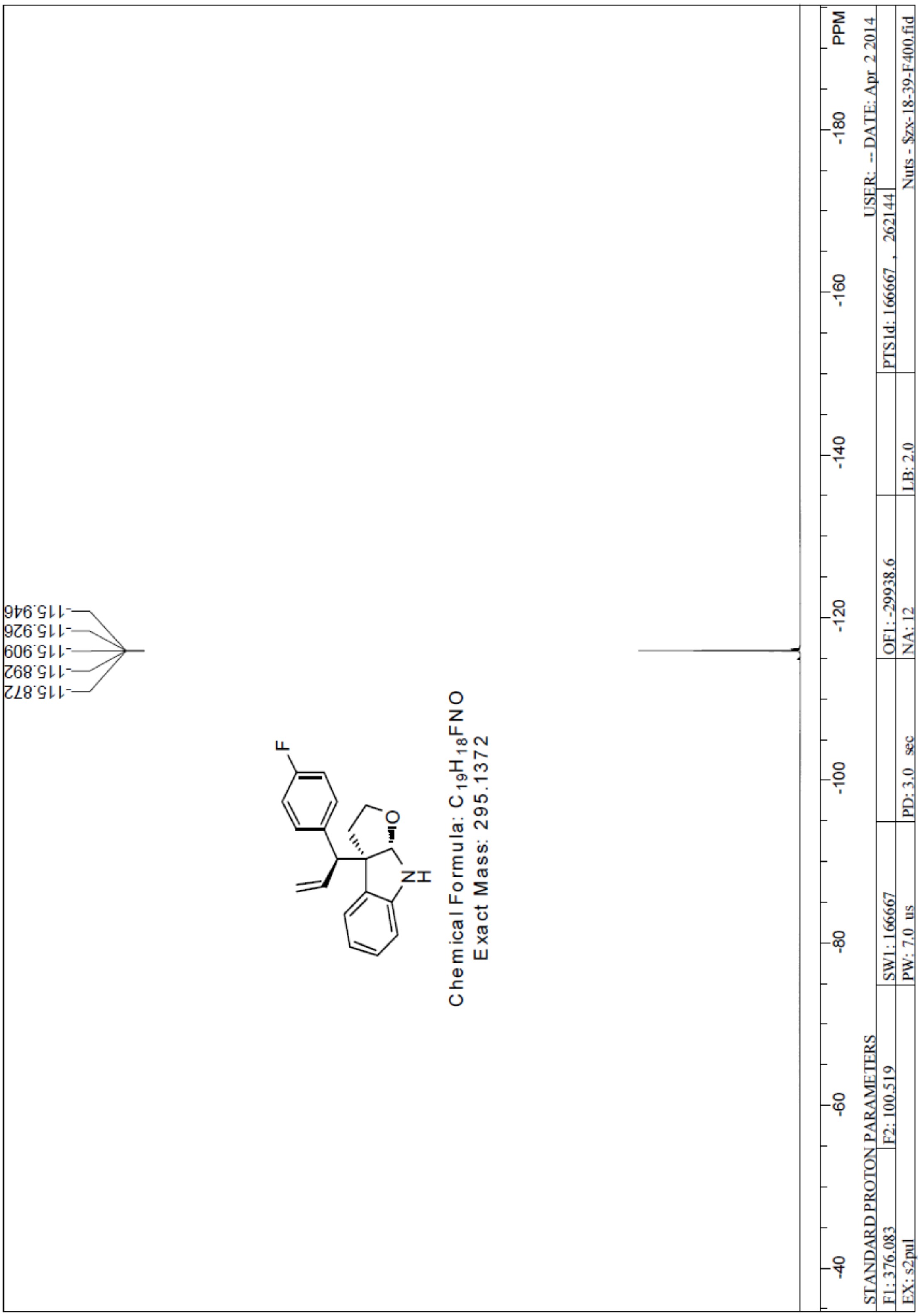

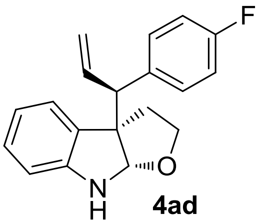

4ad

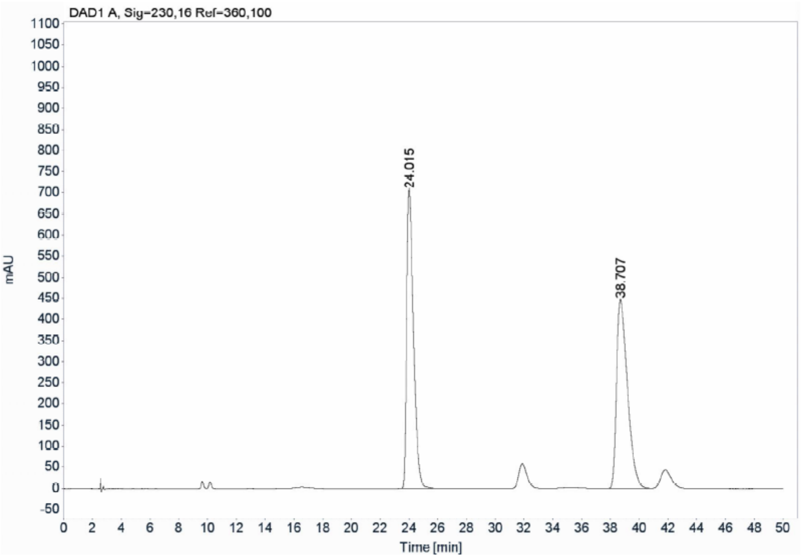

Signal: DAD1 A, Sig=230, 16 Ref=360, 100

RT [min]	Type	Width [min]	Area	Height	Area%
24.015	BB	0.4991	23308.0918	706.2087	49.9011
38.707	BB	0.7776	23400.4629	445.6608	50.0989
		Sum	46708.5547		

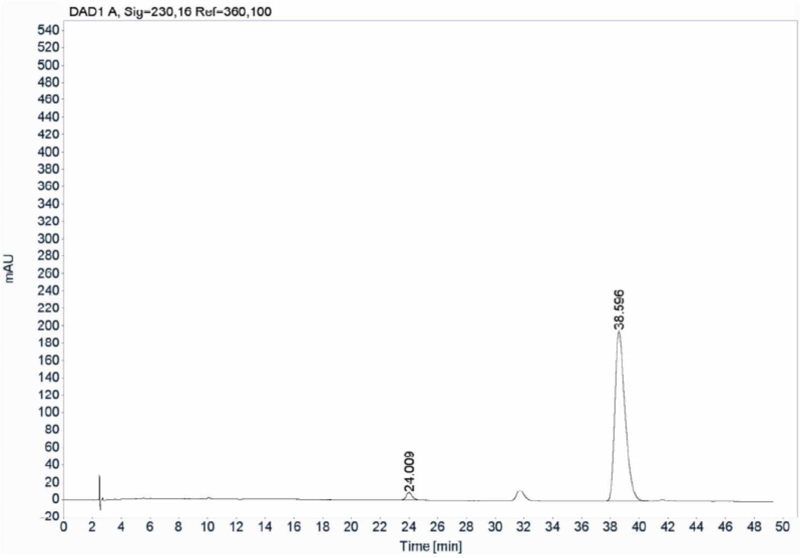

Signal: DAD1 A, Sig=230, 16 Ref=360, 100

RT [min]	Type	Width [min]	Area	Height	Area%
24.009	BB	0.3472	232.5868	7.9521	2.3623
38.596	BB	0.7299	9613.2305	195.0719	97.6377
		Sum	9845.8172		

Compound **4ae**'s ^{1}H NMR Spectra

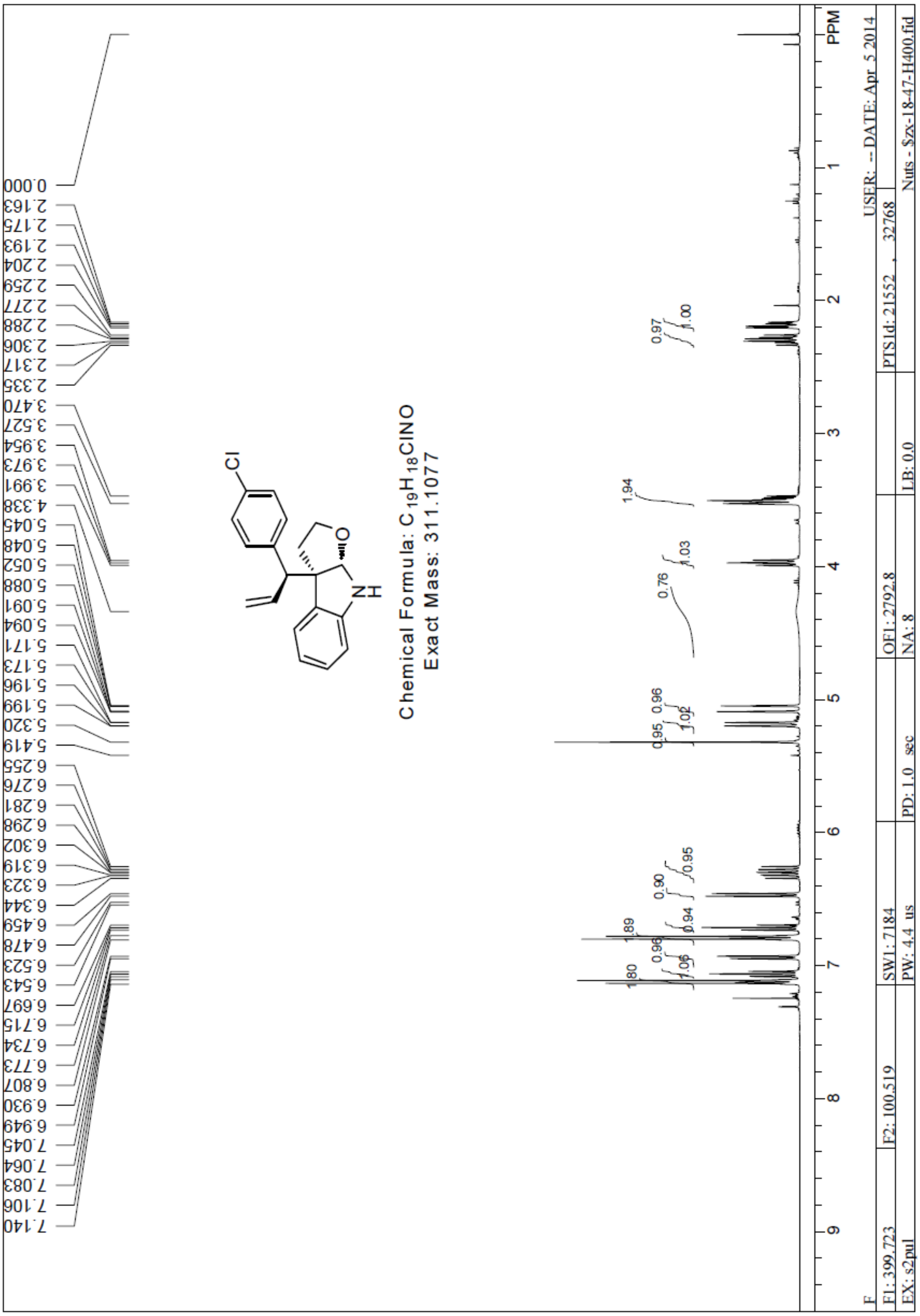

Compound **4ae**'s ^{13}C NMR Spectra

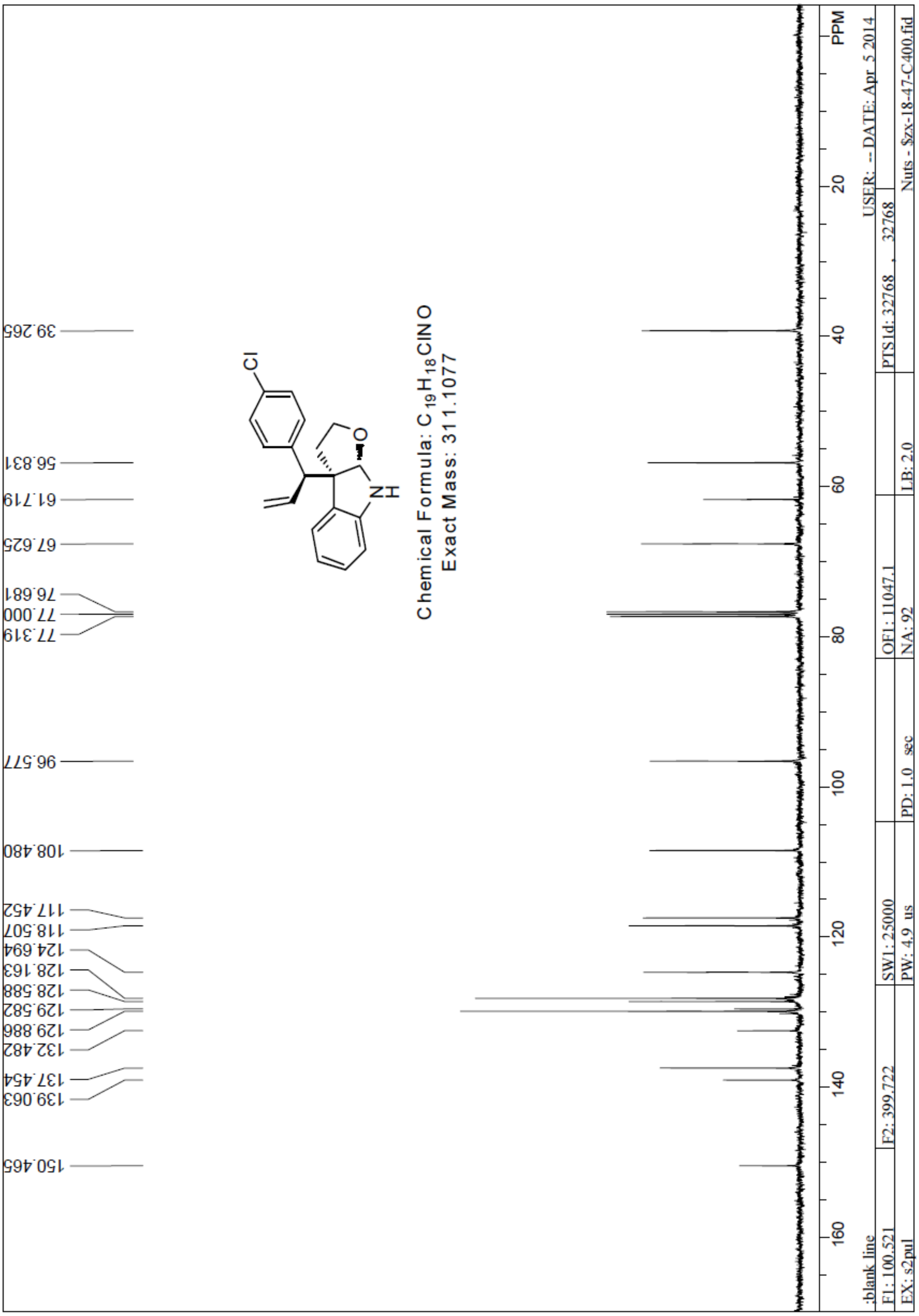

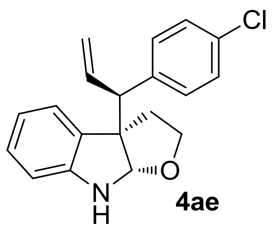

4ae

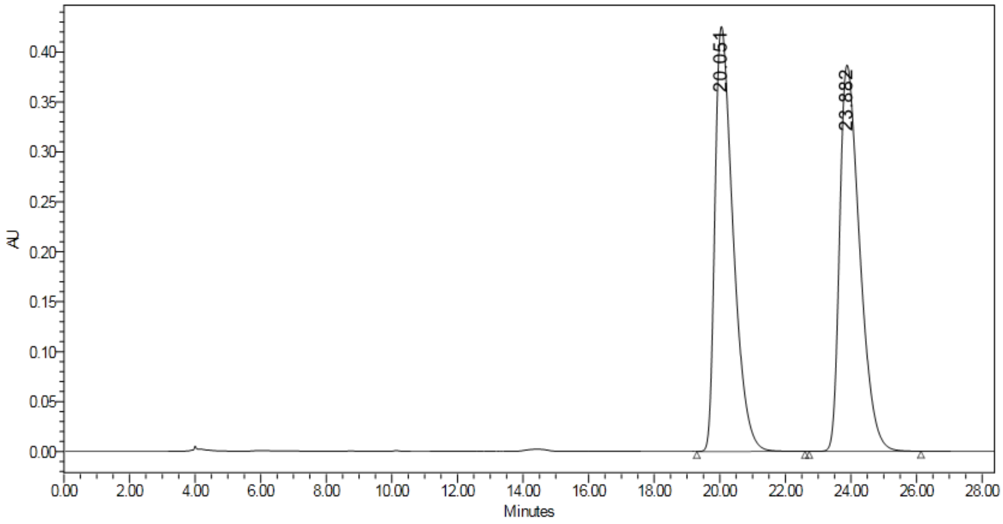

	RT (min)	Area (µV*sec)	% Area	Height (µV)	% Height
1	20.051	16356978	49.88	425374	52.38
2	23.882	16432981	50.12	386676	47.62

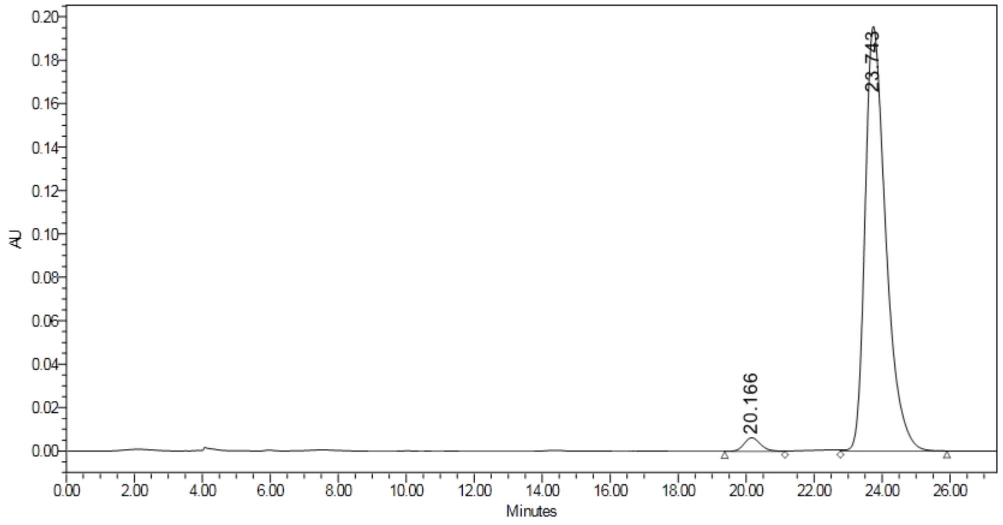

	RT (min)	Area (µV*sec)	% Area	Height (µV)	% Height
1	20.166	210953	2.47	6267	3.11
2	23.743	8324311	97.53	195575	96.89

Compound **4af**'s ^{1}H NMR Spectra

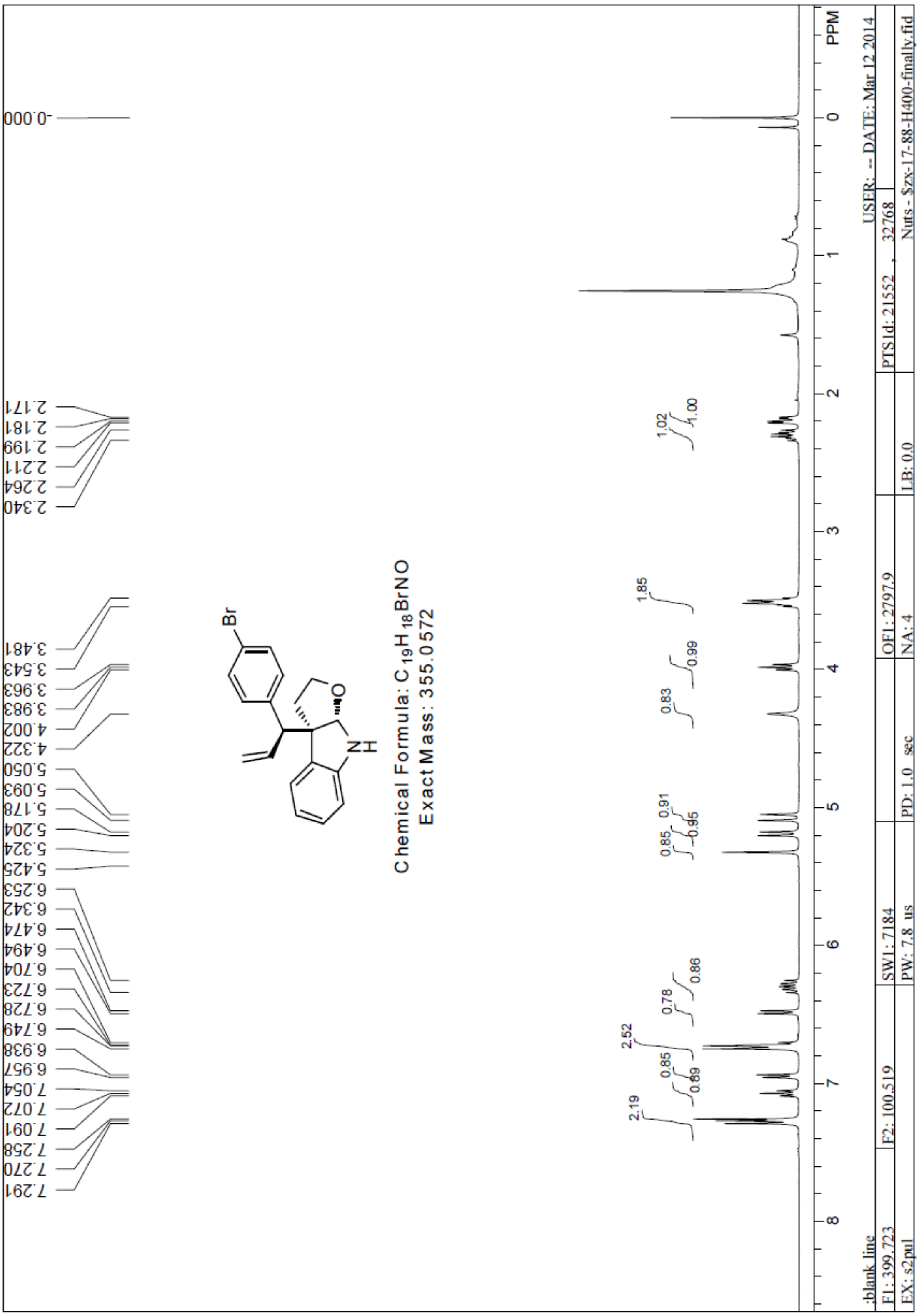

Compound **4af**'s ^{13}C NMR Spectra

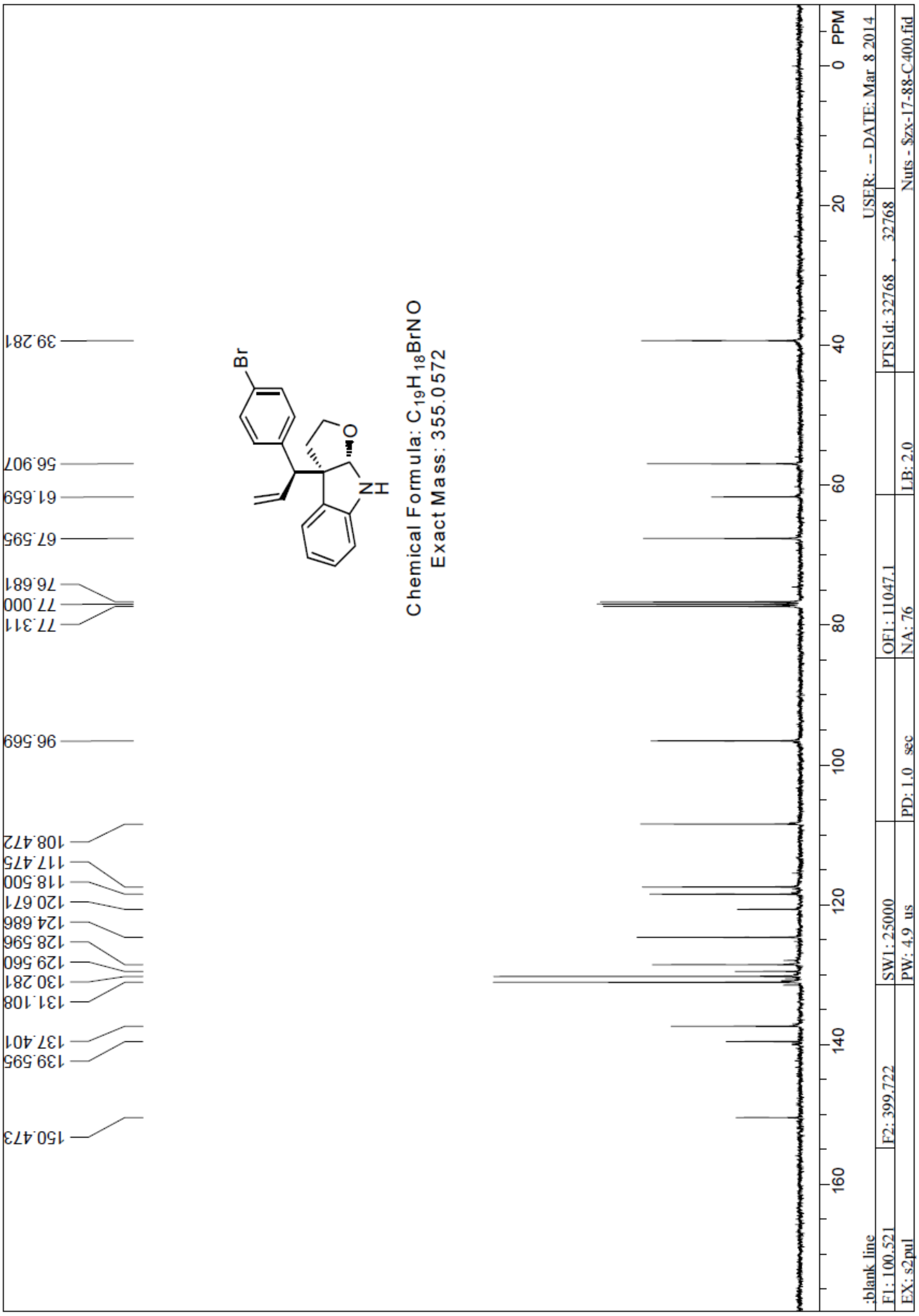

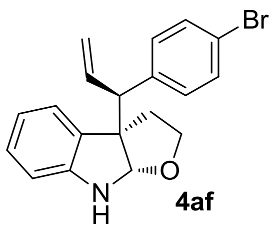

4af

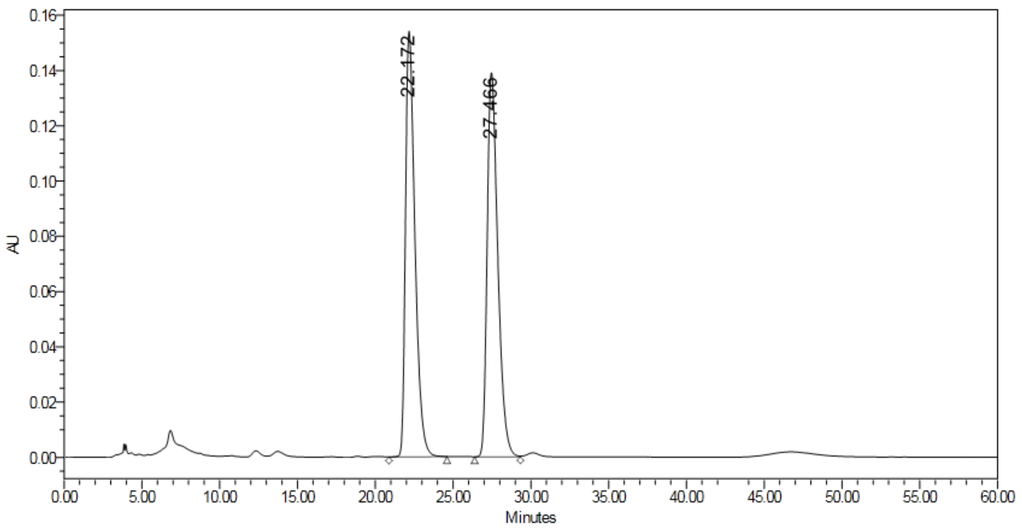

	RT (min)	Area (µV*sec)	% Area	Height (µV)	% Height
1	22.172	6505753	49.67	154117	52.58
2	27.466	6592312	50.33	138975	47.42

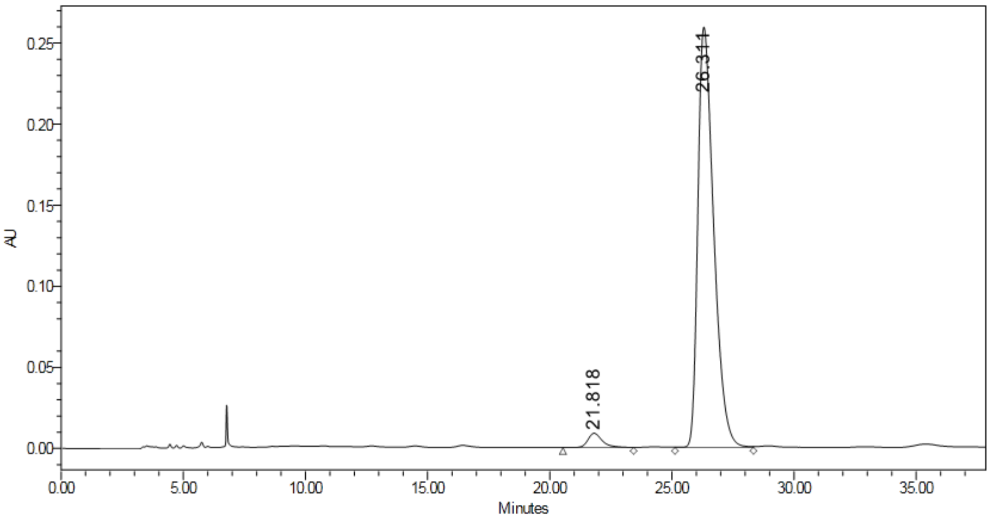

	RT (min)	Area (µV*sec)	% Area	Height (µV)	% Height
1	21.818	358175	2.98	8786	3.28
2	26.311	11676422	97.02	259202	96.72

Compound **4ag**’s ^{1}H NMR Spectra

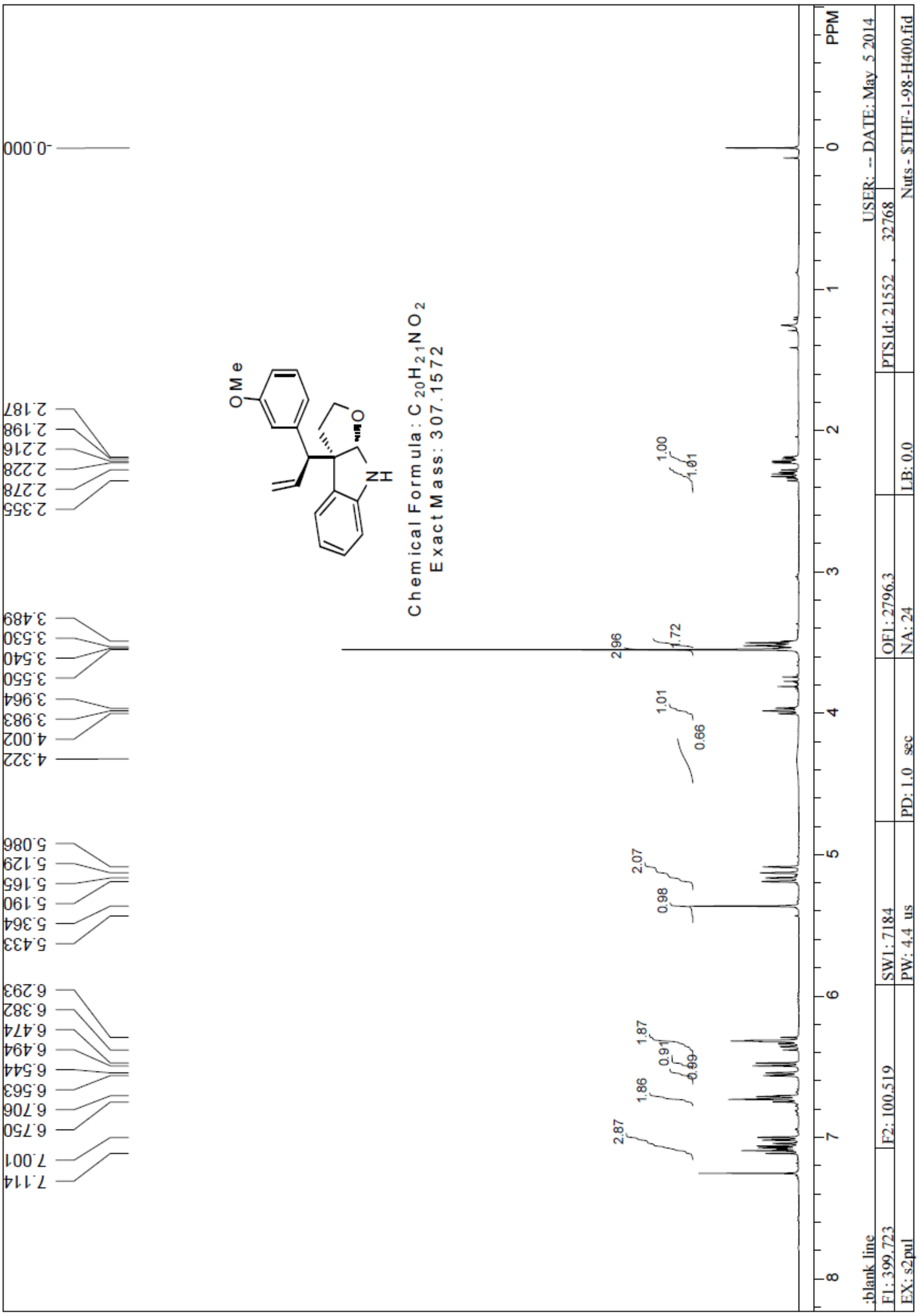

Compound **4ag**'s ^{13}C NMR Spectra

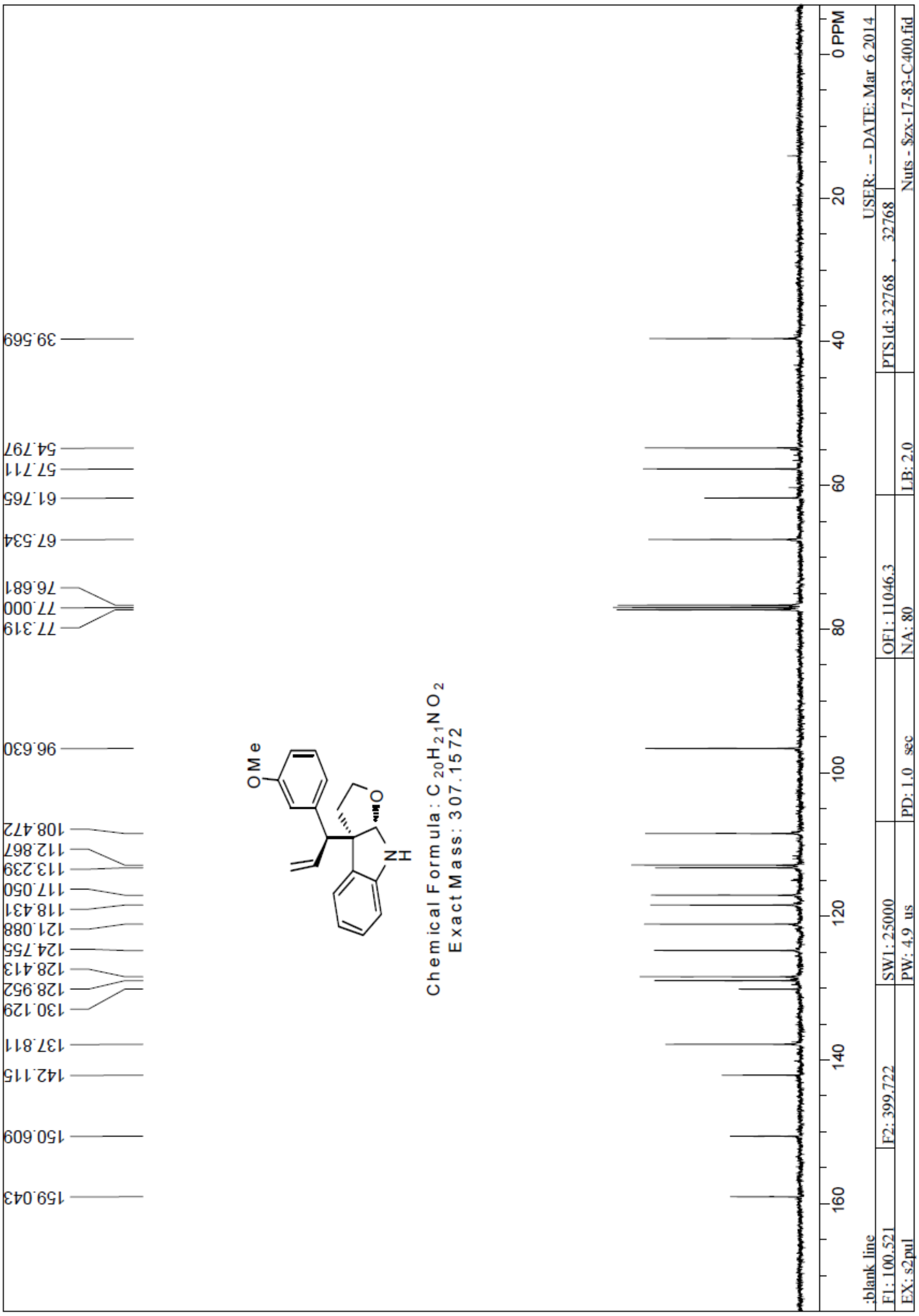

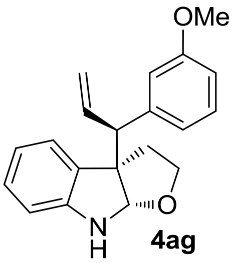

4ag

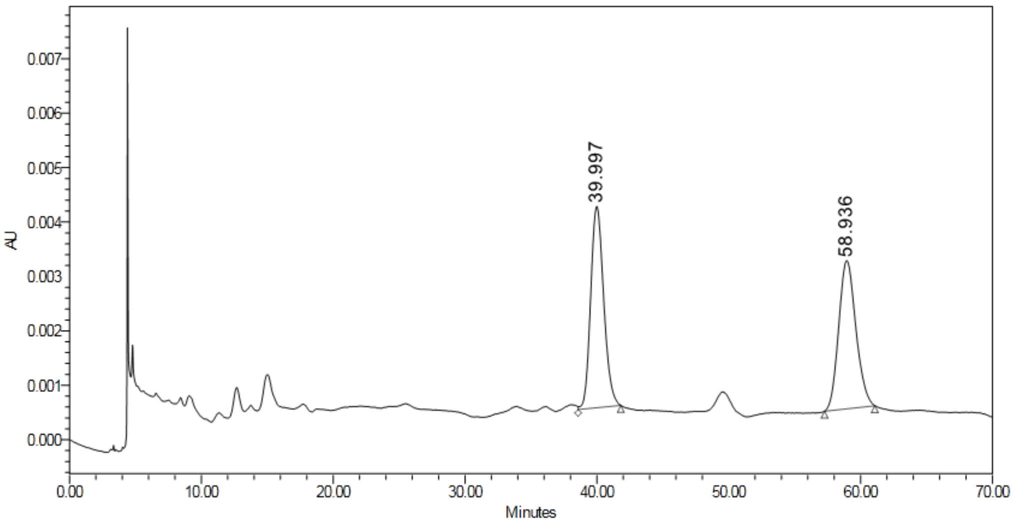

	RT (min)	Area (µV*sec)	% Area	Height (µV)	% Height
1	39.997	254165	50.70	3699	57.59
2	58.936	247190	49.30	2724	42.41

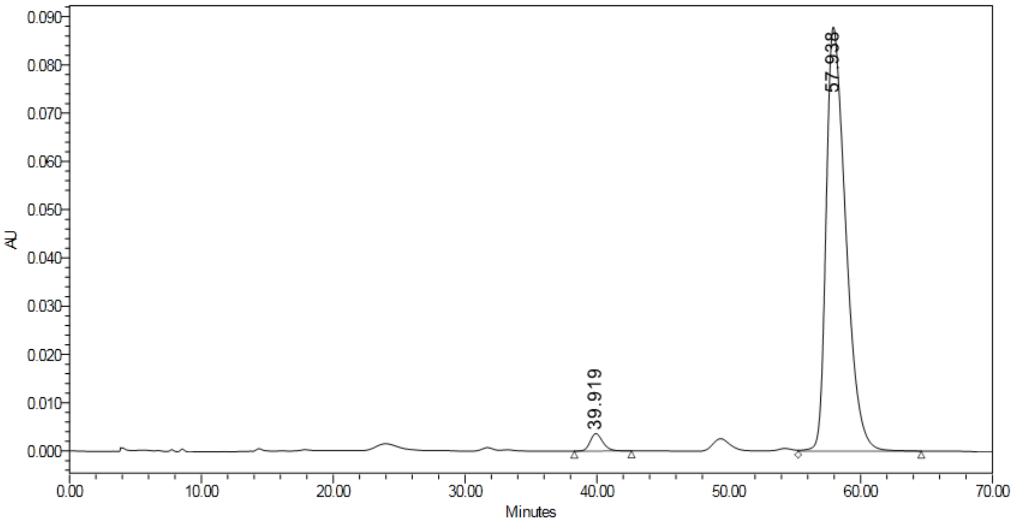

	RT (min)	Area (µV*sec)	% Area	Height (µV)	% Height
1	39.919	243165	2.61	3624	3.96
2	57.938	9072530	97.39	87899	96.04

Compound **4ah**'s ^{1}H NMR Spectra

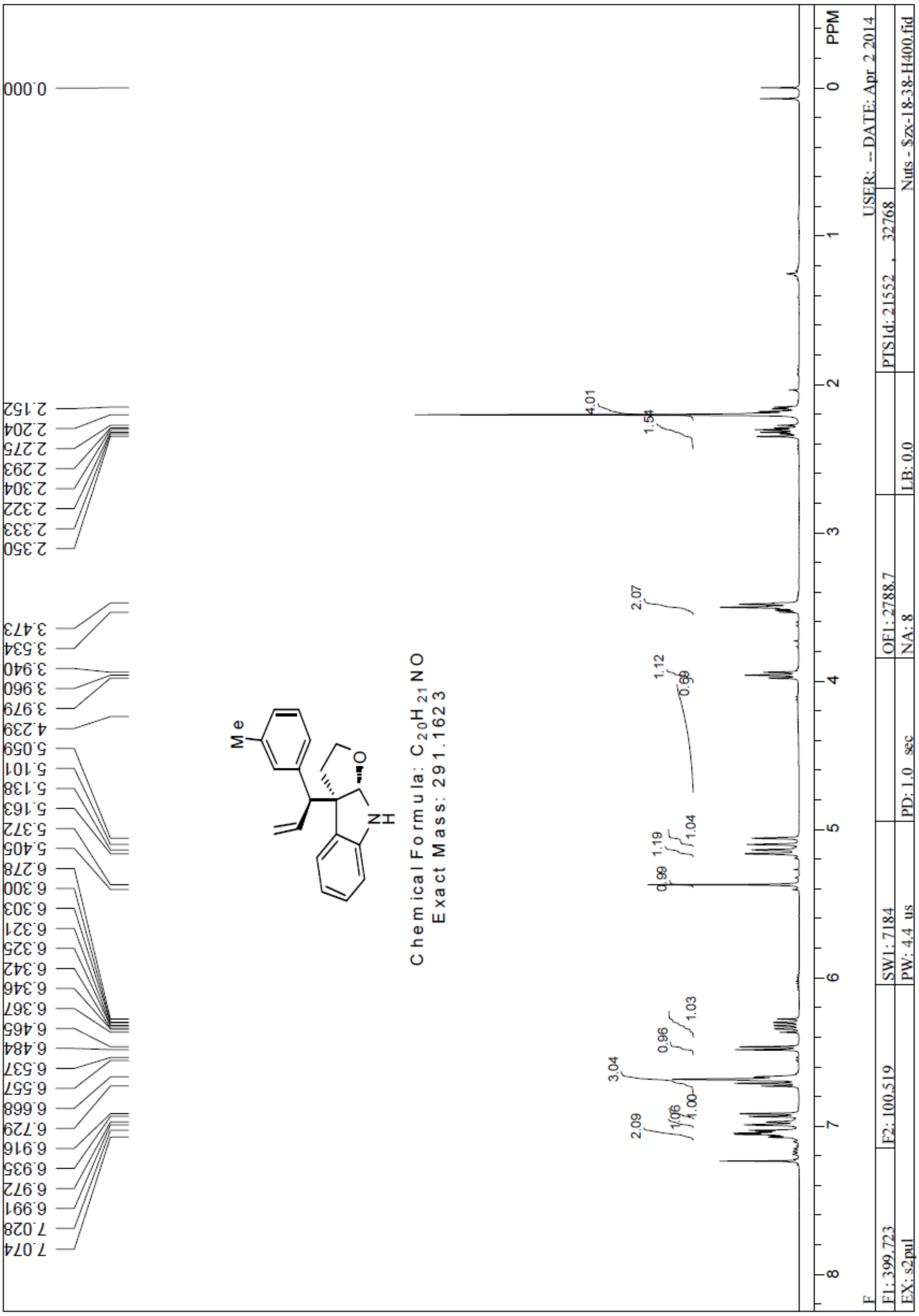

Compound **4ah**’s ^{13}C NMR Spectra

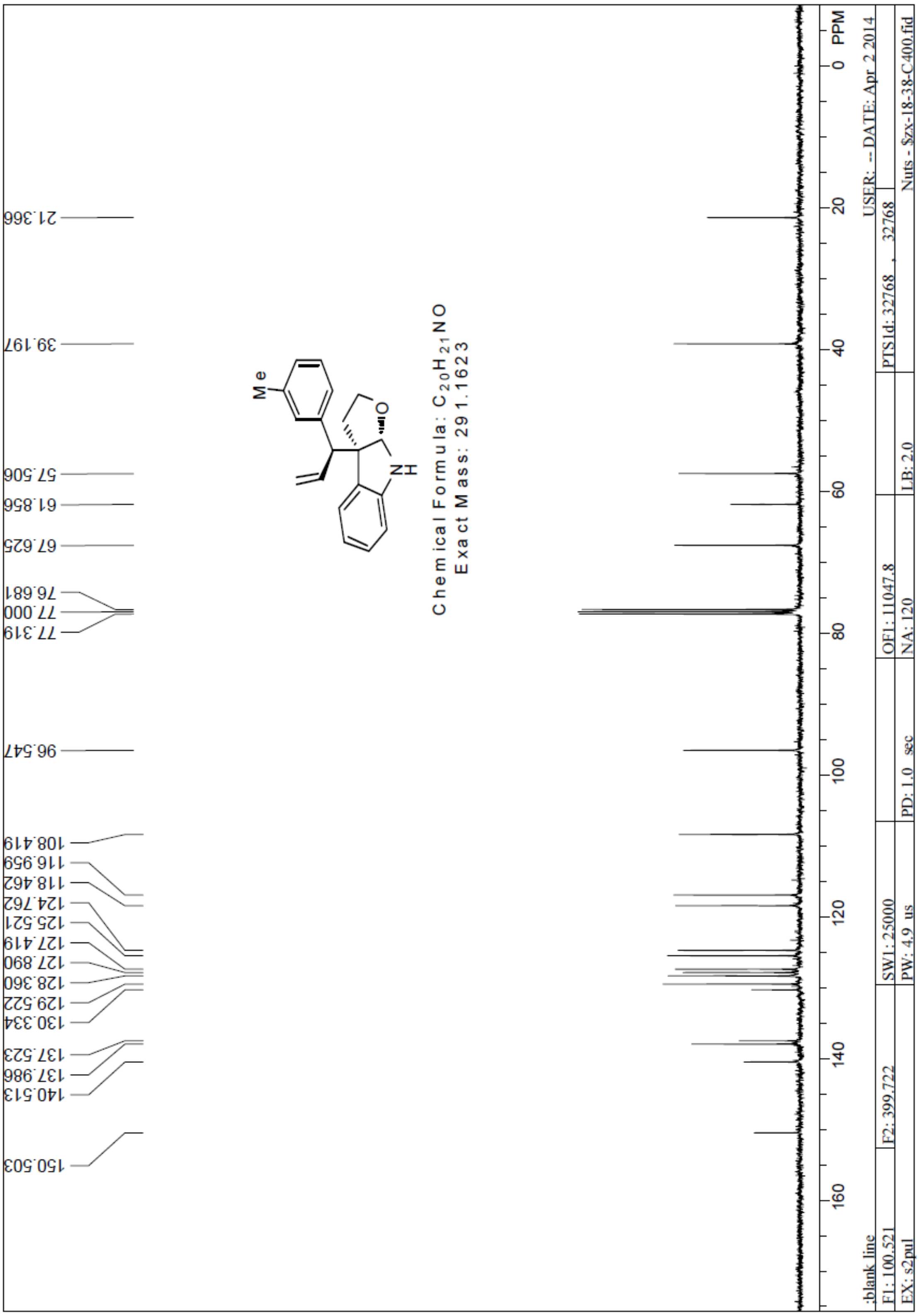

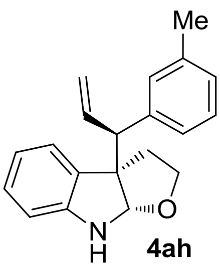

4ah

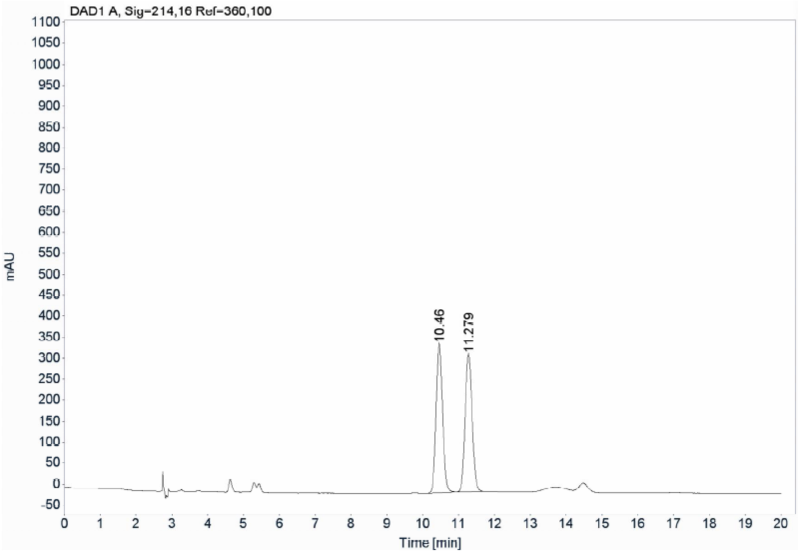

Signal: DAD1 A, Sig=214,16 Ref=360,100

RT [min]	Type	Width [min]	Area	Height	Area%
10.460	BB	0.1989	4502.0894	355.2287	50.1594
11.279	BB	0.2142	4473.4771	327.7354	49.8406
		Sum	8975.5664		

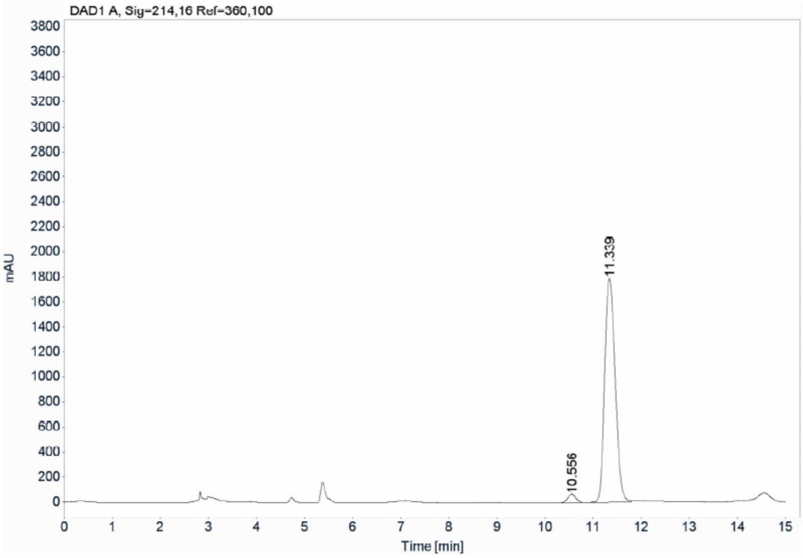

Signal: DAD1 A, Sig=214,16 Ref=360,100

RT [min]	Type	Width [min]	Area	Height	Area%
10.556	BB	0.1928	808.2638	66.0229	2.8743
11.339	BV	0.2422	27312.2109	1788.2399	97.1257
		Sum	28120.4747		

Compound **4ai**'s ^{1}H NMR Spectra

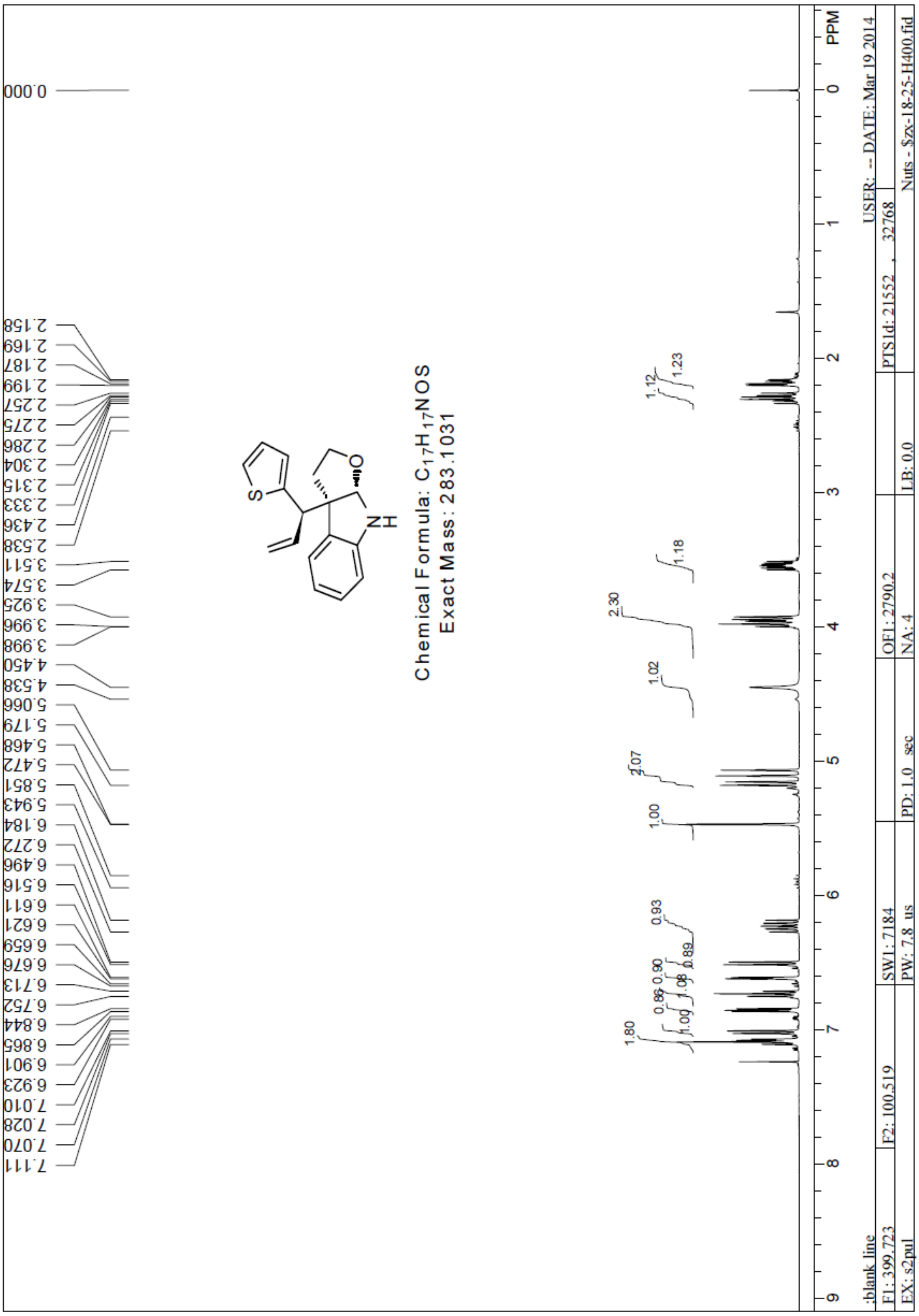

Compound **4ai**'s ^{13}C NMR Spectra

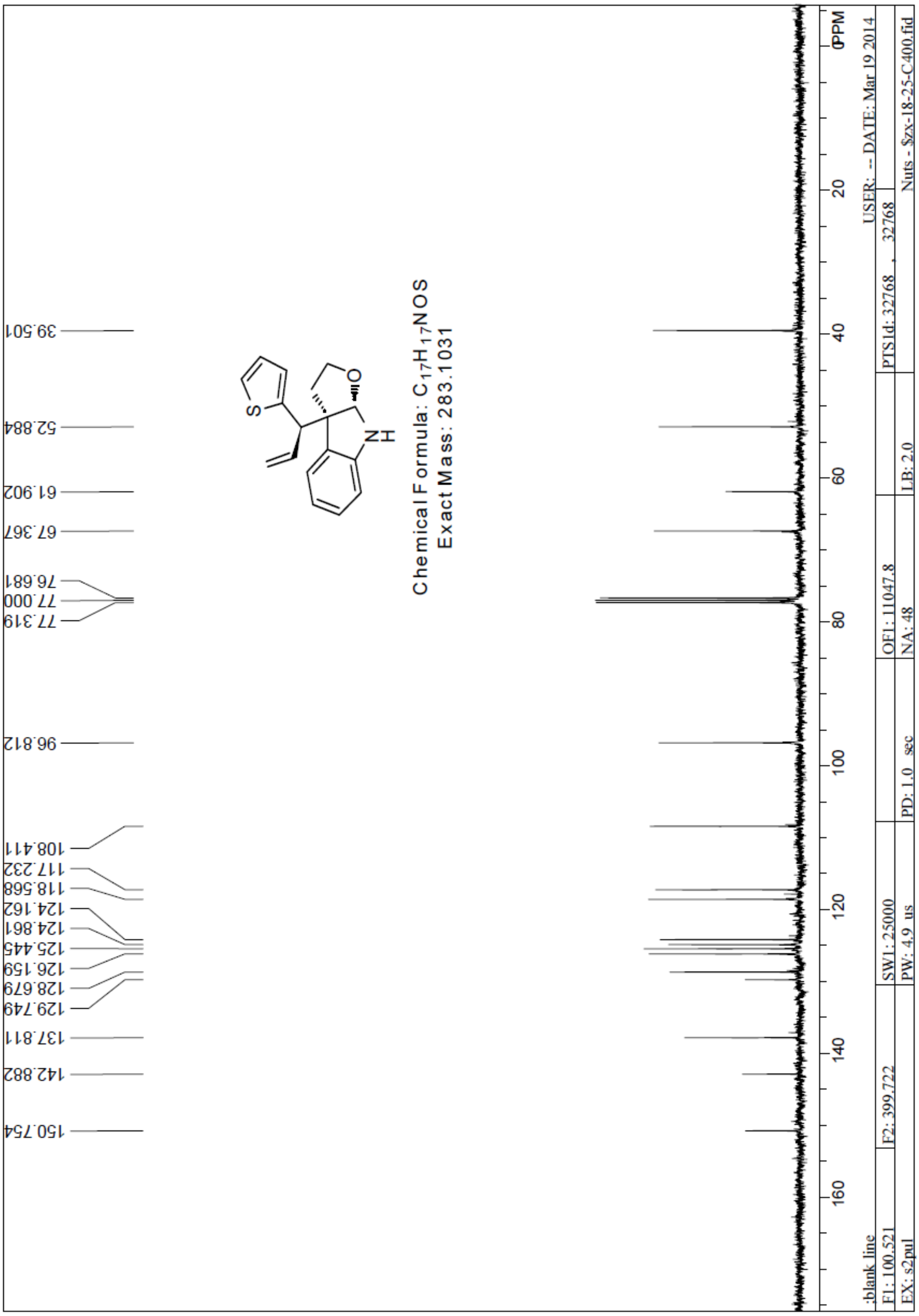

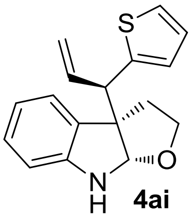

4ai

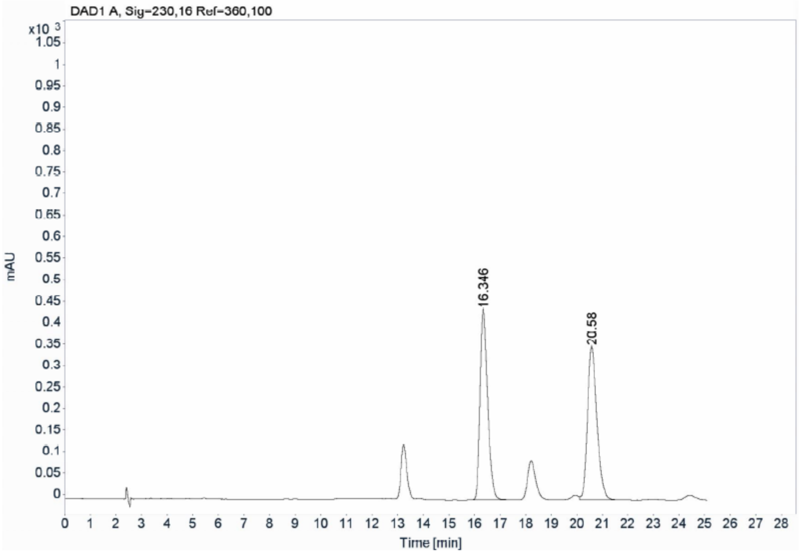

Signal: DAD1 A, Sig=230, 16 Ref=360, 100

RT [min]	Type	Width [min]	Area	Height	Area%
16.346	BB	0.3143	9079.8027	442.7292	49.8705
20.580	VB	0.3899	9126.9639	356.2337	50.1295
		Sum	18206.7666		

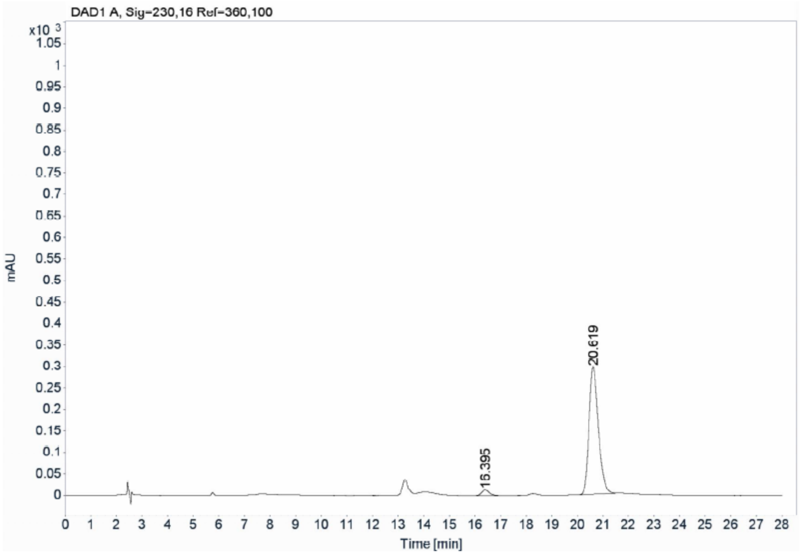

Signal: DAD1 A, Sig=230, 16 Ref=360, 100

RT [min]	Type	Width [min]	Area	Height	Area%
16.395	BB	0.2602	263.4116	13.2341	3.3652
20.619	BB	0.3922	7563.9995	296.9719	96.6348
		Sum	7827.4111		

Compound **4aj**'s ^{1}H NMR Spectra

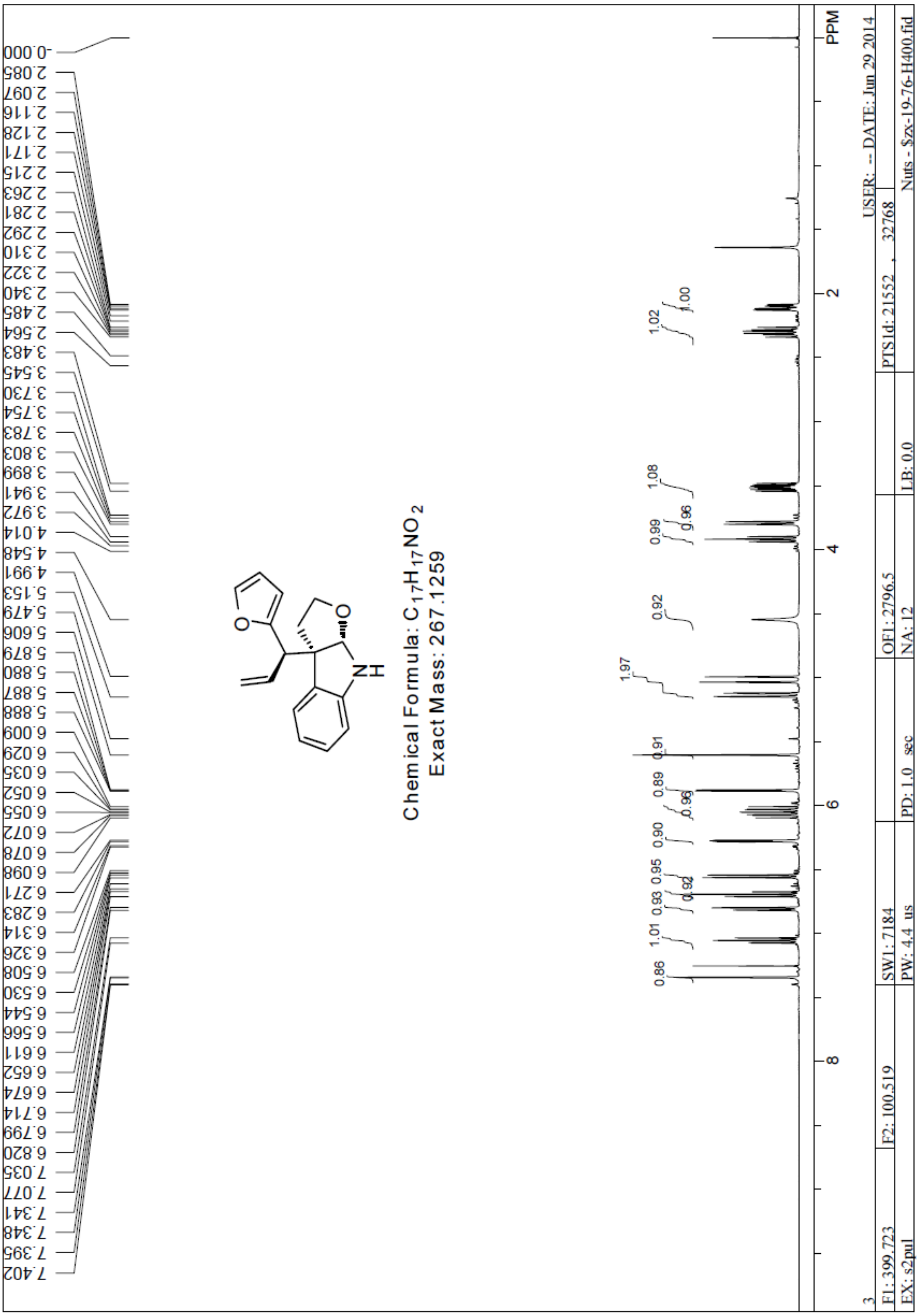

Compound **4aj**'s ^{13}C NMR Spectra

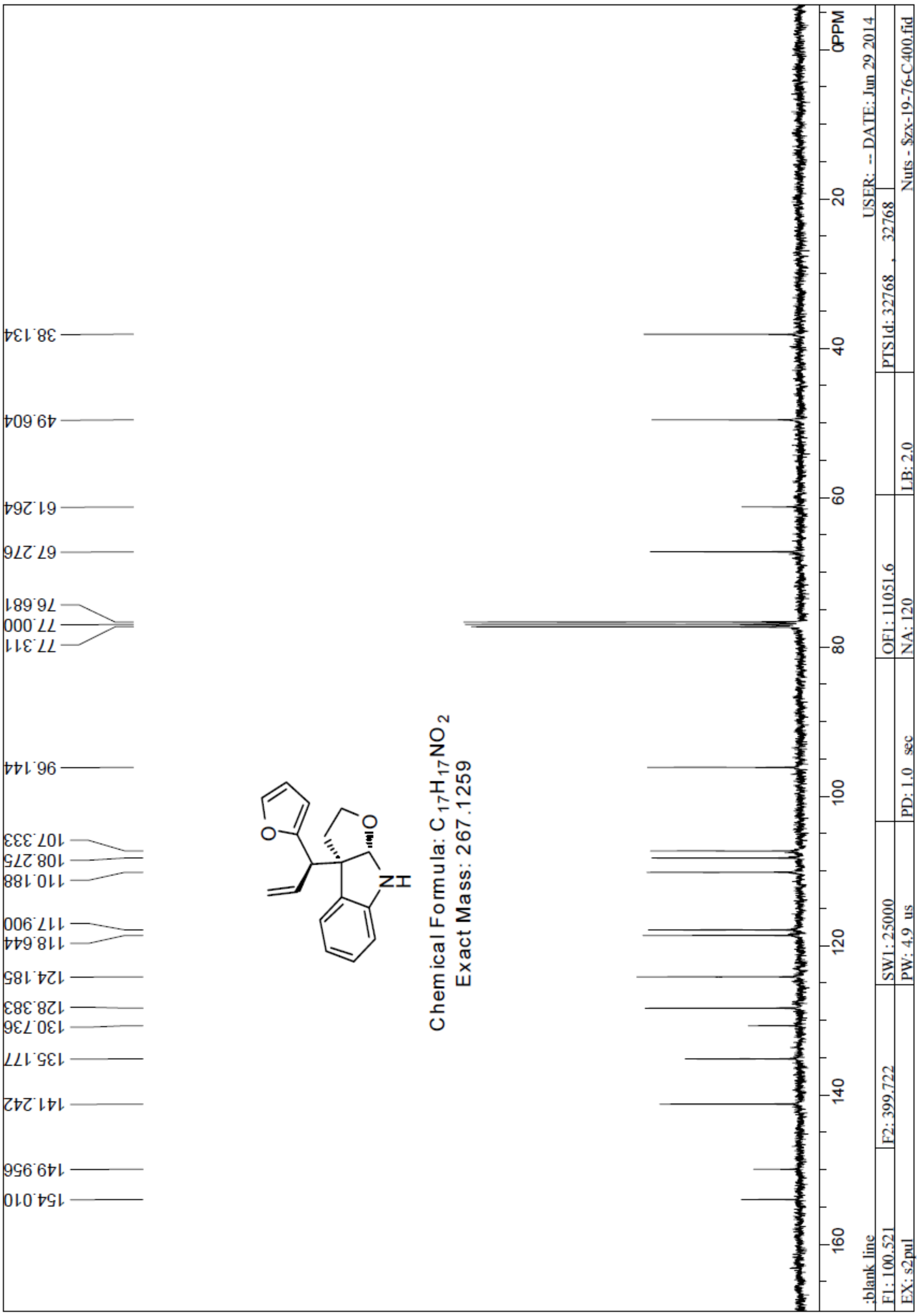

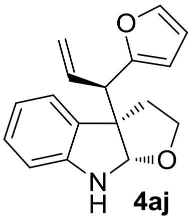

4aj

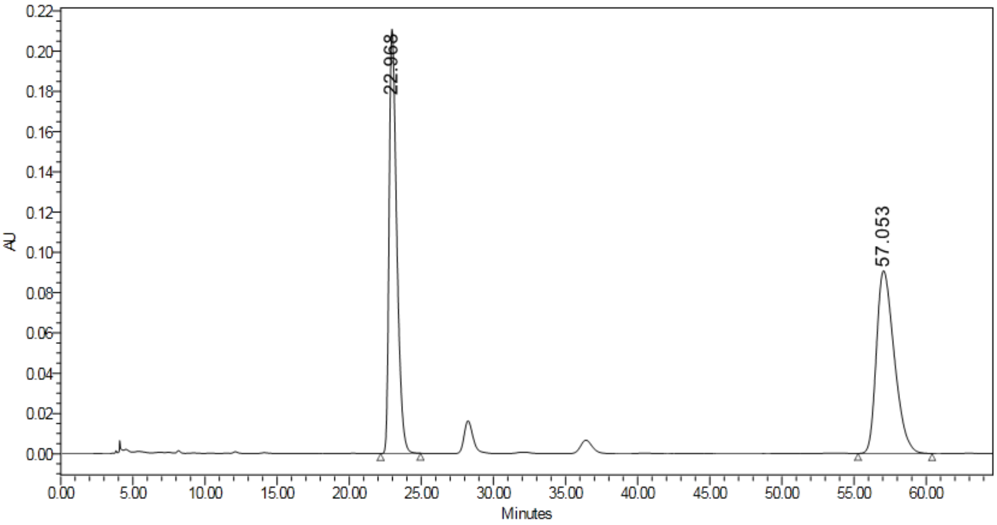

	RT (min)	Area (µV*sec)	% Area	Height (µV)	% Height
1	22.968	7750585	49.73	210893	69.89
2	57.053	7833332	50.27	90849	30.11

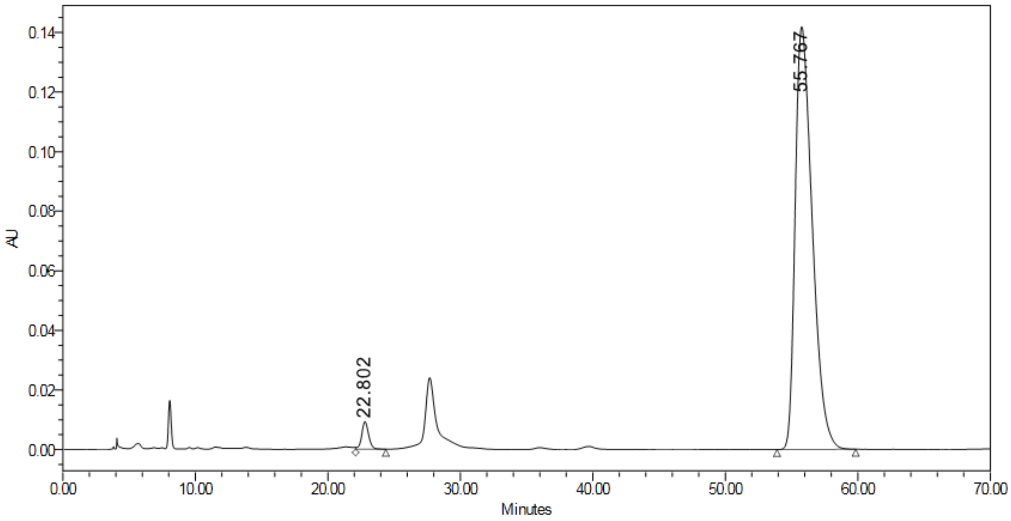

	RT (min)	Area (µV*sec)	% Area	Height (µV)	% Height
1	22.802	331890	2.54	9217	6.10
2	55.767	12734040	97.46	141930	93.90

Compound **4ak**’s ^{1}H NMR Spectra

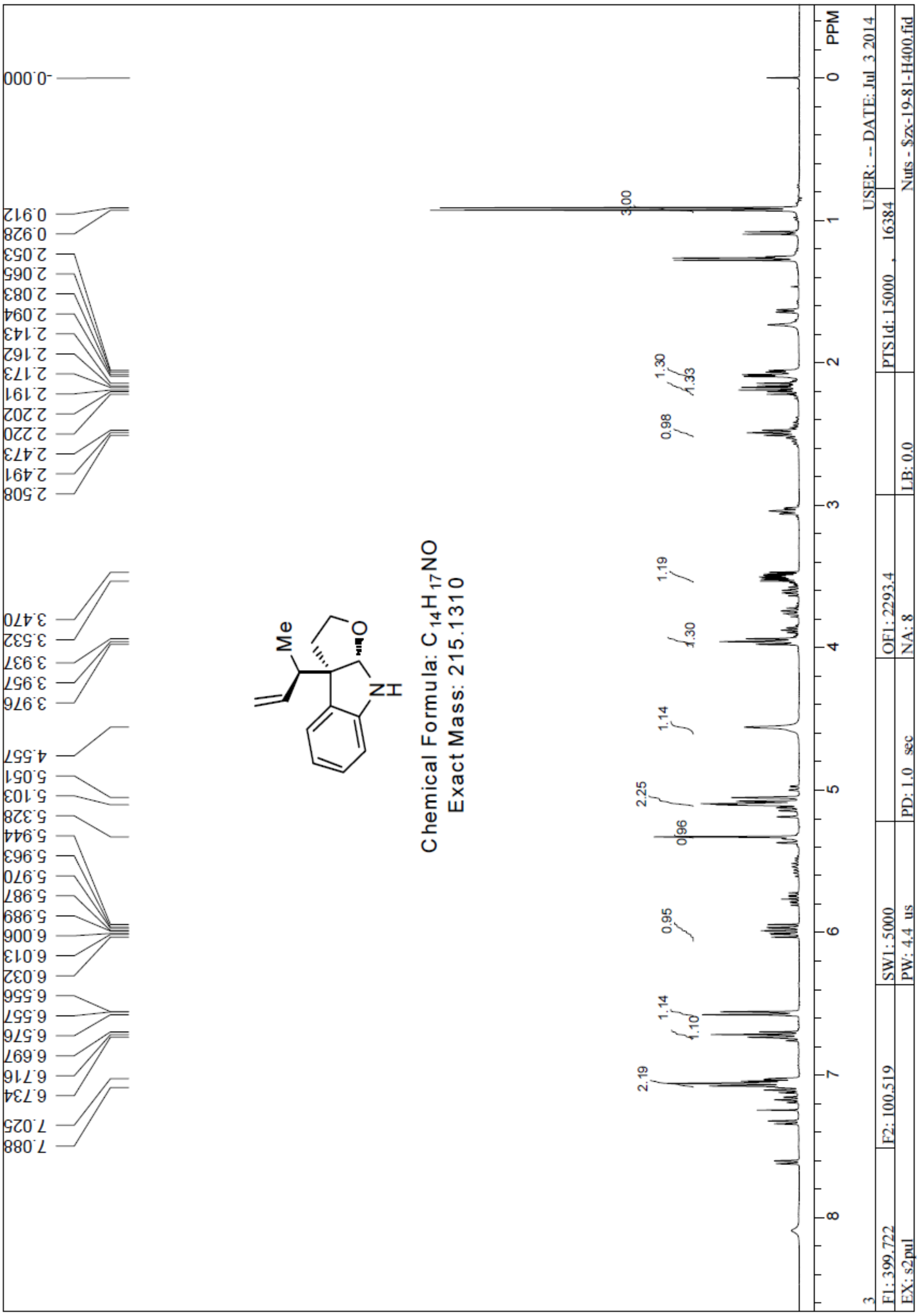

Compound **4ak**'s ^{13}C NMR Spectra

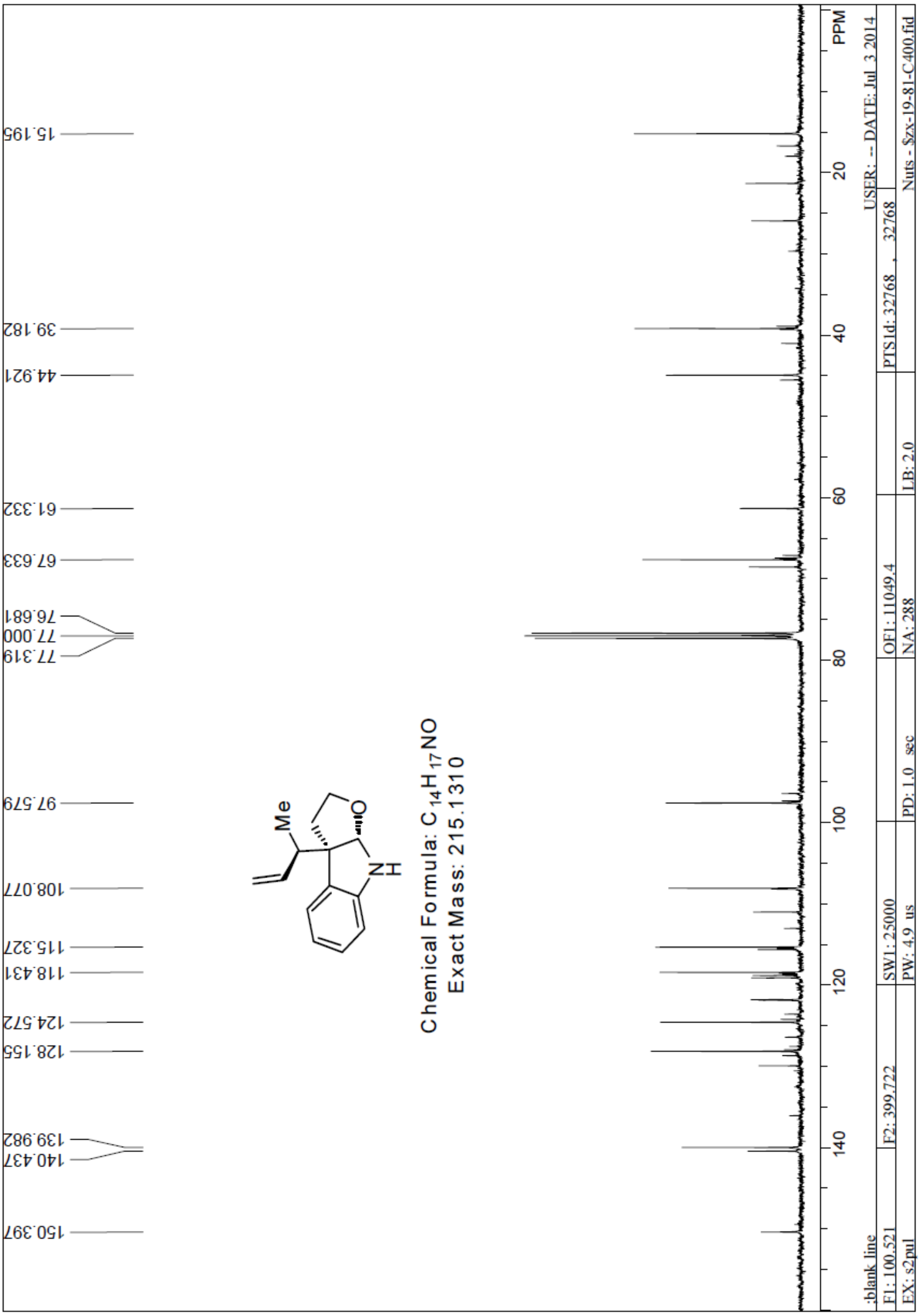

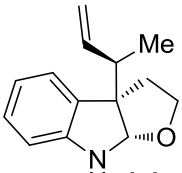

4ak

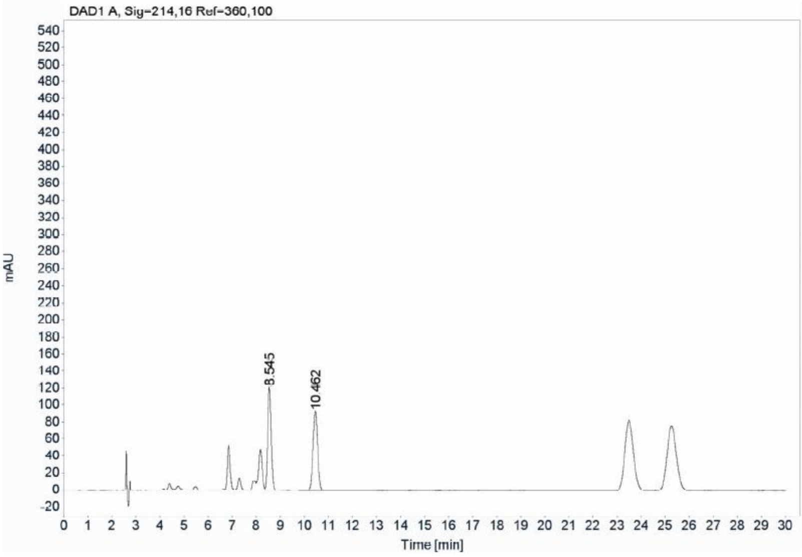

Signal: DAD1 A, Sig=214,16 Ref=360,100

RT [min]	Type	Width [min]	Area	Height	Area%
8.545	VB	0.1532	1188.3176	120.3872	49.5048
10.462	BB	0.2032	1212.0892	92.3072	50.4952
		Sum	2400.4069		

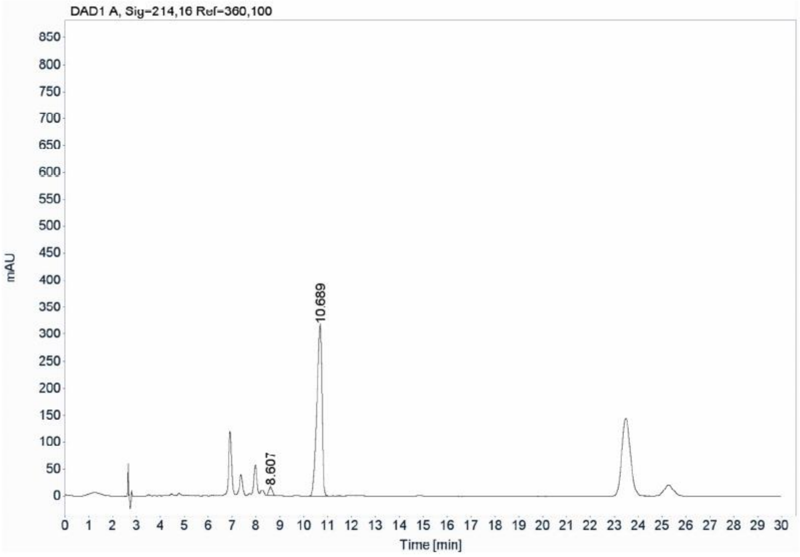

Signal: DAD1 A, Sig=214,16 Ref=360,100

RT [min]	Type	Width [min]	Area	Height	Area%
8.607	BB	0.1432	159.0965	16.9912	3.2793
10.689	BB	0.2222	4692.3721	316.1453	96.7207
		Sum	4851.4686		

Compound **4ba**'s ^{1}H NMR Spectra

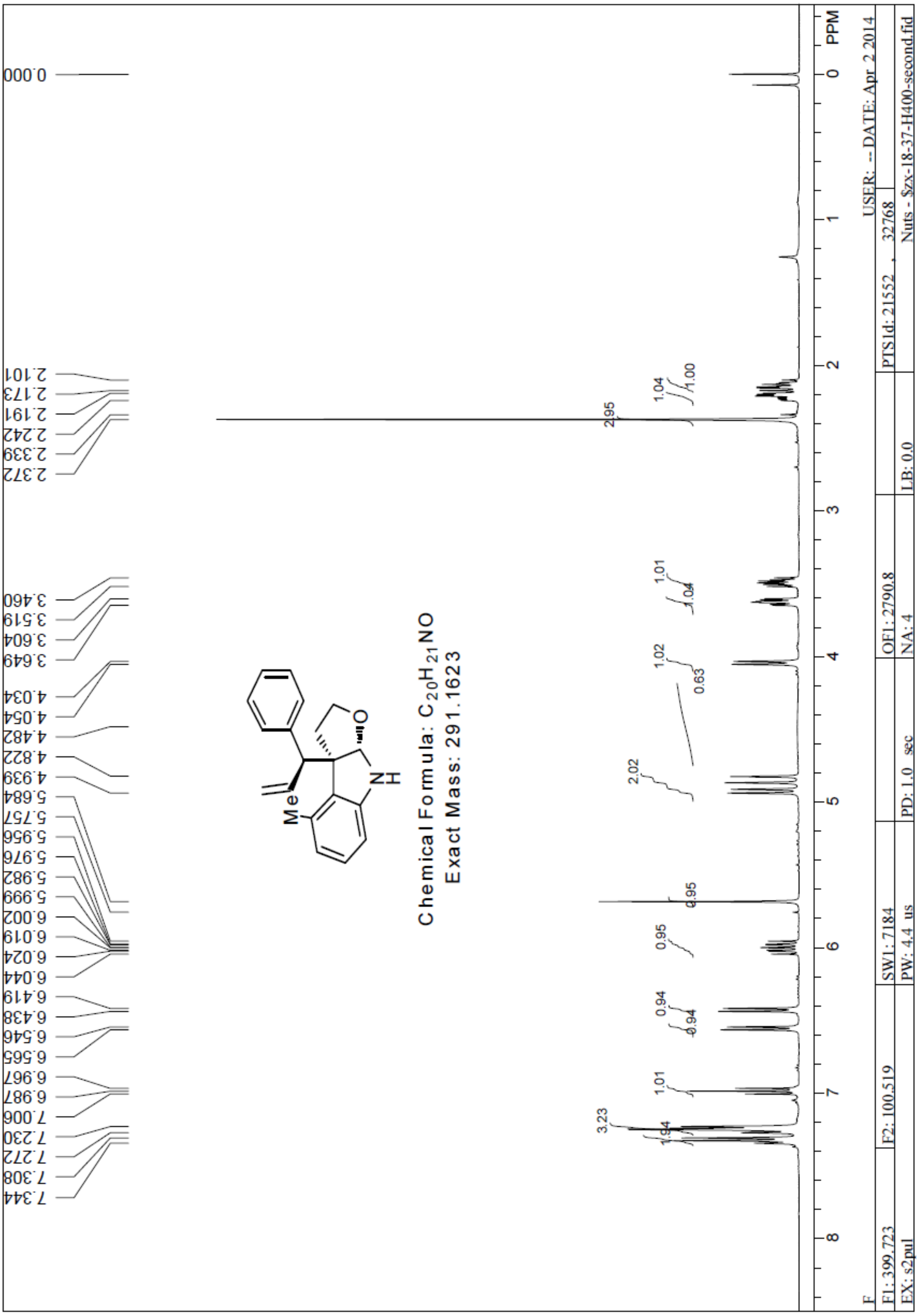

Compound **4ba**’s ^{13}C NMR Spectra

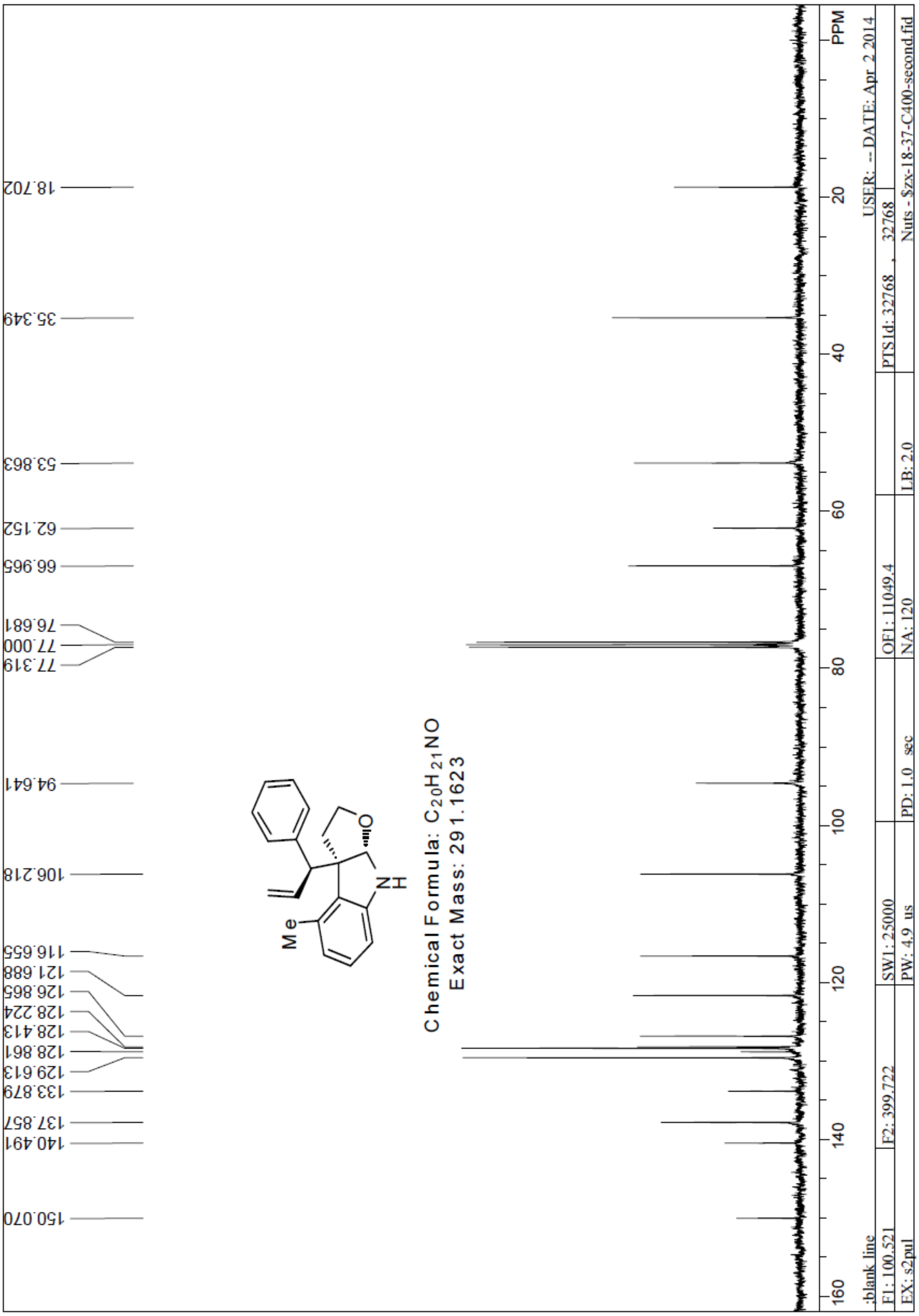

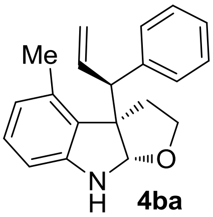

4ba

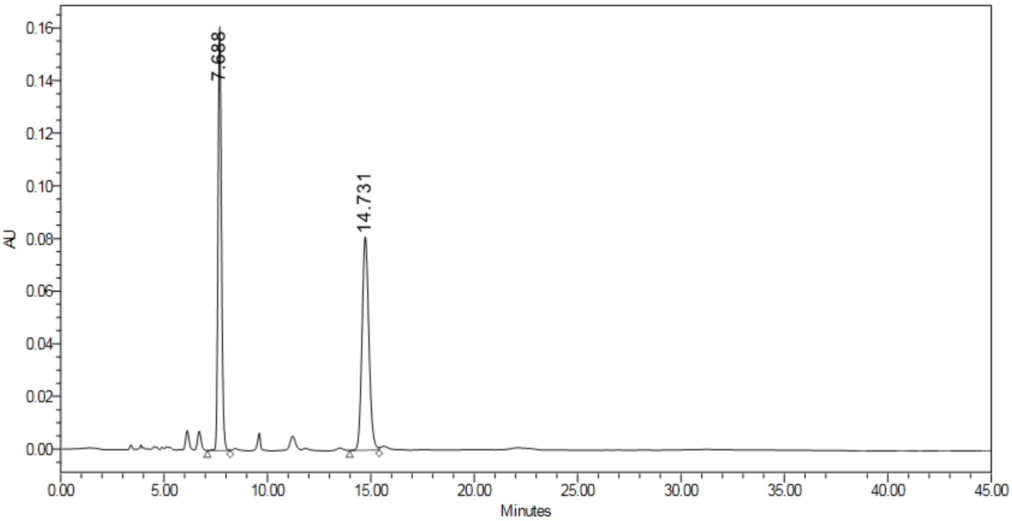

	RT (min)	Area (µV*sec)	% Area	Height (µV)	% Height
1	7.688	1842495	49.74	161040	66.53
2	14.731	1861528	50.26	81002	33.47

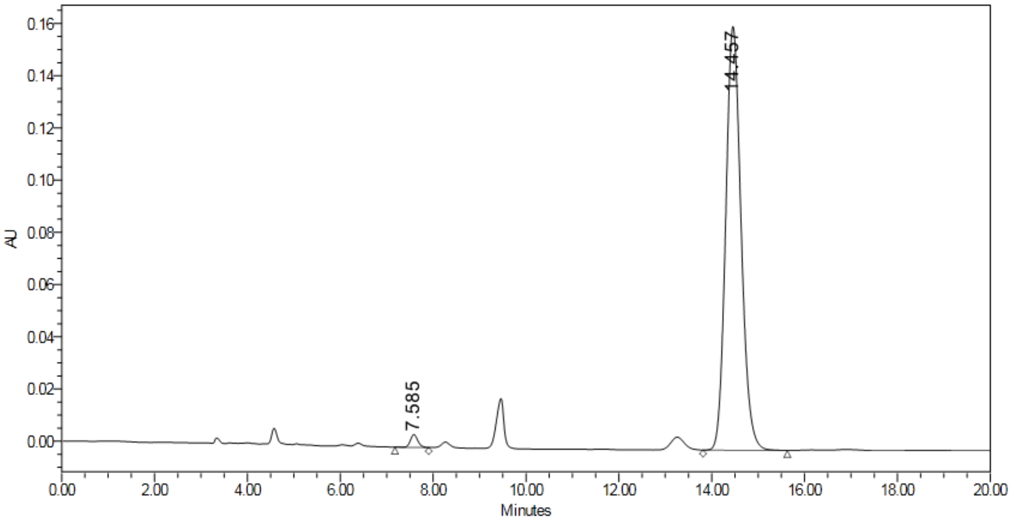

	RT (min)	Area (µV*sec)	% Area	Height (µV)	% Height
1	7.585	56049	1.48	4946	2.96
2	14.457	3728139	98.52	162303	97.04

Compound **4ca**'s ^{1}H NMR Spectra

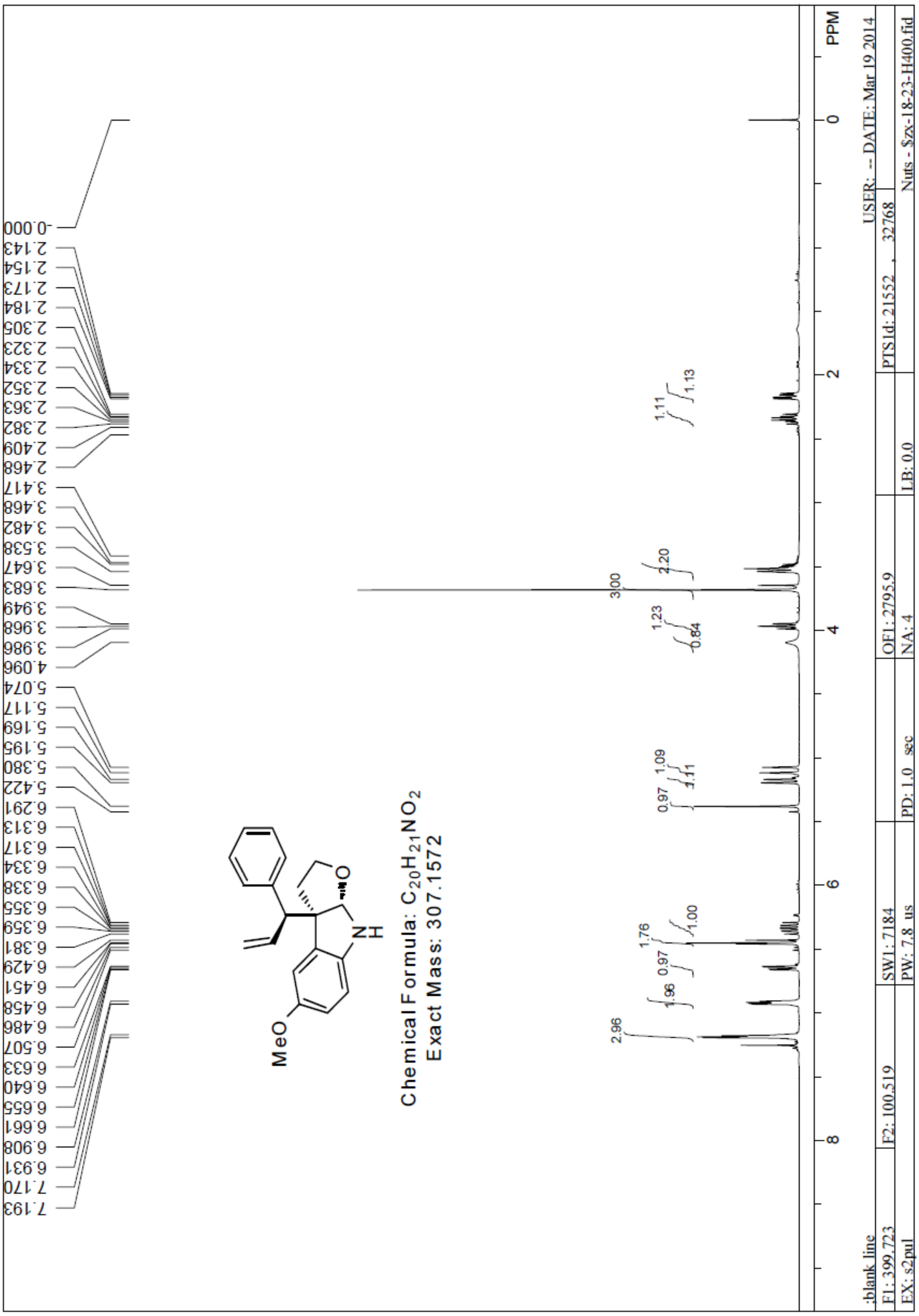

Compound **4ca**’s ^{13}C NMR Spectra

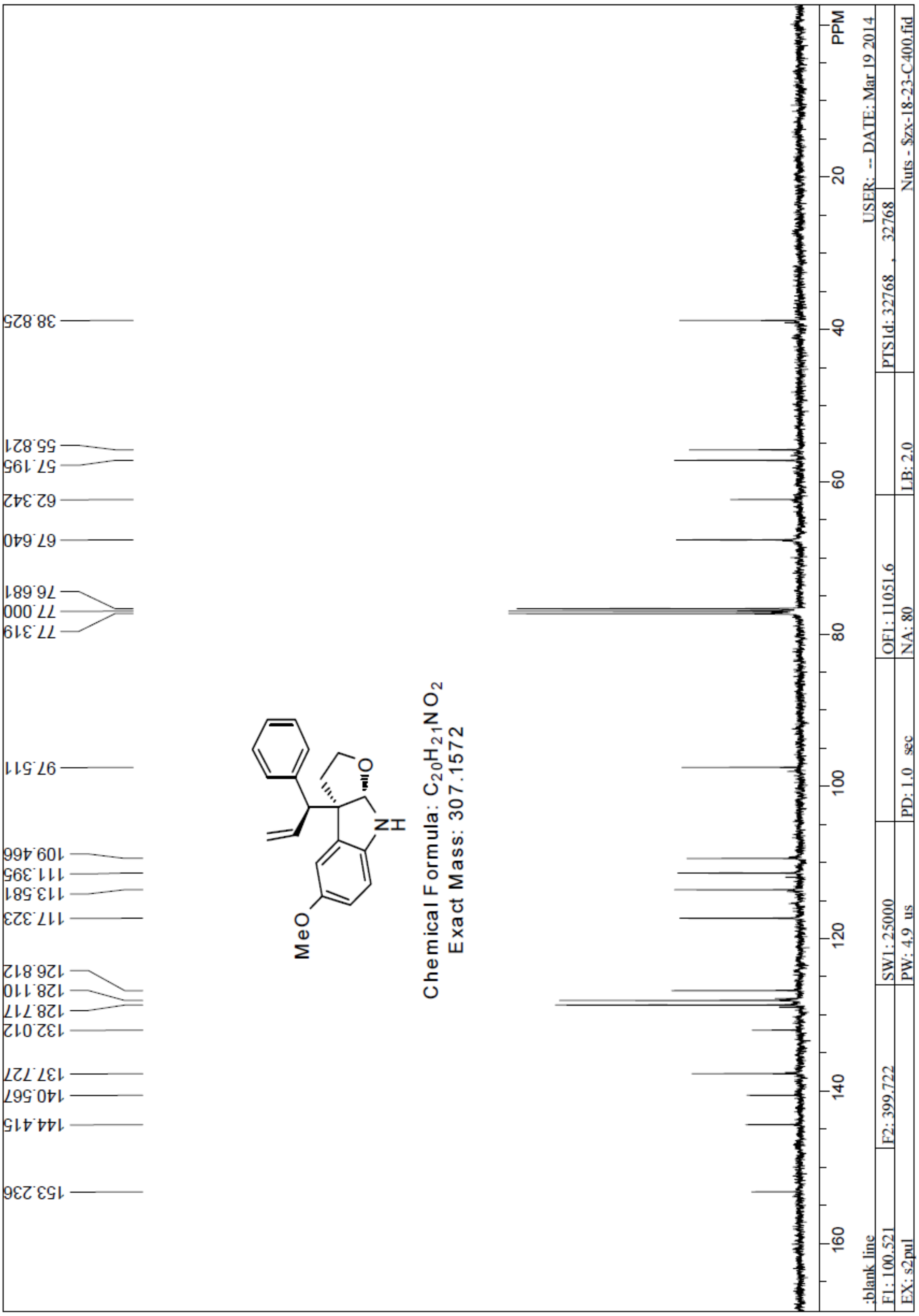

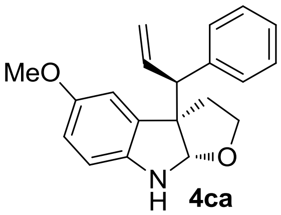

4ca

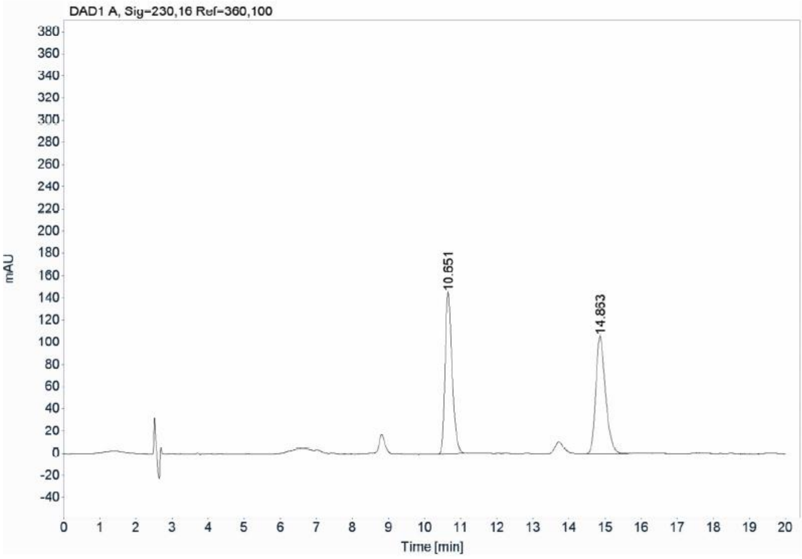

Signal: DAD1 A, Sig=230,16 Ref=360,100

RT [min]	Type	Width [min]	Area	Height	Area%
10.651	BB	0.2041	1949.9290	144.7741	49.8333
14.863	BB	0.2819	1962.9778	105.5728	50.1667
		Sum	3912.9067		

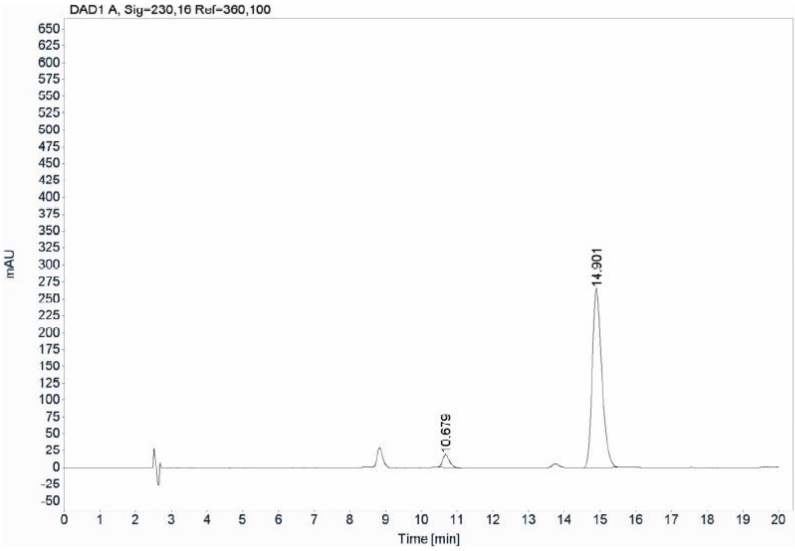

Signal: DAD1 A, Sig=230,16 Ref=360,100

RT [min]	Type	Width [min]	Area	Height	Area%
10.679	BB	0.1976	243.1077	18.2250	4.6779
14.901	BB	0.2819	4953.8779	265.1873	95.3221
		Sum	5196.9856		

Compound **4da**'s ^{1}H NMR Spectra

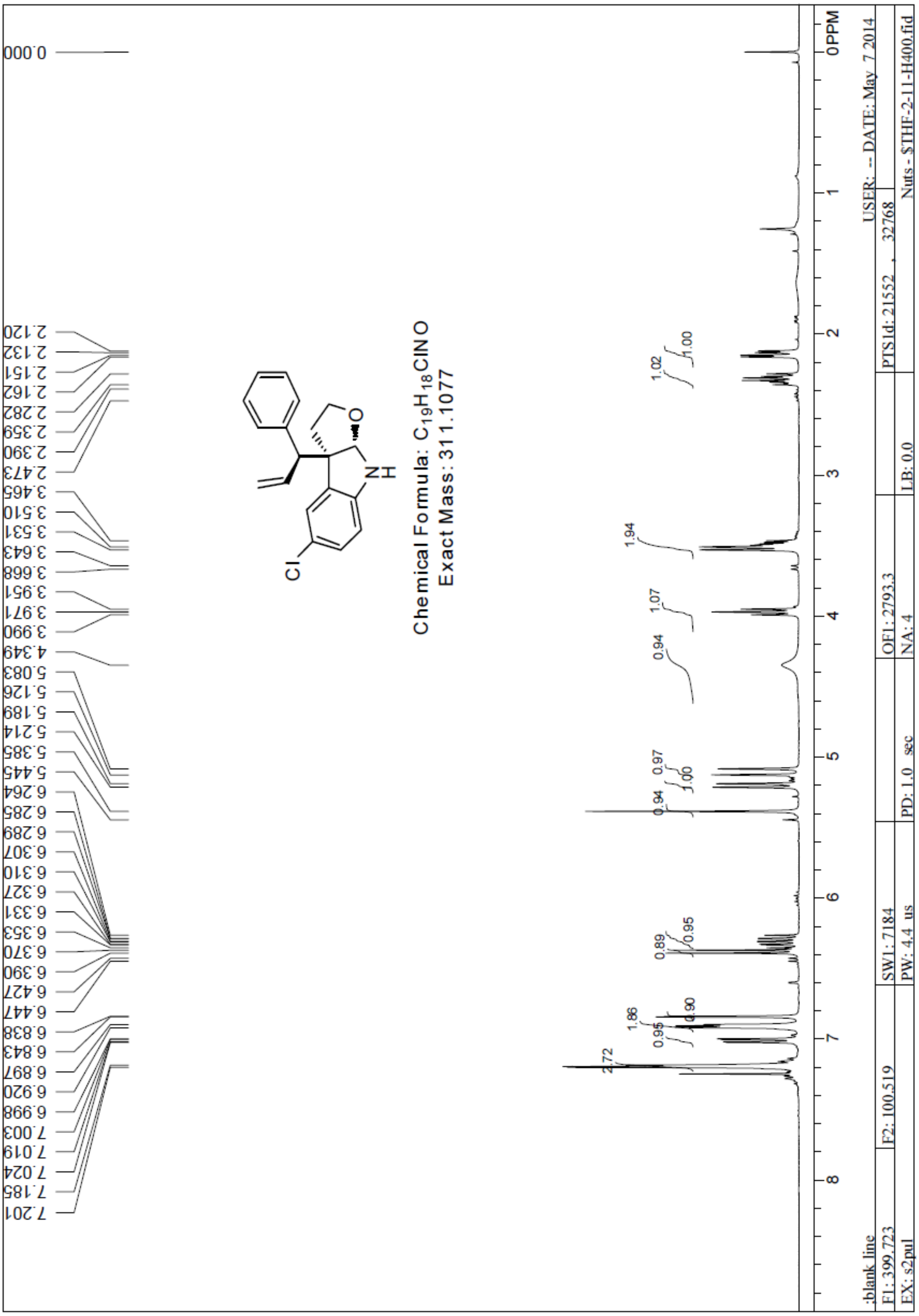

Compound **4da**’s ^{13}C NMR Spectra

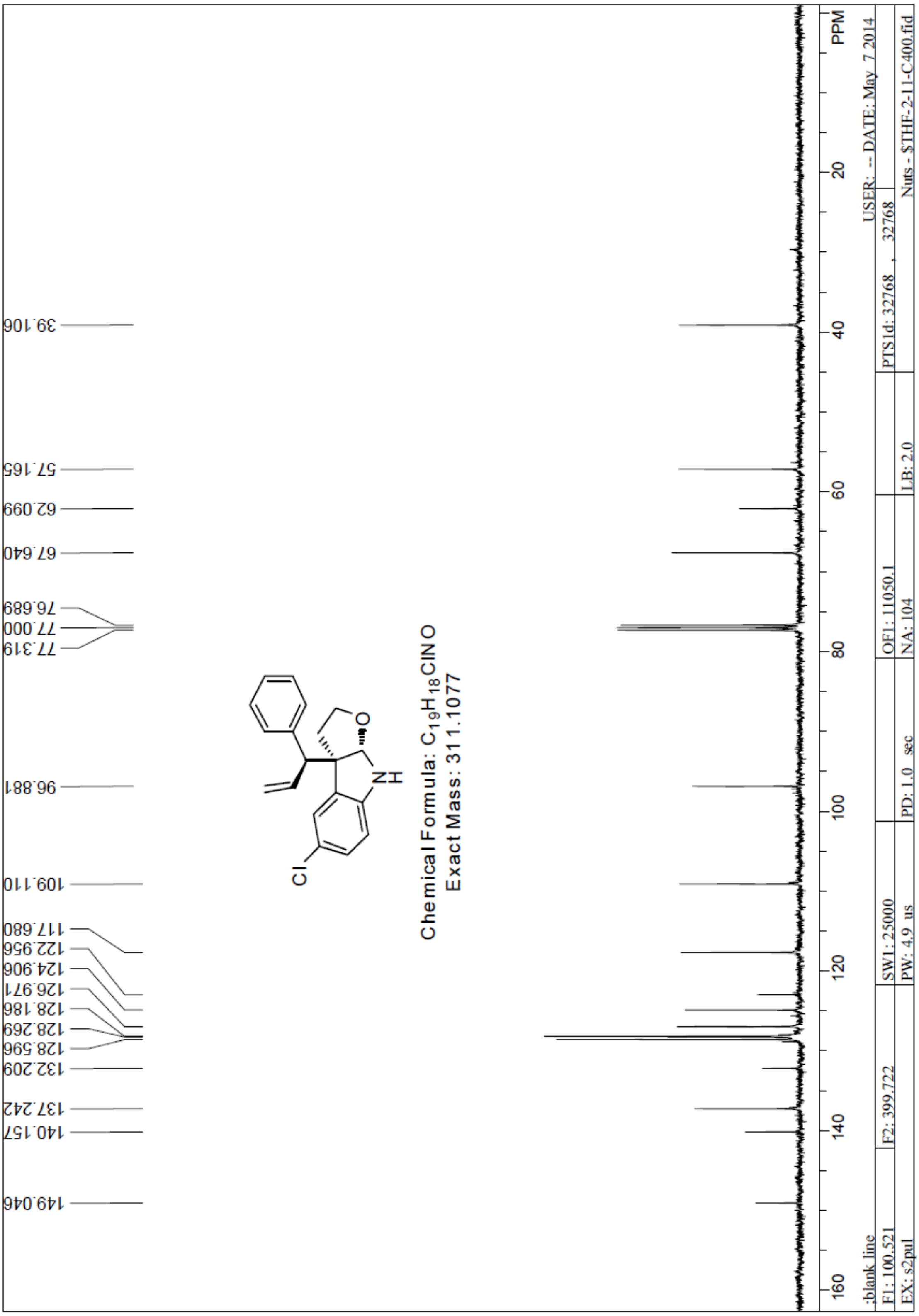

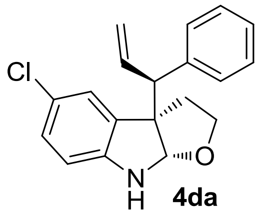

4da

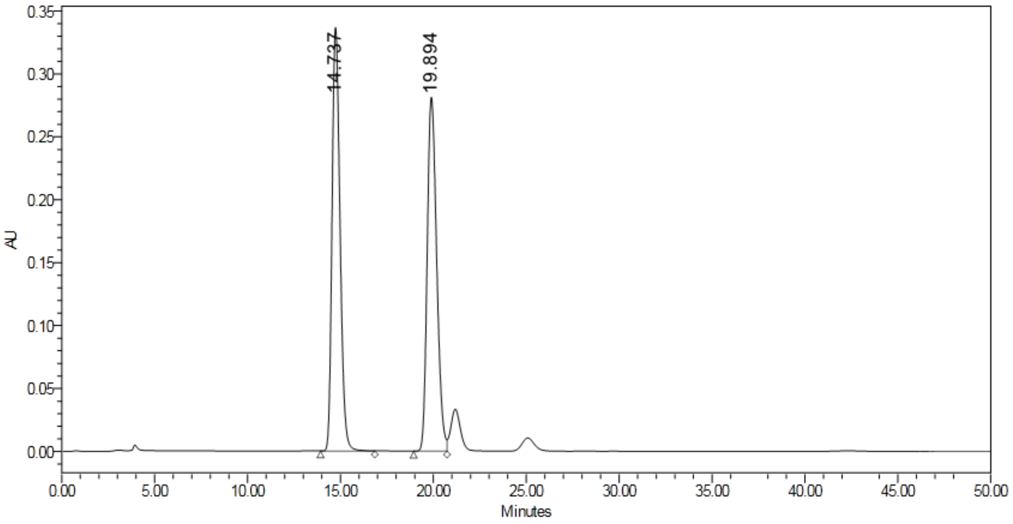

	RT (min)	Area (µV*sec)	% Area	Height (µV)	% Height
1	14.737	10014085	50.06	336639	54.48
2	19.894	9989428	49.94	281275	45.52

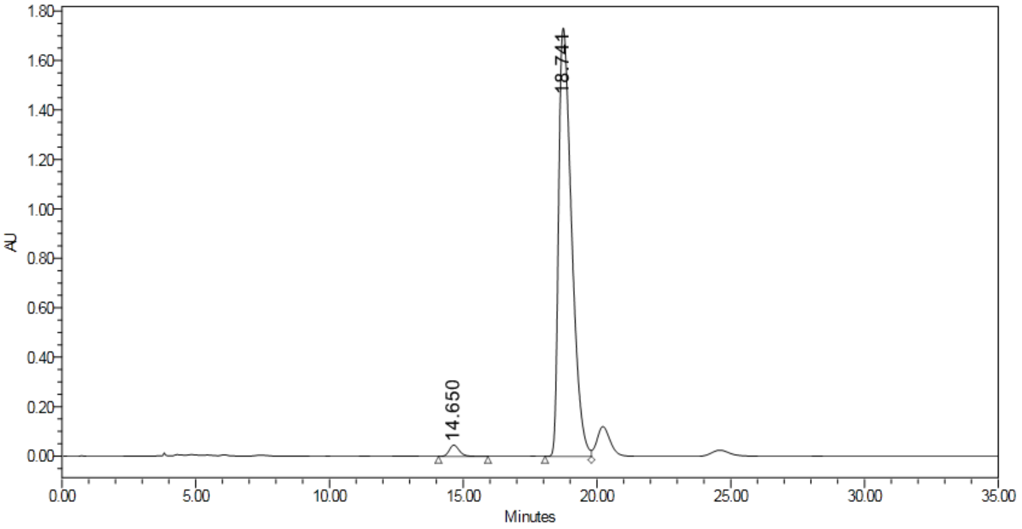

	RT (min)	Area (µV*sec)	% Area	Height (µV)	% Height
1	14.650	1186715	1.99	45626	2.56
2	18.741	58548380	98.01	1733400	97.44

Compound **4ea**’s ^{1}H NMR Spectra

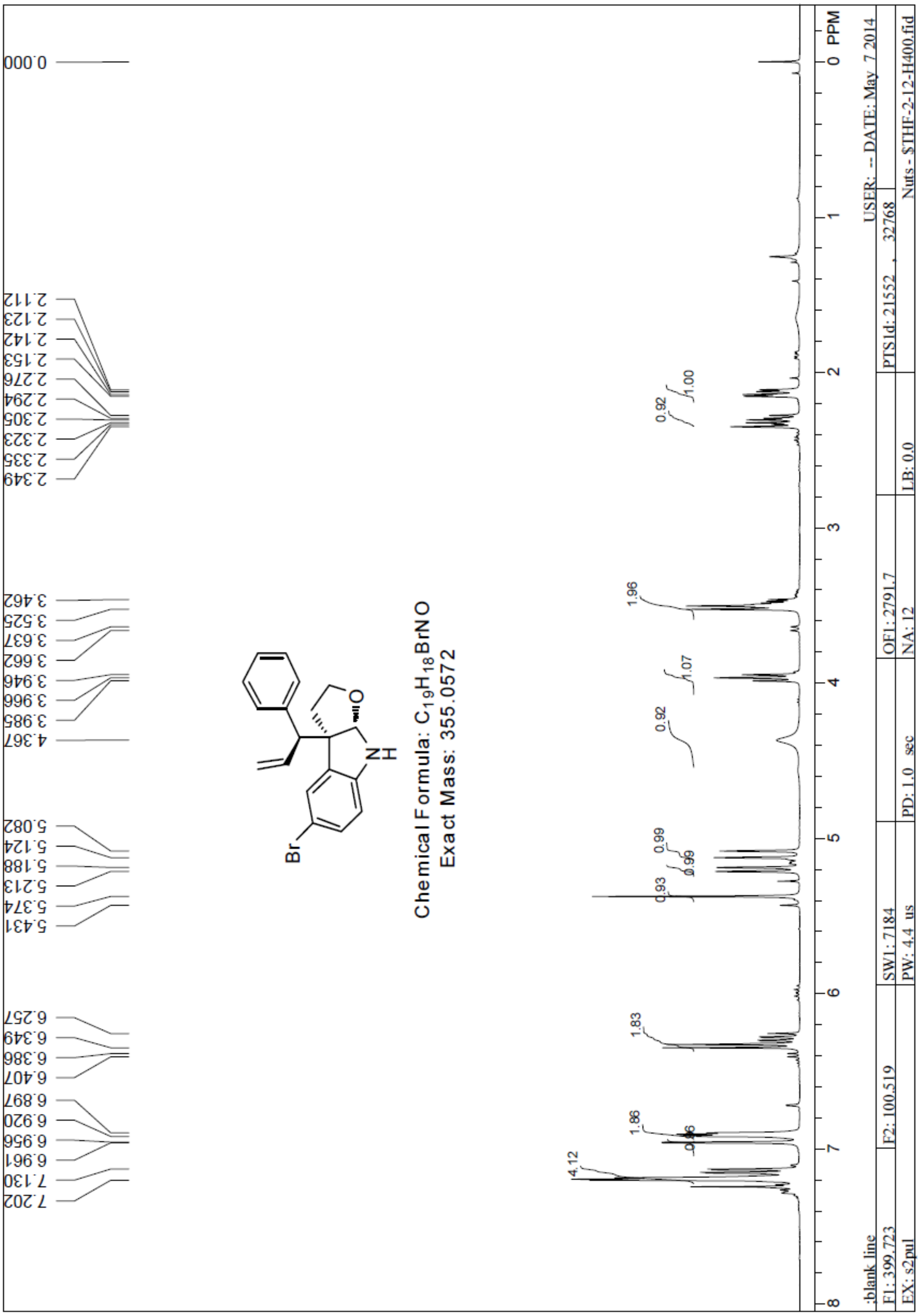

Compound **4ea**'s ^{13}C NMR Spectra

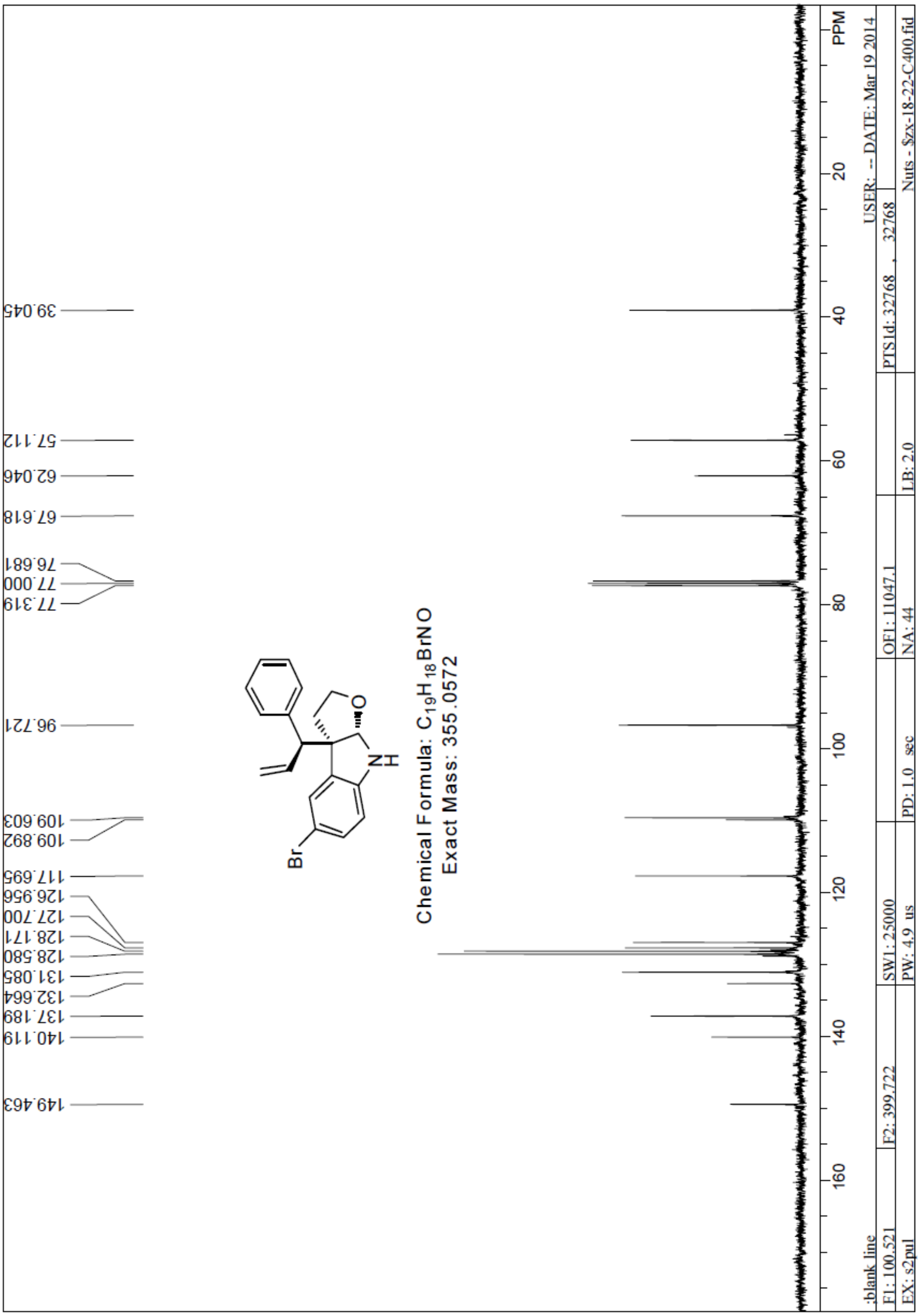

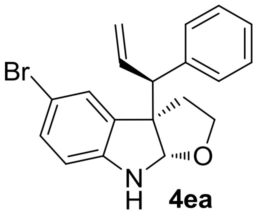

4ea

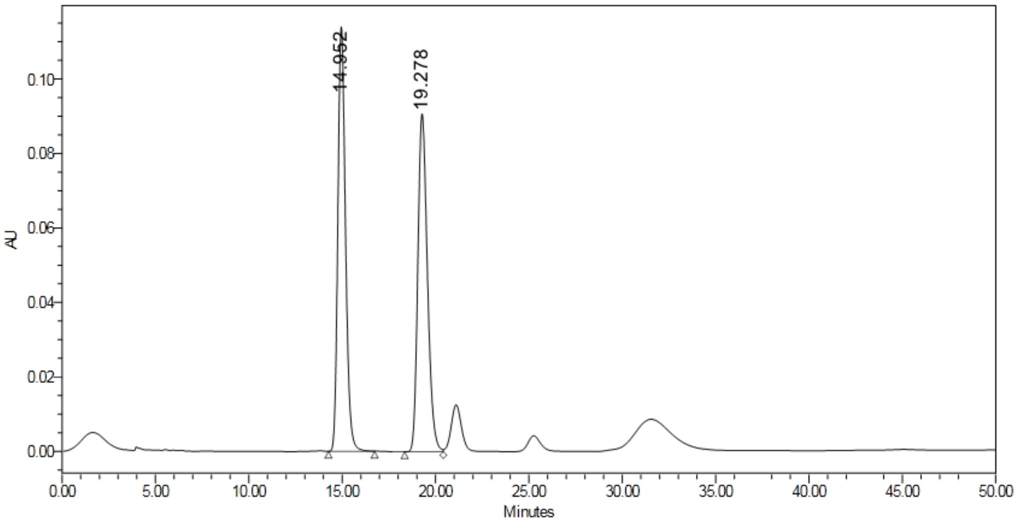

	RT (min)	Area (µV*sec)	% Area	Height (µV)	% Height
1	14.952	3230780	50.01	113974	55.68
2	19.278	3229544	49.99	90723	44.32

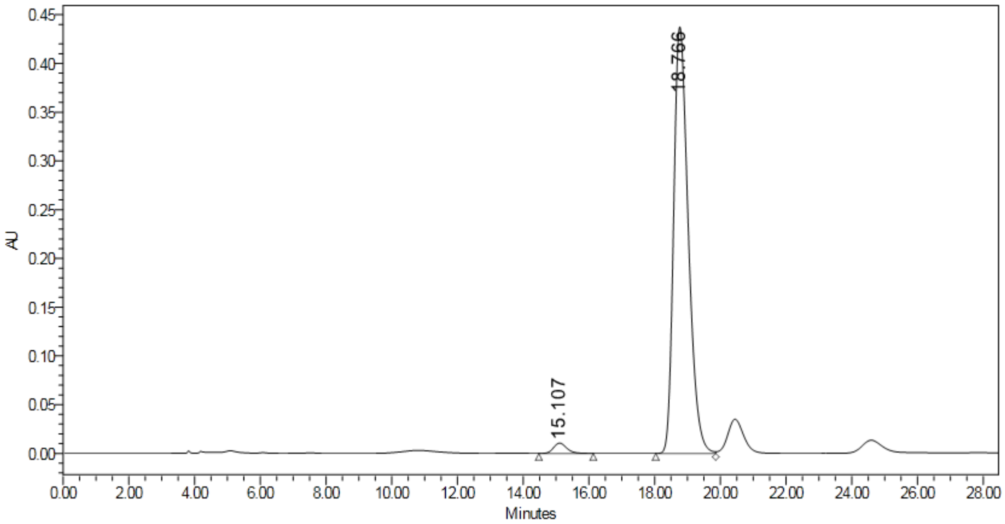

	RT (min)	Area (µV*sec)	% Area	Height (µV)	% Height
1	15.107	293327	2.13	10610	2.37
2	18.766	13503892	97.87	437543	97.63

Compound **4fa**'s ^{1}H NMR Spectra

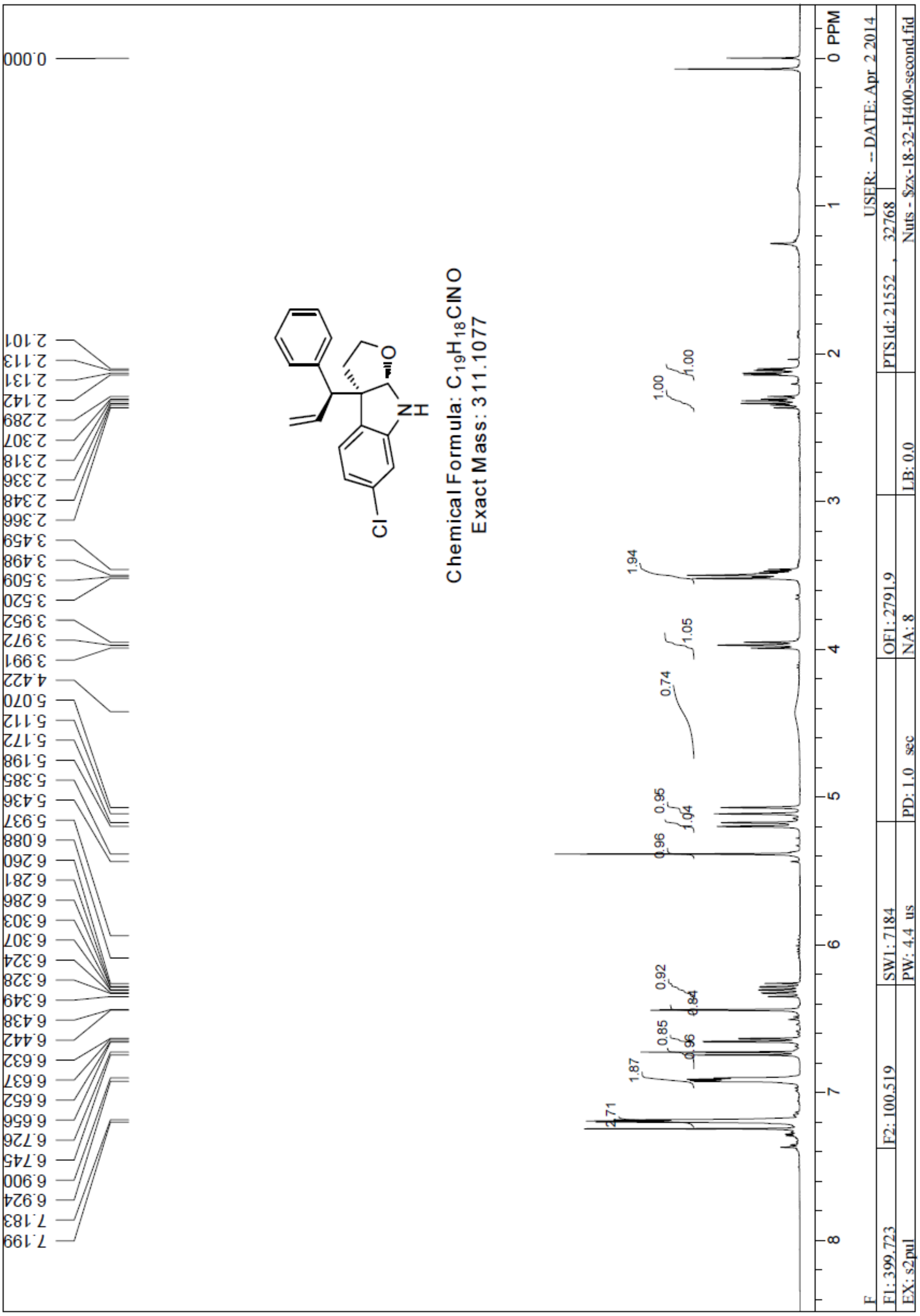

Compound **4fa**'s ^{13}C NMR Spectra

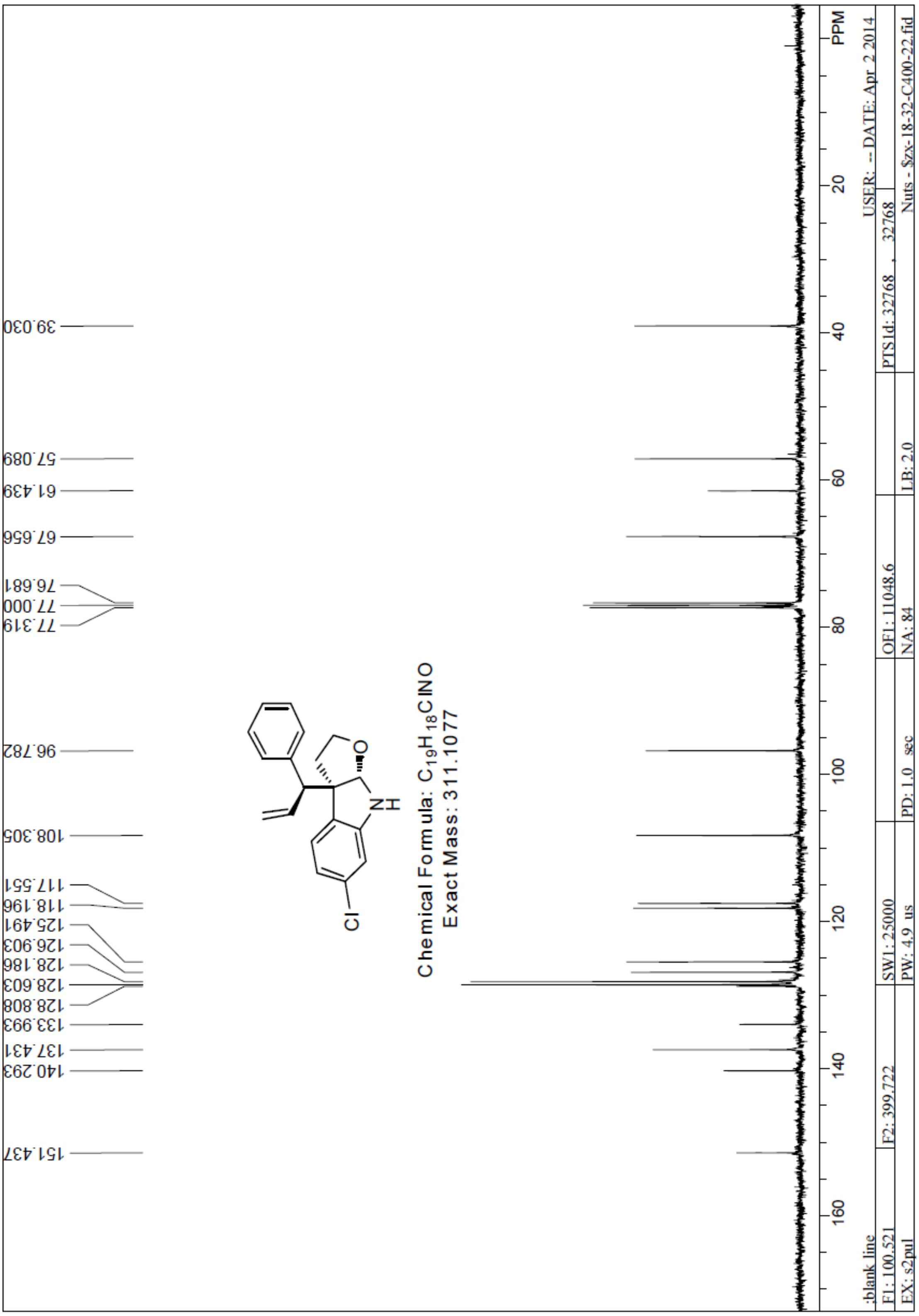

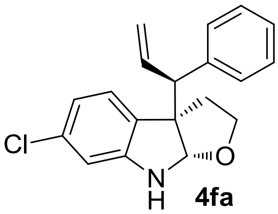

4fa

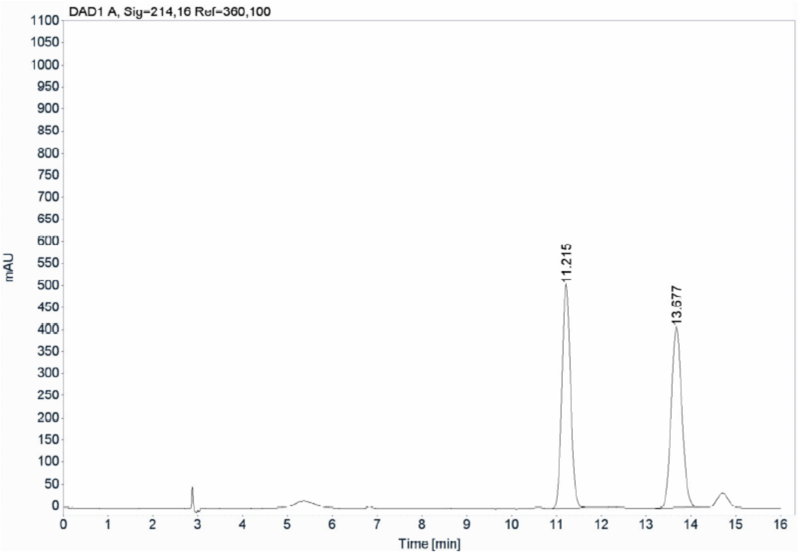

Signal: DAD1 A, Sig=214, 16 Ref=360, 100

RT [min]	Type	Width [min]	Area	Height	Area%
11.215	BB	0.2076	6742.8496	505.6886	49.7880
13.677	MM R	0.2684	6800.2817	408.8831	50.2120
		Sum	13543.1313		

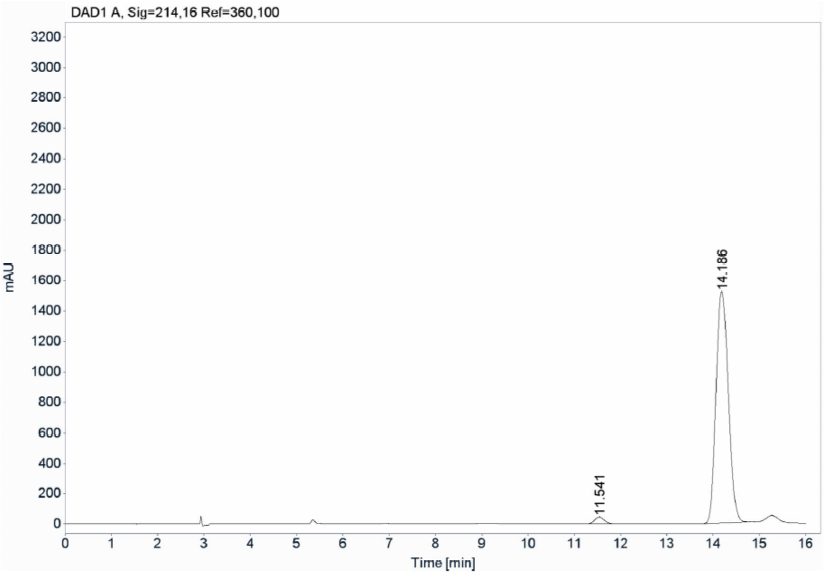

Signal: DAD1 A, Sig=214, 16 Ref=360, 100

RT [min]	Type	Width [min]	Area	Height	Area%
11.541	MM R	0.2437	678.4547	45.7319	2.3628
14.186	BB	0.2914	28036.0645	1518.6459	97.6372
		Sum	28714.5192		

Compound **4ga**’s ^{1}H NMR Spectra

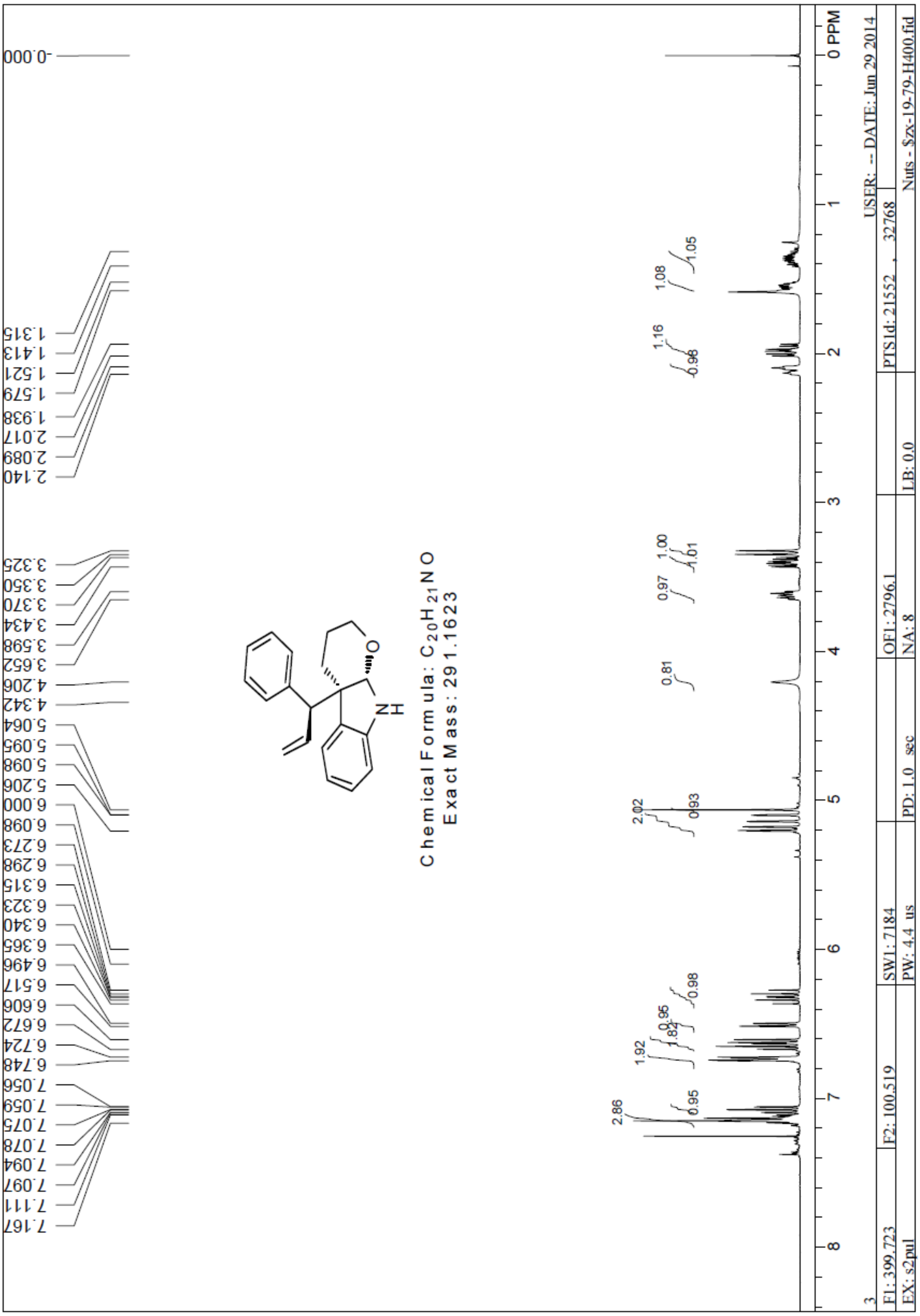

Compound **4ga**'s ^{13}C NMR Spectra

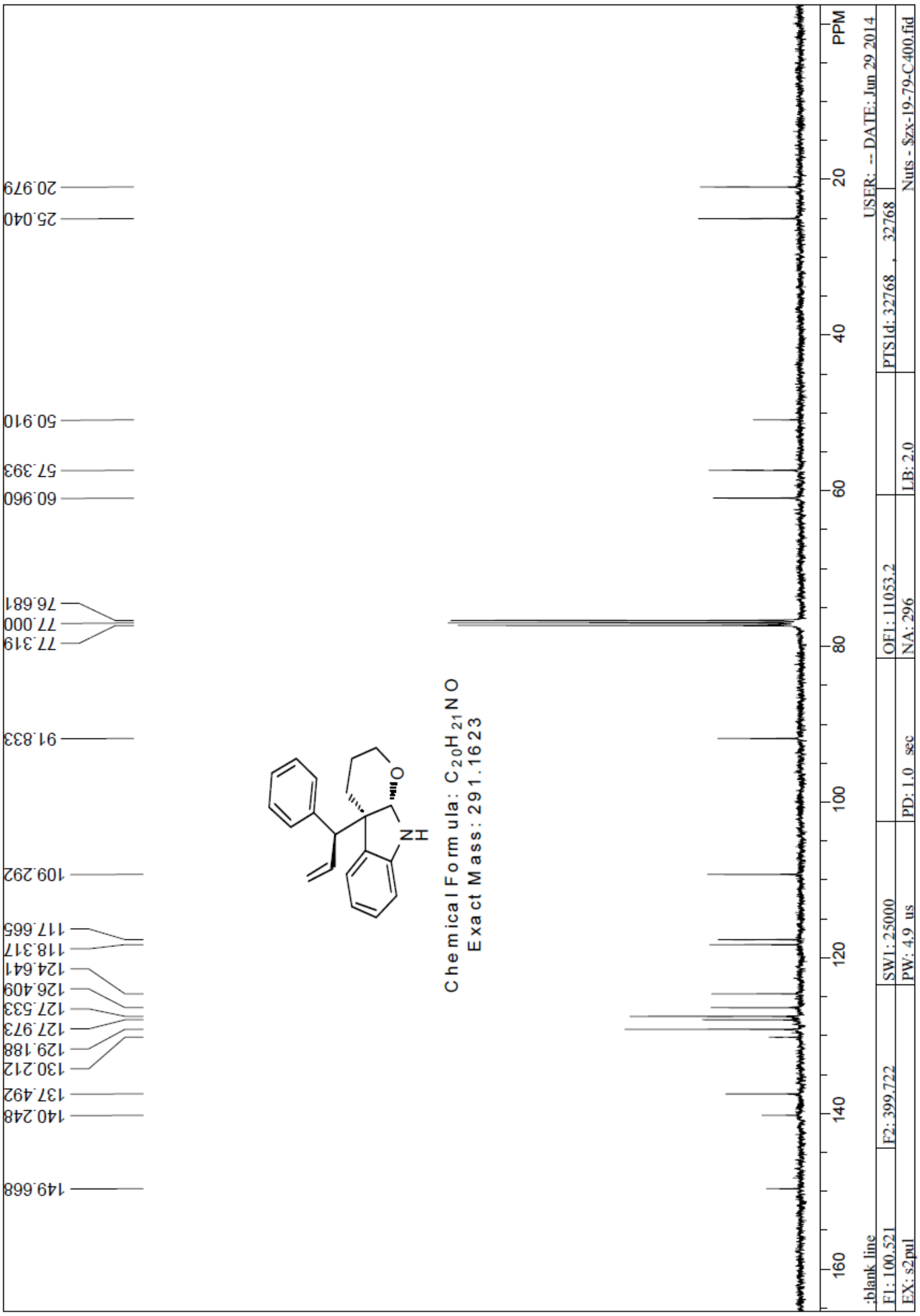

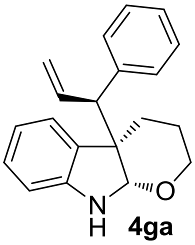

4ga

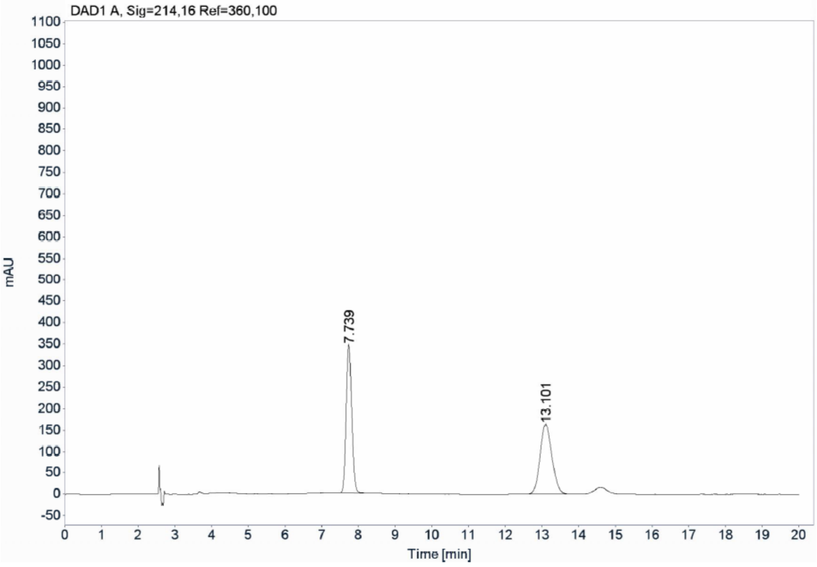

Signal: DAD1 A, Sig=214, 16 Ref=360, 100

RT [min]	Type	Width [min]	Area	Height	Area%
7.739	BV	0.1658	3622.7561	344.1260	49.7159
13.101	BB	0.3490	3664.1548	162.5724	50.2841
		Sum	7286.9109		

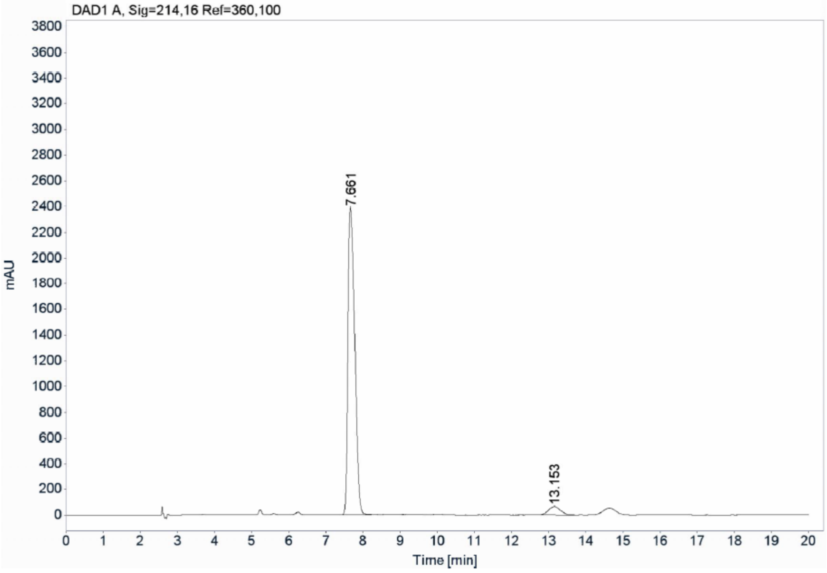

Signal: DAD1 A, Sig=214, 16 Ref=360, 100

RT [min]	Type	Width [min]	Area	Height	Area%
7.661	BV	0.2121	30921.7695	2387.7576	95.5608
13.153	BB	0.3437	1436.4531	63.5545	4.4392
		Sum	32358.2227		

Compound **4ha**'s ^{1}H NMR Spectra

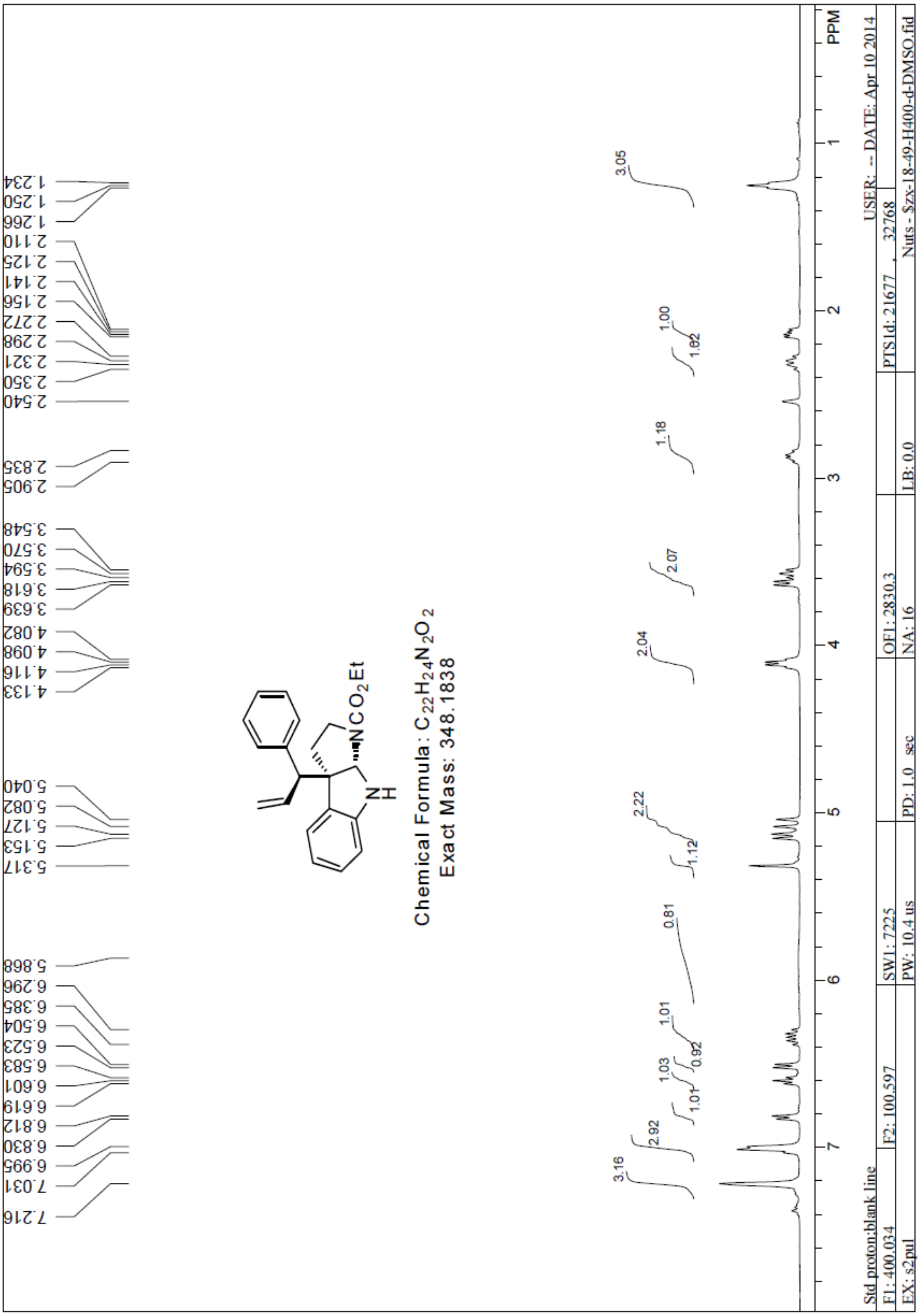

Compound **4ha**'s ^{13}C NMR Spectra

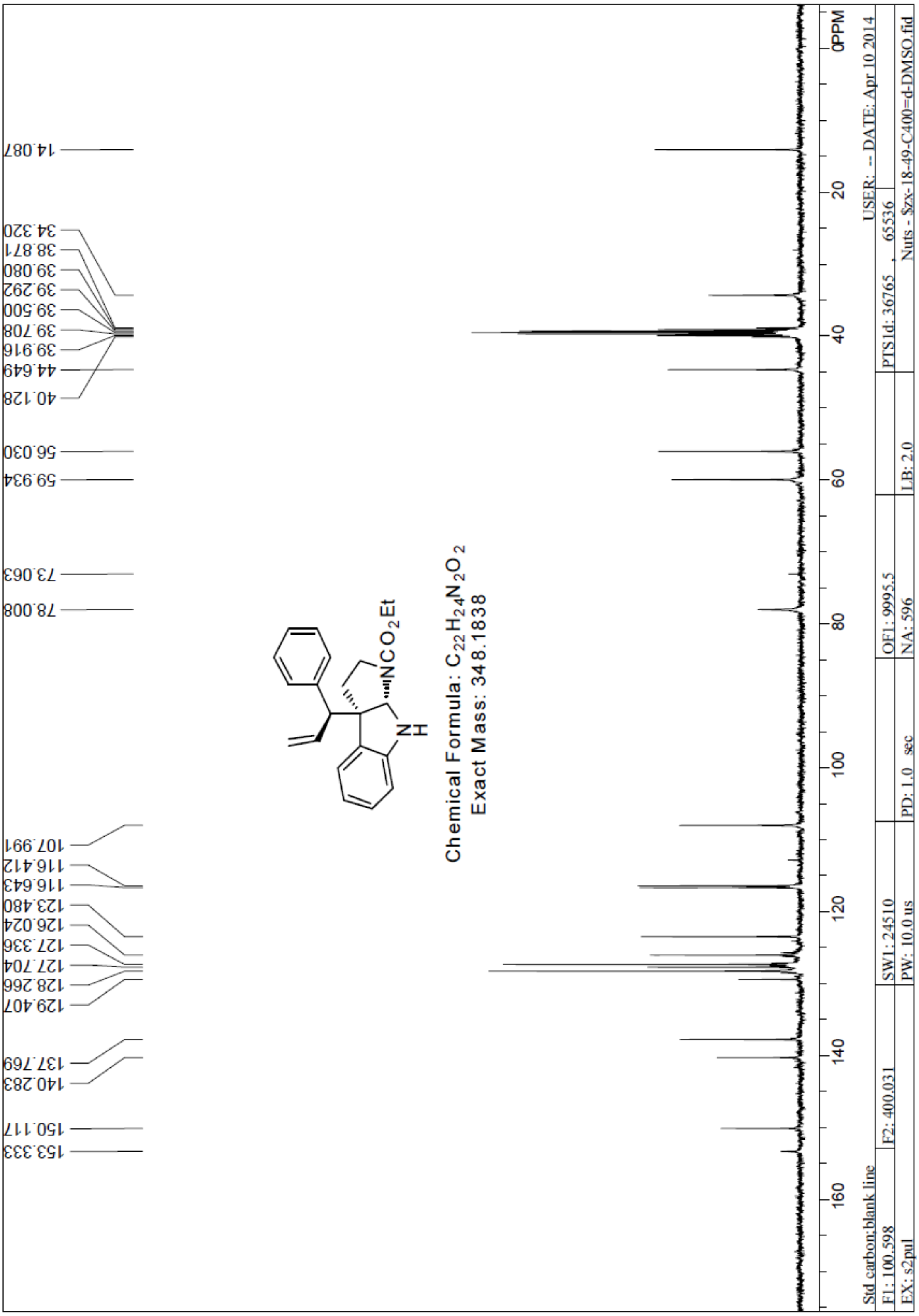

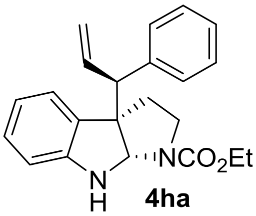

4ha

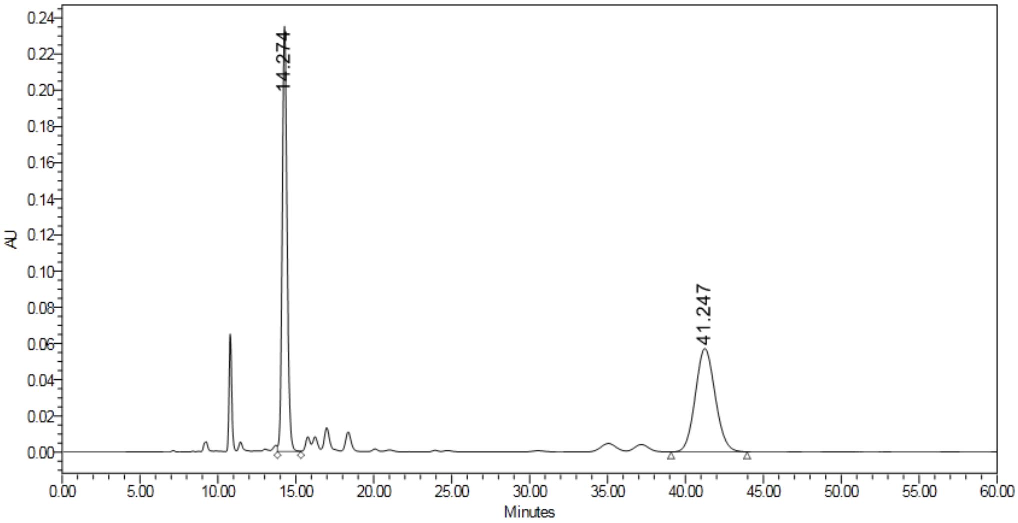

	RT (min)	Area (µV*sec)	% Area	Height (µV)	% Height
1	14.274	5081346	50.06	235135	80.45
2	41.247	5069507	49.94	57125	19.55

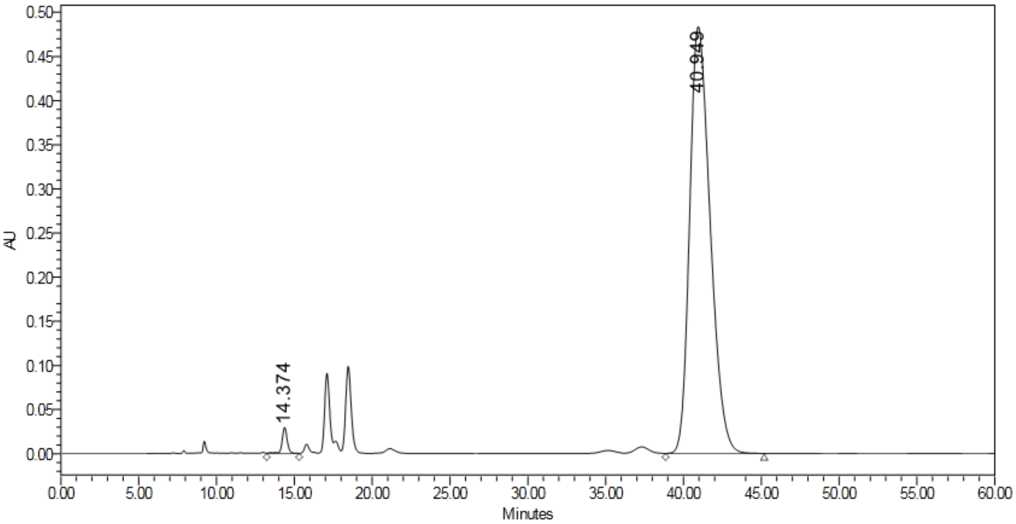

	RT (min)	Area (µV*sec)	% Area	Height (µV)	% Height
1	14.374	692763	1.54	29551	5.76
2	40.949	44379925	98.46	483594	94.24

Compound **4ia**’s ^{1}H NMR Spectra

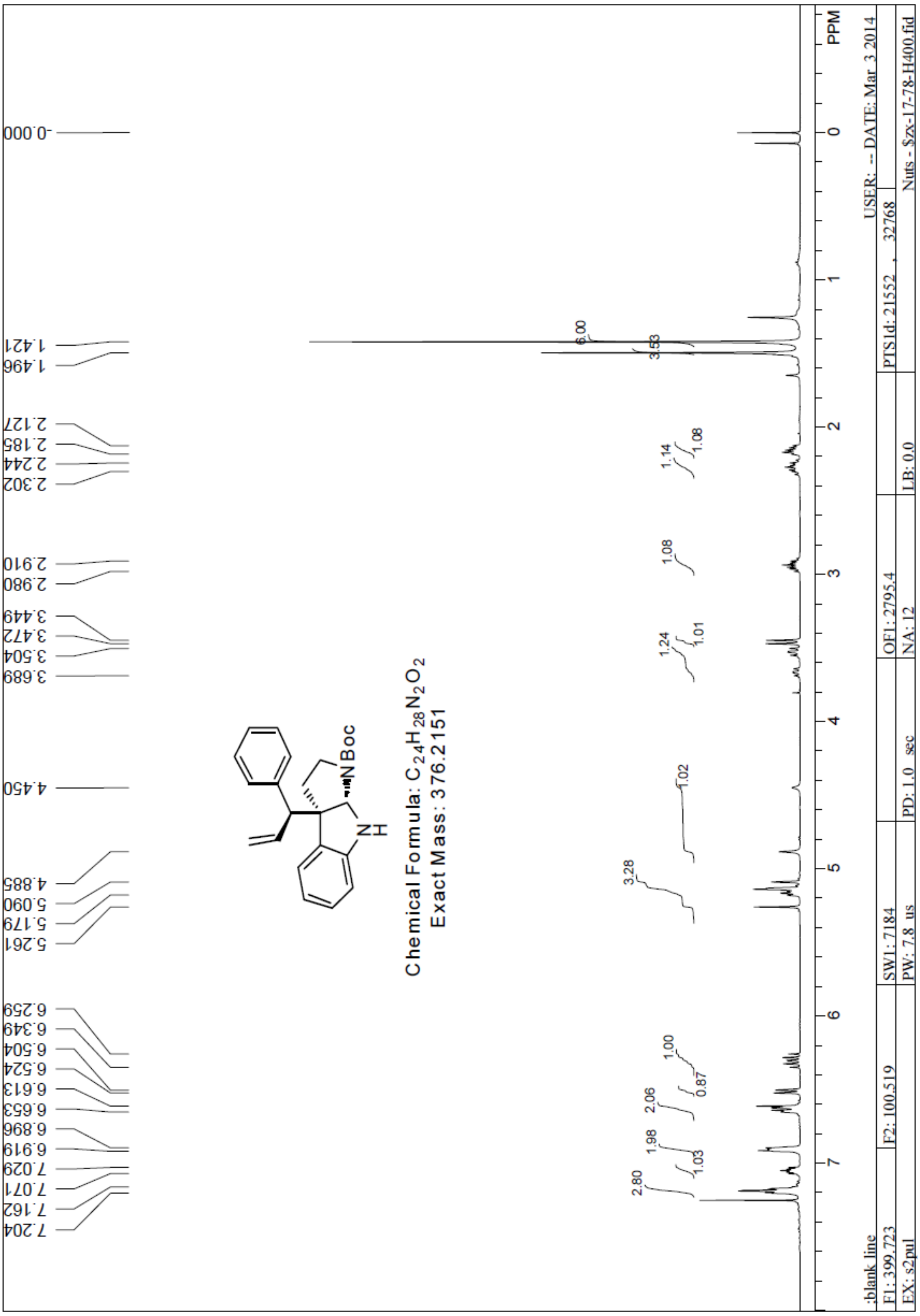

Compound **4ia**’s ^{13}C NMR Spectra

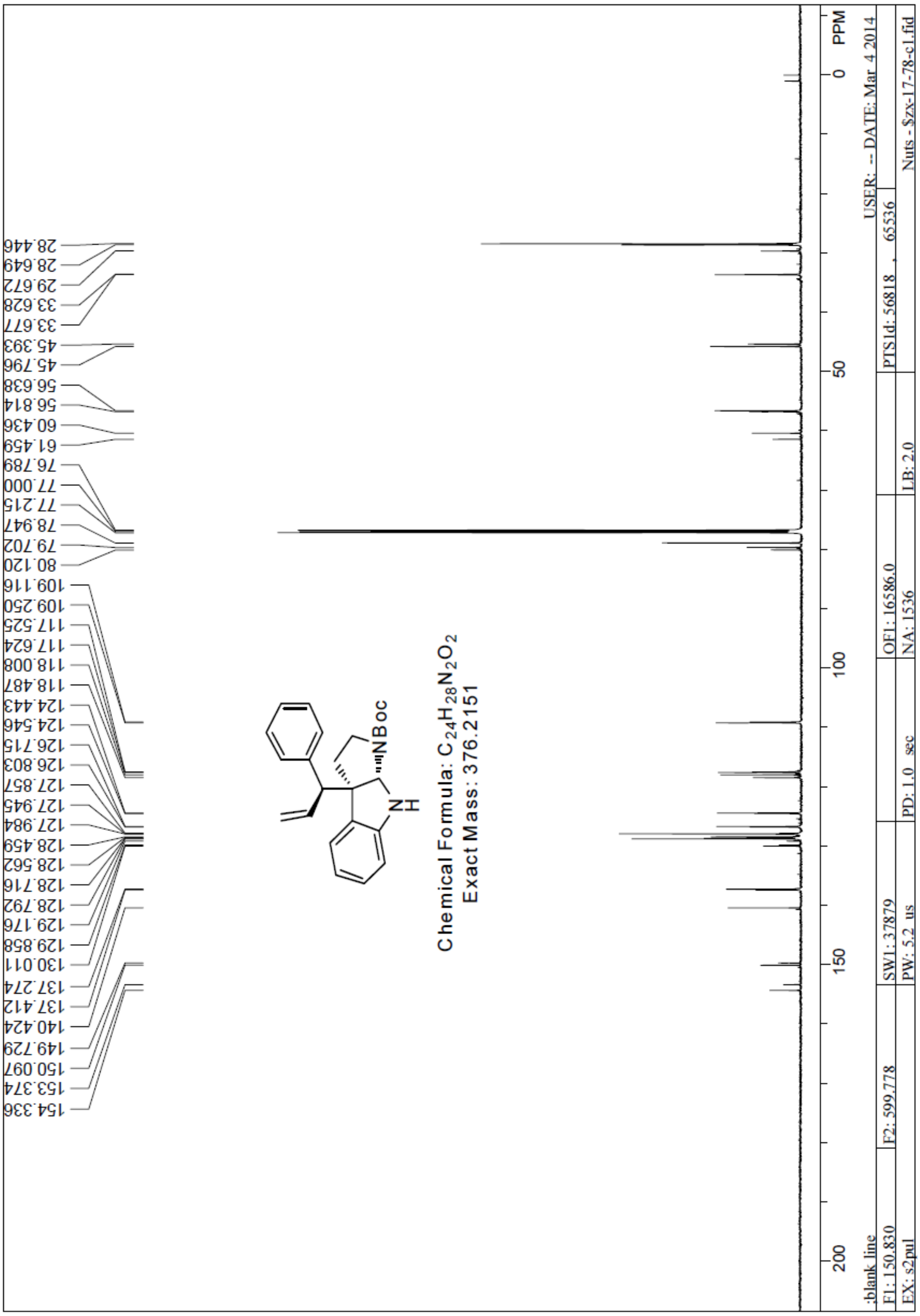

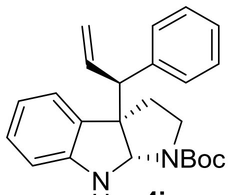

4ia

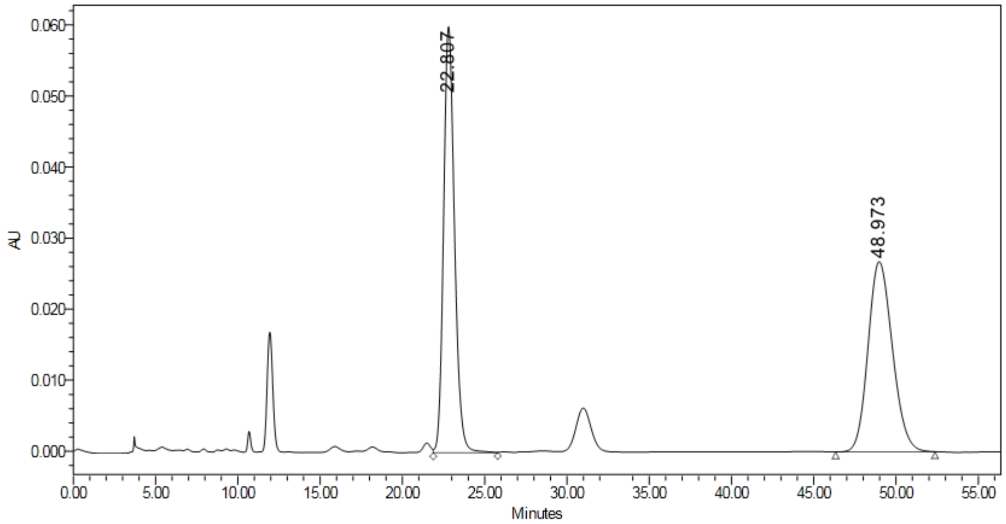

	RT (min)	Area (μV*sec)	% Area	Height (μV)	% Height
1	22.807	2762480	50.03	59982	69.15
2	48.973	2759411	49.97	26761	30.85

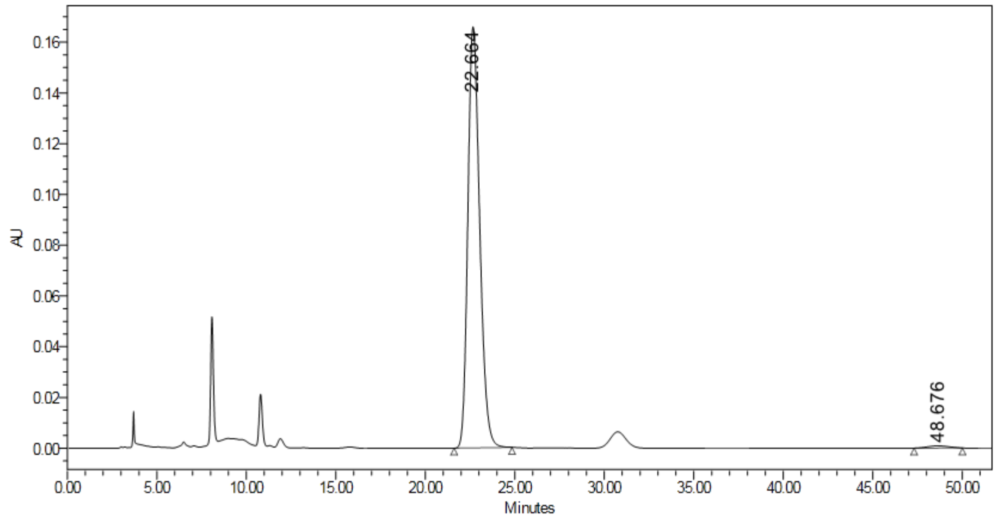

	RT (min)	Area (μV*sec)	% Area	Height (μV)	% Height
1	22.664	7612679	99.08	165937	99.48
2	48.676	71059	0.92	859	0.52

Compound **4ja**'s ^{1}H NMR Spectra

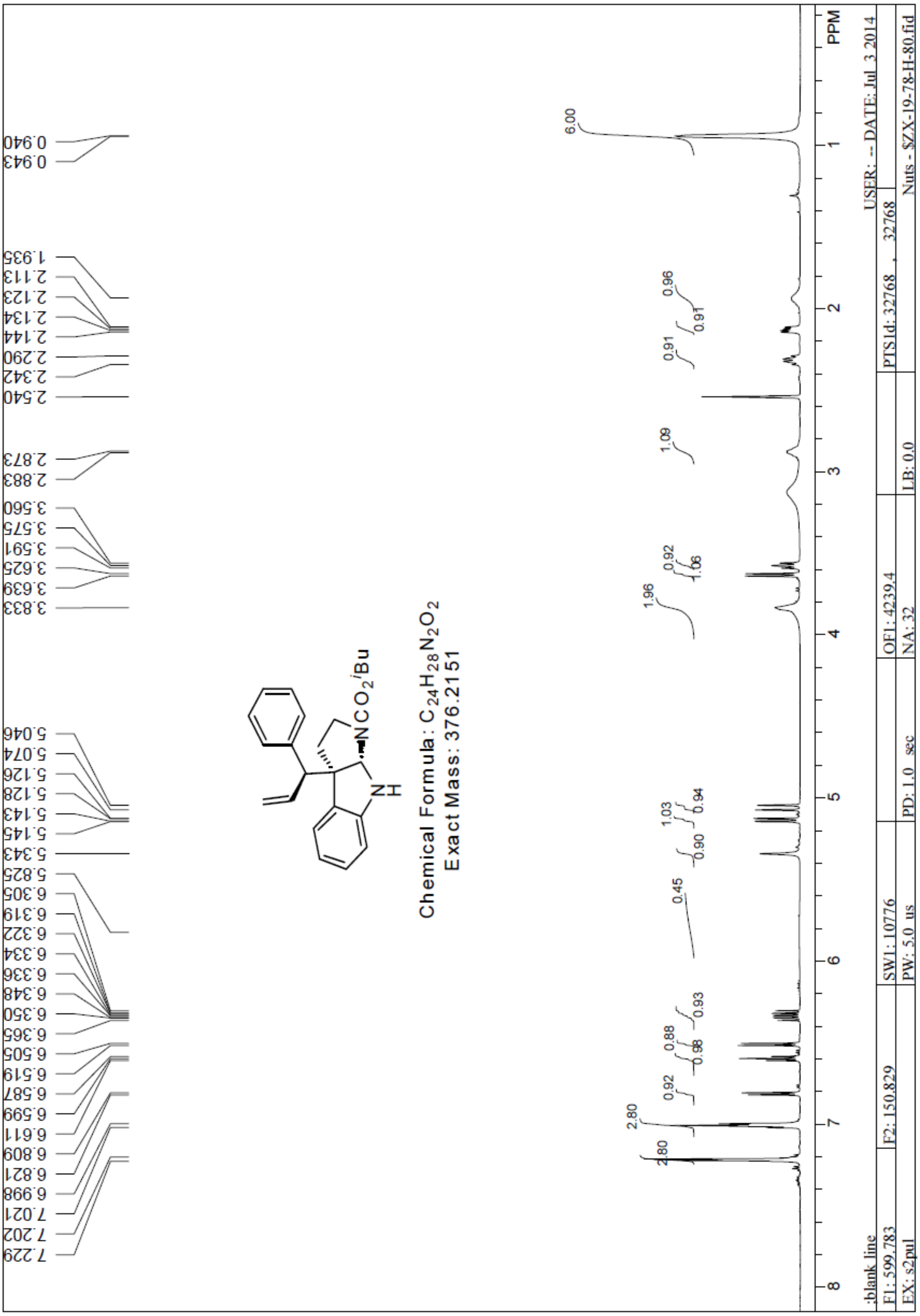

Compound **4ja**'s ^{13}C NMR Spectra

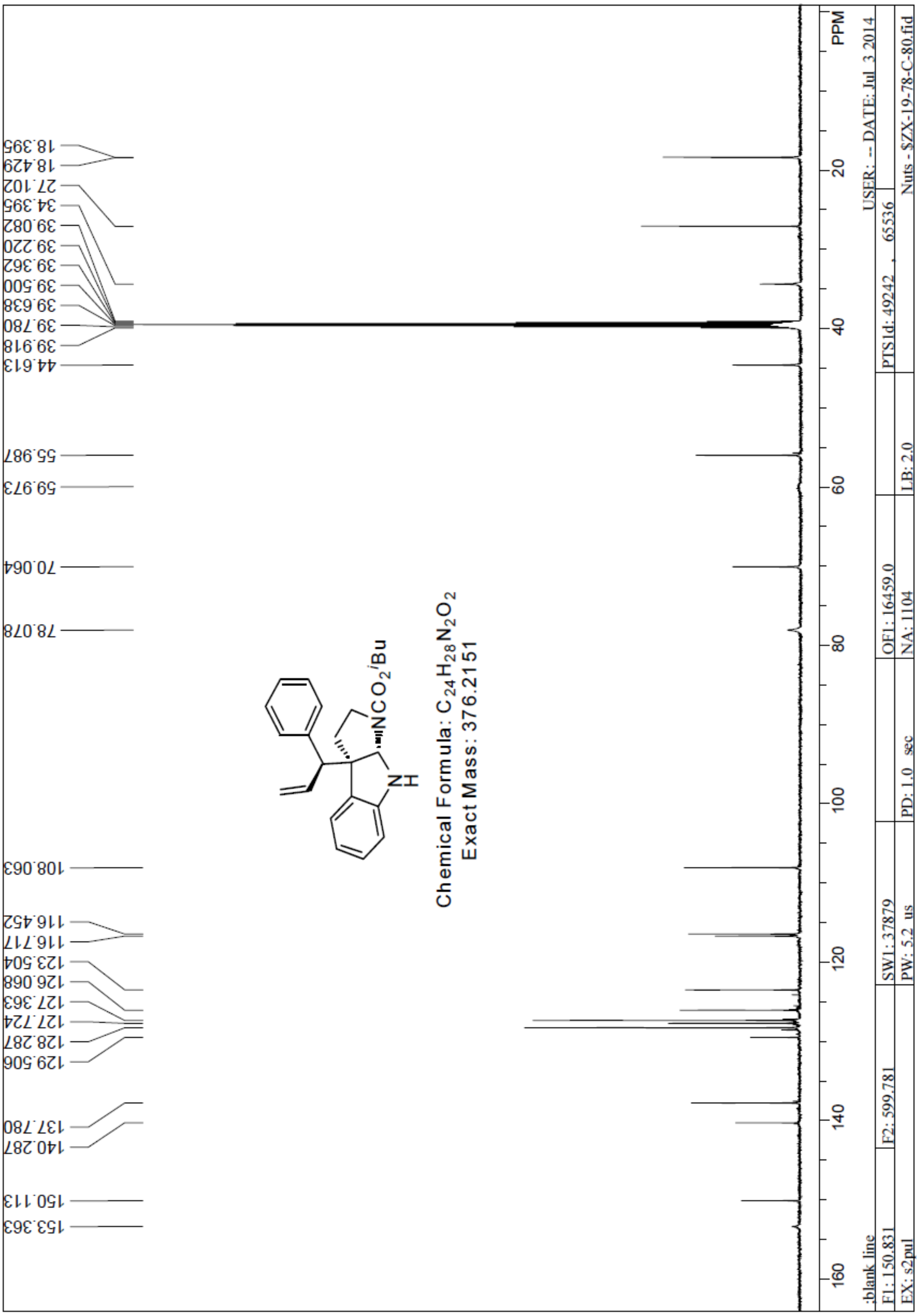

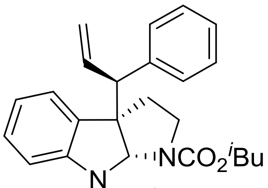

4ja

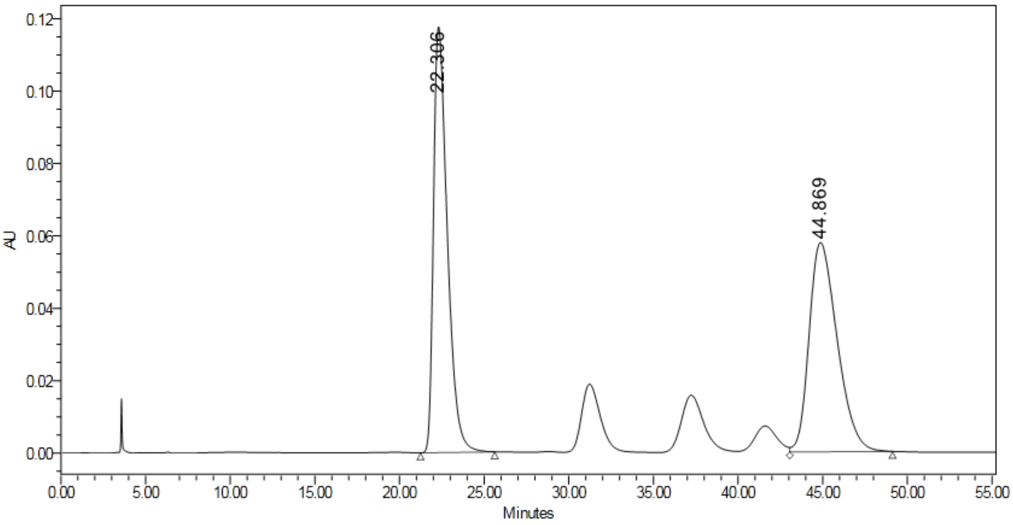

	RT (min)	Area (µV*sec)	% Area	Height (µV)	% Height
1	22.306	6659938	49.97	117663	67.04
2	44.869	6668700	50.03	57853	32.96

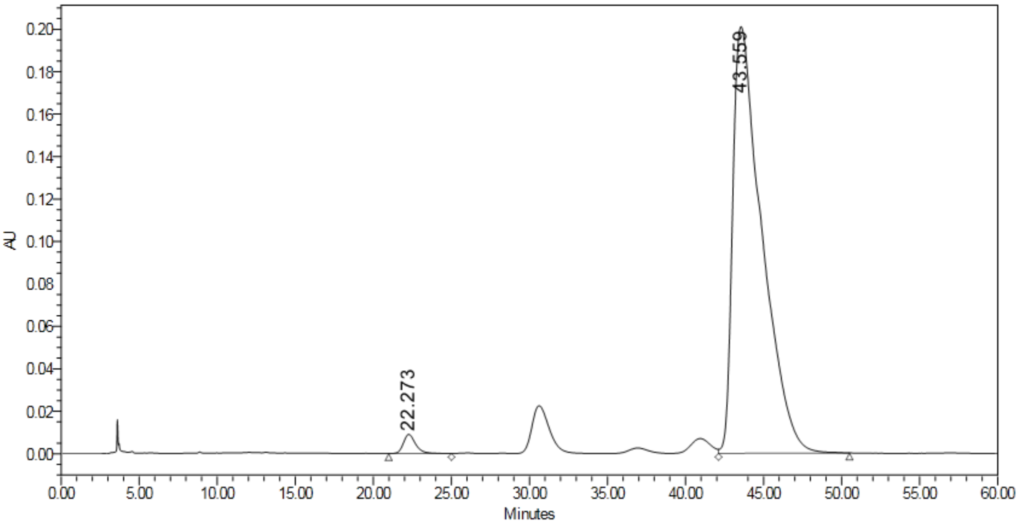

	RT (min)	Area (µV*sec)	% Area	Height (µV)	% Height
1	22.273	497125	1.87	9018	4.29
2	43.559	26125650	98.13	201056	95.71

Compound **4ka**'s ^{1}H NMR Spectra

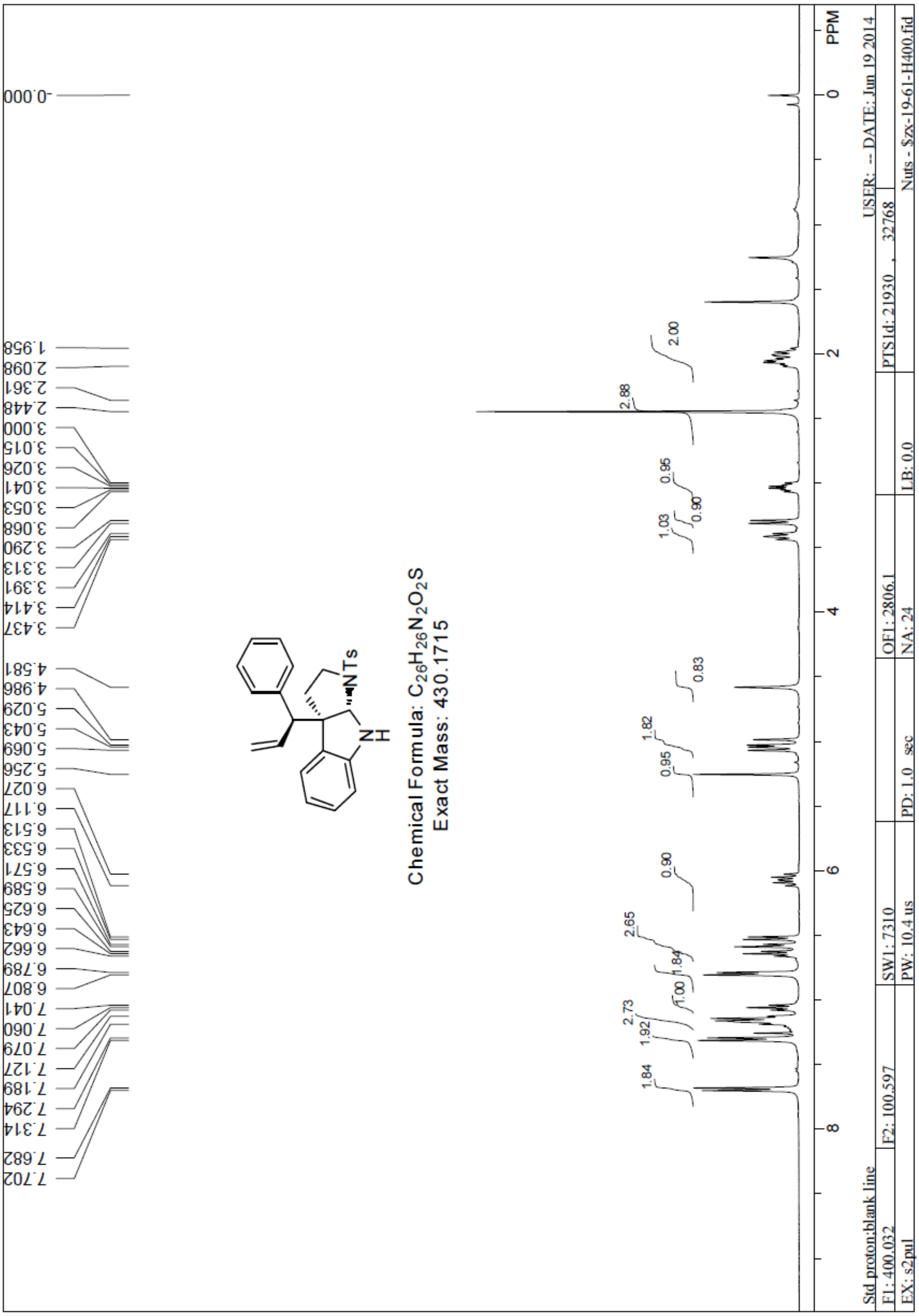

Compound **4ka**'s ^{13}C NMR Spectra

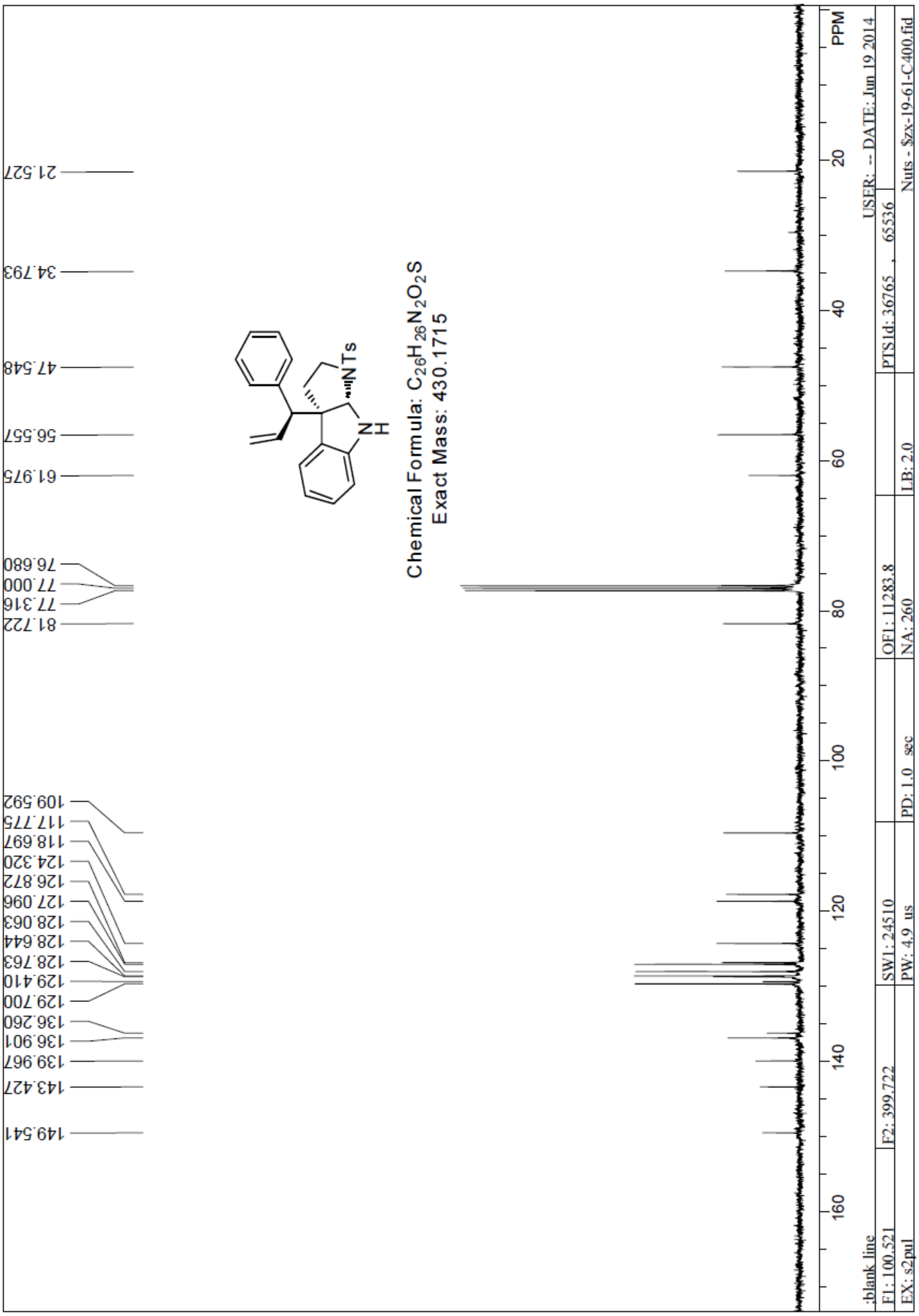

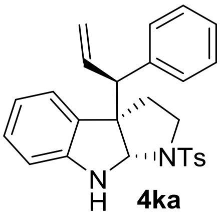

4ka

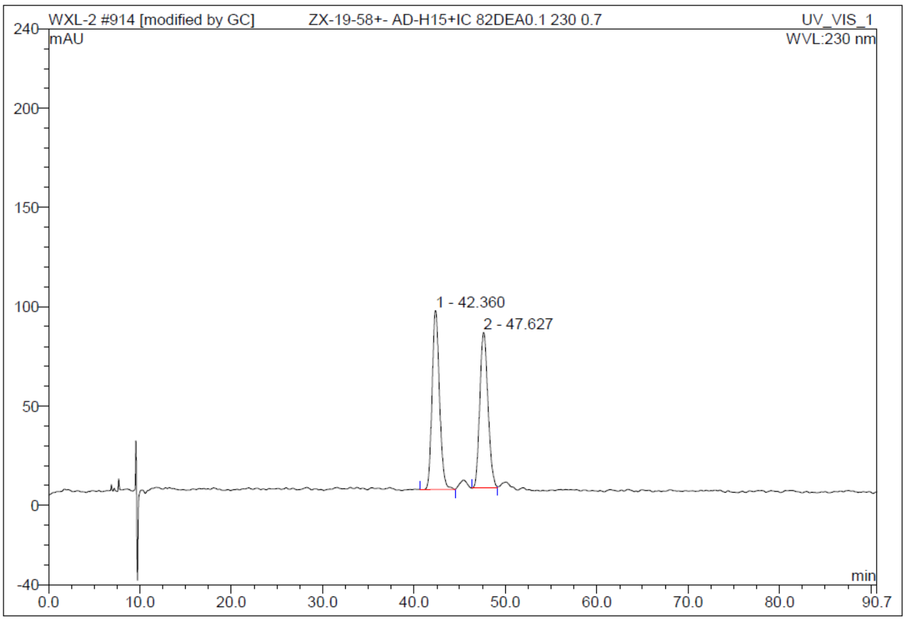

No.	Ret.Time min	Peak Name	Height mAU	Area mAU*min	Rel.Area %	Amount	Type
1	42.36	n.a.	90.189	84.603	50.76	n.a.	BMB*
2	47.63	n.a.	78.491	82.064	49.24	n.a.	BM *
Total:			168.680	166.668	100.00	0.000	

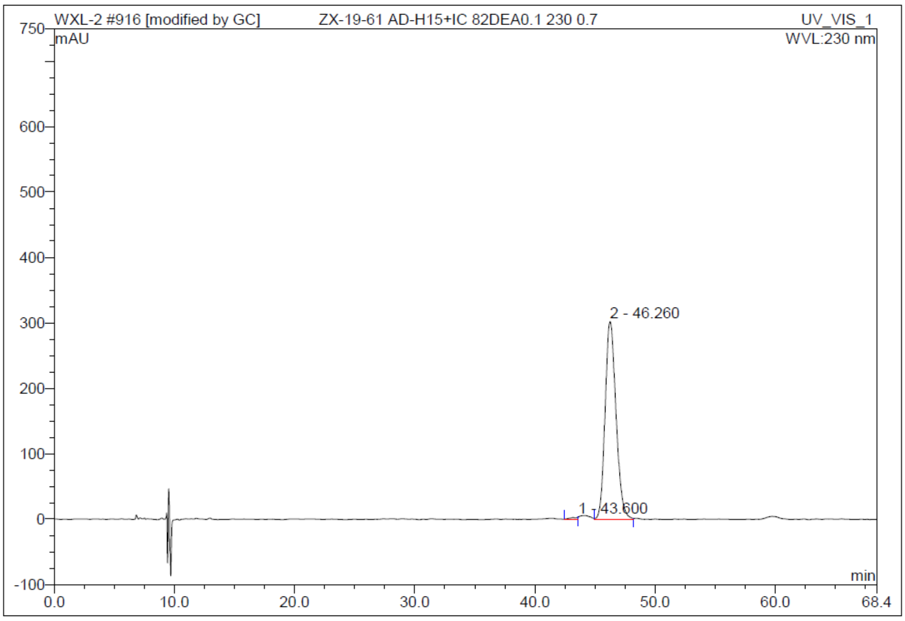

No.	Ret.Time min	Peak Name	Height mAU	Area mAU*min	Rel.Area %	Amount	Type
1	43.60	n.a.	3.065	1.807	0.57	n.a.	BM *
2	46.26	n.a.	302.217	317.271	99.43	n.a.	M *
Total:			305.283	319.078	100.00	0.000	

Compound **4la**'s ^{1}H NMR Spectra

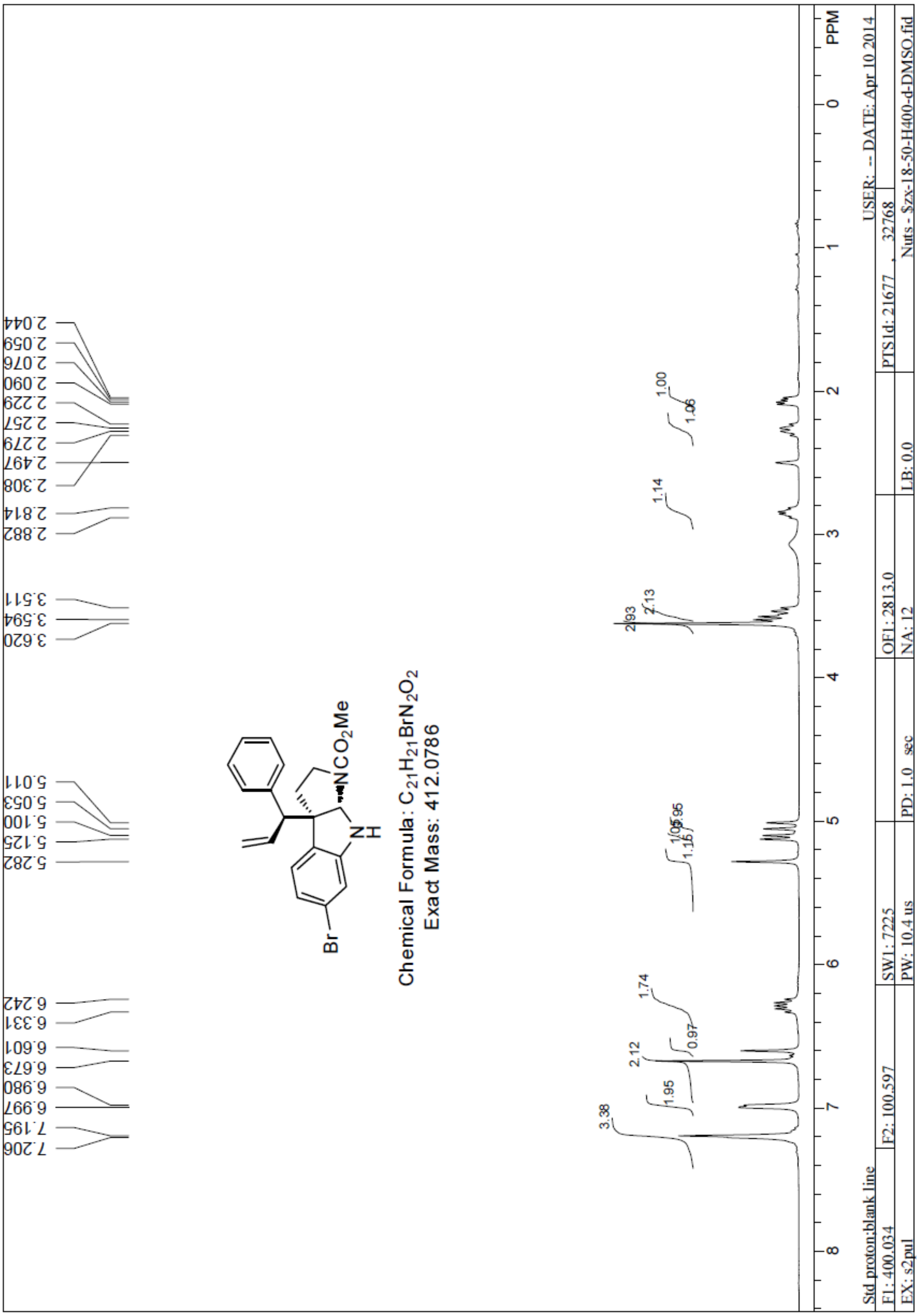

Compound **4la**'s ^{13}C NMR Spectra

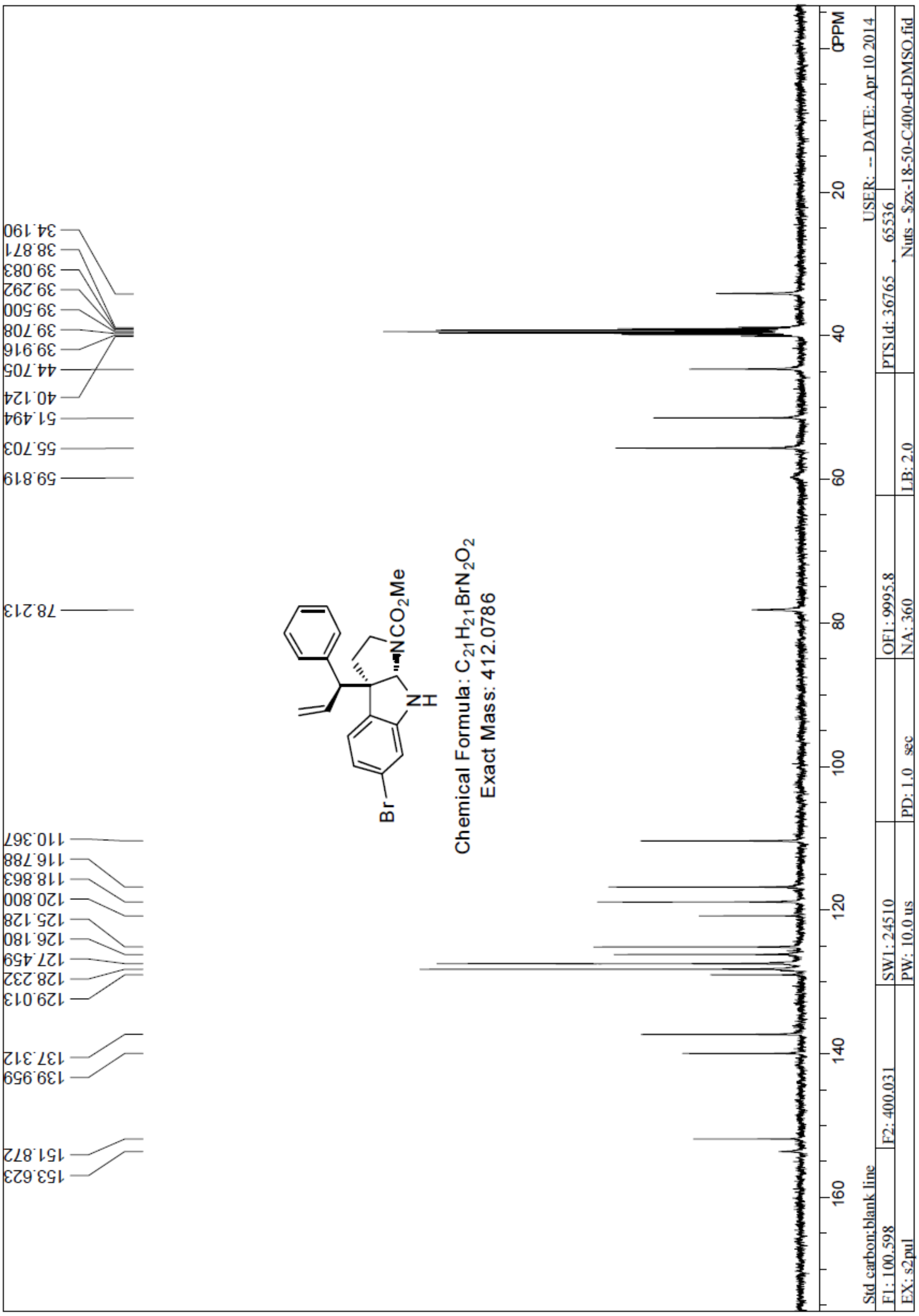

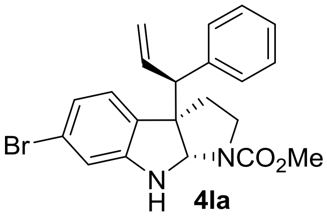

4la

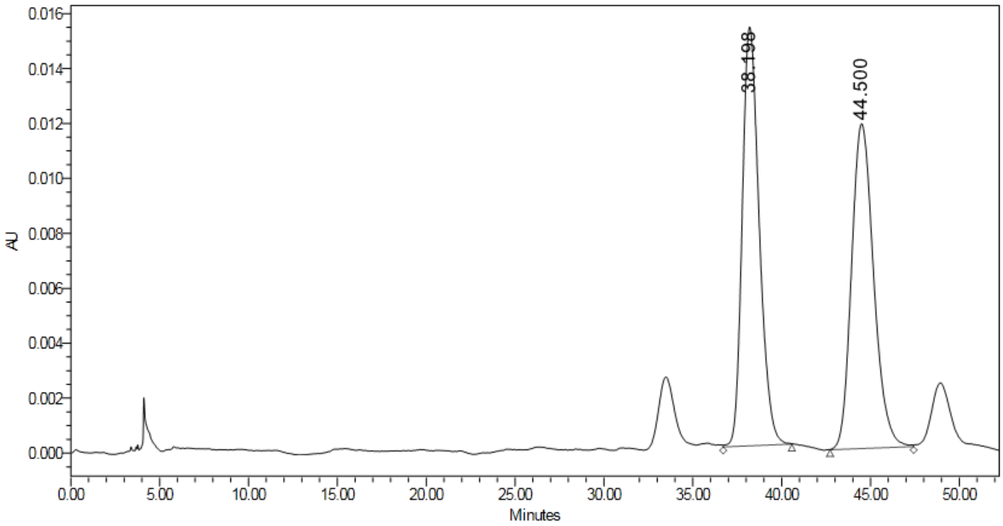

	RT (min)	Area (µV*sec)	% Area	Height (µV)	% Height
1	38.198	1013238	49.73	15247	56.32
2	44.500	1024309	50.27	11825	43.68

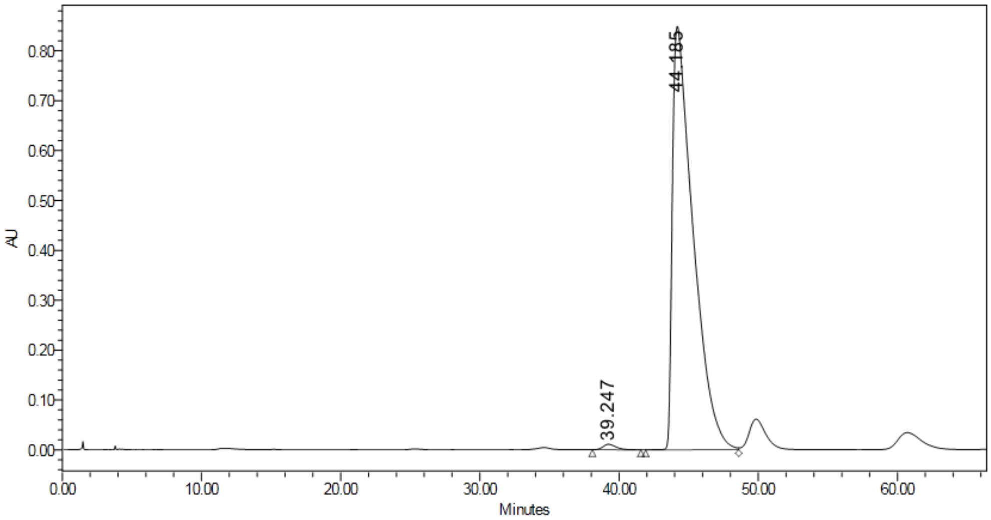

	RT (min)	Area (µV*sec)	% Area	Height (µV)	% Height
1	39.247	704846	0.80	10965	1.28
2	44.185	87788145	99.20	848839	98.72

Compound **4ma**’s ^{1}H NMR Spectra

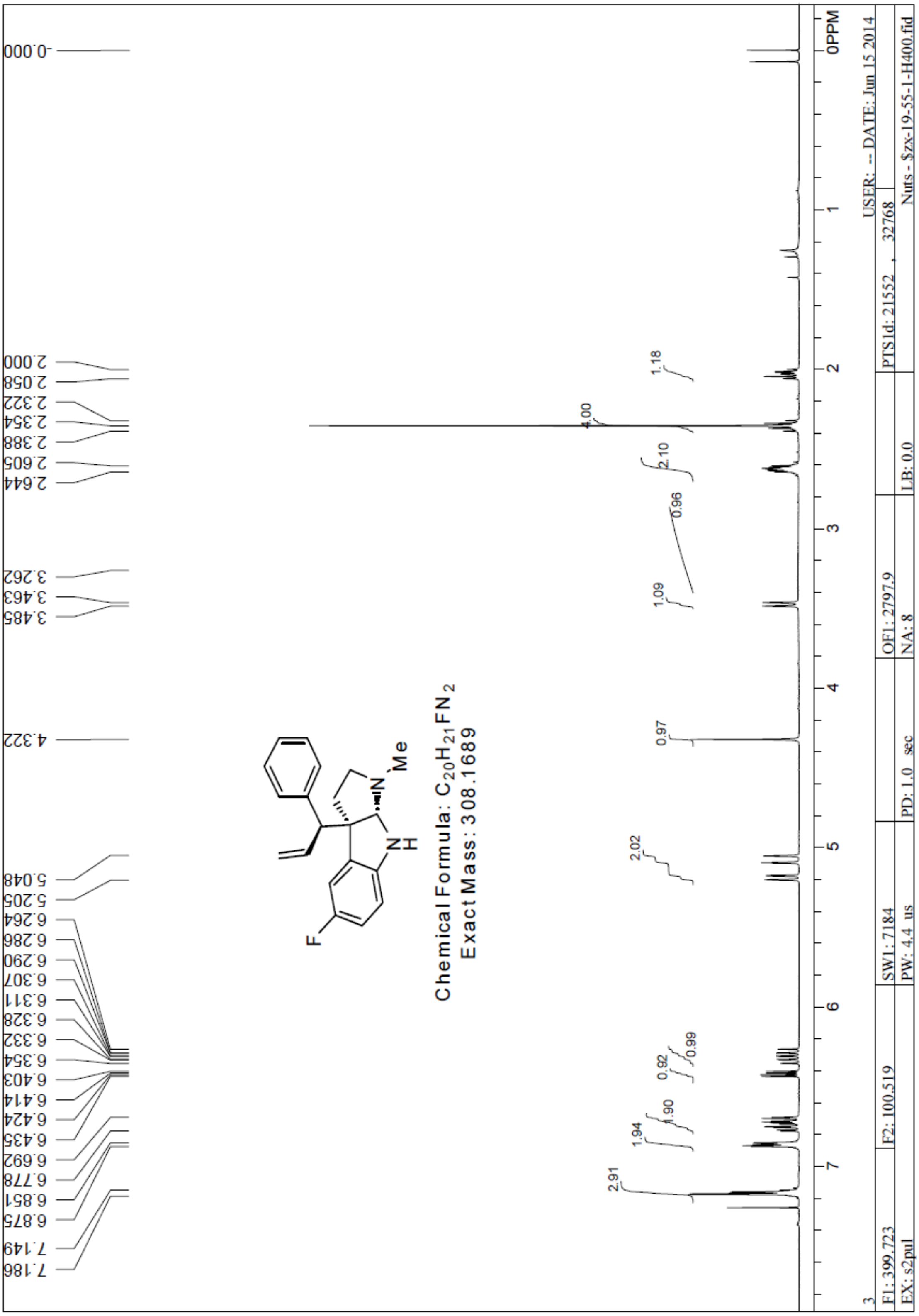

Compound **4ma**'s ^{13}C NMR Spectra

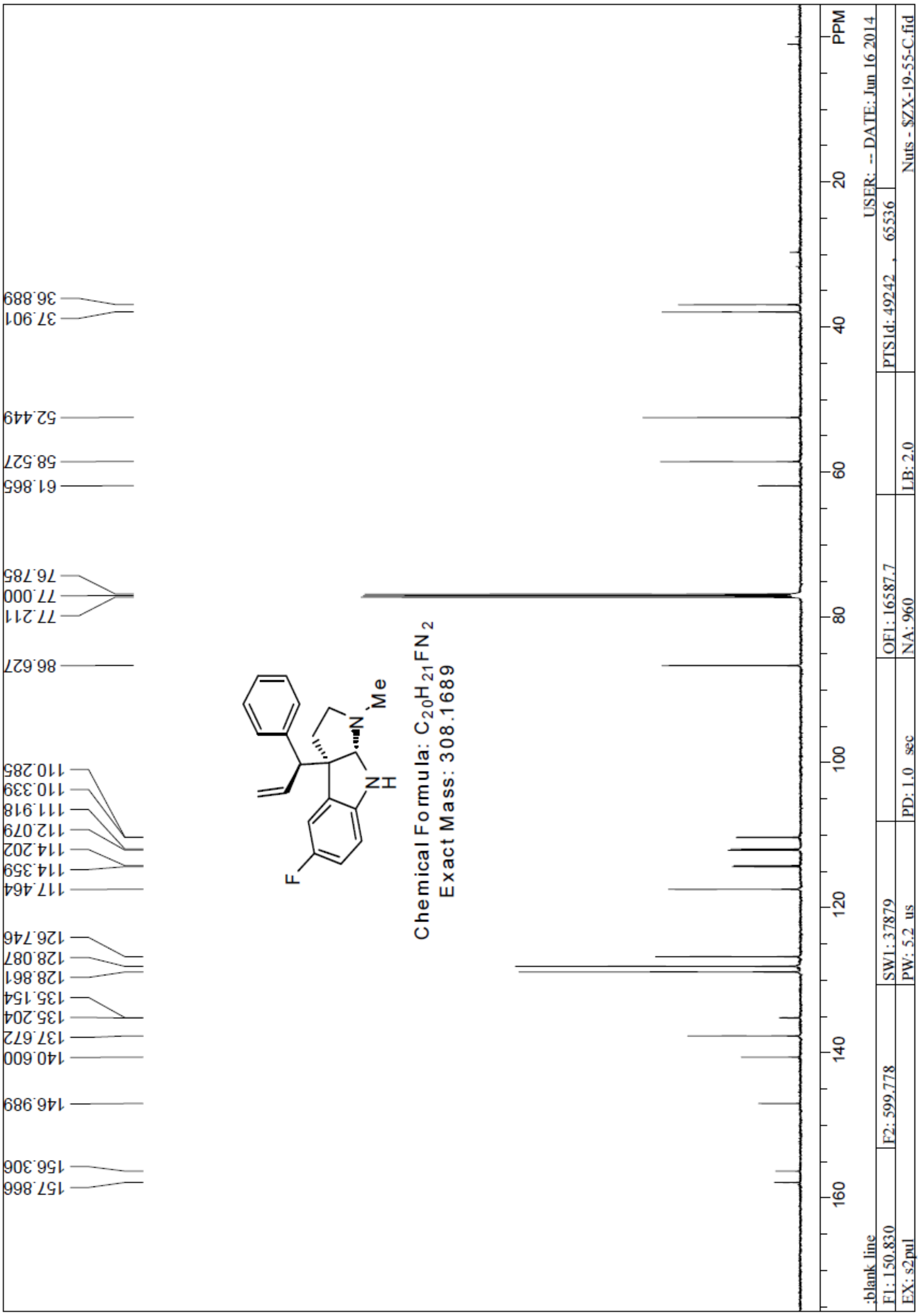

Compound **4ma**’s ^{19}F NMR Spectra

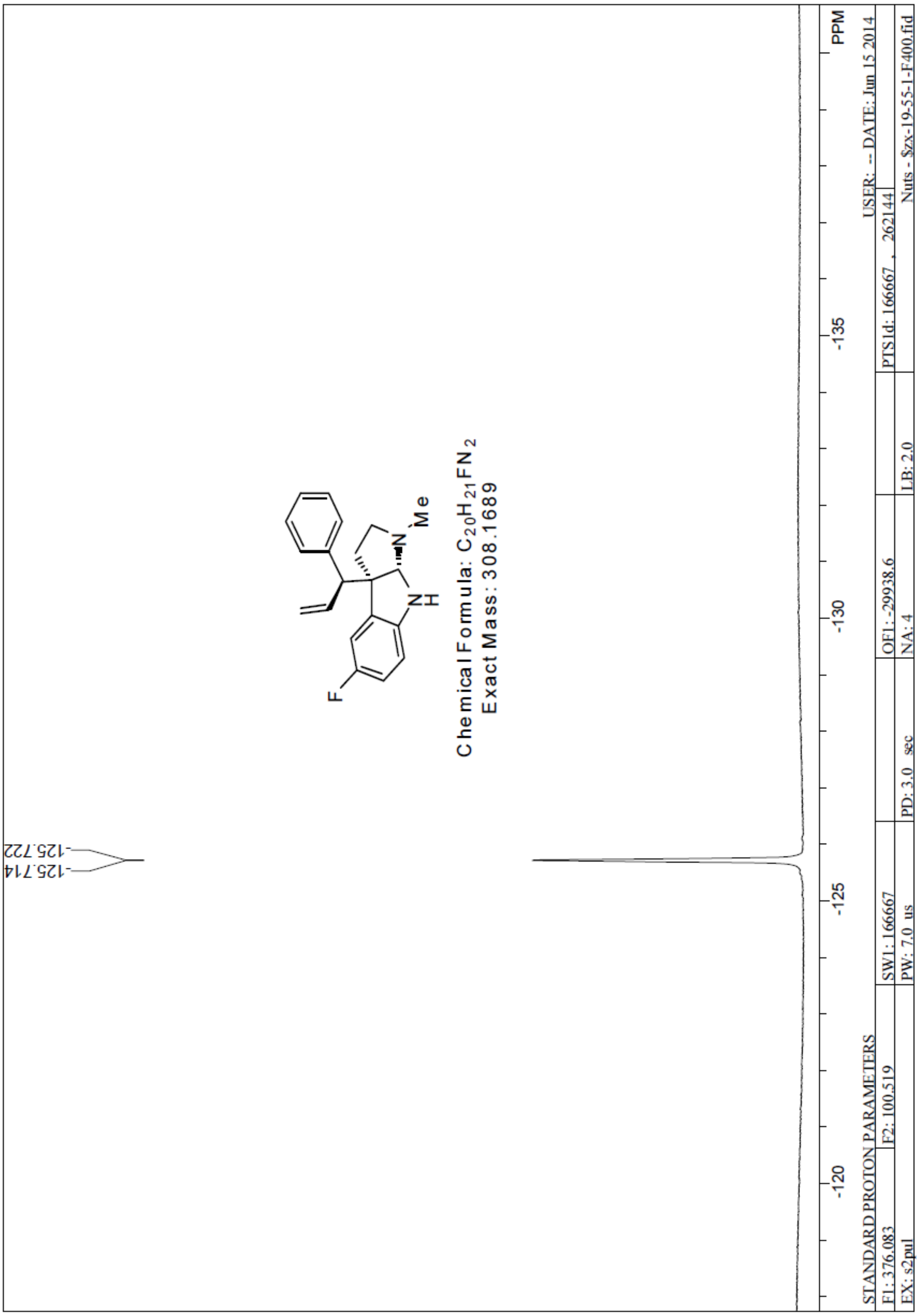

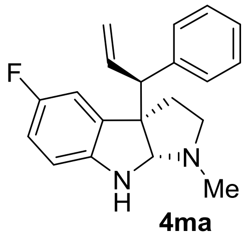

4ma

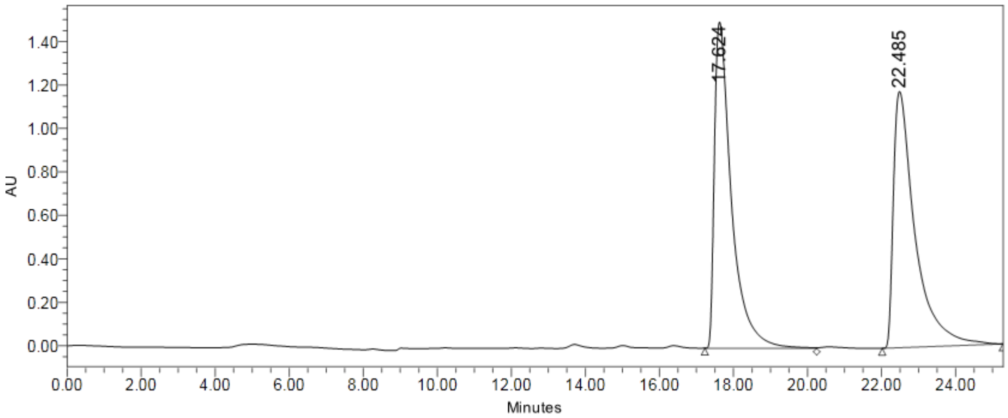

Channel: W2489 ChA; Processed Channel: W2489 ChA 214nm; Injection: 1; Date Acquired: 6/15/2014 10:45:20 PM CST; Result Id: 1296; Processing Method: 1

Peak Name:

	Injection	RT	Area	% Area	Height
1	1	22.485	47838783	49.85	1179045
2	1	17.624	48121729	50.15	1501299
Mean		20.055			
Std. Dev.		3.438			
% RSD		17.14			

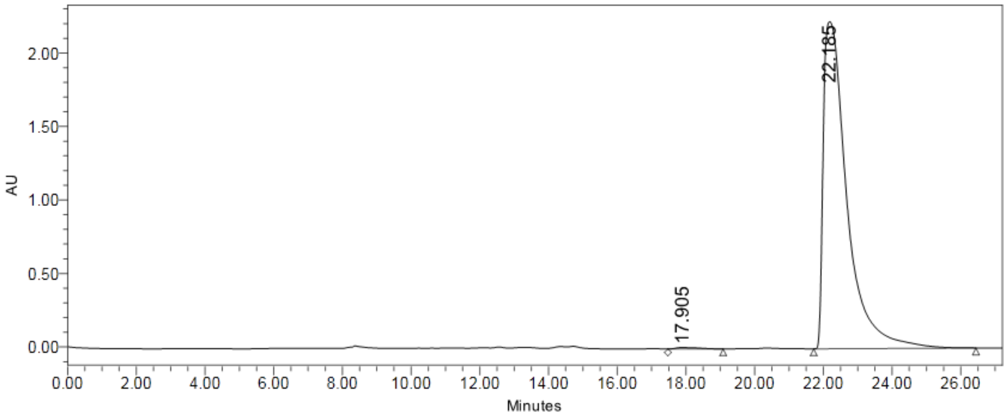

Channel: W2489 ChA; Processed Channel: W2489 ChA 214nm; Injection: 1; Date Acquired: 6/15/2014 11:11:02 PM CST; Result Id: 1298; Processing Method: 1

Peak Name:

	Injection	RT	Area	% Area	Height
1	1	22.185	106358335	99.58	2225673
2	1	17.905	449010	0.42	10887
Mean		20.045			
Std. Dev.		3.026			
% RSD		15.10			

Compound **4na**'s ^{1}H NMR Spectra

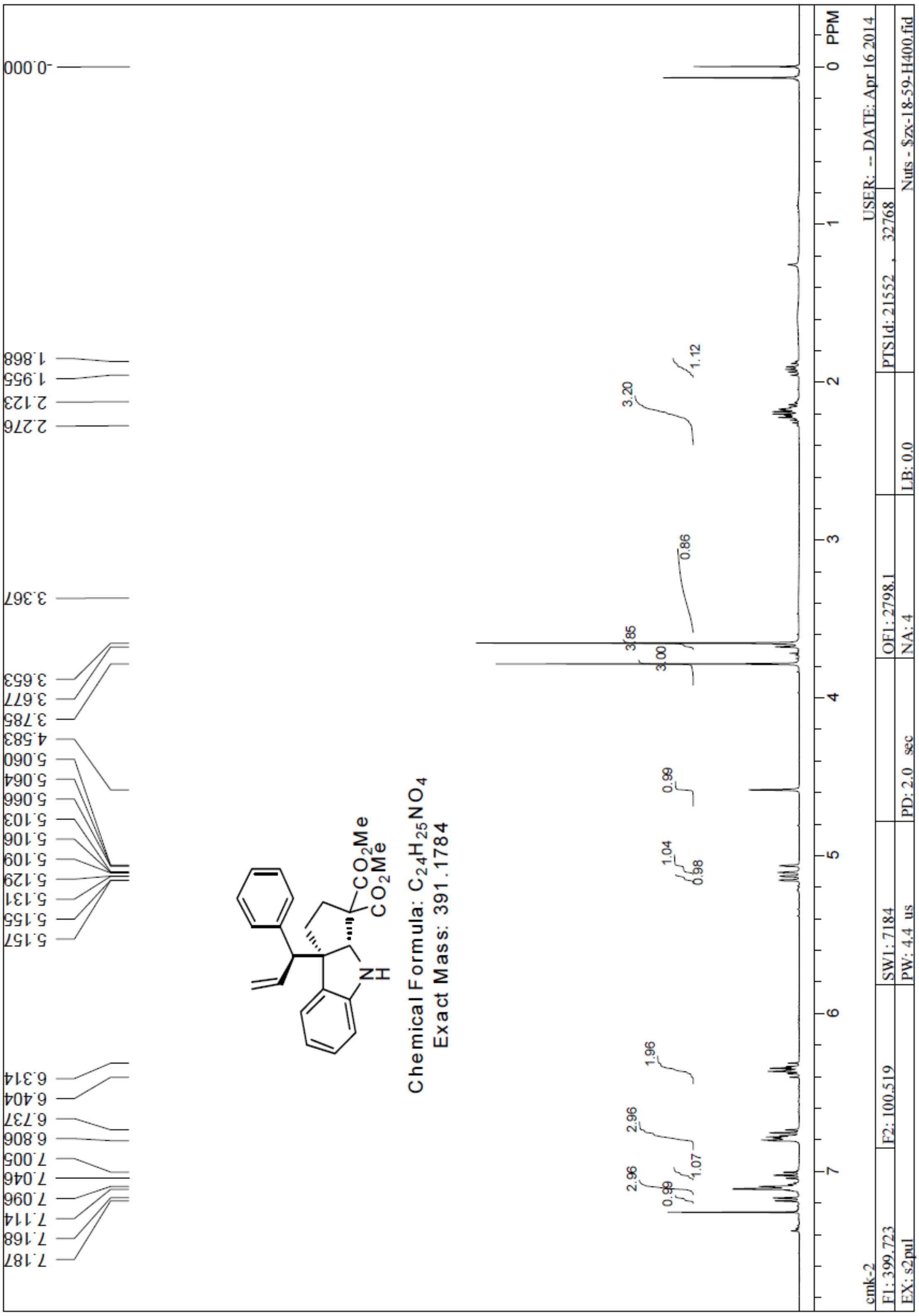

Compound **4na**'s ^{13}C NMR Spectra

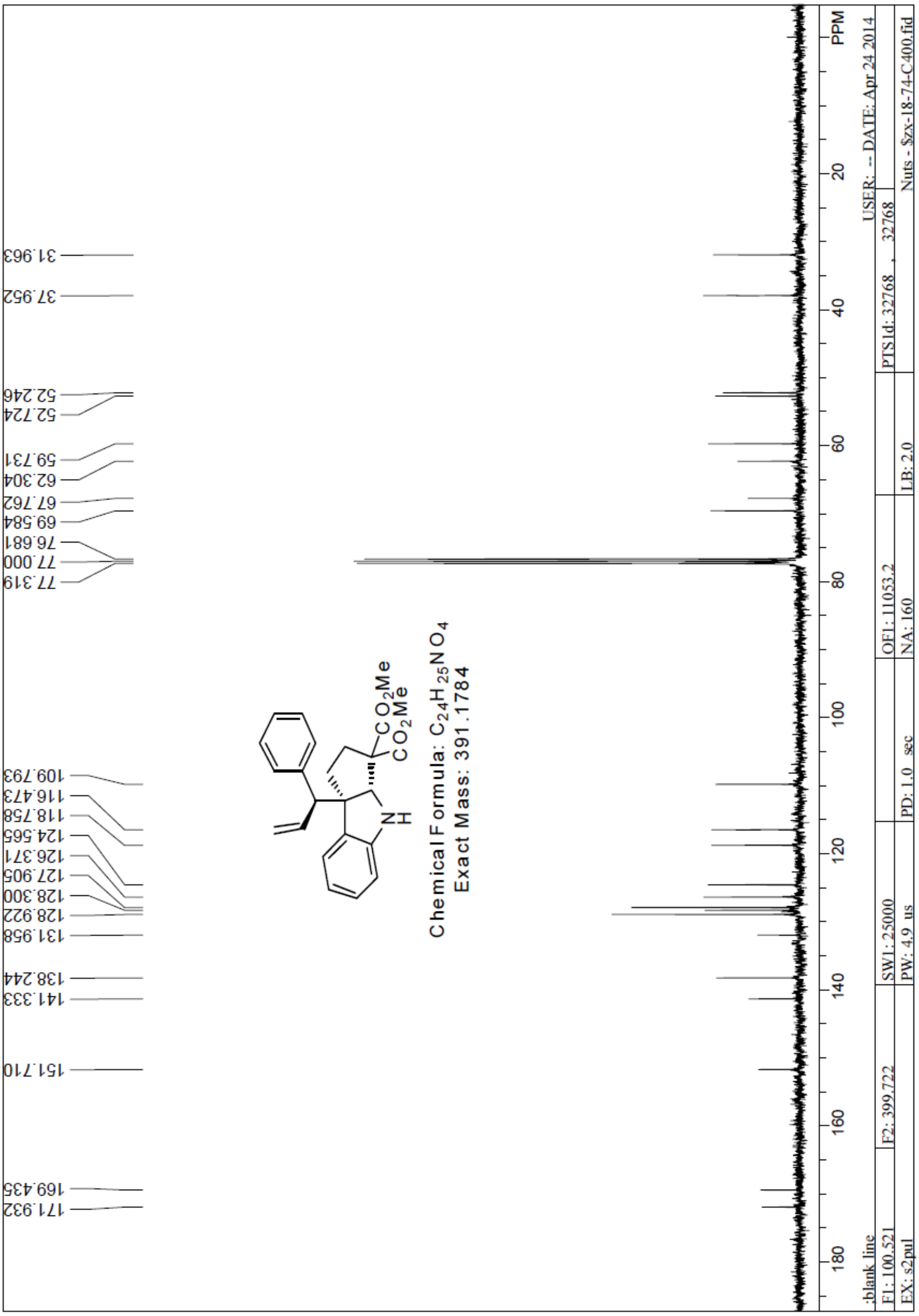

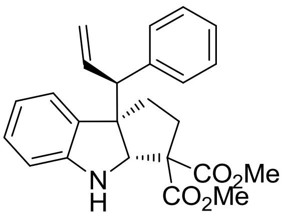

4na

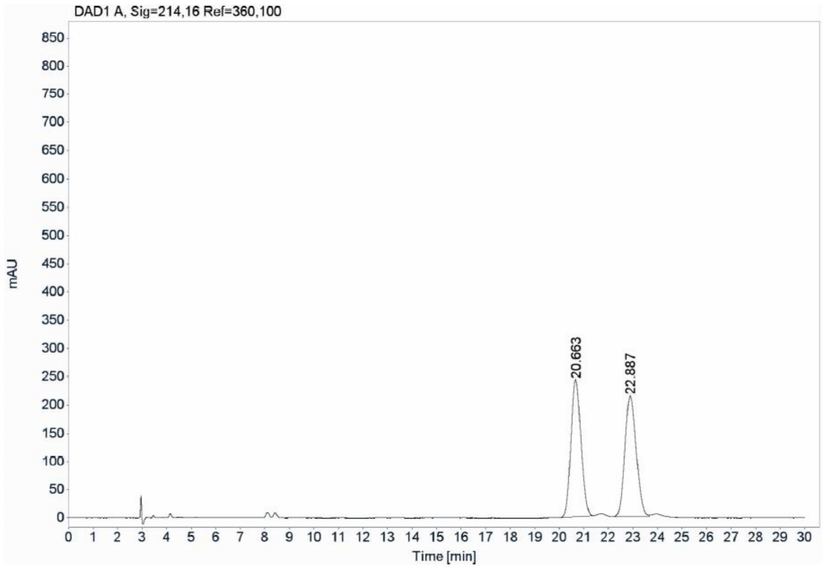

Signal: DAD1 A, Sig=214, 16 Ref=360, 100

RT [min]	Type	Width [min]	Area	Height	Area%
20.663	BB	0.4270	6924.3062	242.5693	49.9690
22.887	BV	0.4585	6932.8911	214.2695	50.0310
		Sum	13857.1973		

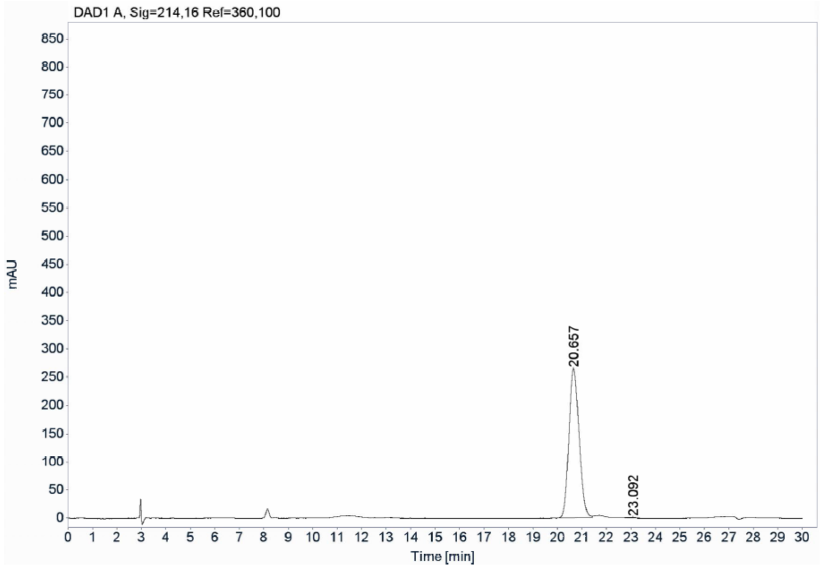

Signal: DAD1 A, Sig=214, 16 Ref=360, 100

RT [min]	Type	Width [min]	Area	Height	Area%
20.657	BV	0.4441	7656.1157	264.9999	99.5629
23.092	MM R	0.5352	33.6144	1.0467	0.4371
		Sum	7689.7302		

Compound **4oa**'s ^{1}H NMR Spectra

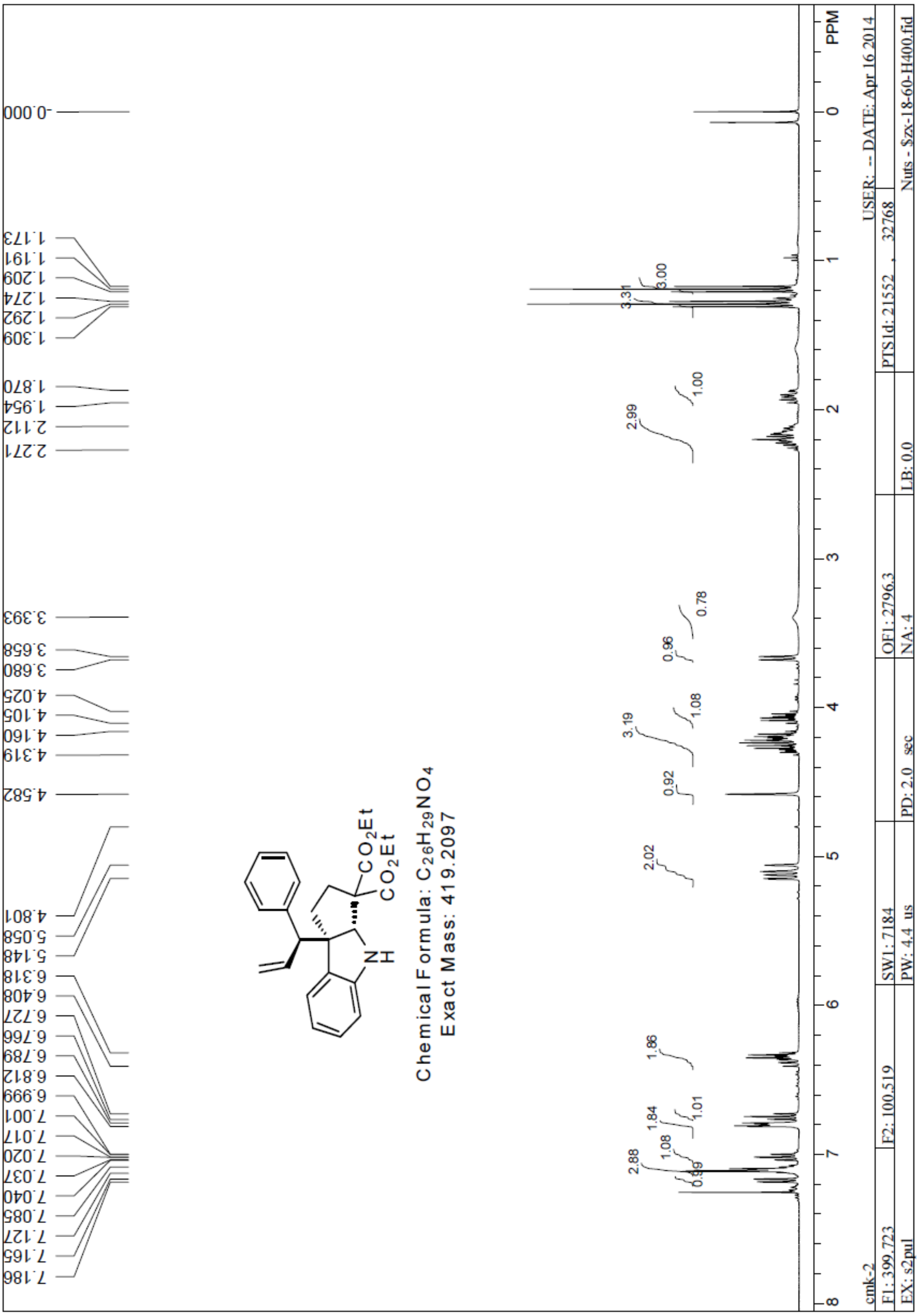

Compound **4oa**'s ^{13}C NMR Spectra

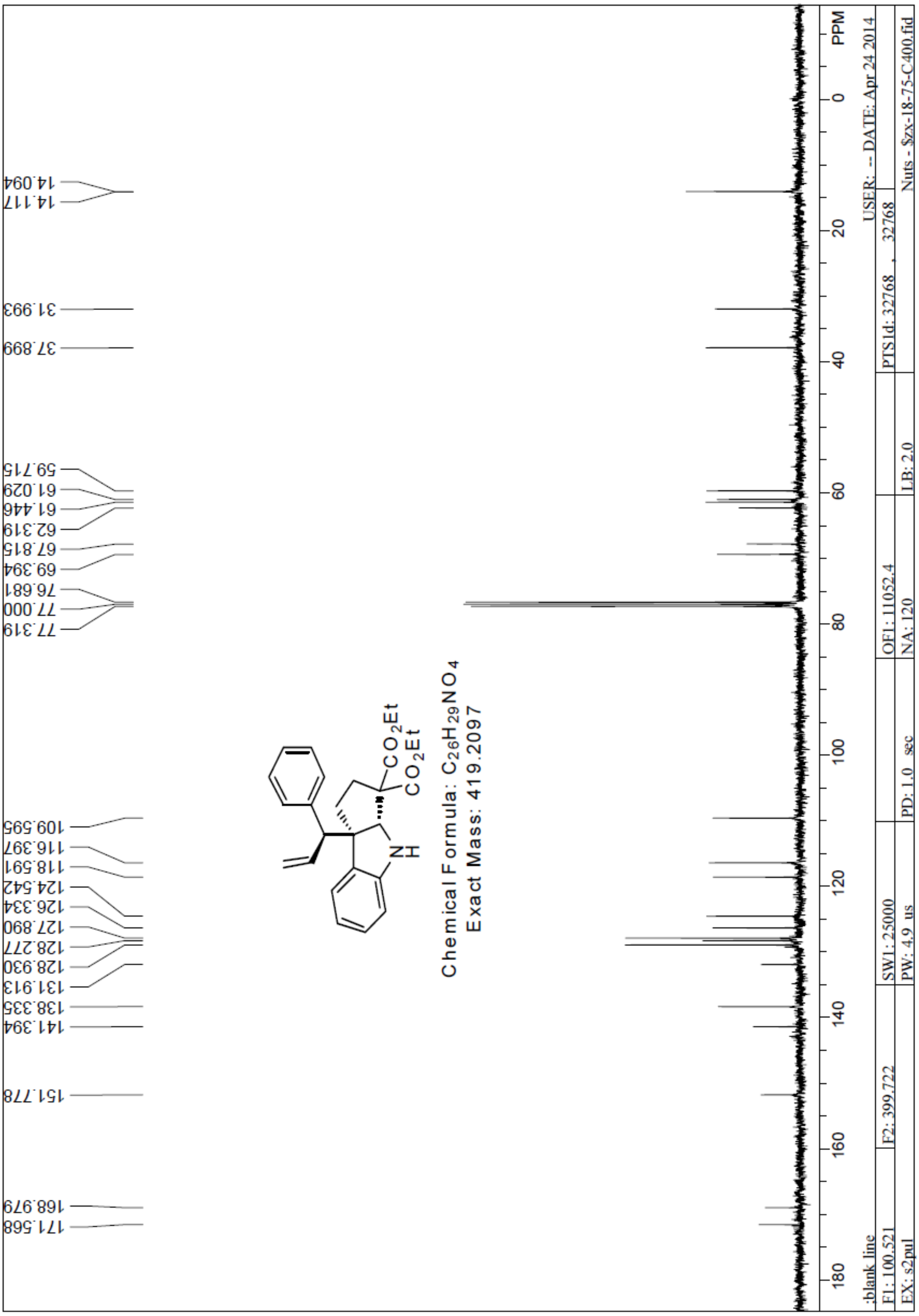

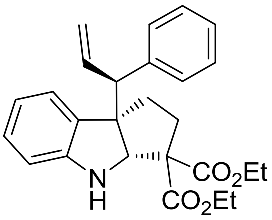

4oa

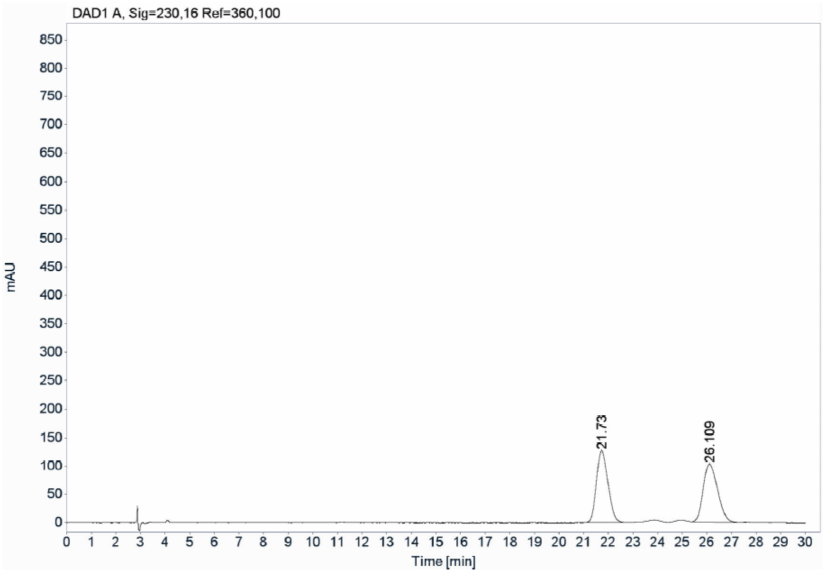

Signal: DAD1 A, Sig=230,16 Ref=360,100

RT [min]	Type	Width [min]	Area	Height	Area%
21.730	BB	0.4728	4132.1968	127.2657	49.8984
26.109	BB	0.5366	4149.0244	102.8496	50.1016
		Sum	8281.2212		

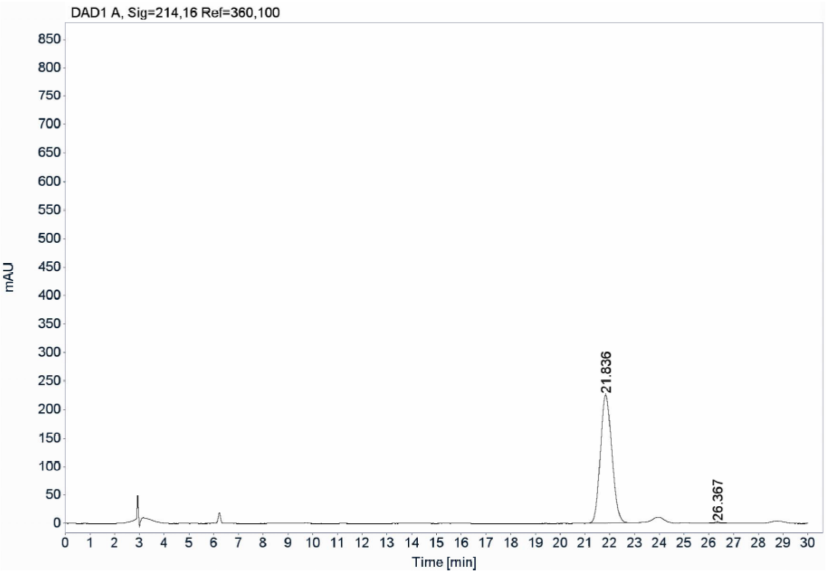

Signal: DAD1 A, Sig=214,16 Ref=360,100

RT [min]	Type	Width [min]	Area	Height	Area%
21.836	VB	0.4733	7285.8862	225.2725	99.0480
26.367	MM R	0.6248	70.0285	1.8679	0.9520
		Sum	7355.9147		

Compound **4pa**'s ^{1}H NMR Spectra

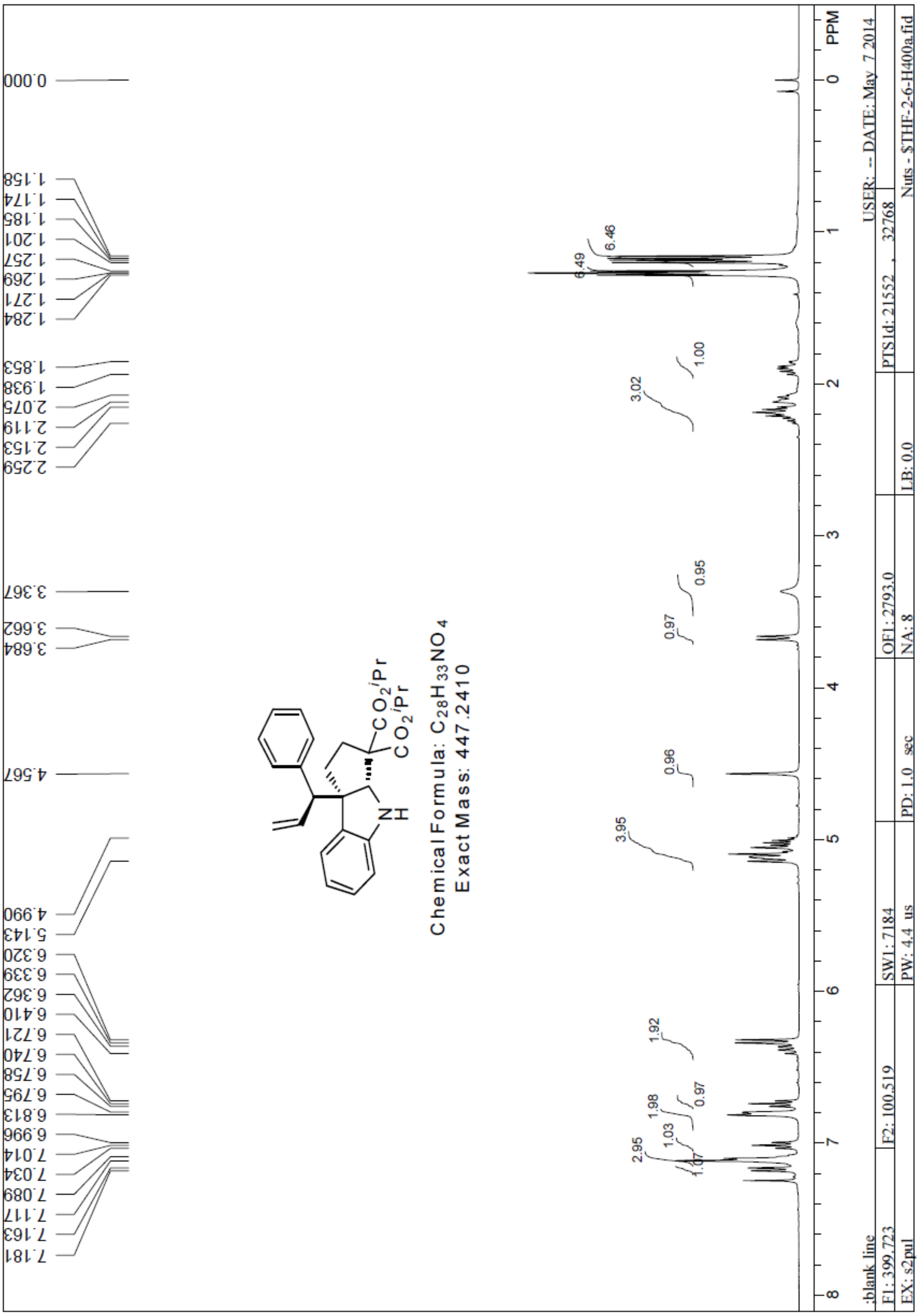

Compound **4pa**'s ^{13}C NMR Spectra

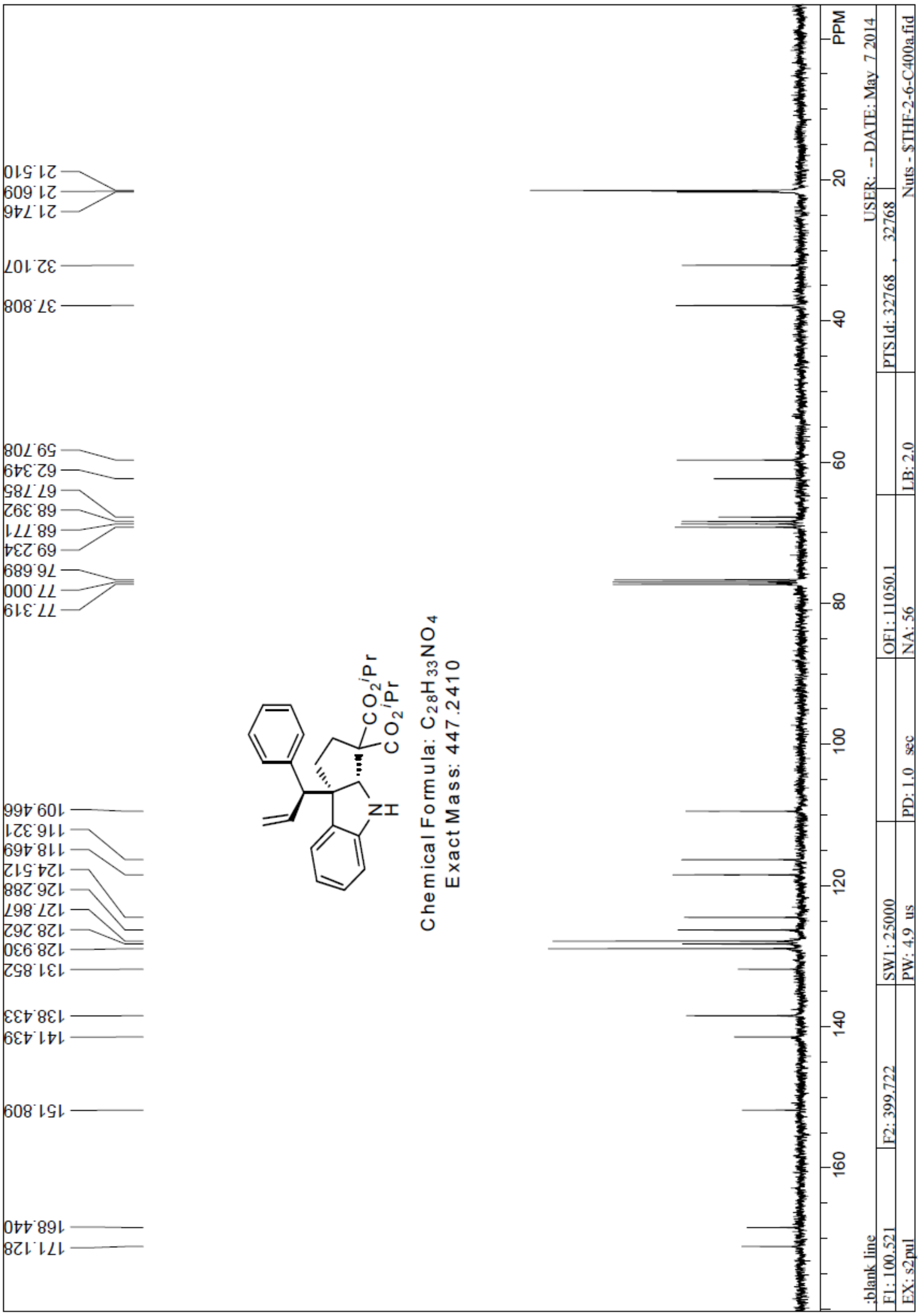

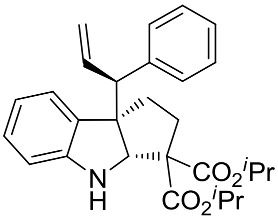

4pa

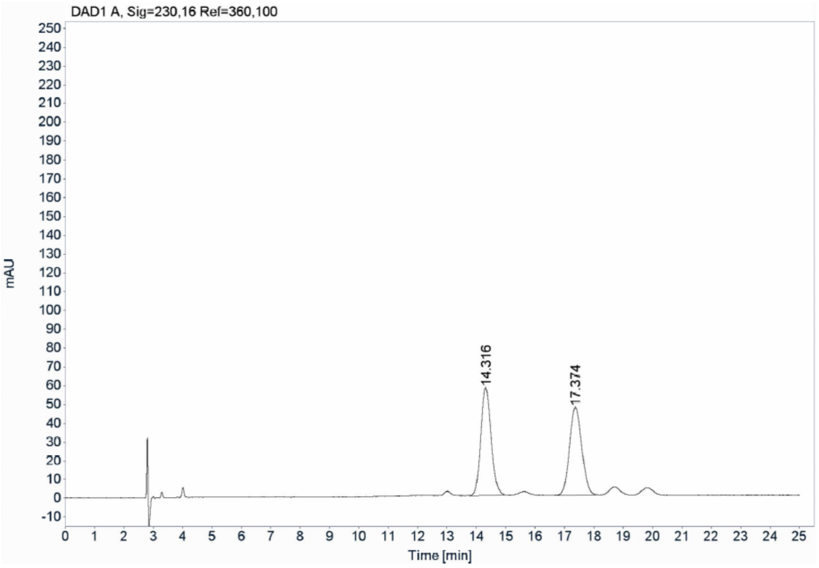

Signal: DAD1 A, Sig=230,16 Ref=360,100

RT [min]	Type	Width [min]	Area	Height	Area%
14.316	BB	0.3506	1403.9894	57.3497	50.9972
17.374	BV	0.4111	1349.0835	46.6965	49.0028
		Sum	2753.0729		

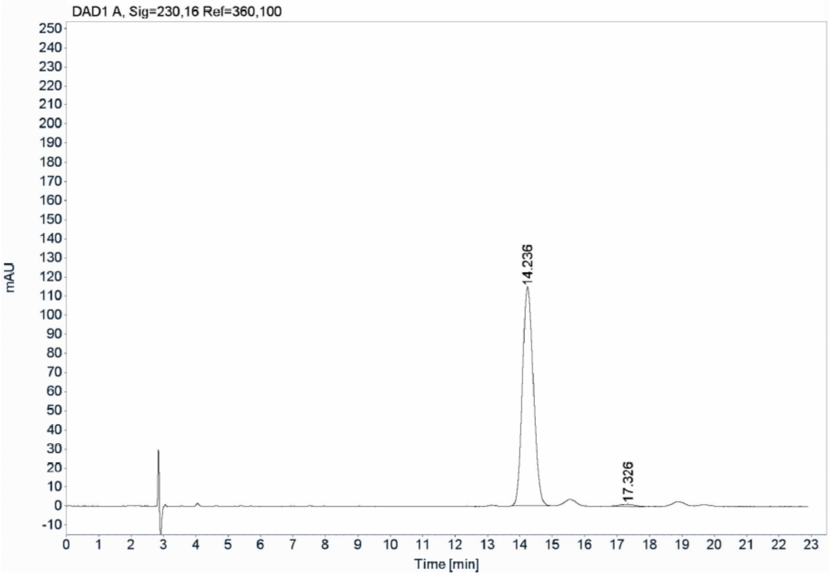

Signal: DAD1 A, Sig=230,16 Ref=360,100

RT [min]	Type	Width [min]	Area	Height	Area%
14.236	BB	0.3664	2701.6035	114.4654	98.5628
17.326	MM R	0.5300	39.3942	1.2387	1.4372
		Sum	2740.9978		

Compound **4qa**’s ^{1}H NMR Spectra

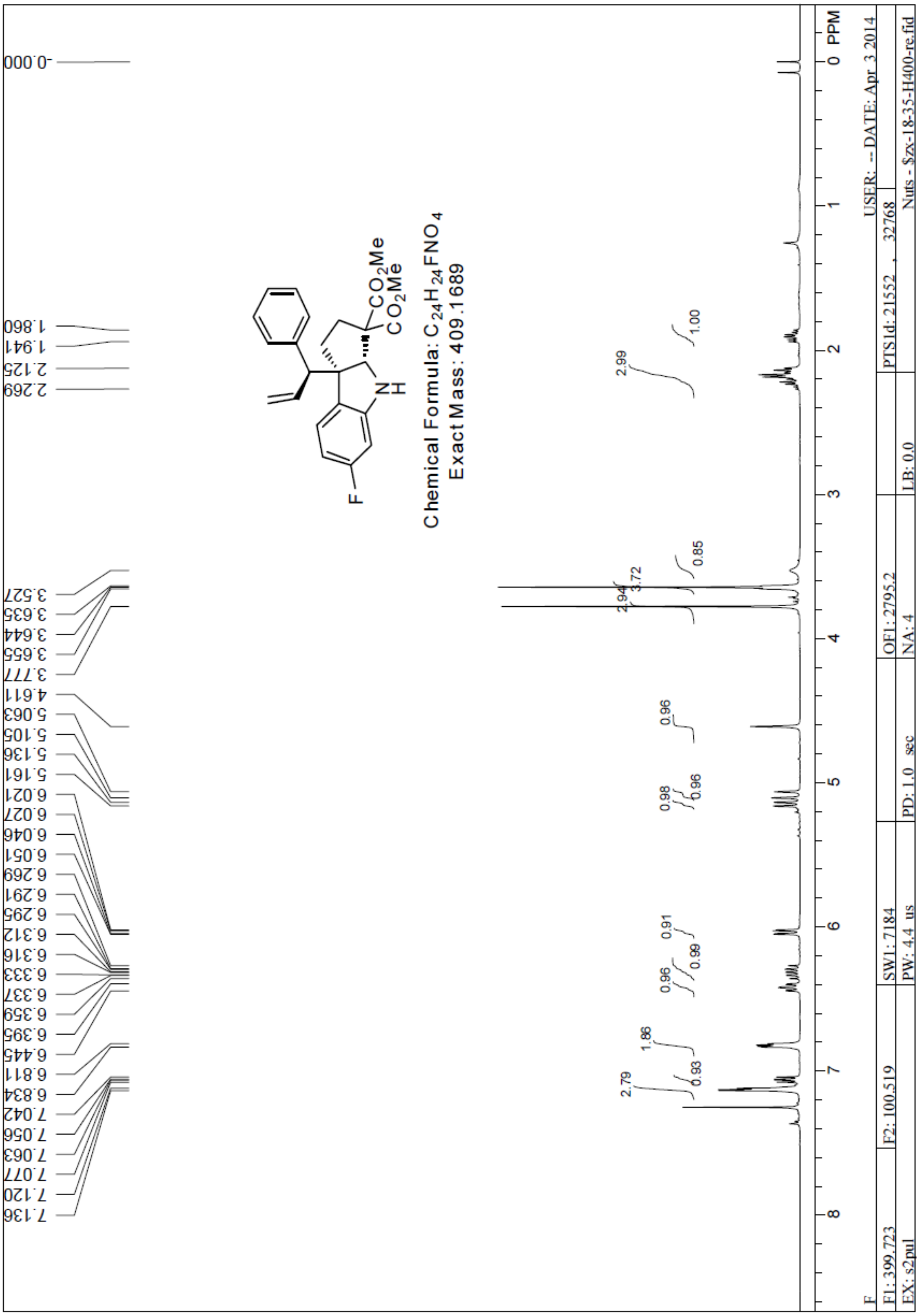

Compound **4qa**’s ^{13}C NMR Spectra

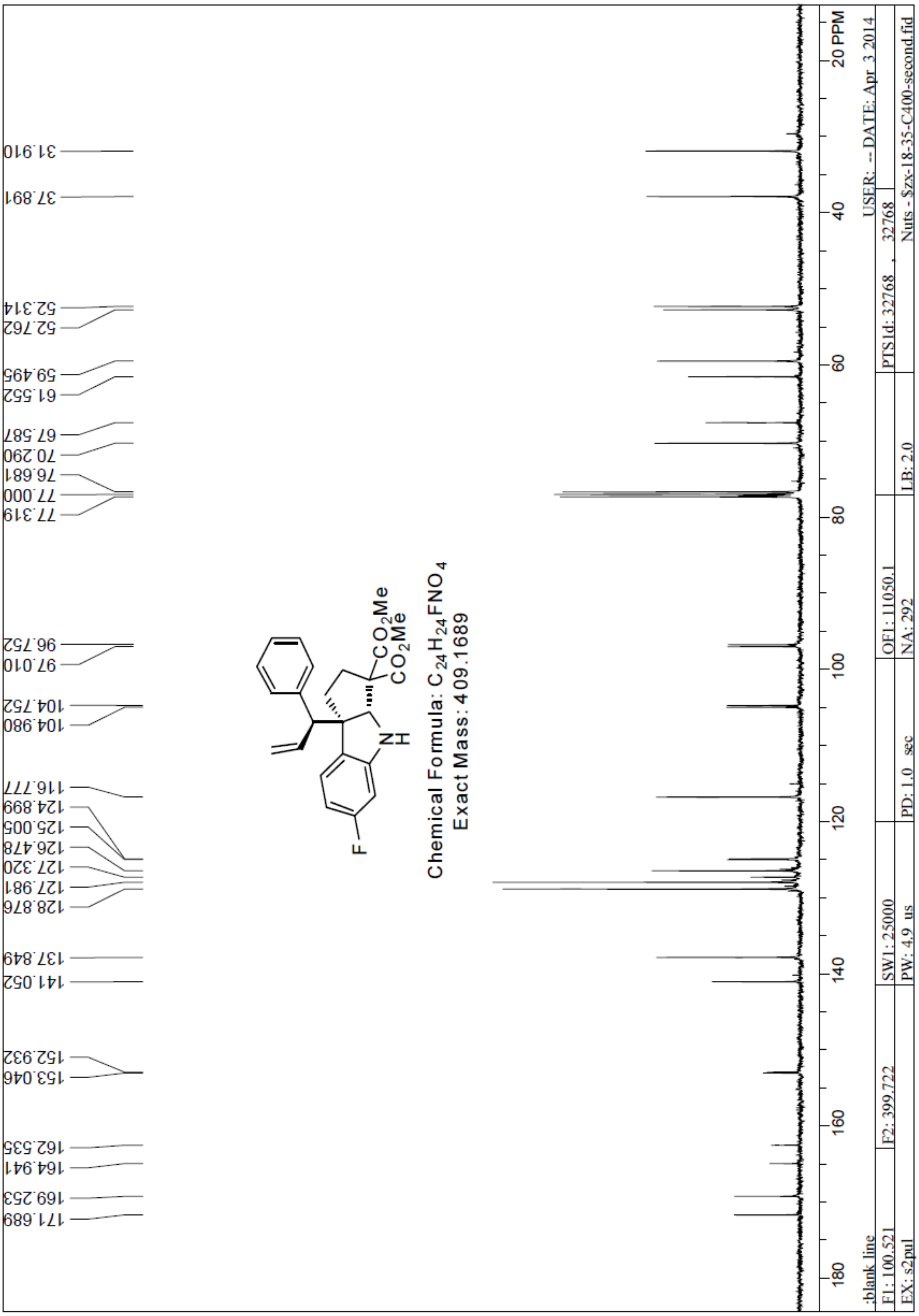

Compound **4qa**'s ^{19}F NMR Spectra

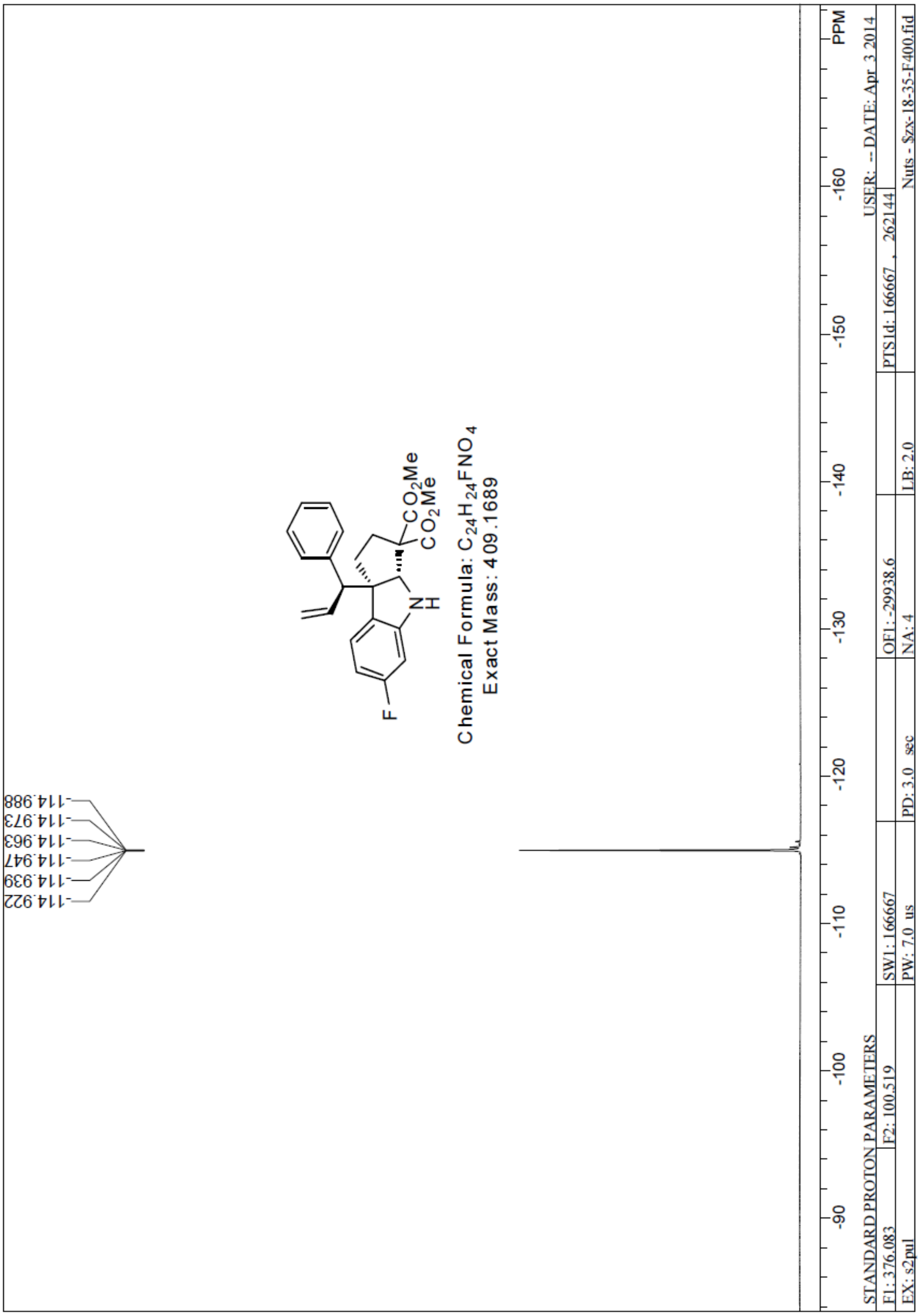

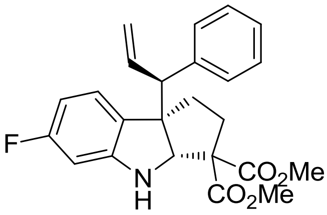

4qa

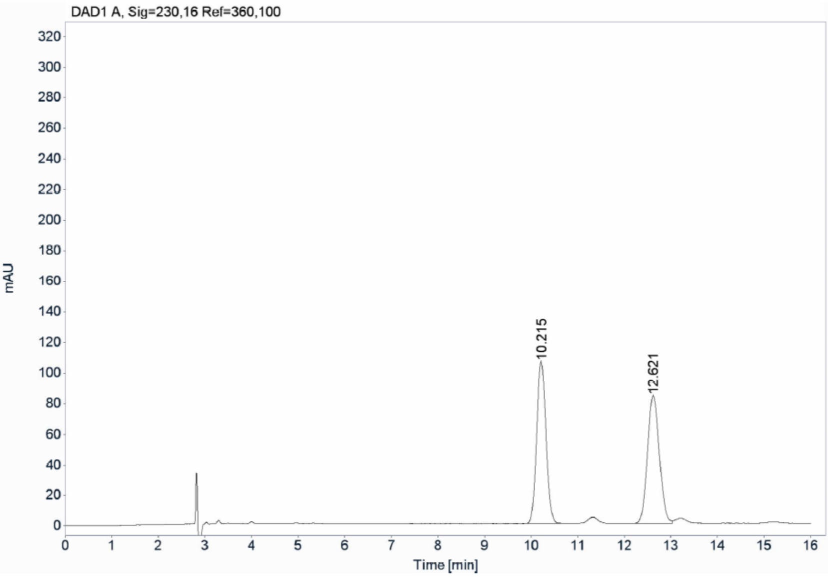

Signal: DAD1 A, Sig=230, 16 Ref=360, 100

RT [min]	Type	Width [min]	Area	Height	Area%
10.215	BB	0.2155	1473.0198	105.7437	49.8572
12.621	BV	0.2759	1481.4584	83.5192	50.1428
		Sum	2954.4781		

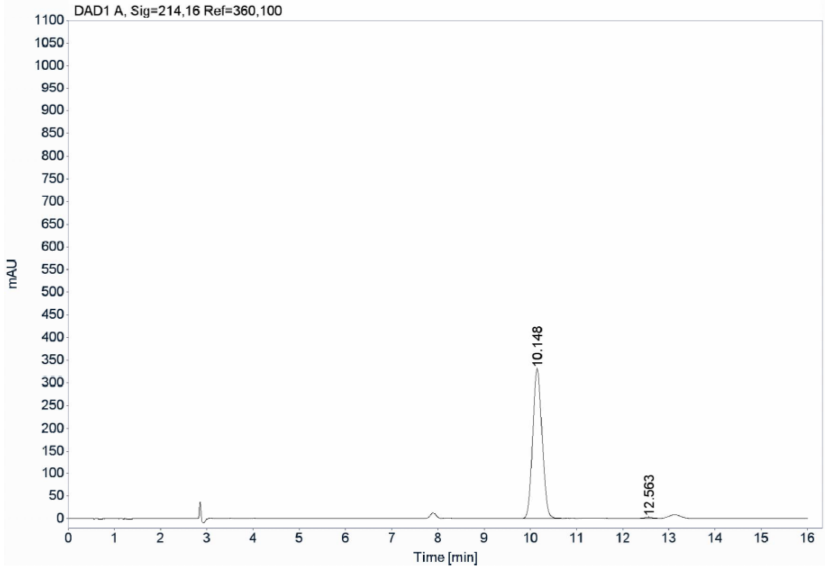

Signal: DAD1 A, Sig=214, 16 Ref=360, 100

RT [min]	Type	Width [min]	Area	Height	Area%
10.148	VB	0.2228	4695.8584	330.4548	99.4048
12.563	MM R	0.2486	28.1167	1.8852	0.5952
		Sum	4723.9751		

Compound *cis*-**4ra**'s ^{1}H NMR Spectra

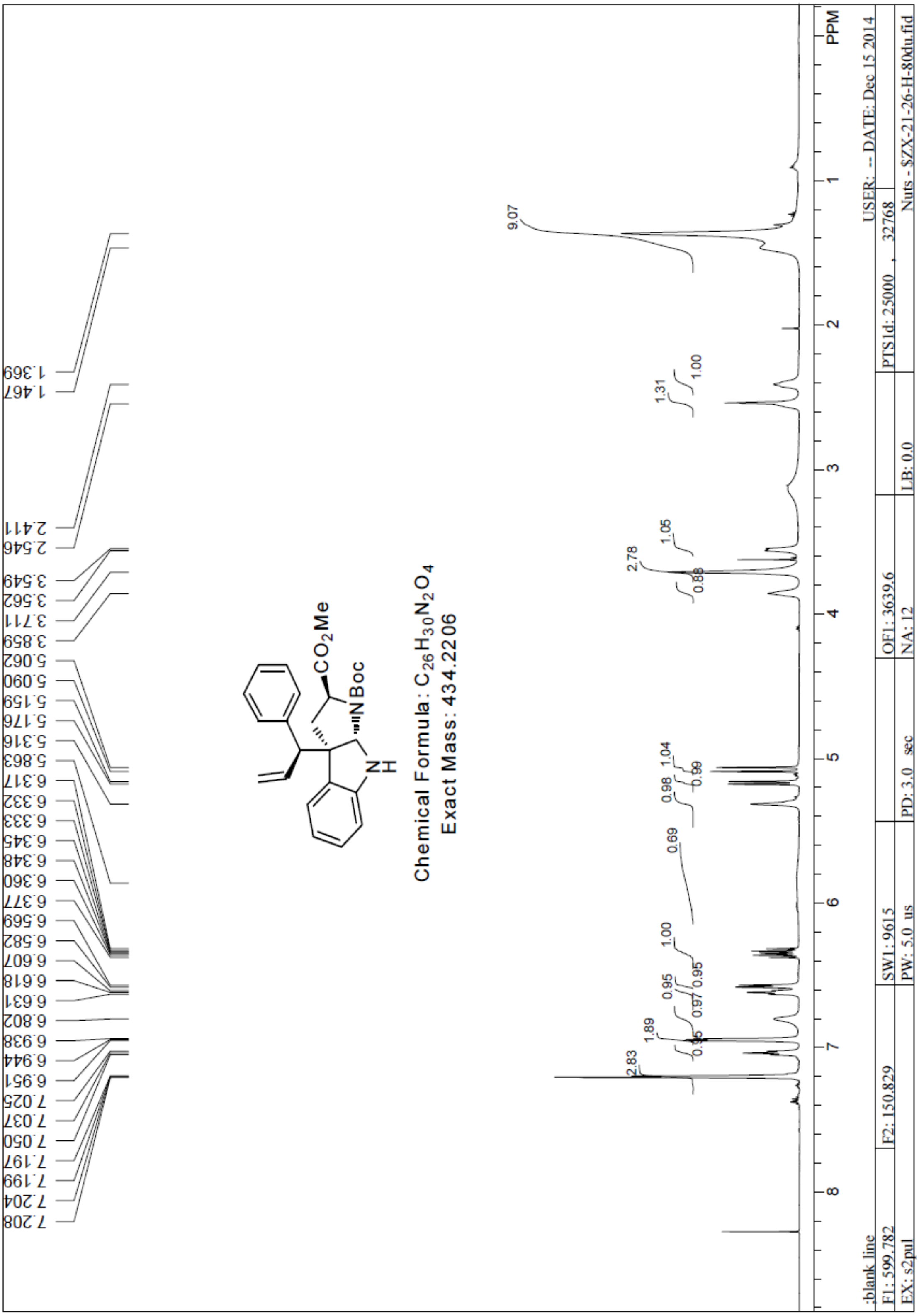

Compound *cis*-**4ra**’s ^{13}C NMR Spectra

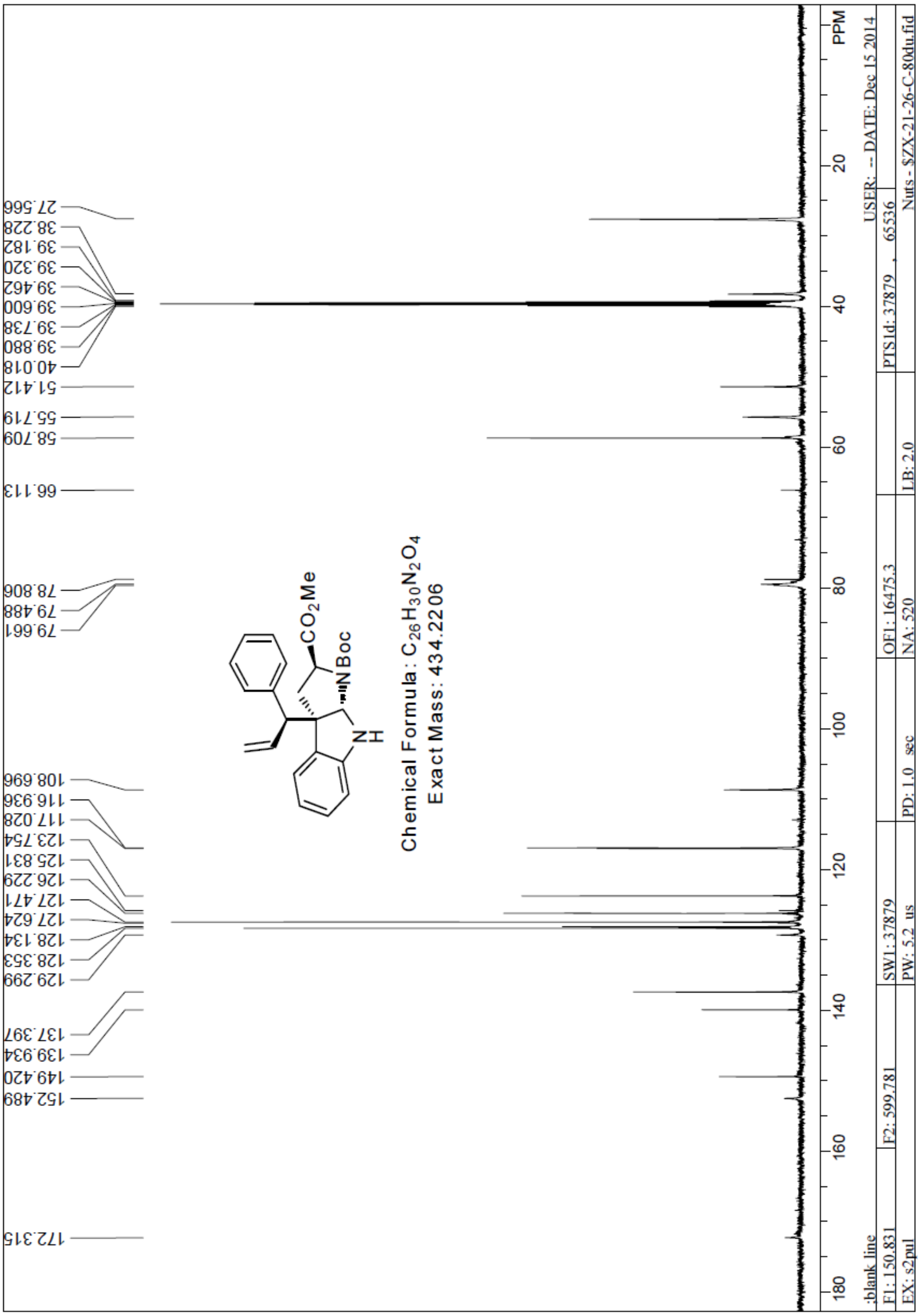

Compound *cis*-**4ra**'s ^{1}H-^{1}H COSY Spectra

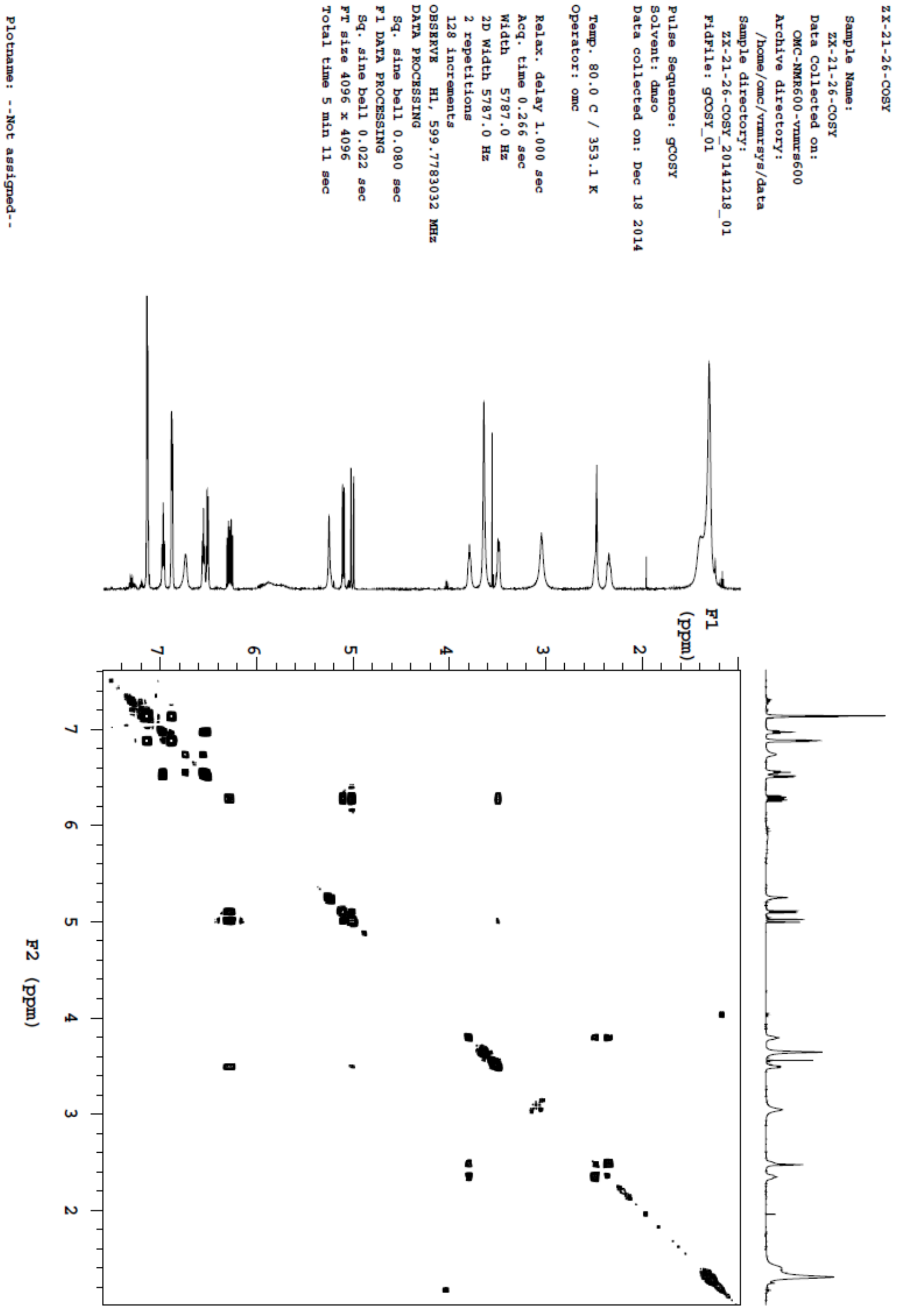

Compound *cis*-**4ra**’s 2D-NOESY Spectra

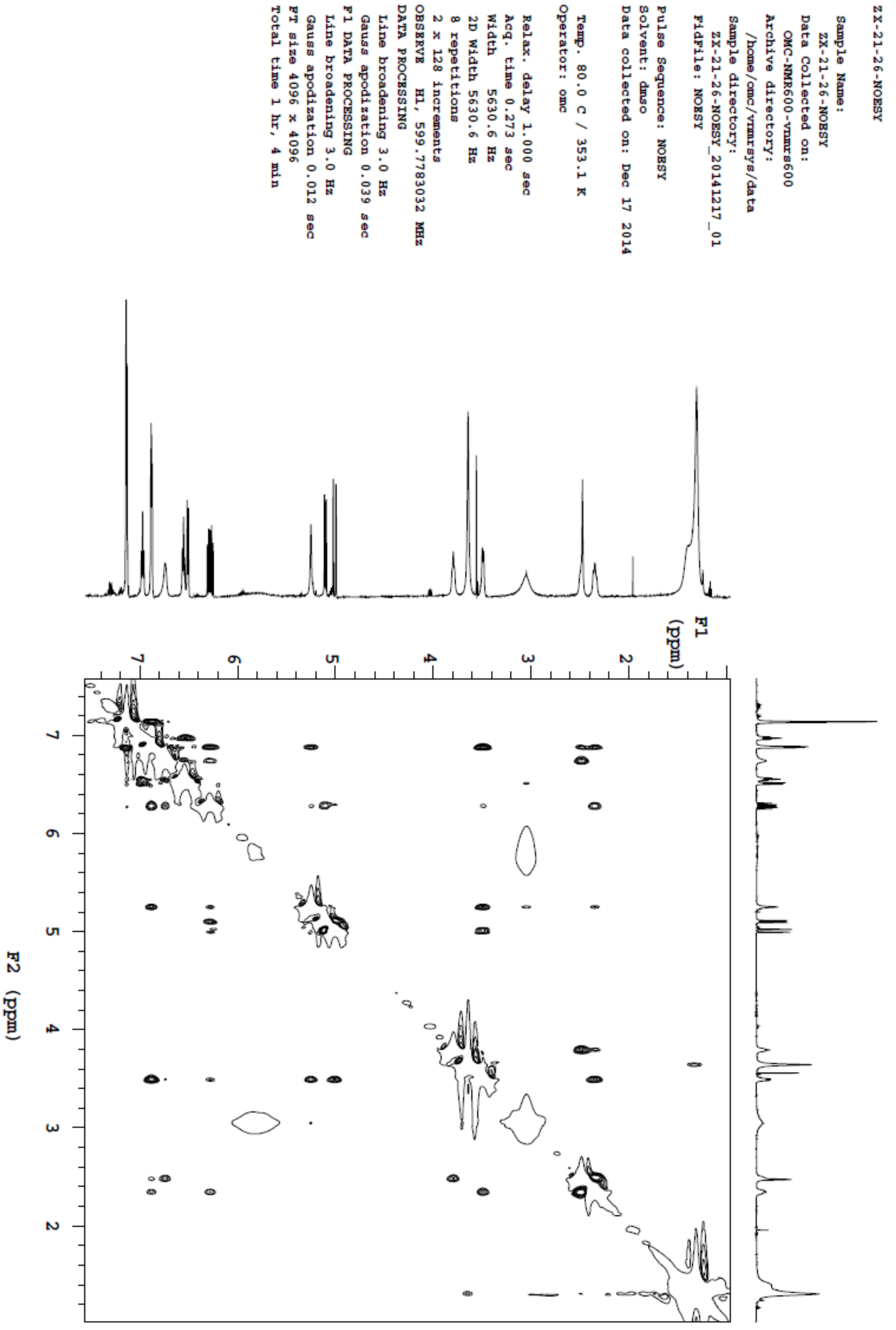

Compound *trans*-**4ra**'s ^{1}H NMR Spectra

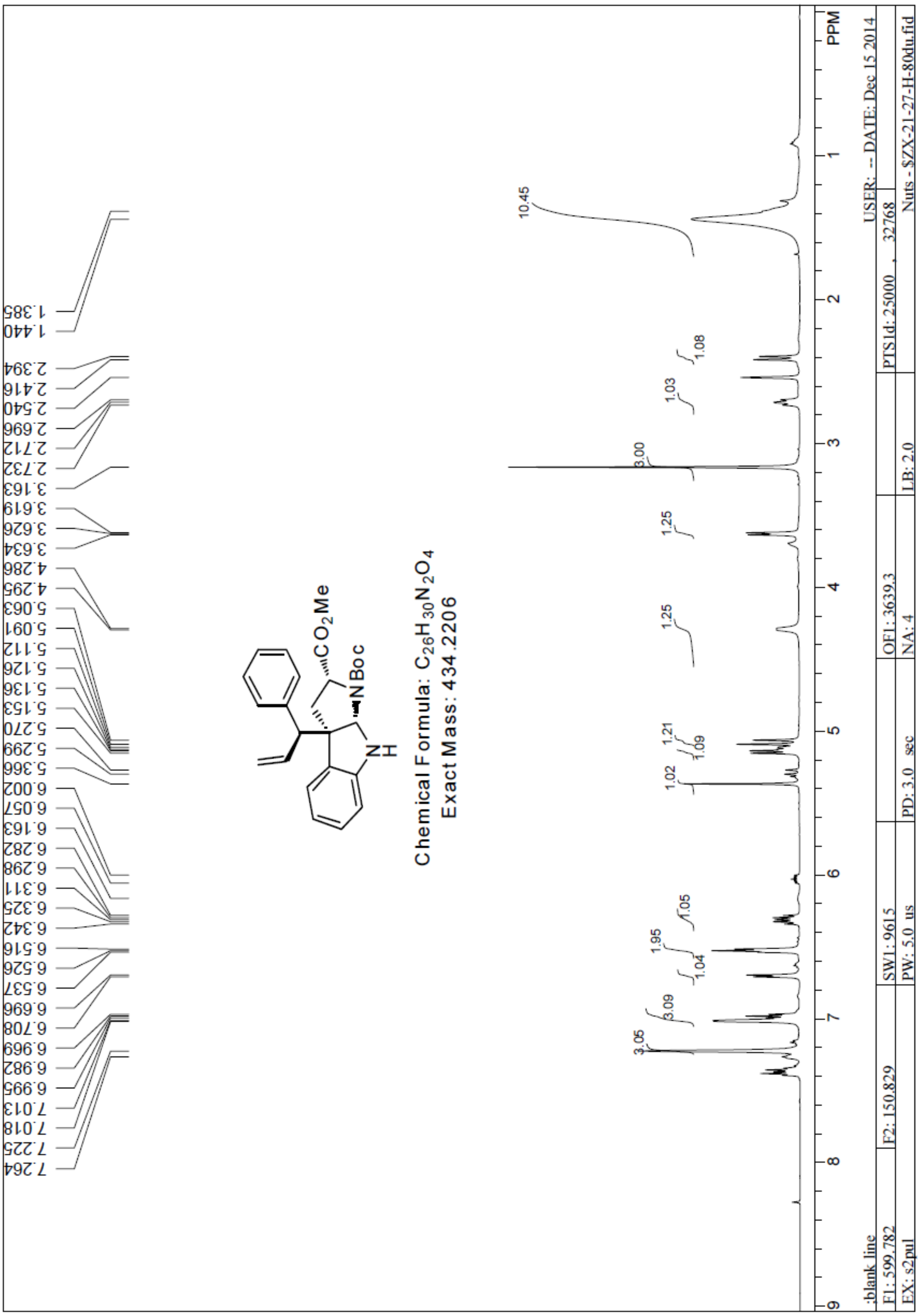

Compound *trans*-**4ra**'s ^{13}C NMR Spectra

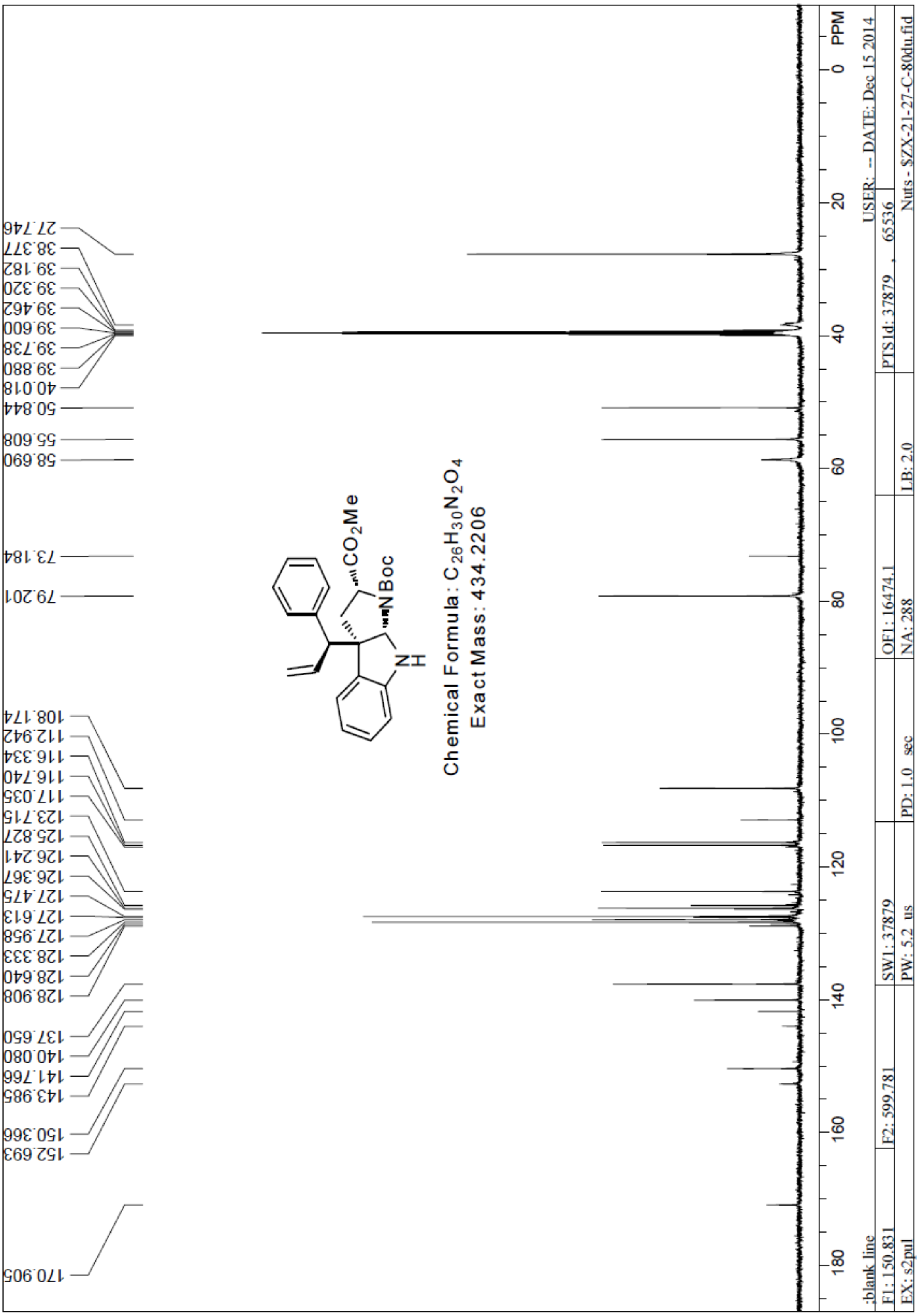

Compound *trans*-**4ra**'s ^{1}H-^{1}H COSY Spectra

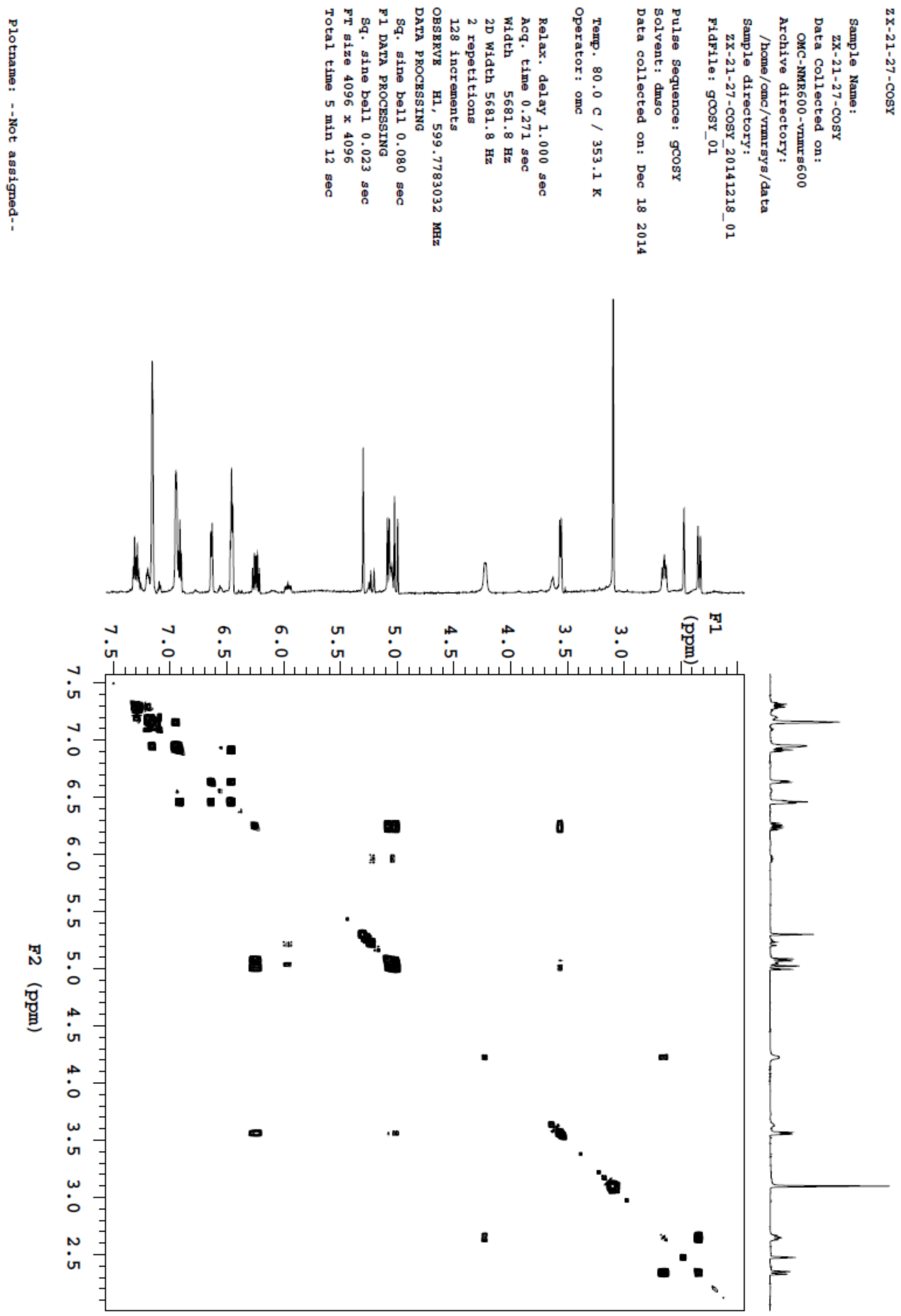

Compound *trans*-**4ra**'s 2D-NOESY Spectra

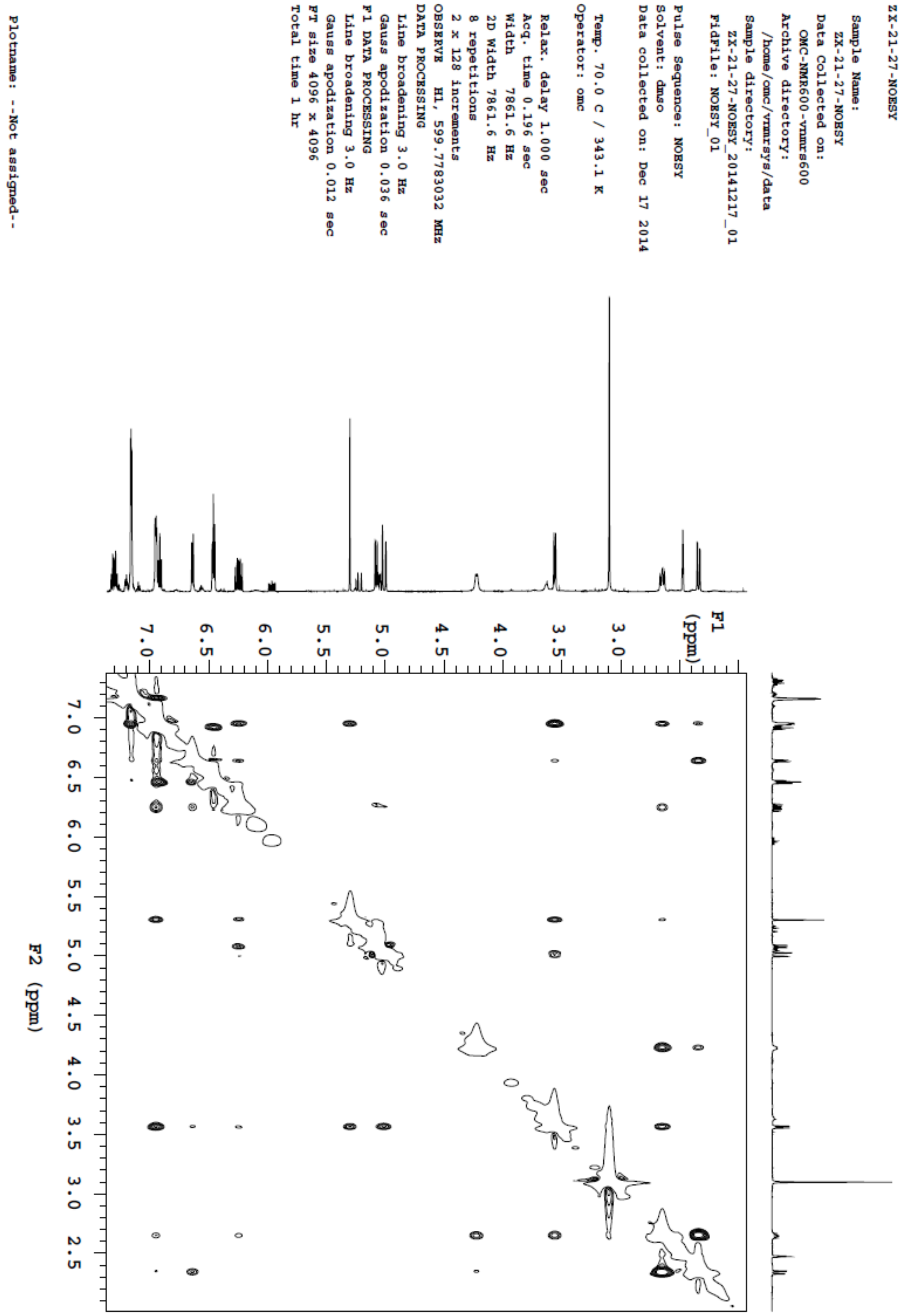

Compound **7**'s ^{1}H NMR Spectra

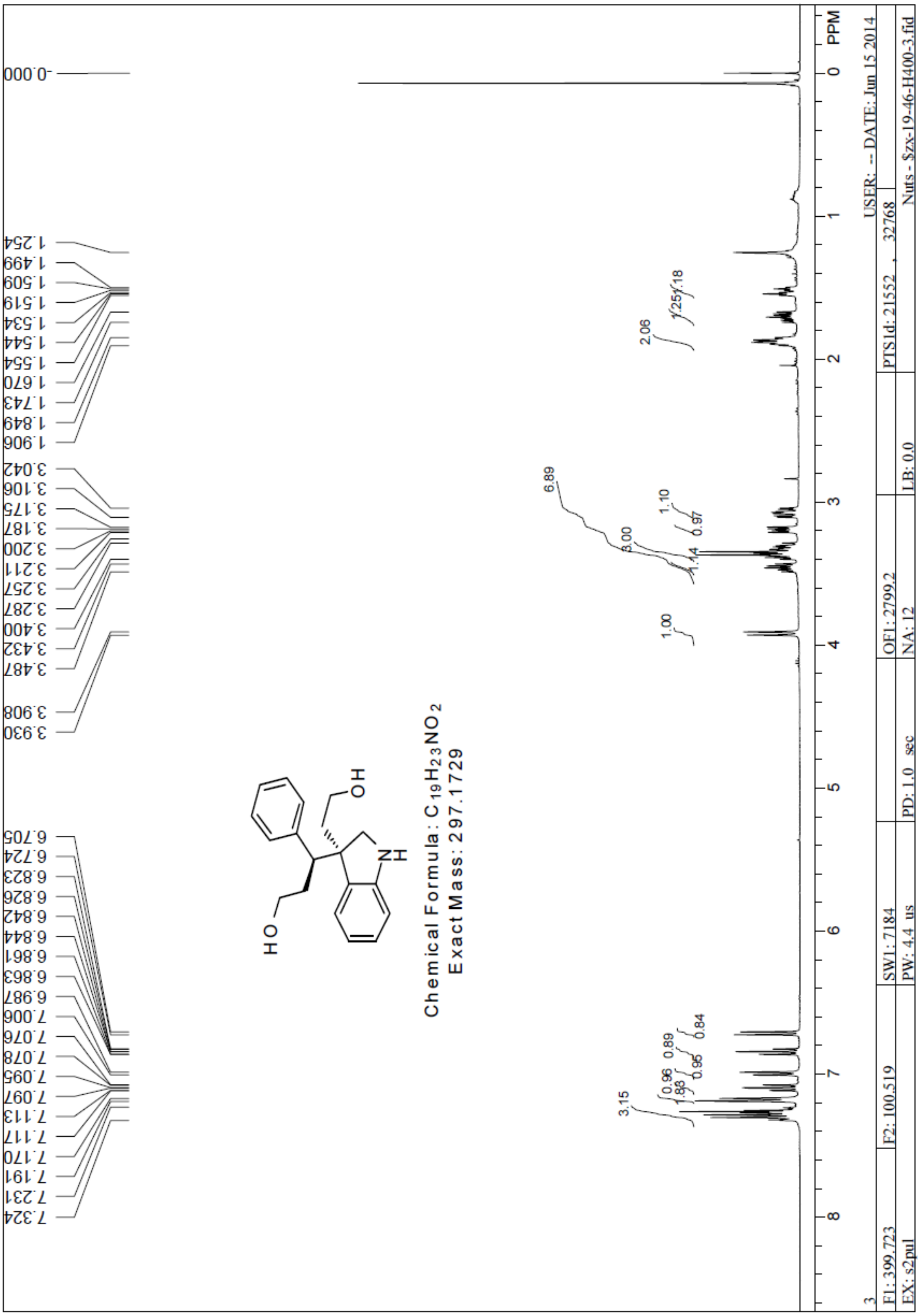

Compound **7**’s ^{13}C NMR Spectra

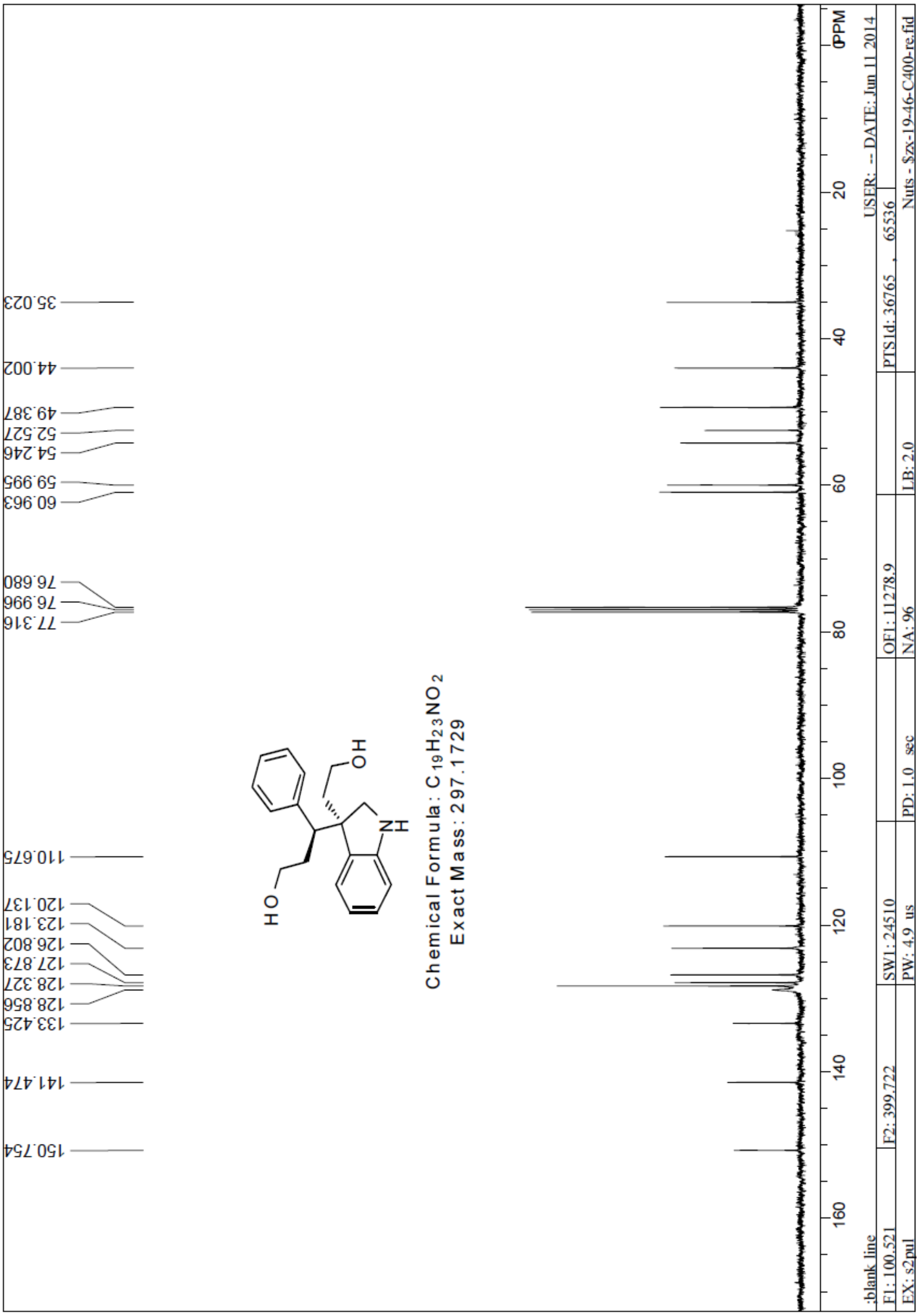

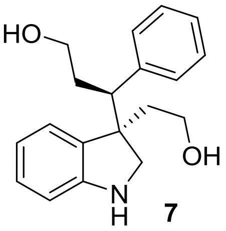

7

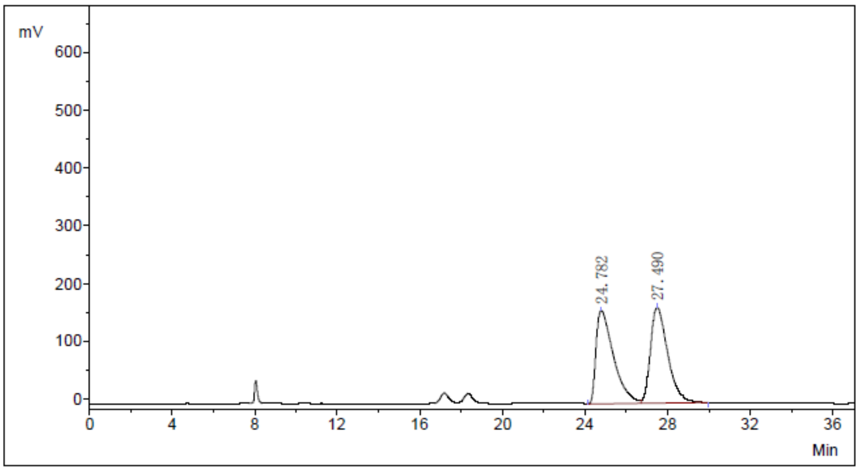

No.	PeakNo	ID. Name	R. Time	PeakHeight	PeakArea	PerCent
1	1	Unknown	24.782	161251.8	9465163.5	49.6670
2	2	Unknown	27.490	165357.4	9592086.8	50.3330
Total				326609.2	19057250.4	100.0000

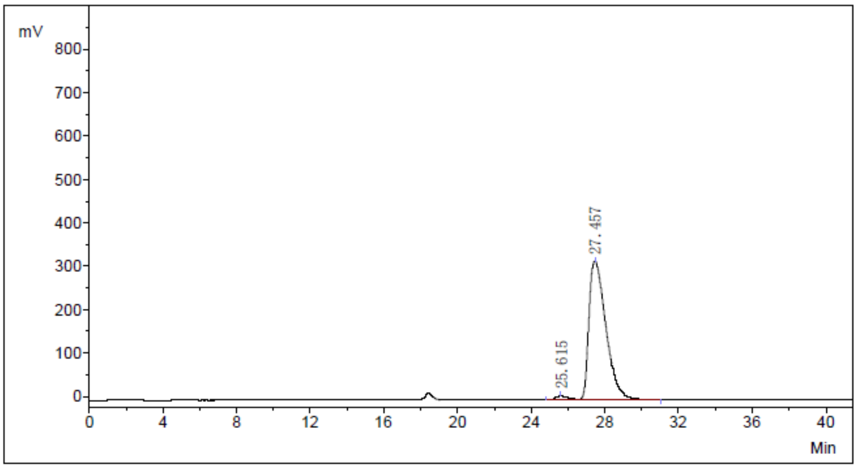

No.	PeakNo	ID. Name	R. Time	PeakHeight	PeakArea	PerCent
1	1	Unknown	25.615	9945.7	527741.2	2.5085
2	2	Unknown	27.457	320568.4	20510002.0	97.4915
Total				330514.1	21037743.2	100.0000

Compound **8**'s ^{1}H NMR Spectra

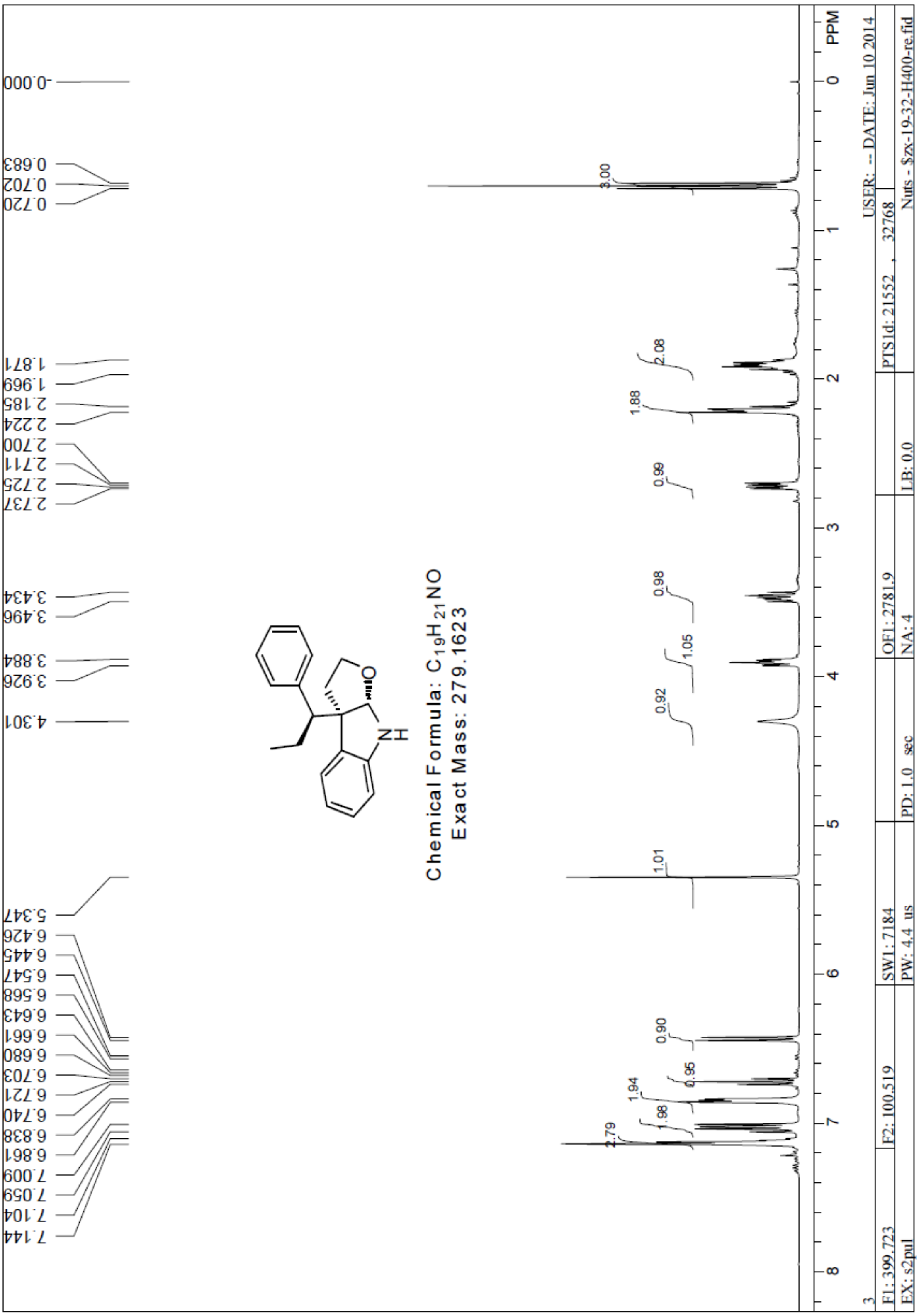

Compound **8**’s ^{13}C NMR Spectra

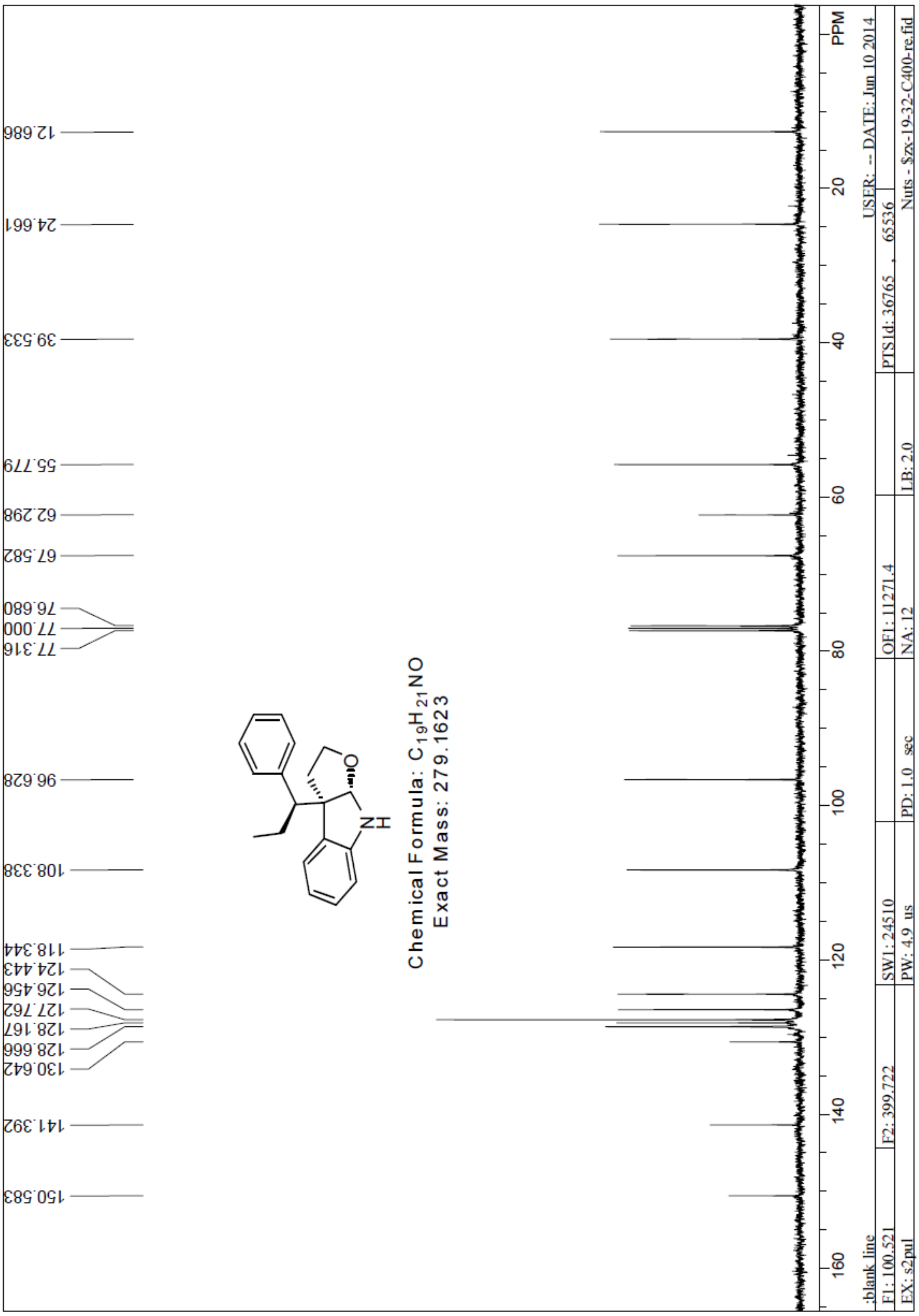

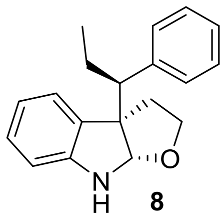

8

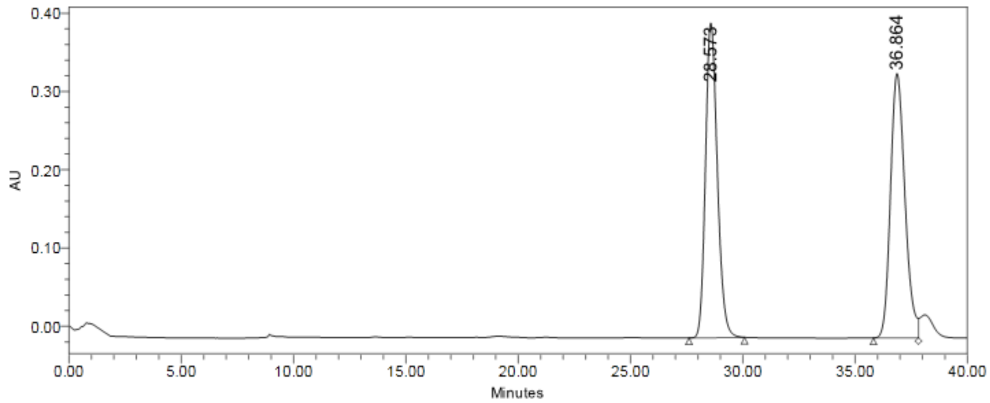

Channel: W2489 ChA; Processed Channel: W2489 ChA 254nm; Injection: 1; Date Acquired: 6/10/2014 7:38:38 AM CST; Result Id: 1246; Processing Method: 1

Peak Name:

	Injection	RT	Area	% Area	Height
1	1	36.864	15085666	50.58	337880
2	1	28.573	14738620	49.42	402181
Mean		32.719			
Std. Dev.		5.863			
% RSD		17.92			

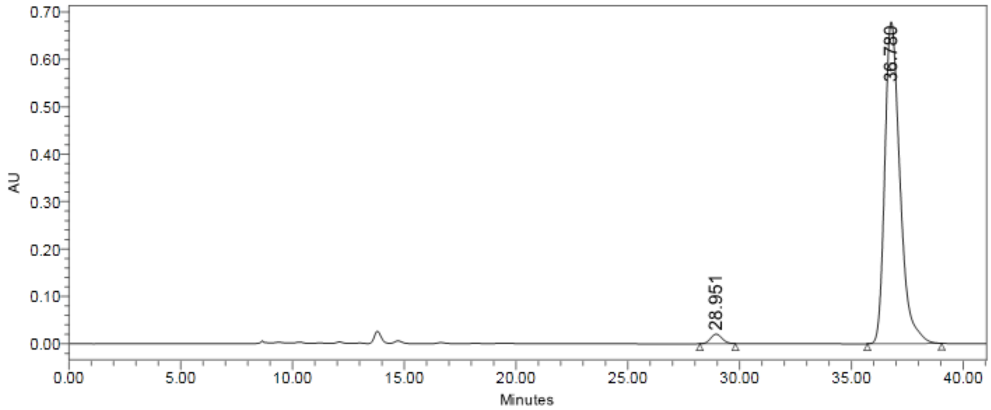

Channel: W2489 ChA; Processed Channel: W2489 ChA 254nm; Injection: 1; Date Acquired: 6/10/2014 8:19:04 AM CST; Result Id: 1191; Processing Method: 1

Peak Name:

	Injection	RT	Area	% Area	Height
1	1	36.780	32067662	97.78	678875
2	1	28.951	728629	2.22	20326
Mean		32.866			
Std. Dev.		5.536			
% RSD		16.84			

Compound **9**'s ^{1}H NMR Spectra

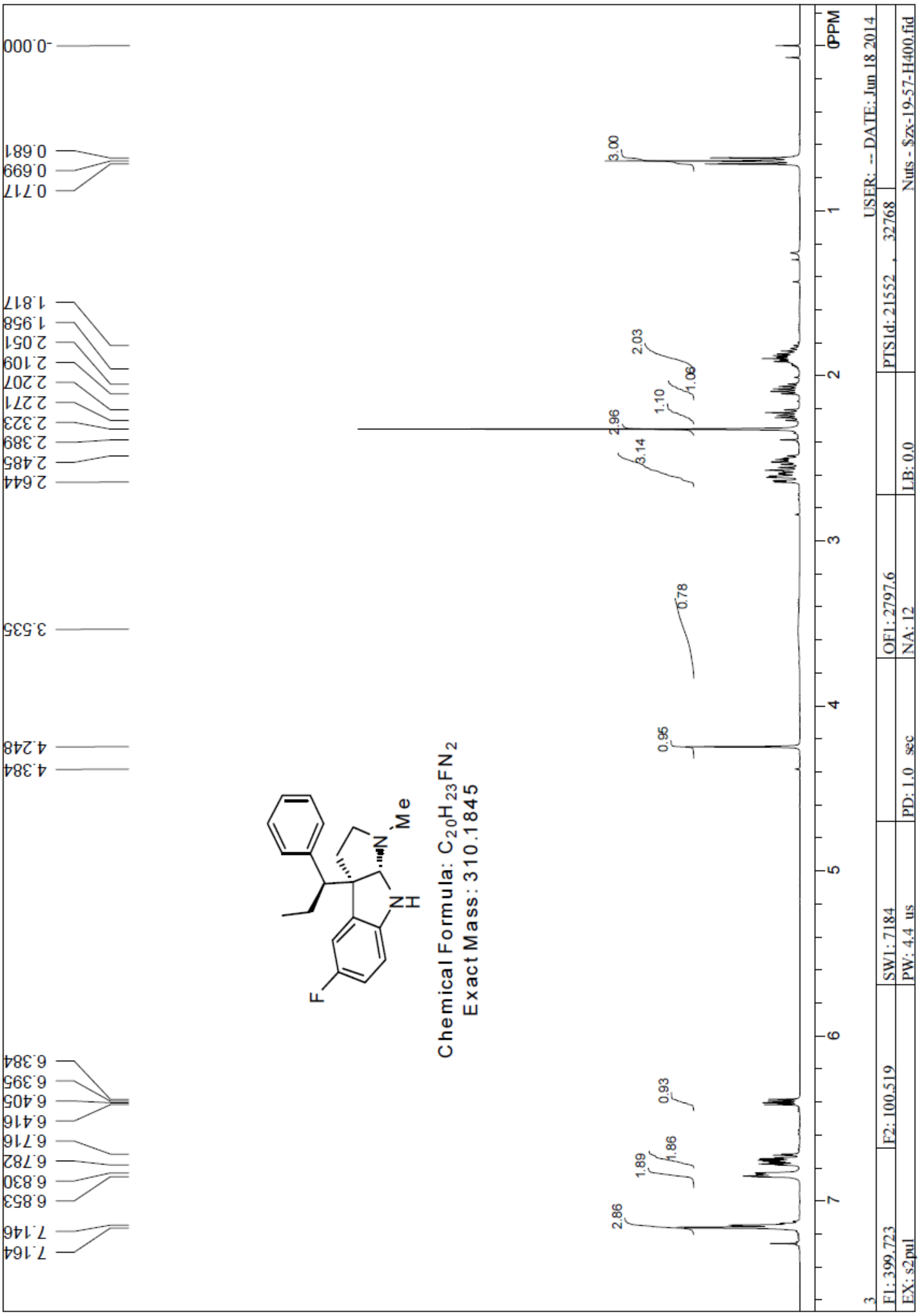

Compound **9**'s ^{13}C NMR Spectra

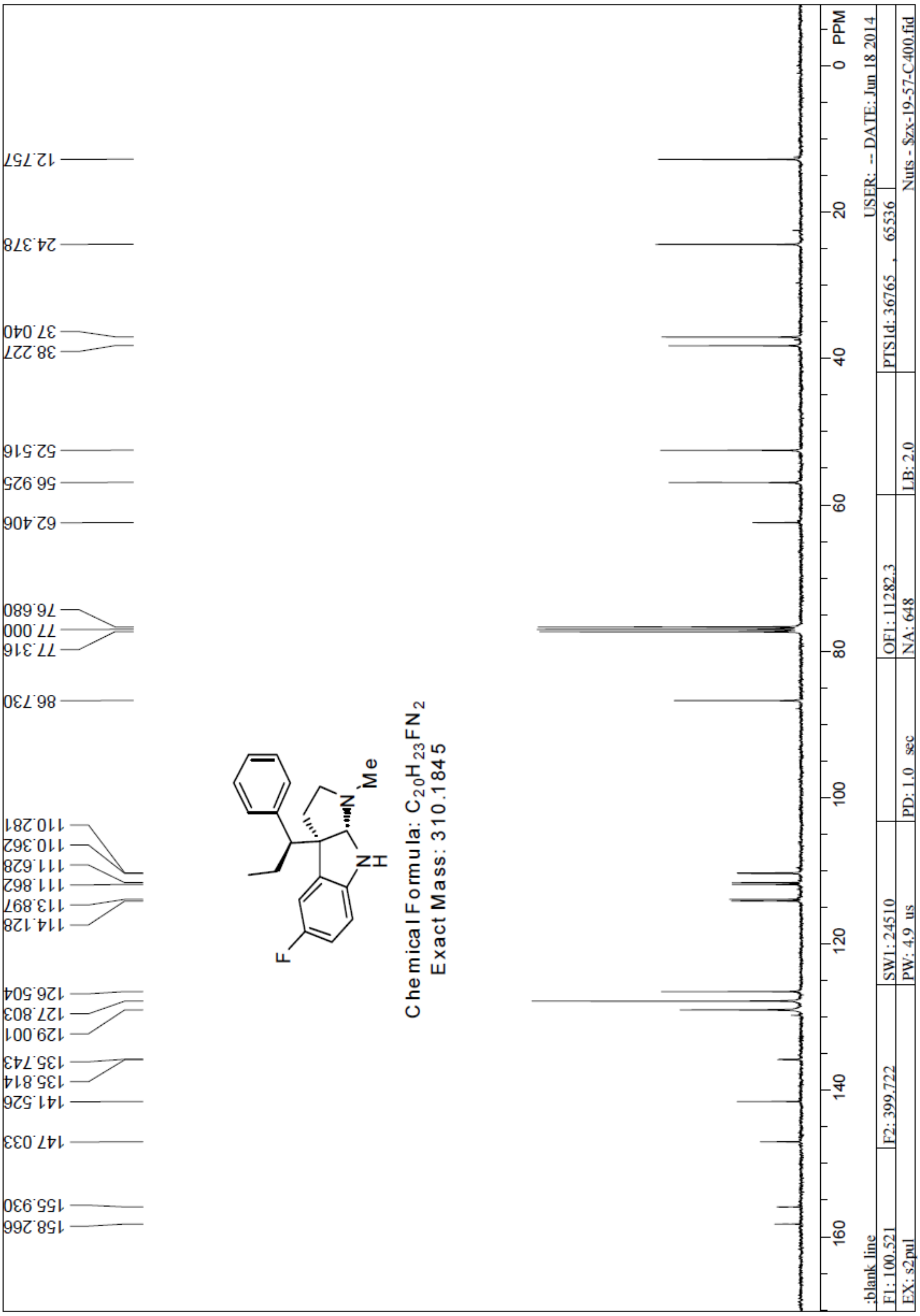

Compound **9**'s ^{19}F NMR Spectra

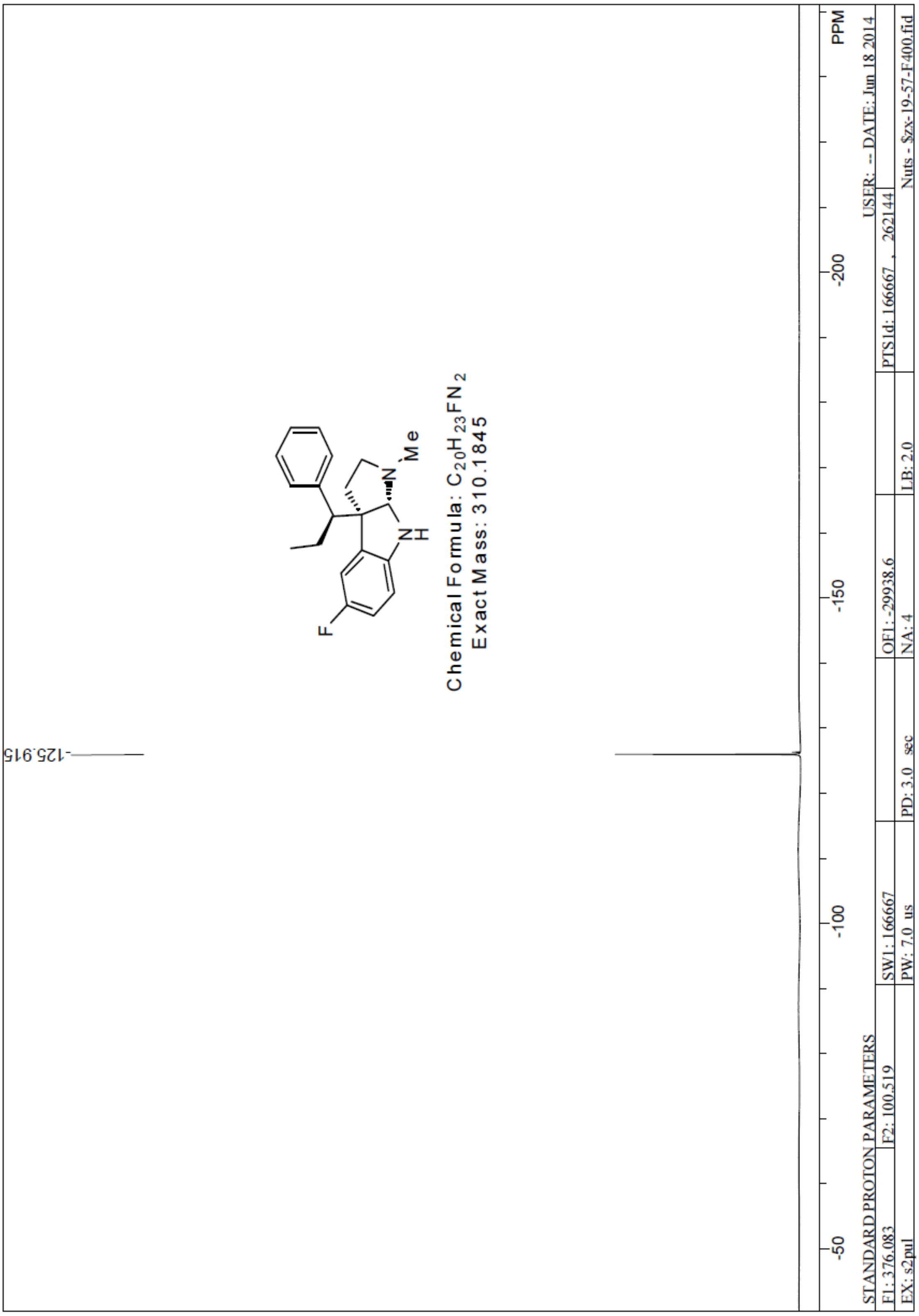

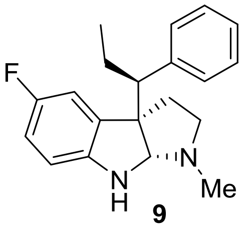

9

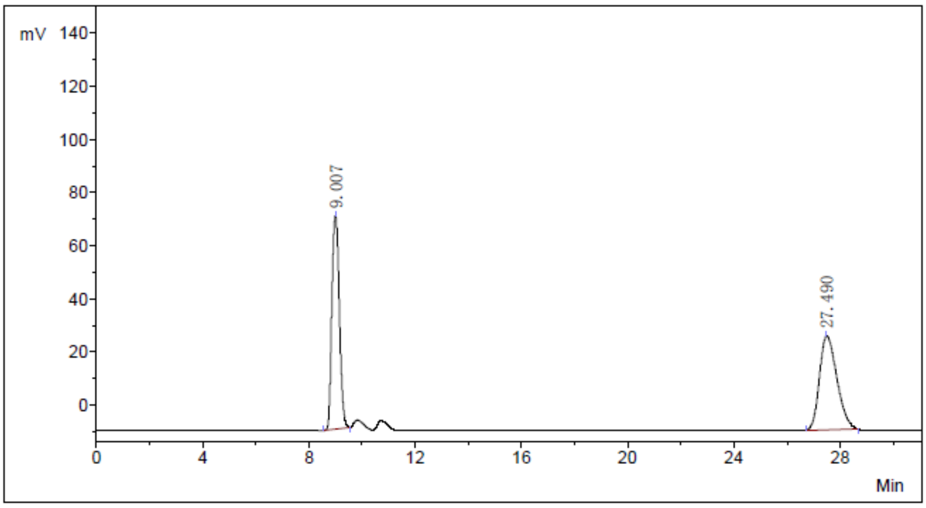

No.	PeakNo	ID.Name	R.Time	PeakHeight	PeakArea	PerCent
1	1	Unknown	9.007	80213.0	1582908.0	49.5378
2	2	Unknown	27.490	35356.8	1612447.3	50.4622
Total				115569.8	3195355.3	100.0000

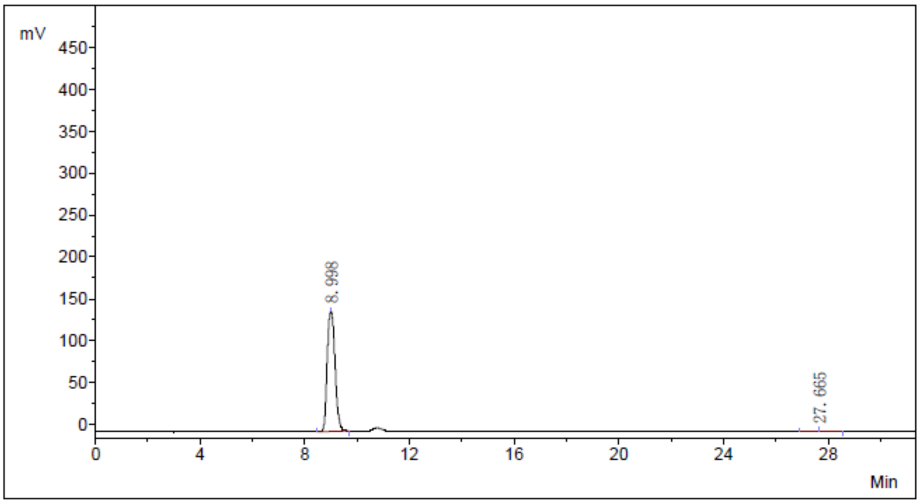

No.	PeakNo	ID.Name	R.Time	PeakHeight	PeakArea	PerCent
1	1	Unknown	8.998	144005.0	3001531.3	98.9876
2	2	Unknown	27.665	647.4	30697.9	1.0124
Total				144652.4	3032229.2	100.0000

Compound **6**'s ^{1}H NMR Spectra

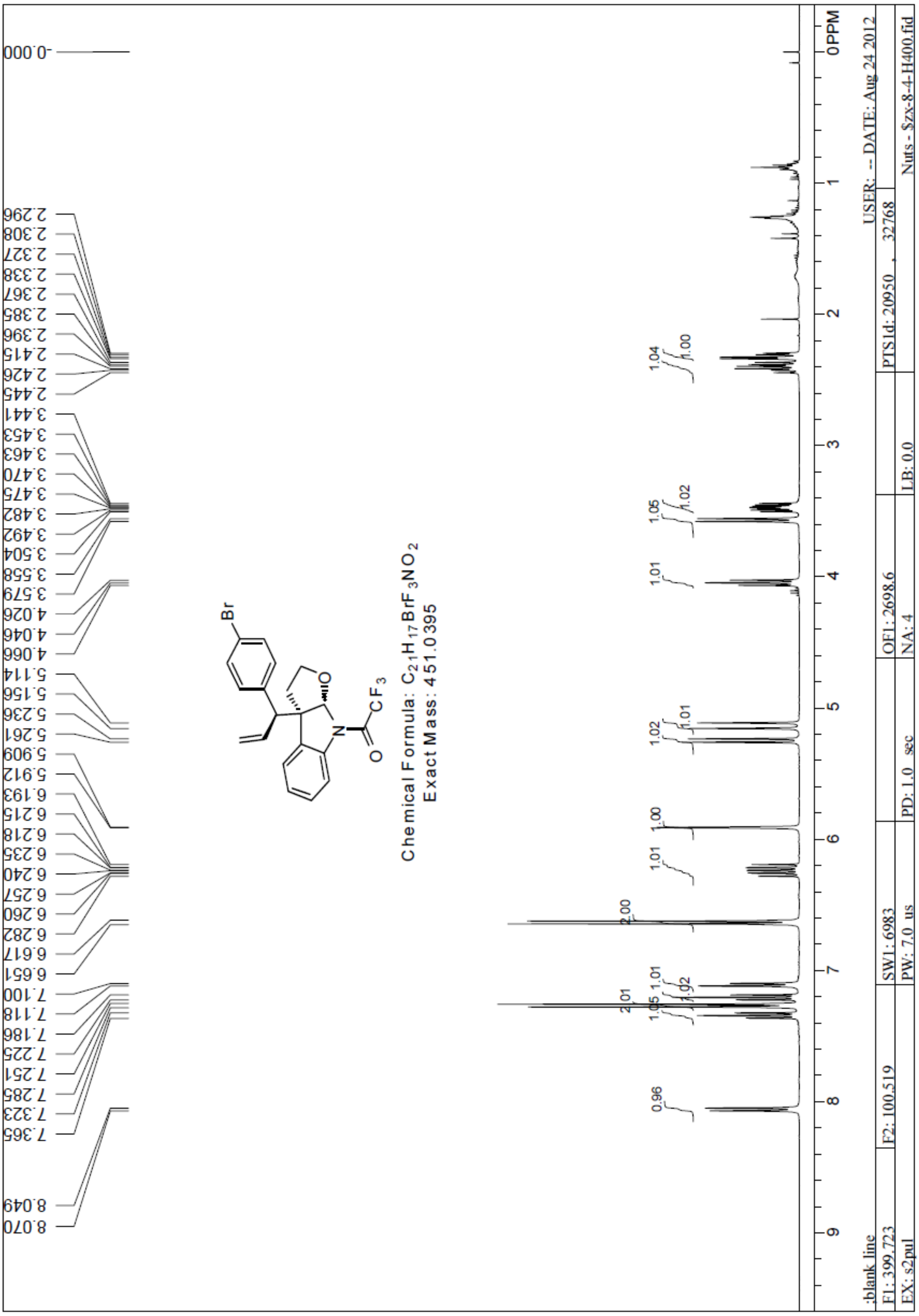

Compound **6**’s ^{13}C NMR Spectra

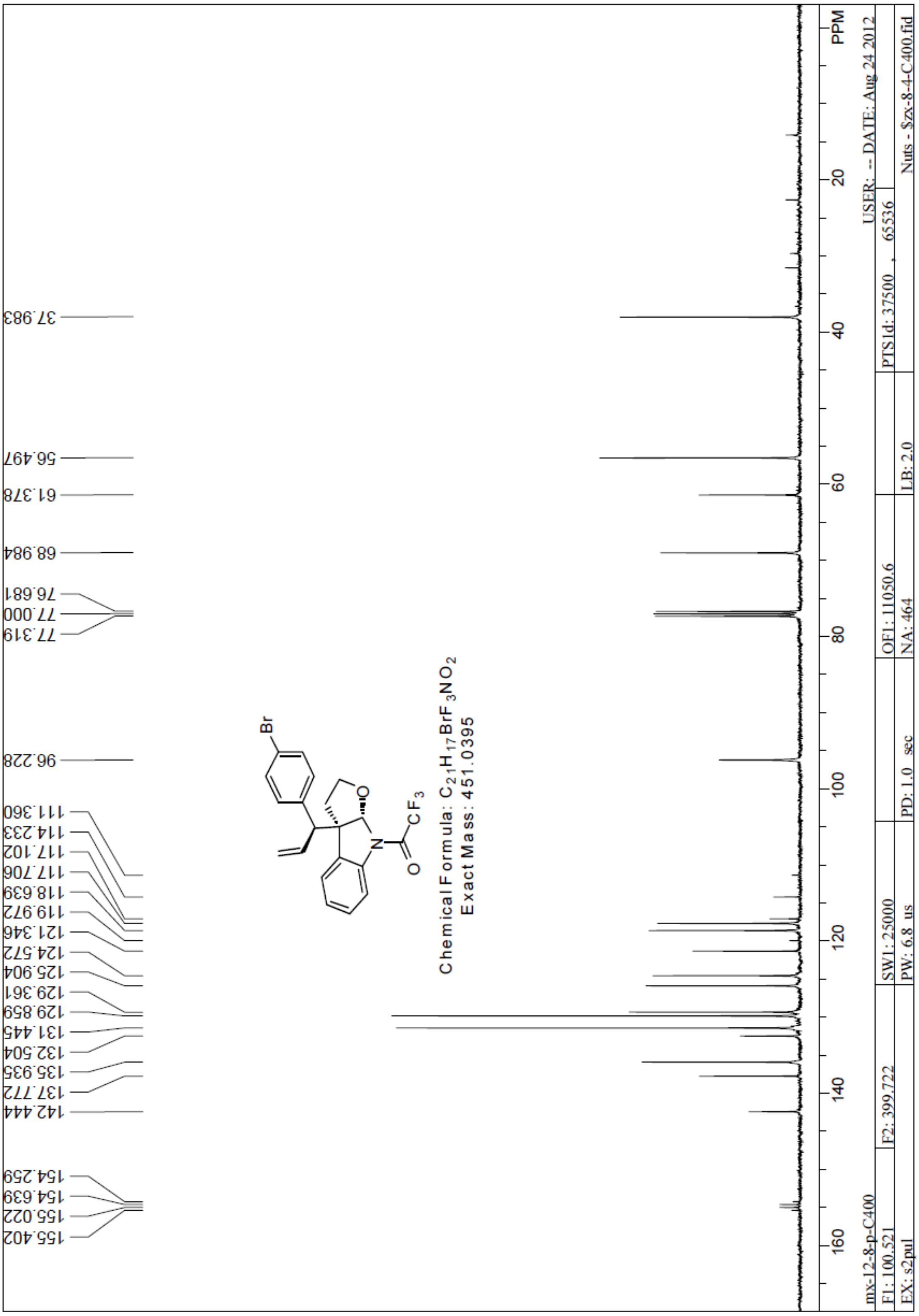

Compound **6**’s ^{19}F NMR Spectra

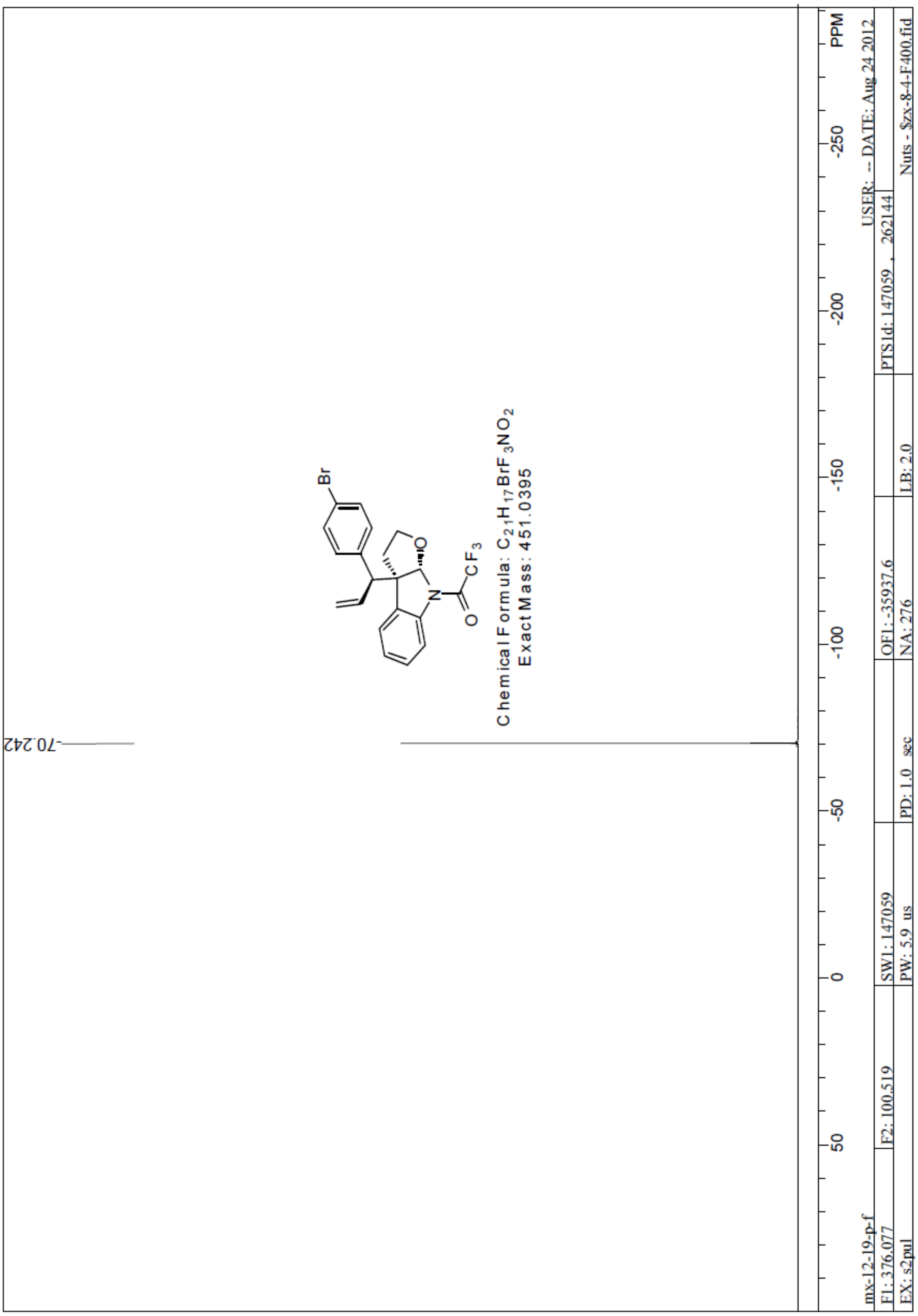

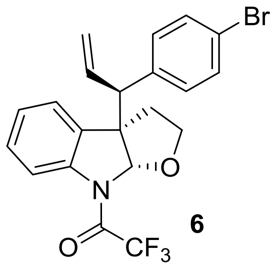

6

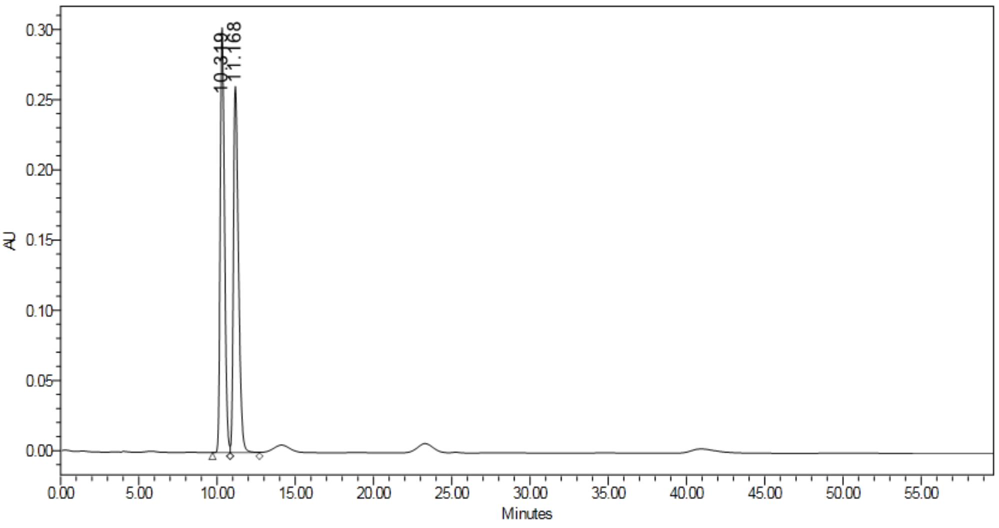

	RT (min)	Area (µV*sec)	% Area	Height (µV)	% Height
1	10.319	5815771	49.80	302720	53.71
2	11.168	5862061	50.20	260935	46.29

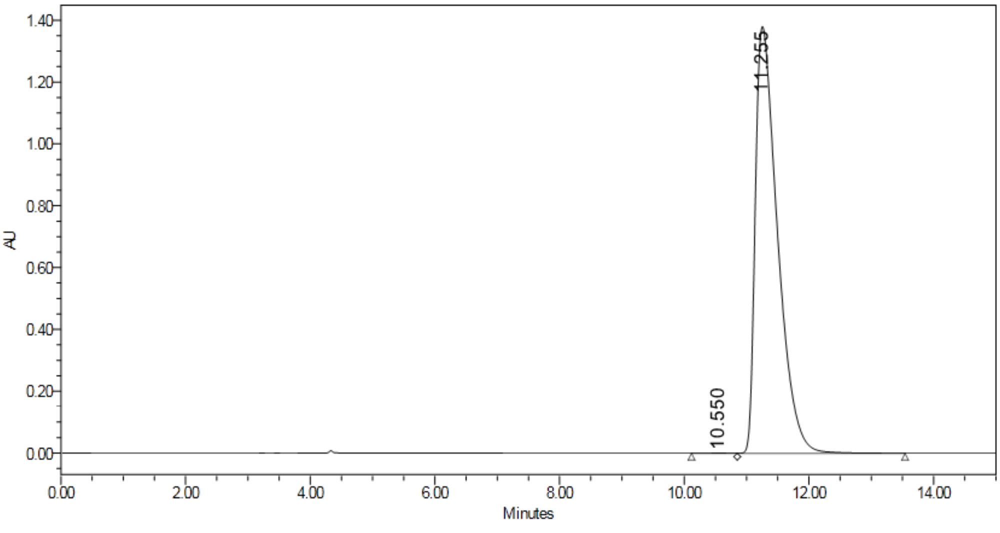

	RT (min)	Area (µV*sec)	% Area	Height (µV)	% Height
1	10.550	9359	0.03	447	0.03
2	11.255	34057631	99.97	1380660	99.97
